# Matching Helical and Supramolecular Chirality in a Perylene Diimide Dimer: Impact on (Chir)Optical Properties

**DOI:** 10.1021/jacs.6c11478

**Published:** 2026-09-02

**Authors:** Mya Kotecha, Denis Hartmann, Artemijs Krimovs, Oussama Errida, Tiberiu-M. Gianga, Georgia R.F. Orton, Giuliano Siligardi, Robert Pal, Giovanni Bressan, Timothy A. Barendt

**Affiliations:** † 1724 University of Birmingham, School of Chemistry, Birmingham B15 2TT, U.K.; ‡ 3057 University of Durham, Dept. Of Chemistry, Durham DH1 3LE, U.K.; § Diamond Light Source, Harwell Sci. & Innovation Campus, Didcot OX11 0DE, U.K.; ∥ 14295 University of East Anglia, School of Chemistry, Norwich NR4 7TJ, U.K.

## Abstract

Understanding the interplay between different forms of chirality in organic materials is critical for tuning and optimizing their (chir)­optical properties. There is a growing interest in chiral chromophores such as bay-substituted perylene diimides (PDIs) due to their intrinsic helicity (***M***/***P***) which, at a higher level of organization, can also generate supramolecular chirality (***M***_***s***_/***P***_***s***_). The relationship between helical and supramolecular chirality governs interchromophore coupling, rotatory strengths, and subsequently circular dichroism (CD) and circularly polarized luminescence (CPL) spectra. Herein, we report the first PDI dimers that match helical and supramolecular chirality, e.g., ***PP***–***P***_***s***_. In agreement with theory, this chiral synergy contributes to enhanced CD and broadband CPL with high CPL brightness, properties of significant value for optical filtering in next-generation chiral sensors and displays.

## Graphical abstract

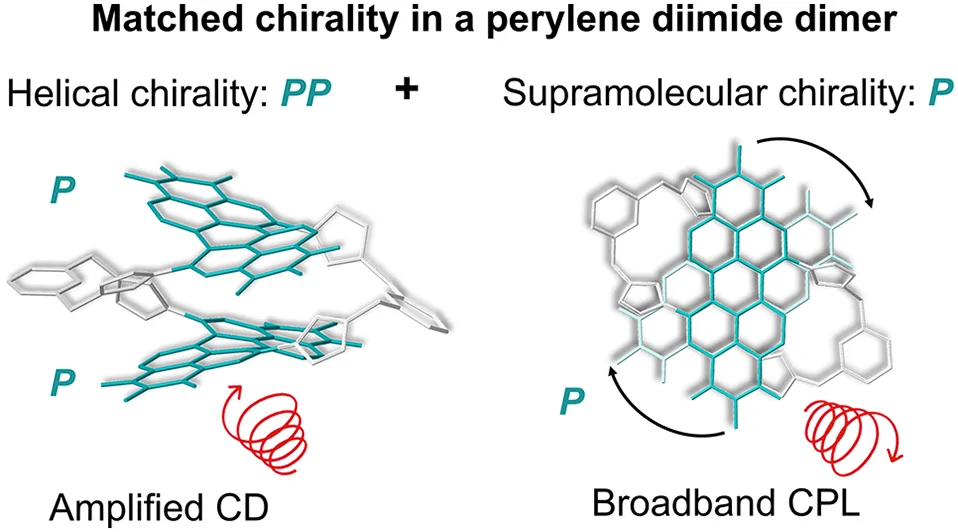

## Introduction

Chirality is a fundamental property that governs the interaction of materials with circularly polarized light at both the molecular and supramolecular level.^1^ In many cases, chiral organic materials comprise a planar chromophore functionalized with a chiral group which, while often generating only weak molecular chiroptical responses,^2^ directs the formation of extended chiral assemblies with amplified circular dichroism (CD) and/or circularly polarized luminescence (CPL).^3–5^ Alternatively, a burgeoning field focuses on twisted aromatics,^6^ where helical distortion of the aromatic framework affords pronounced molecular chiroptical properties, prominent examples including helicenes,^7^ twisted acenes^8^ and warped nanographenes.^9^

Despite growing interest in twisted chromophores,^10^ our understanding of chiral synergy effects in systems containing multiple hierarchical sources of chirality remains limited. In particular, understanding how intrinsic helicity (i.e., ***M***/***P***) influences supramolecular chirality^11^ (***M***_***s***_/***P***_***s***_) is critical, since these chiral arrangements govern excitonic coupling and, in turn, (chir)­optical properties.^12,13^ This includes control over the intensity (*g*_abs/lum_),^14^ purity (monosignate),^15^ and bandwidth (Δλ)^16^ of the CD/CPL response, as well as the CPL brightness (*B*_CPL_).^17^ Monosignate broadband (Δλ ≥ 200 nm) CD is unusual for small molecules^18^ and, to the best of our knowledge, analogous broadband CPL is often limited to polymeric materials.^16,19,20^ Such properties are desirable for applications including chiroptical imaging, security inks and lighting.^21–23^

Perylene diimides (PDIs) are an important class of fluorescent chromophores^18^ and are widely employed as building blocks for electronic and/or photonic materials^24–26^ due to their tunable redox and photophysical properties, accessible through core functionalization.^27^ Moreover, PDIs are becoming increasingly significant in chiroptics^28^ since substitution at the perylene bay regions induces helical distortion of the aromatic core (***M***/***P***).^29^ Supramolecular structure–property relationships are frequently investigated using PDI oligomers^30,31^ which, in the case of helical PDIs, include bis-PDI macrocycles due to chromophore preorganization.^32–34^

In this context, configurational stability is essential to enable the isolation and characterization of individual PDI dimer stereoisomers. We previously reported a chirally locked bis-PDI macrocycle, **3** (Figure 1a), in which the PDI helicity (e.g., ***MM***) is mismatched with the dimer's supramolecular chirality (***P***_***s***_).^35^ Alternative dimers have colinear PDIs, so do not exhibit supramolecular chirality.^34,36,37^ Although chiral synergy has recently been explored in spiro-fused PDI helicenes,^38^ the fundamental question of how alignment between helical and supramolecular chirality influences (chir)­optical properties remains unresolved.

**Figure 1:**
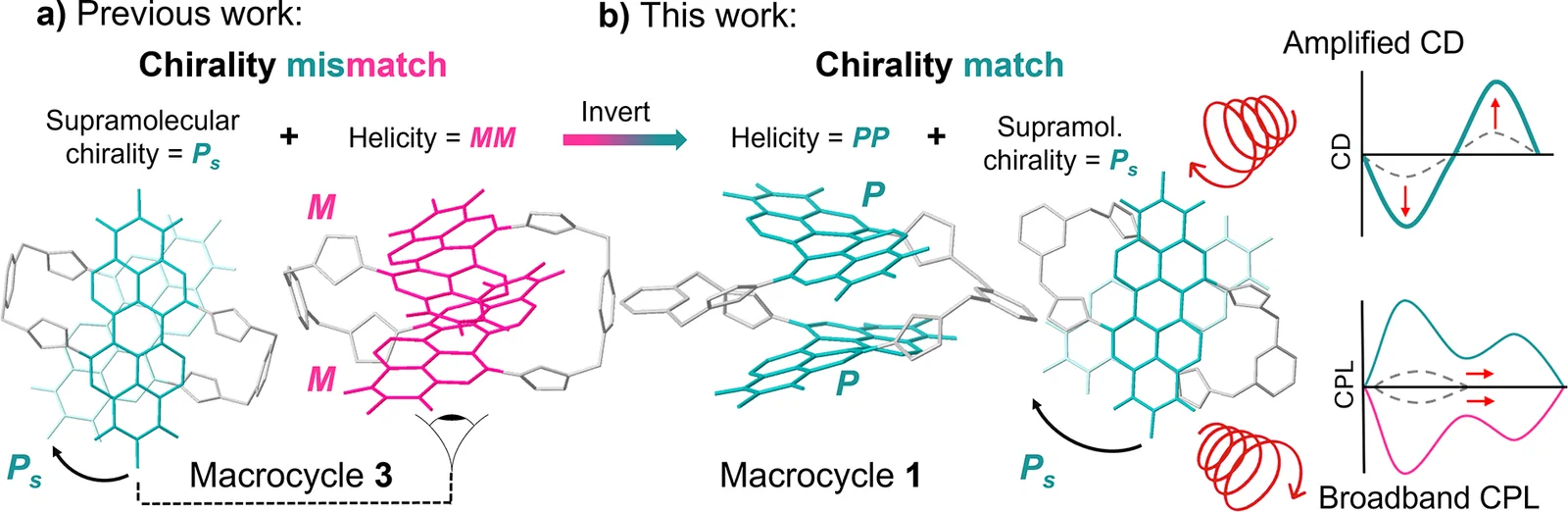
**a)** A previous bis-PDI macrocycle (**3**) in which PDI helicity and supramolecular chirality are mismatched, **b)** a new bis-PDI macrocycle (**1**) with matched chirality and the impact on the dimer’s chiroptical properties.

Herein, we report a new macrocycle which, for the first time, matches helical and supramolecular chirality in a PDI dimer, i.e., ***PP***–***P***_***s***_ and ***MM***–***M***_***s***_ (Figure 1b). Relative to our previous macrocycle design (Figure 1a),^35^ matched chirality was achieved through linker contraction, replacing *p*-aryl with *m*-aryl linkers, which redirects the PDI’s (1,2,3-triazole) bay substituents into the macrocycle cavity to alleviate ring strain.^39^ This inverts PDI helicity^40^ while preserving supramolecular chirality (Figure 1b). Furthermore, the use of *m*-pyridyl (macrocycle **1**) or *m*-xylyl (**2**) linkers elucidate the role of intramolecular hydrogen bonding in reinforcing the target geometry of the chiral π-stack.

The resulting bis-PDI macrocycles are configurationally stable, enabling all stereoisomers to be isolated and analyzed in both solution and the solid state. PDI homochirality (e.g., ***PP***) is shown to enhance excitonic coupling relative to the heterochiral dimer (***MP***). Moreover, supported by theory, matching PDI helicity with supramolecular chirality in macrocycle **1** (e.g., ***PP***–***P***_***s***_; Figure 1b) is an important contributor to the dimer’s amplified CD response relative to mismatched **3** (***MM***–***P***_***s***_; Figure 1a). Chiral synergy is also found to underpin strong, broadband and monosignate CPL spectra of macrocycle **1**, with exceptional brightness arising from the interaction between bright local excited and excimer states, revealed by ultrafast spectroscopy.

## Results and Discussion

### Synthesis and Characterization

The bis-PDI macrocycles with *m*-pyridyl (**1**) or *m*-xylyl (**2**) linkers were prepared by multistep syntheses (Figure 2a), via PDI monomer **5** or **6**, as detailed in the Supporting Information (SI), Section 1. Importantly, two distinct PDI dimer macrocycles were obtained upon purification by preparative thin-layer chromatography, consistent with the isolation of homochiral (***MM*** and ***PP***) and heterochiral (***MP***) stereoisomers. Indeed, chiral HPLC (SI, Section 5) resolved two enantiomers from the homochiral samples, which were assigned by a combination of CD spectroscopy and computation (SI, Section 10). All macrocycles were characterized by ^1^H (Figure 2b) and ^13^C NMR spectroscopy and high-resolution mass spectrometry (SI, Section 1). The distinct ^1^H NMR spectra of homochiral (***MM***/***PP***) and heterochiral (***MP***) dimer macrocycles confirmed their diastereomeric relationship (Figure 2b).

For both **1** and **2**, ^1^H NMR spectroscopy evidenced the slow interconversion of ***MP*** into ***MM***/***PP*** at 100 °C (Figure 2b). This demonstrates the thermodynamic stability of homochiral over heterochiral stereoisomers, likely due to the chiral complementarity between the two PDI units in the former.

However, all macrocycle stereoisomers are configurationally stable at room temperature, with negligible change in the CD spectra of ***MM***/***PP*** enantiomers after three months (Figures S6.6 and S6.7). This was confirmed by chiral HPLC, with reinjection of pure ***MM***/***PP***/***MP*** samples giving a single peak on the chromatogram (Figures S5.2 and S5.5). The free energy barrier to interconversion (Δ*G* ^‡^) was determined by time-course CD spectroscopy at 112 °C, giving Δ*G* ^‡^ = 131 kJ mol^–1^ (*t*_1/2_ = 6.5 h) and 122 kJ mol^–1^ (0.5 h) for the *m*-pyridyl (**1**) and *m*-xylyl (**2**) macrocycles respectively (SI, Section 6). The difference is discussed below, yet both values are significantly larger than that of a dynamic *p*-xylyl linked macrocycle analogue with Δ*G* ^‡^ ≈ 90 kJ mol^–1^.^32^ This is likely due to the reduced macrocyclic ring size (by just two atoms) in macrocycles **1** and **2**, and the more acute angle (120° vs 180°) imposed by their *m*-aryl linkers. Van‘t Hoff analysis of variable temperature CD spectra revealed that the enthalpic barrier to stereoisomer interconversion for *m*-aryl linked macrocycle **1** (Δ*H* ^‡^ = 218 kJ mol^–1^; Figure S6.5) is significantly larger than that in the *p*-aryl analogue (81 kJ mol^–1^).^32^

## Dimer Geometry

We grew single crystals suitable for X-ray diffraction (XRD) and obtained structures of three macrocycles; specifically heterochiral (**1-** ***MP*** and **2-** ***MP***) and homochiral (**1-** ***MM***/***PP***) dimers, including racemic and enantiopure structures for the latter (Figure 3 and SI, Section 4). A cofacial π-stacked dimer was evident from the crystal structure of **1-** ***MM***/***PP*** (Figure 3), with a PDI–PDI separation of *d*_π–π_ = 4.3 Å, sufficient for excitonic coupling (vide infra).^41^ Importantly, both racemic and enantiopure crystal structures of **1-** ***MM***/***PP*** show that the PDI’s helical chirality (***M***/***P***, dihedral β = 18°) and the dimer’s supramolecular chirality (***M***_***s***_/***P***_***s***_, twist α = 60°) are matched, i.e., ***MM*** gives ***M***_***s***_ and ***PP*** gives ***P***_***s***_. This dimer geometry is distinct to previous twisted PDI-containing dimer macrocycles, where supramolecular chirality is either absent^34,36,37^ or mismatched with PDI helicity.^32,35^

In solution (1,1,2,2-tetrachloroethane-d_2_), the ^1^H NMR spectrum of **1-** ***MM***/***PP*** is notable for the large upfield shift of the triazole proton signal (H_d_, Figure 2b) in comparison to the PDI monomer **5** (Δδ = 1.9 ppm) and the other bis-PDI macrocycles (∼1.3 ppm). This is indicative of ring-current effects arising from the close approach of the PDI π-surfaces in **1-** ***MM***/***PP***,^32,42^ i.e., intramolecular π–π stacking, and in line with the dimer geometry seen in the X-ray crystal structure (Figure 3).

The crystal structure of **1-** ***MM***/***PP*** also revealed four intramolecular hydrogen bonds between the pyridine and triazole heterocycles in the macrocycle linkers (average CH···N distance = 3.1 Å, Figure 3). Such interactions promote the formation of a homochiral, cofacial PDI π-stacked geometry. These interactions were found to persist in solution since, upon heating **1-** ***MM***/***PP***, the ^1^H NMR signal for the triazole protons (H_d_) remains broader than the other peaks, characteristic of their involvement in hydrogen bonding (Figure S2.2).

**Figure 2:**
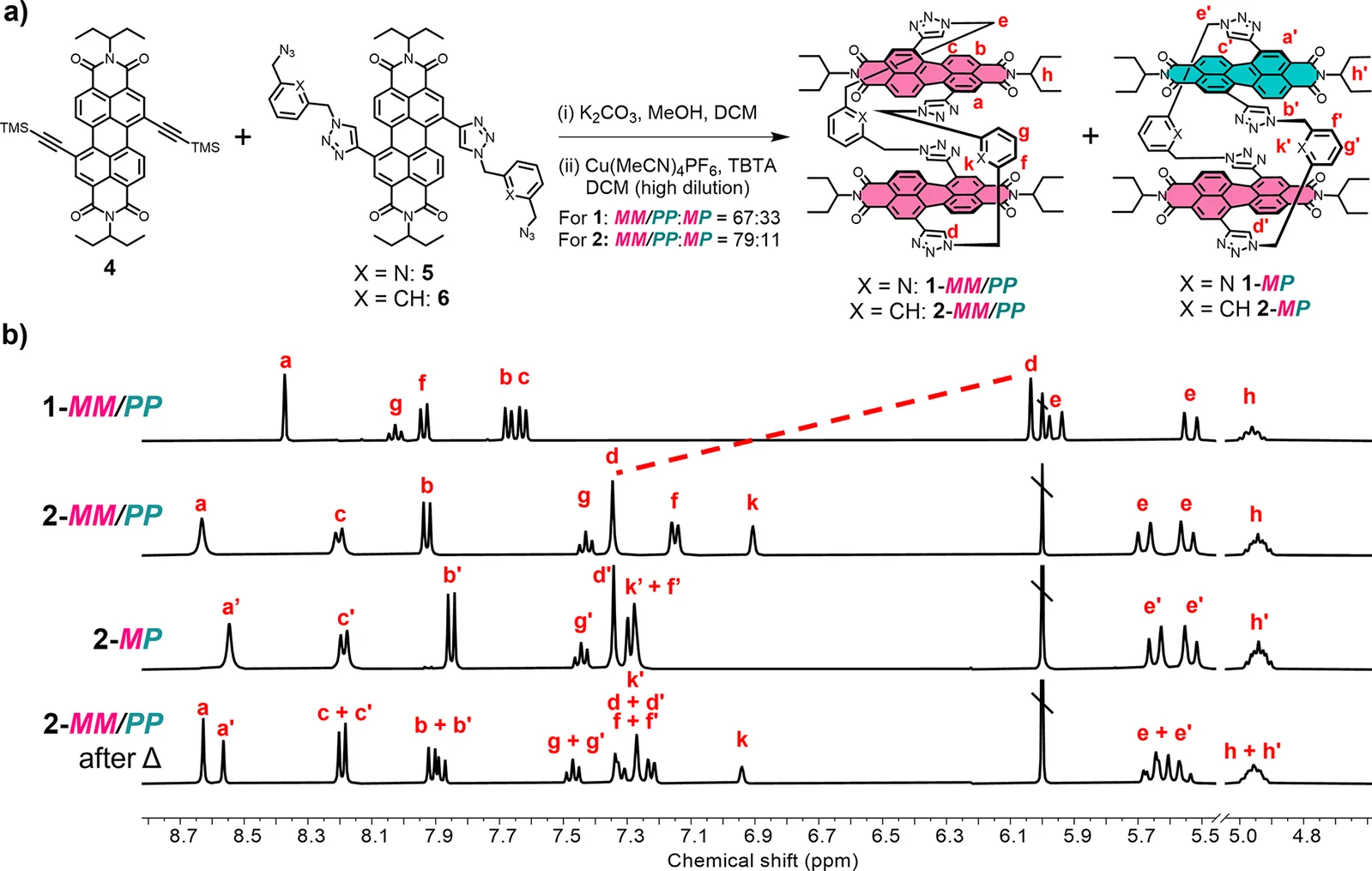
**a)** Synthesis of bis-PDI macrocycles **1-*MM***/***PP***/***MP*** (*m*-pyridyl) and **2-*MM***/***PP***/***MP*** (*m*-xylyl), with (′) used to indicate protons of the ***MP*** stereoisomer and TBTA = tris­(benzyltriazolylmethyl)­amine, **b)** comparison of the ^1^H NMR spectra (373 K, 400 MHz, 1,1,2,2-tetrachloroethane-d_2_) of a selection of macrocycle stereoisomers, including **2-*MM***/***PP*** after heating at 100 °C for 8 h.

**Figure 3:**
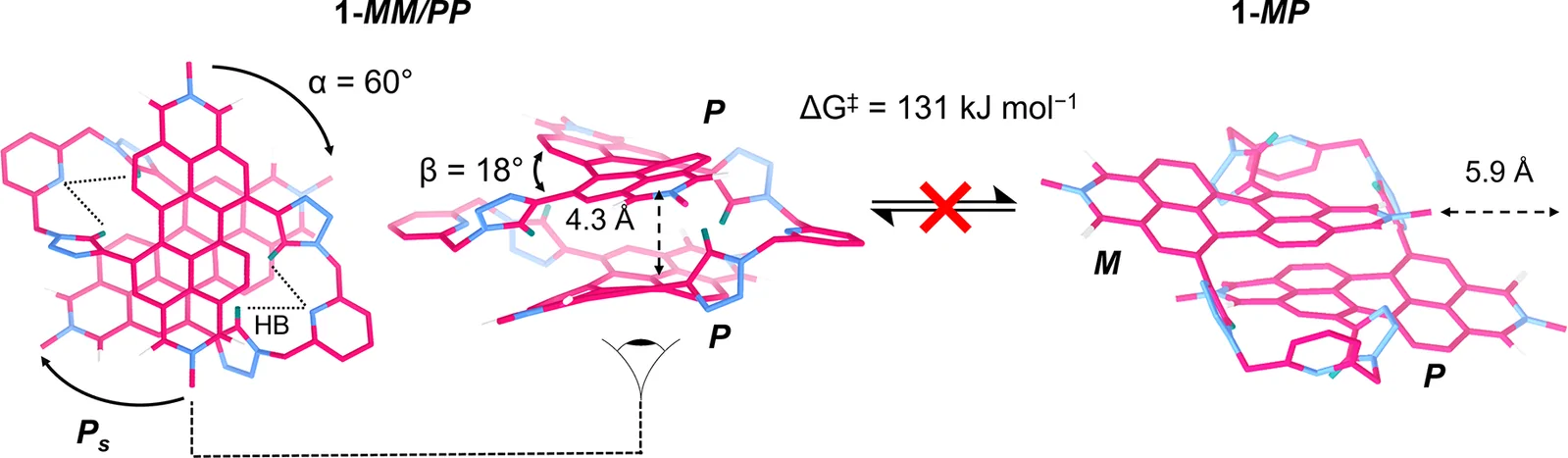
X-ray crystal structures of bis-PDI macrocycles **1-** ***MM***/***PP*** (homochiral) and **1-** ***MP*** (heterochiral). HB = hydrogen bond and *d*_π–π_ = 4.3 Å in **1-** ***MM***/***PP***.

Furthermore, ^1^H–^1^H ROESY NMR of **1-** ***MM***/***PP*** reveals a H_c_↔H_d_ correlation (Figure S2.4) consistent with intramolecular hydrogen bonding that orients H_d_ toward the macrocycle cavity, as seen in the crystal structure (Figures 3 and S2.5). We also employed the noncovalent interaction (NCI) index method^43^ to analyze a structure of **1-** ***PP*** obtained from DFT optimization of its crystal structure (B97–3c^44^/def2-TZVP^45^ with implicit solvation in toluene [conductor-like polarizable continuum model]; SI, section 10). This analysis confirmed the presence of intramolecular pyridine–triazole hydrogen bonding alongside π–π stacking interactions between the PDI units (Figure S10.11).

Alongside ^1^H NMR spectroscopy (Figure S2.3), ^1^H–^1^H ROESY and DOSY NMR spectroscopy provide further evidence that **1-** ***MM***/***PP*** and **1-** ***MP*** adopt the distinct dimer geometries observed in their crystal structures (Figure 3). For **1-** ***MM***/***PP***, an H_a_↔H_c_ cross-peak is consistent with the rotational displacement of the PDI units within the cofacial π-stack that brings these protons into close spatial proximity (Figures S2.4 and S2.5). In contrast, **1-** ***MP*** exhibits an H_a_↔H_b_ cross-peak, reflecting the slipped π-stacked geometry in which long-axis displacement brings these protons closer together (Figures S2.8 and S2.9). Furthermore, **1-** ***MP*** diffuses slower than **1-** ***MM***/***PP*** (*D* = 1.303 [±0.008] vs 1.472 [±0.004] × 10^–10^ m^2^ s^–1^; Table S2.6), indicating a larger hydrodynamic radius that is consistent with its more elongated structure (Figure 3).

In the absence of a crystal structure for the homochiral *m*-xylyl macrocycle **2-** ***MM***/***PP***, we used computational chemistry (CREST code^46^ and the gfn2 tight-binding method^47^) to predict the lowest energy conformer of **2-** ***PP*** (Figure S10.12). The calculated structure features a cofacial π-stacked arrangement of the PDI units, similar to that of **1-** ***MM***/***PP***. In addition to the ^1^H NMR data (Figures 2b and S2.3), ^1^H–^1^H ROESY and DOSY NMR spectroscopy provide further support for this geometry in solution. Specifically, multiple through-space proton correlations in the ROESY spectrum agree with the calculated structure of **2-** ***PP*** (Figures S2.6 and S2.7), while **2-** ***MM***/***PP*** exhibits a smaller diffusion coefficient than PDI monomer **5** (*D* = 1.378 [±0.002] vs 1.708 [±0.002] × 10^–10^ m^2^ s^–1^; Table S2.6) and a value similar to that of macrocycle **1-** ***MM***/***PP*** (1.472 [±0.004] × 10^–10^ m^2^ s^–1^), consistent with a π-stacked dimeric structure.

Like **1-** ***PP***, the **2-** ***PP*** PDI dimer is also found to have matched helical–supramolecular chirality, i.e., ***PP***–***P***_***s***_. However, only a single CH···N hydrogen bond is found in each linker, occurring between the two triazole groups, which is expected to be a weaker interaction than that in **1-** ***MM***/***PP*** due to the lower Lewis basicity of the 1,2,3-triazole compared to pyridine. Furthermore, NCI index analysis identified PDI–PDI π-stacking as the dominant noncovalent interaction in the calculated structure of **2-** ***PP*** (Figure S10.11). This analysis was supported by ^1^H NMR spectroscopy in solution (SI, Section 2). Relative to **1-** ***MM***/***PP***, the triazole proton H_d_ is shifted downfield in **2-** ***MM***/***PP*** (Δδ = 1.3 ppm; Figure 2b), indicating reduced shielding by the neighboring PDI π-surface.^42^ This observation is consistent with the calculated structure of **2-** ***PP*** (Figure S10.12), in which H_d_ may be directed away from the PDI because, unlike in **1-** ***MM***/***PP***, it is not involved in intramolecular hydrogen bonding to the *m*-aryl group in the linker. These differences in intramolecular hydrogen bonding may explain the larger Δ*G* ^‡^ measured for *m*-pyridyl macrocycle **1** compared to *m*-xylyl analogue **2**.

## Dimer Excitonic Coupling

Building on the XRD and NMR spectroscopic analysis, we used photophysical techniques to determine the excitonic coupling of the macrocyclic PDI dimer stereoisomers in solution and relate this to their geometry. The UV–vis spectrum of macrocycle **1-** ***MM***/***PP***, in both 1,1,2,2-tetrachloroethane and toluene (Figures 4a and S3.3), is consistent with cofacial π-stacking interactions and excitonic coupling between the PDI units. The λ_max_ of the lowest energy absorption band, S_0_ → S_1_, is blue-shifted from 555 to 544 nm, and exhibits a larger vibronic peak ratio (0–1/0–0 > 1), relative to PDI monomer **5** in toluene (Figure 4d), characteristic of H-type coupling^18^ in the PDI dimer (*J*_*Tot*_ = +463 cm^–1^,^48^ SI Section 11).^49^ To further demonstrate the role of intramolecular (CH···N) hydrogen bonding in stabilizing a dimer geometry with strong H-type excitonic coupling, we measured the UV–vis spectrum of **1-** ***MM***/***PP*** following protonation of the *m*-pyridyl group(s) (Figure S3.4). The new spectrum strongly resembles that of PDI monomer **5** (Figure 4d), indicating weaker coupling.

The UV–vis spectrum of heterochiral macrocycle **1-** ***MP*** in toluene shows a vibronic peak ratio closely resembling the PDI monomer **5** (Figures 4b, d) and so exhibits weaker excitonic coupling (*J*_*Tot*_ = +119 cm^–1^). This is due to opposing PDI helical chirality in the dimer which, as suggested by the crystal structure of **1-** ***MP*** (Figure 3), arises from a long-axis displacement of the chromophores (*d* = 5.7 Å), reducing *J*_*Tot*_.^50^

Computational chemistry was used to further validate that the distinct geometries of **1-** ***MM***/***PP*** and **1-** ***MP*** (Figure 3) are consistent with the differences in their excitonic coupling. For both macrocycles, the lowest energy conformers predicted from a conformer search (vide supra) resemble the respective crystal structure. Moreover, the predicted UV–vis and CD spectra of these conformers (TD-ωB97x^51^/def2-SVP;^45^ SI, Section 10) agree with those measured for the PDI dimers in toluene. Specifically, a larger Davydov splitting is predicted for **1-** ***MM***/***PP*** relative to **1-** ***MP*** (Tables S10.1 and S10.2) which is consistent with its stronger H-type coupling arising from the cofacial (vs slipped) π-stacked geometry.

**Figure 4:**
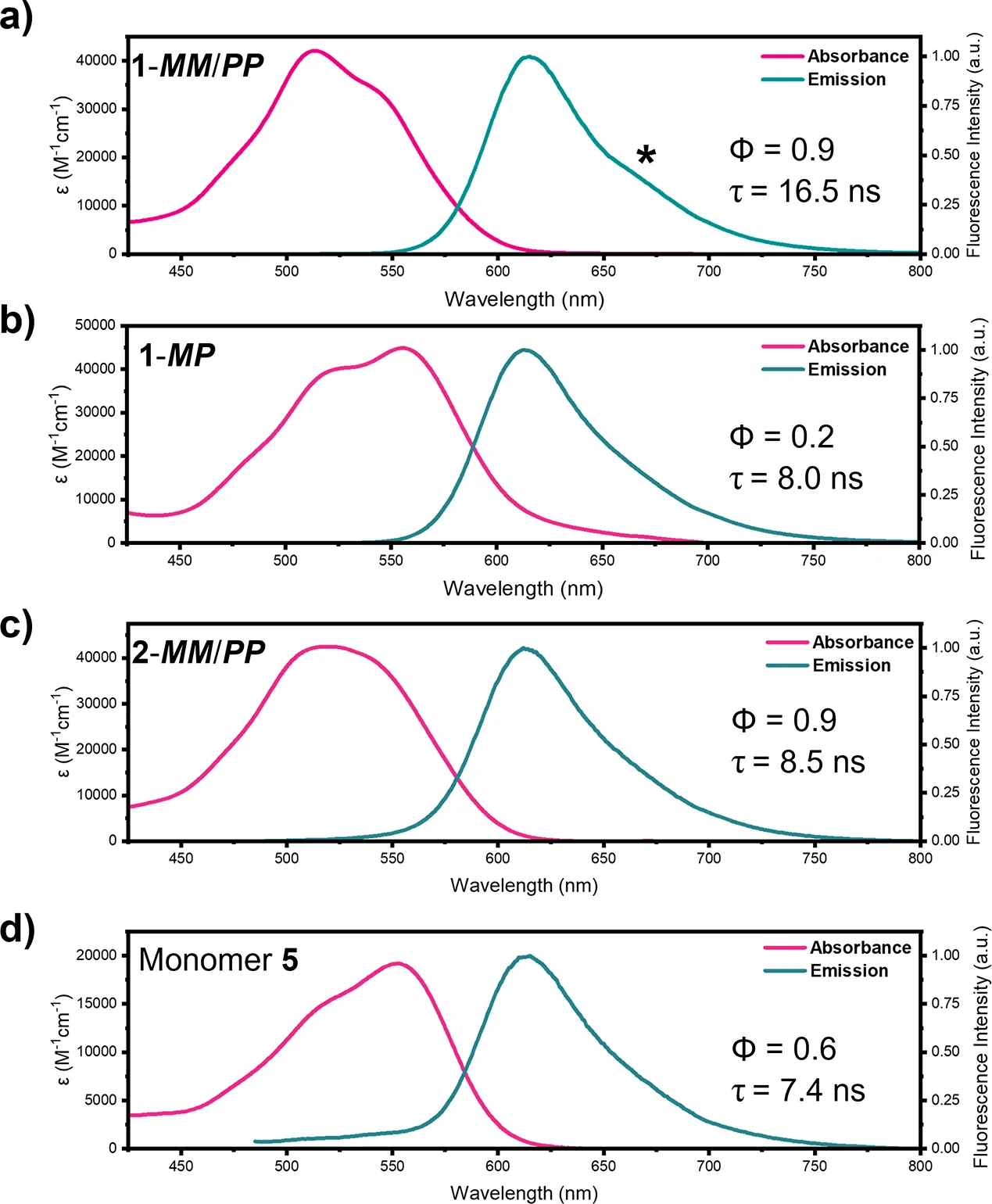
UV–vis absorption (11 μM, pink) and fluorescence emission (11 μM, λ_ex_ = 450 nm, teal) spectra in toluene of bis-PDI macrocycles, **a)** **1-** ***MM***/***PP*** (* = EX shoulder, vide infra), **b) 1-** ***MP***, **c) 2-** ***MM***/***PP***, and **d)** PDI monomer **5**.

The UV–vis spectra of *m*-xylyl macrocycles **2-** ***MM***/***PP*** and **2-** ***MP*** reveal weaker PDI–PDI coupling than **1-** ***MM***/***PP*** (Figures 4a,c and S3.5), while the loss of vibronic structure is consistent with ground state inhomogeneity arising from reduced intramolecular hydrogen bonding. This is supported by ROESY and DOSY NMR spectroscopy (SI, Section 2), with **2**-***MM***/***PP*** exhibiting fewer through-space correlations between PDI and linker protons than **1-** ***MM***/***PP*** (Table S2.1), as well as a smaller diffusion coefficient (Table S2.6), both consistent with greater conformational freedom in the *m*-xylyl macrocycle. Notably, the *m*-pyridyl and *m*-xylyl linkers are not expected to directly contribute to the low-energy electronic transitions of the PDI dimer since, for both **1-** ***MM***/***PP*** and **2-** ***MM***/***PP***, the frontier molecular orbitals associated with these transitions are almost exclusively localized on the ditriazole PDI units (Figures S10.7–S10.10). The crystal structure of **2-** ***MP*** (Figure S4.5) reveals the same long-axis displacement as **1-** ***MP*** (5.7 Å), which would also explain its weaker excitonic coupling.

The dependence of excitonic coupling on dimer stereochemistry is further evidenced from fluorescence spectroscopy in toluene. While the spectrum of **1-** ***MP*** (Figure 4b) is akin to that of PDI monomer **5** (Figure 4d), with maxima at 610 nm for both species, evidencing emission from the local excited (LE) state, the strongly coupled homochiral dimer **1-** ***MM***/***PP*** exhibits an additional red-shifted fluorescence shoulder at ∼650 nm that is broader and of lower intensity relative to the LE band (Figure 4a). Based on the longer, wavelength-independent fluorescence lifetime of **1-** ***MM***/***PP*** (τ = 16.5 ns, Figures S9.1 and 9.2), relative to **1-** ***MP*** (8.0 ns) and monomer **5** (7.4 ns), we propose that this shoulder is due to emission from an excimer (EX) population. Importantly, the fluorescence shoulder is not observed in **1-** ***MP***, monomer **5** or *m*-xylyl macrocycles **2** (vide infra).

The position of the fluorescence shoulder for **1-** ***MM***/***PP*** is consistent with previously reported PDI EXs.^52,53^ It has no concentration dependence (Figure S3.6), evidencing intramolecular EX formation.^54^ We can also exclude impurities or multiple ground states as an explanation for the shoulder since the excitation and absorption spectra of **1-** ***MM***/***PP*** are in excellent agreement (Figure S3.7). Likewise, vibronic progression is ruled out since the shoulder is observed in neither monomer **5** (Figure 4d), nor dimer **1-** ***MP*** (Figure 4b). The latter also demonstrates the importance of chiral complementary for EX population.

In line with shorter fluorescence lifetimes (∼8 ns) and a lack of EX shoulder in their fluorescence spectra (Figures 4c and S3.5), EX formation is negligible in the *m*-xylyl macrocycles (**2**). Here, the weaker intramolecular hydrogen bonding in **2-** ***MM***/***PP*** is not sufficient to promote a strongly coupled, cofacial PDI dimer geometry. These findings are underpinned by the following femtosecond transient absorption (fsTA) spectroscopy studies.

## Transient Absorption Spectroscopy

We used fsTA spectroscopy to support our interpretation of the photophysics of the dimers. The fsTA measurements were performed on toluene and DMSO (SI, Section 9) solutions of the PDI dimer macrocycles (**1-** ***MM***/***PP***, **2-** ***MM***/***PP*** and **1-** ***MP***) and monomer **5** (Figure S9.3) following ∼100 fs, 545 nm excitation, using a previously described setup.^55^ At *T* = 200 fs, all dimers exhibit qualitatively similar spectral features, consisting of a broad negative signal spanning 500–650 nm arising from the combined ground-state bleach (GSB) and stimulated emission (SE), alongside a broad excited-state absorption (ESA) extending from ∼650 nm to beyond 1100 nm. Differences between PDI dimers and monomer are most apparent in the 700–900 nm region: while **1-** ***MM***/***PP*** (Figure 5a) shows a relatively featureless, sloping ESA peaking at 900 nm, closely resembling the PDI monomer **5** (Figure S9.3), **2-** ***MM***/***PP*** (Figure 5e) exhibits two maxima (∼700 and ∼900 nm), while **1-** ***MP*** (Figure 5c) displays an additional shoulder at ∼800 nm.

In apolar toluene, distinct relaxation pathways emerge over the ps time scale. For **2-** ***MM***/***PP*** and **1-** ***MP*** (Figures 5c,e) a reshaping of the GSB/SE band into a more structured profile, with minima at ∼560 and ∼610 nm, matching the absorption and emission maxima, is observed. Concomitantly, the ESA amplitude decreases by ∼15–20%. In **1-** ***MP***, this relaxation is further accompanied by a redshift of the ESA shoulder initially located at ∼800 nm.

In contrast, **1-** ***MM***/***PP*** (Figure 5a) does not exhibit a SE redshift. Instead, a new, weak 600–650 nm positive feature emerges, while the 700–900 nm (LE) ESA band remains largely unchanged. Macrocycle **1-** ***MM***/***PP*** shows slow relaxation, whereas **2-*MM***/***PP*** and **1-** ***MP*** exhibit faster decay, and continued ESA evolution.

The fsTA data sets were analyzed by global fitting using Glotaran,^56^ assuming sequential first-order evolution through *n* states with increasing time constants τ_*n*__+1_ > τ_*n*_. Two or three components were required to achieve good fits. Corresponding evolution associated difference spectra (EADS) are shown in Figure 5b,d,f.

For **1-** ***MM***/***PP*** (Figure 5b), the initial spectrum is dominated by a strong ESA centered at 900 nm, assigned to S_1_ → S_n_ from the LE state promptly populated upon photoexcitation. The fastest 0.3 ps component is characterized by the appearance of a weak ESA feature in the 620–650 nm region and partial SE quench, and is assigned to EX formation, with negligible changes elsewhere. This is followed by a 24 ps component with further EX ESA rise time, but nearly identical spectral profile, suggesting minor evolution within a single potential energy surface (PES). This component decays to the ground state with a 16.5 ns lifetime (fixed to match time-resolved fluorescence data).

In contrast to fully formed PDI excimers, which show a broad, flat ESA extending from ∼600 nm into the NIR, together with quenching of the LE ESA and SE,^57–62^ **1-*MM***/***PP*** retains the ∼900 nm LE ESA and develops only a weak EX ESA, indicating that evolution toward EX character occurs on a ∼300 fs time scale but remains partial. The high fluorescence quantum yield (Φ = 0.9 for **1-** ***MM***/***PP***) and the dual LE/EX fluorescence and CPL (vide infra) are consistent with this picture, while the excitation and detection wavelength-independent fluorescence lifetimes (Figures S9.1 and S9.2) and excitation spectra (Figure S3.7) argue against parallel excited-state populations and conformational heterogeneity,^63^ placing the LE and EX signatures within a homogeneous excited-state population. Two limiting descriptions are consistent with our data: a dynamical LE ⇄ EX equilibrium established within ∼300 fs of photoexcitation, or a state of intrinsically mixed LE/EX character formed on the same time scale. In both limits, the observable is a sub-picosecond relaxation to a time-independent LE/EX population ratio (i.e., 650/900 nm amplitude ratio), so the two are kinetically degenerate over our accessible delay range. Neither global nor target analysis can distinguish between such two scenarios, which would require an observable sensitive to the LE–EX interconversion barrier. Together with steady-state absorption (and excitation; Figure S3.7) spectra indicating significant H-type excitonic coupling (*J*_*Tot*_ = +463 cm^–1^), this suggests that the relatively large interchromophore distance (*d*_π–π_ = 4.3 Å) and tilt angle (α = 60°, Figure 3) in **1-*MM***/***PP*** promote LE/EX mixing (or equilibration) in a nonpolar environment.

**Figure 5:**
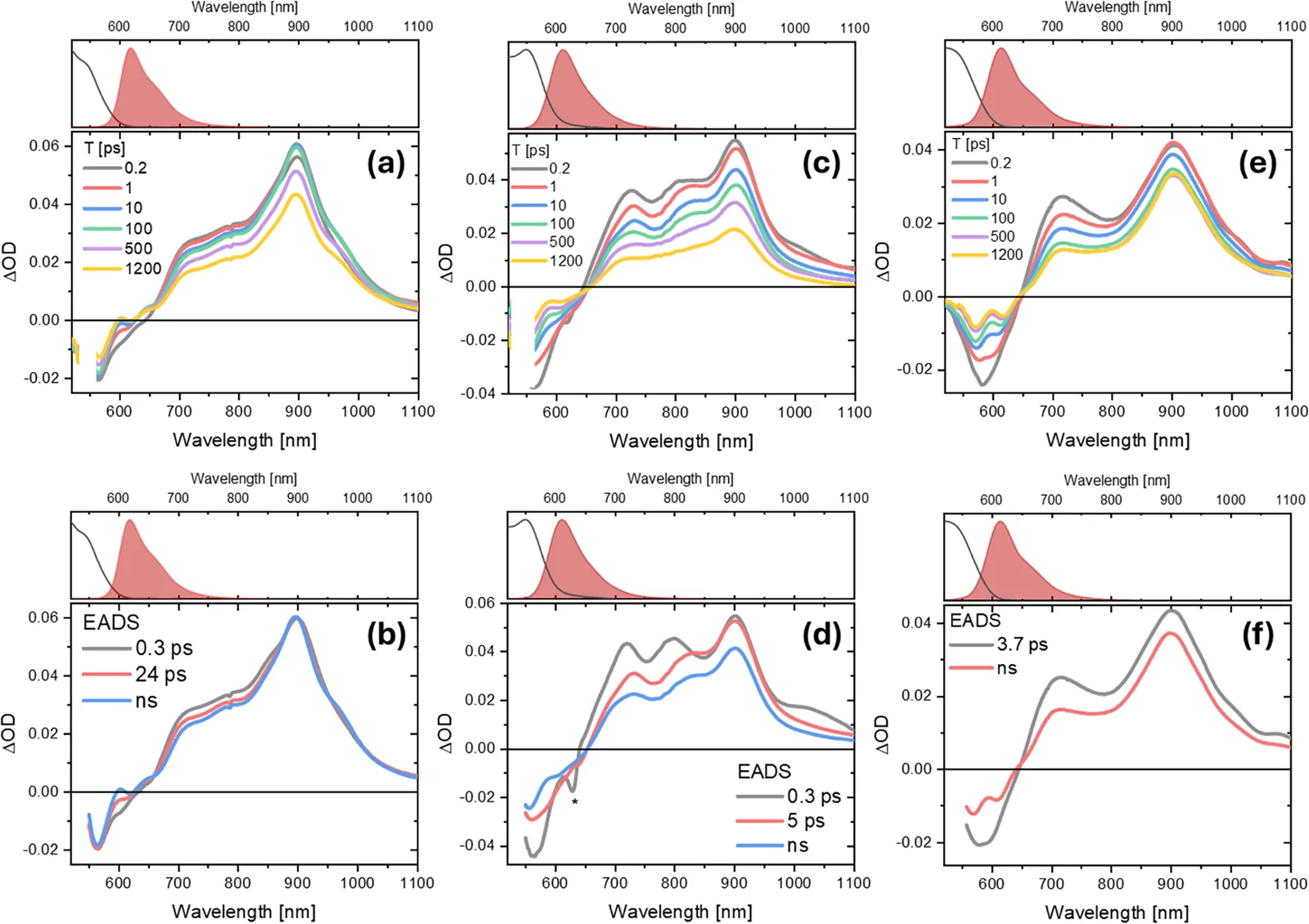
(**a**, **b**) fsTA spectra at selected pump–probe delays and evolution associated difference spectra from global fitting of **1-** ***MM***/***PP*** in toluene. (**c**, **d**) same as (**a**, **b**) for **1-*MP***. The asterisk in (**d**) indicates that the initial spectrum contains an artifact arising from stimulated Raman scattering of the toluene solvent (545 nm pump, ∼3000 cm^–1^ aromatic C–H stretch), contributing around pulse-overlap time. (**e**, **f**) same as (**a**, **b**) for **2-** ***MM***/***PP***. Steady-state absorption (black) and fluorescence emission (red shade) spectra are reproduced from Figure 4 in the top panels.

EX formation in cofacial rylene dimers requires contraction of the interchromophoric distance.^52,60,63,64^ In 1 ***-MM***/***PP***, this pathway is suppressed by the rigid cyclic framework anchored by hydrogen bonding and π–π interactions. Hence, the EX state cannot relax to its lower-energy configuration and remains energetically close (within *kT*) to the LE state. This enables LE–EX interconversion (or mixing), giving rise to tens of ns fluorescence and dual CPL (vide infra).

To verify that the fluorescence/CPL 650 nm shoulder contains LE/EX contributions, we performed fsTA of both macrocycle **1** dimers in polar DMSO (Figure S9.4). In polar environments, EX states are expected to be quenched through symmetry-breaking charge separation (SBCS),^62,65,66^ followed by charge recombination (CR) repopulating S_0_. We observe ps SBCS dynamics in **1** *-**MM***/***PP*** in DMSO, and its steady-state fluorescence no longer exhibits the EX shoulder (Figure S9.4). Dimer **1** ***-*** ***MP*** also shows few ps SBCS and hundreds of ps CR in DMSO (Figure S9.4).

For *m*-xylyl macrocycle **2-** ***MM***/***PP*** in toluene (Figure 5f), the 3.7 ps component shows a SE redshift arising from ditriazole PDI planarization (akin to monomer **5**, Figure S9.3), accompanied by a NIR ESA amplitude reduction. The absence of this SE redshift in **1-** ***MM***/***PP*** may reflect structural constraints on ditriazole PDI planarization imposed by the intramolecular CH···N hydrogen bonding and/or competition between the few ps PDI planarization and the 0.3 ps onset of the LE–EX equilibrium.

In contrast to **1-** ***MM***/***PP***, the absence of EX ESA formation at 620–650 nm for **2-** ***MM***/***PP*** indicates that the photoexcited population remains in a LE state. This assignment is supported by the close similarity of the absorption, fluorescence, and CPL (vide infra) spectra to those of uncoupled PDI monomer **5**, together with a comparable fluorescence lifetime (8.0 vs 7.4 ns).

Similarly, no EX ESA is formed in heterochiral dimer **1-** ***MP*** (Figure 5d) consistent with its weakly coupled absorption spectrum and long axis displacement (5.9 Å). Instead, a ∼0.3 ps component involves decay of the ∼700 and ∼1050 nm ESA and redshift of the ∼800 nm feature, followed by a ∼5 ps amplitude loss with minimal spectral evolution. We assign this fast spectral evolution to structural relaxation within the LE manifold and subsequent population loss via nonradiative pathways, in agreement with its nearly 5-fold lower fluorescence quantum yield (Φ = 0.2 vs 0.9 for **1-** ***MM***/***PP***). Nonradiative relaxation is likely facilitated by increased dimer flexibility, with weaker π–π and hydrogen bonding interactions, due to mismatched PDI helicity.

The presence of the ∼800 nm ESA feature in **1-** ***MP***, and its absence in both **1-** ***MM***/***PP*** and **2-** ***MM***/***PP***, may reflect the reduced symmetry of the heterochiral dimer. A symmetry forbidden S_1_ → S_n_ transition in the homochiral dimers could gain oscillator strength in the less symmetrical heterochiral **1-** ***MP***. Alternatively, the feature could be vibronic in origin, arising from a ∼1390 cm^–1^ Raman-active mode such as a vinyl stretching, more strongly displaced between the S_1_ and S_n_ surfaces in **1-** ***MP*** than in the homochiral dimers. The spectral evolution of the ESA profile would then reflect relaxation of the S_1_ population causing a change in the Franck–Condon factors of such a vibration between S_1_ and S_n_. The present data do not allow us to distinguish between these two scenarios; resolving the origin of this feature would require further computational or resonance Raman characterization, which is beyond the scope of this study.

## Circular Dichroism

Our next goal was to understand how PDI dimer geometry and coupling impacted the chiroptical properties, beginning with CD. For both macrocycles **1** and **2**, the ***MM*** and ***PP*** enantiomers gave the expected mirror image CD spectra in toluene (Figure 6a), with a Cotton effect for the S_0_ → S_1_ transition (∼530 nm), confirming excitonic coupling between the two PDI units.^67^ The stronger excitonic coupling in **1-** ***MM***/***PP***, with synergistic π-stacking and hydrogen bonding, leads to a larger *g*_abs_ (9 × 10^–3^ at 325 nm) than that of **2-** ***MM***/***PP*** (*g*_abs_ = 6 × 10^–3^; Figure 6a).

**Figure 6:**
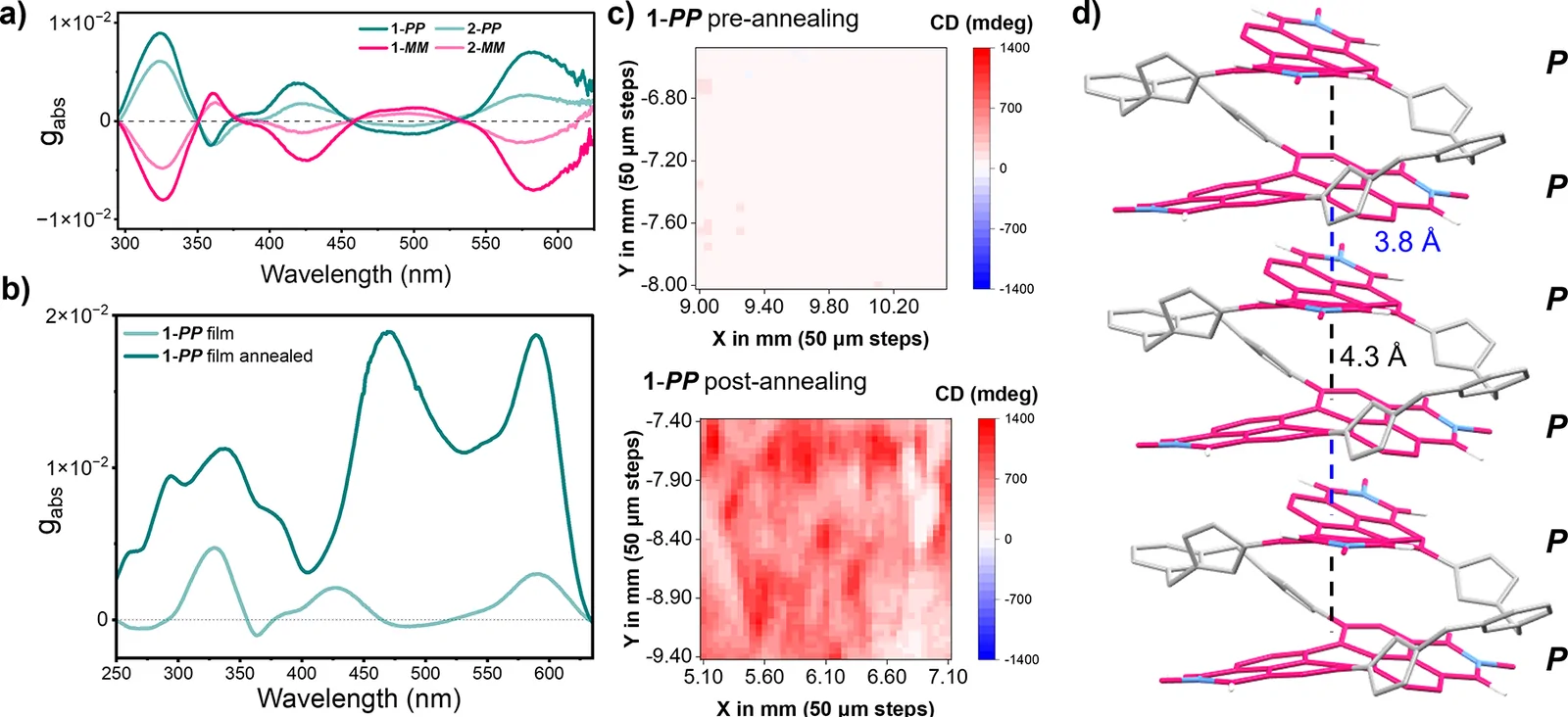
**a)** CD spectra of the enantiomers of bis-PDI macrocycles **1** and **2** in toluene (25 μM), **b)** MMP CD spectra of a thin film of **1-** ***PP*** before and after annealing, **c)** MMP CD maps (50 μm spatial resolution) of an area of the **1-** ***PP*** thin film before annealing (1.5 × 1.5 mm map at λ = 576 nm) and after annealing (2 × 2 mm map at λ = 590 nm), **d)** packing of **1-** ***PP*** from its X-ray crystal structure (enantiopure) showing the intermolecular (blue) and intramolecular (black) *d*_π–π_ between PDIs.

The *g*_abs_ for **1-** ***MM***/***PP*** is over three times larger than that of helical PDI dimers without supramolecular chirality.^34,36^ Moreover, **1-** ***MM***/***PP*** *g*_abs_ values are over two times larger than for a structurally related bis-PDI macrocycle, **3-** ***MM***/***PP*** (Table 1),^35^ which exhibits similar H-type coupling (*J*_*Tot*_ = +420 cm^–1^), yet importantly, unlike **1-** ***MM***/***PP***, its PDI helical and supramolecular chirality are opposing (Table 1).

**Table 1:** A Comparison of Experimental and Calculated Chiroptical Parameters of the ***PP*** Enantiomer of Bis-PDI Macrocycles **1** and, from Previous Work, **3** ^35^

**PDI dimer** (linker)	Helical–supramol. chirality	**Transition** (λ)^a^	***g***_**abs**_ (×10^–3^)^b^	***R*** (10^–40^ erg cm^3^)^c^	**|*μ*|** ^c^	**|*m*|** ^c^	**cos *θ*** ^c^
**1** ***-PP*** (*m*-aryl)	***PP***–***P***_***s***_	1 (583 nm)	7.2	731	1.63	1.31	0.22
		2 (500 nm)	–1.3	–751	3.33	0.68	–0.03
**3-** ***PP*** (*p*-aryl)	***PP***–***M***_***s***_	1 (610 nm)	–3.0	–223	0.45	0.01	–1.00
		2 (525 nm)	0.5	292	3.27	0.01	1.00

a The first (1) and second (2) lowest energy transitions, with λ taken from experimental CD spectra.

b Experimental value.

c Calculated value.

This result is consistent with our calculations of the two lowest energy transitions for the ***PP*** enantiomer of both macrocycle dimers **1** and **3** (Table 1), i.e., from their predicted rotatory strengths (*R*), which determine CD intensity. Macrocycle **1-** ***PP***, with matching ***P***_***s***_ supramolecular chirality, is predicted to have larger *R*, than **3-** ***PP*** with opposing ***M***_***s***_. The *R* for a transition between *i* and *j* states may be expressed as

R=Im(μij·mij)≈|μij||mij|cos⁡θ 1

where ***μ_ij_*** and ***m_ij_*** are the electric and magnetic transition dipole moment vectors respectively, and *θ* is the angle between them.^68^ Therefore, the calculations also show that the larger *R* predicted for chirally matched **1** (***PP***–***P***_***s***_) is due to ***m*** being up to two orders of magnitude larger than in chirally mismatched **3** (***PP***–***M***_***s***_), despite a less favorable relative orientation of ***μ*** and ***m*** (i.e., a smaller cos *θ*). We note that structural effects, such as conformational rigidity and ring strain arising from linker modification, may also contribute to the differences in CD measured between macrocycles **1** and **3**.

We also investigated the CD of the best-performing macrocycle enantiomers **1-** ***MM*** and **1-** ***PP*** in thin films prepared by dropcasting from *o*-dichlorobenzene. For this, we used Mueller–Matrix polarimetry (MMP) to accurately quantify and map CD across the films (SI, Section 7), since this technique deconvolutes the individual circular dichroism/birefringence (CD/CB) and linear dichroism/birefringence (LD/LB) contributions to the signal.^69,70^ Prior to thermal annealing (Figure 6b,c), the thin films of **1-** ***MM*** and **1-** ***PP*** exhibited homogeneous CD (*g*_abs_ = 3 × 10^–3^ at 589 nm) with equal and opposite CD spectra consistent with those in solution (Figure 6a). This indicates the macrocycles are discrete species within the isotropic film, and indeed the lack of chiroptical activity displayed by racemic films further supports the molecular origin of the CD.

Thermal annealing (100 °C) of **1-** ***MM***/***PP*** thin films leads to a six-fold increase in the *g*_abs_ (2 × 10^–2^ at 589 nm, Figures 6b and S7.16), with chiroptical homogeneity maintained across the CD maps (Figure 6c). Due to the emergence of film birefringence observed by polarized optical microscopy (Figure S7.17) and the increased crystallinity evidenced by powder XRD (Figure S4.6), the enhanced CD is attributed to increased ordering of bis-PDI macrocycles through π–π self-assembly. This interpretation is consistent with their strong tendency to form ordered π-stacked assemblies in the solid state (Figure 6d). Indeed, the positioning of the PDI’s triazole substituents to face into the macrocycle cavity ensures that the external π-surfaces of the dimer are unhindered for π-stacking interactions (*d*_π–π_ = 3.8 Å, Figure 6d). Extended π-stacking is also supported by a change in the CD spectrum, with the notable absence of Cotton effects leading to broadband CD between 250 and 600 nm (Figure 6b), likely due to nondegenerate (off-resonance) intra- and inter-molecular excitonic coupling.^71^ Importantly, MMP revealed that the enantiomers **1-** ***MM*** and **1-** ***PP*** again gave equal and opposite CD spectra (Figure S7.16), demonstrating that CD amplification is not due to LD/LB contamination in the anisotropic films.^5^

## Circularly Polarized Luminescence

The matched helical–supramolecular chirality in bis-PDI macrocycle enantiomers **1-** ***PP***–***P***_***s***_ and **1-** ***MM***–***M***_***s***_ also has important consequences for their CPL. Intriguingly, the mirror image CPL spectra (*g*_lum_ = 5 × 10^–3^) contain two monosignate bands at λ = 600 and 650 nm (Figure 7), which, to the best of our knowledge, is unprecedented for organic small molecule emitters. Instead, previous chiral materials with two CPL bands report bisignated spectra.^72–74^

**Figure 7:**
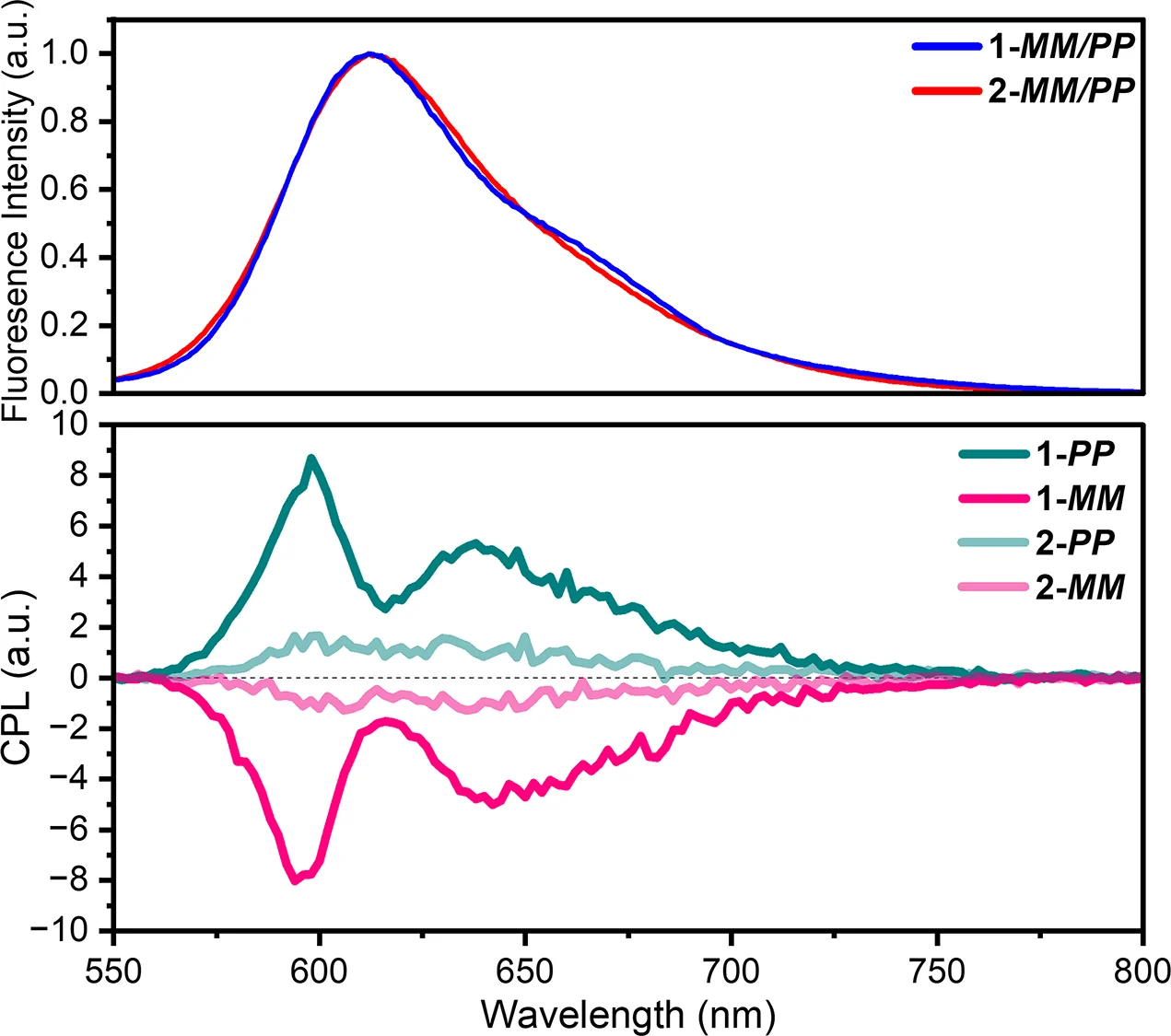
Fluorescence emission (top) and CPL (bottom) spectra for bis-PDI macrocycle enantiomers **1-** ***MM***/***PP*** and **2-** ***MM***/***PP*** in toluene (40 μM, λ_ex_ = 450 nm, spectra shown are an average of ten scans). The arrow indicates the EX band in **1-** ***MM***/***PP***.

The dual, monosignate CPL of **1** is attributed to dual LE and EX emission, as the CPL bands directly mirror the two features in the fluorescence spectrum (Figure 7): the 600 nm peak assigned to LE emission and the red-shifted 650 nm shoulder assigned to EX emission. This assignment is also consistent with EXs often generating a single, red-shifted CPL band.^75^ Importantly, in **1-** ***MM***/***PP***, the chirality of the LE and EX states is defined by the PDI helicity (***MM***/***PP***) and supramolecular chirality (***M***_***s***_/***P***_**s**_) respectively, with their matched chirality therefore explaining why both CPL bands are the same sign (−/+).

Therefore, owing to its strong, dual-banded, monosignate CPL, macrocycle **1** exhibits a broadband CPL spectrum (*g*_lum_ ≥ 10^–3^ from 560–760 nm; Figure S8.1) that is unique among chiral emitters displaying distinct LE and EX CPL bands (Table S8.1). In previous systems, the LE CPL band is substantially weaker than the EX band,^76,77^ by approximately an order of magnitude, and the two CPL bands frequently possess opposite signs, giving rise to bisignate spectra.^72,73,78,79^ Monosignate, broadband CPL is valuable because it removes the risk of signal cancelation and thus the need for narrow bandpass filters. Optical filtering is often required in applications with chiral lanthanide complexes containing, for instance, Eu­(III), due to their narrow, multisignate CPL.^80^ Macrocycle **1-** ***MM***/***PP*** also possesses a large Φ (0.9), resulting in high CPL brightness at 600 nm (*B*_CPL_ = 104 M^–1^ cm^–1^), which is excellent for a macrocycle^81^ and among chiral emitters in general.^17^

In the absence of EX population, macrocycle **2-** ***MM***/***PP*** exhibits a single CPL band (Figure 7), and thus an overall narrower CPL spectrum (Δλ ≈ 50 nm where *g*_lum_ ≥ 10^–3^), consistent with the LE character of its excited state. Compared to **1-** ***MM***/***PP***, the smaller *g*_lum_ (1 × 10^–3^) of **2-** ***MM***/***PP*** is in line with its weaker CD, and thus results in a lower *B*_CPL_ = 18 M^–1^ cm^–1^, further evidencing the importance of intramolecular hydrogen bonding in rigidifying chiral structures.

## Summary and Conclusions

We report the first PDI dimer with matching helical (e.g., ***PP***) and supramolecular (***P***_***s***_) chirality, achieved via the rational design of macrocycles with *m*-aryl linkers. The configurational stability of these macrocycles enables all dimer stereoisomers (***PP***, ***MM*** and ******MP******) to be isolated and characterized, including by X-ray crystallography.

Excitonic coupling is shown to depend on PDI helicity, being nearly four times larger in dimers with matched (i.e., ***PP*** and ***MM***) vs mismatched (***MP***) chirality, due to the cofacial vs slipped geometry of the π-stack. Furthermore, intramolecular hydrogen bonding, engineered via *m*-pyridyl linker choice, supplements the π–π interactions, leading to enhanced configurational stability and excitonic coupling in macrocycle **1**.

The combination of homochirality and rigidity results in excimer population in **1-*MM***/***PP*** which, supported by transient absorption studies, is shown to be in equilibrium with the local excited state, leading to a distinct CPL spectrum with two bands. Critically, due to the matched helical and supramolecular chirality in **1-*MM***/***PP***, both CPL bands have the same sign, which explains the strong broadband spectrum (Δλ = 200 nm with *g*_lum_ > 10^–3^) and exceptional *B*_CPL_ (104 M^–1^ cm^–1^) for a small organic chiral emitter. Alongside linker-induced structural effects, matched chirality (e.g., ***PP*** **–** ***P***_***s***_) is identified as an important contributing factor to the amplification of g_abs_, which manifests in thin films with strong, uniform, and broadband CD (*g*_abs_ = 10^–2^, Δλ = 350 nm).

Overall, this work demonstrates the importance of matching chromophore helicity, and its subsequent alignment with supramolecular chirality, for tuning the excitonic coupling and (chir)­optical properties of a π-stack. These fundamental new insights will inform the rational design of efficacious chiral materials that contain multiple types of chirality.

## Supplementary Material





## Notes

The DFT-optimized structures (ZIP)
Details of experimental procedures, including synthetic methods, characterization, crystallographic, spectroscopic data, methods, DFT calculations, and additional Schemes, Figures, and Tables (Sections 1–13) (PDF)
