# Supplementary material for: Matching Helical and Supramolecular Chirality in a Perylene Diimide Dimer: Impact on (Chir)Optical Properties
Source: J Am Chem Soc. 2026 Sep 2;148(36):38964–75. doi: 10.1021/jacs.6c11478 (PMC13587683; doi:10.1021/jacs.6c11478)
Supplement: Supplementary file 1 [file ja6c11478_si_002.pdf]

Supporting Information

Matching Helical and Supramolecular Chirality in a Perylene Diimide Dimer - Impact on (Chir)optical Properties

Mya Kotecha,[a] Denis Hartmann,[a] Artemijs Krimovs,[b] Oussama Errida,[a] Tiberiu-M. Gianga,[c] Georgia R. F. Orton,[a] Giuliano Siligardi,[c] Robert Pal,[b] Giovanni Bressan[d,*] and Timothy A. Barendt[a,*]

[a]University of Birmingham, School of Chemistry, Birmingham, B15 2TT, UK
* Email: t.a.barendt@bham.ac.uk
[b]University of Durham, Dept. of Chemistry, Durham, DH1 3LE, UK
[c]Diamond Light Source, Harwell Sci. & Innovation Campus, Didcot, OX11 0DE, UK
[d]University of East Anglia, School of Chemistry, Norwich, NR4 7TJ, UK
* Email: g.bressan@uea.ac.uk

Contents

1. Synthesis and characterisation 2
2. Further ^{1}H NMR spectroscopy 9
3. Further photophysics 20
4. X-ray diffraction 24
5. Chiral HPLC 30
6. Circular dichroism 33
7. Mueller–matrix polarimetry 41
8. Circularly polarised luminescence 51
9. Time-resolved fluorescence and femtosecond transient absorption spectroscopy 54
10. Computational chemistry 58
11. Excitonic coupling calculations 70
12. NMR spectra 71
13. MALDI spectra 84
14. References 88

1. Synthesis and characterisation

Reagents were purchased from commercial sources (Merck, Acros Organics, Fluorochem and Alfa Aesar) and used without further purification. $Cu(MeCN)_4 \cdot PF_6$ was stored in a desiccator. tris((1-benzyl-4- triazolyl)methyl)amine (TBTA) was prepared following a literature procedure.[1] Solvents were used as supplied (analytical/HPLC-grade from Fisher or Sigma-Aldrich). If required, solvents were dried over molecular sieves (3 Å) overnight before use and stored under N_2 atmosphere. In some cases, anhydrous solvents were degassed with N_2 and dried using an Innovative Technology PureSolv MD 5 solvent purification system. Water was distilled and microfiltered using an ELGA DV 35 Purelab water purification system. Eluent mixtures are reported in volume:volume or %vol. sho

Note: Organic azides (e.g., **7** and **8**) are *potentially unstable and explosive*. Therefore, the following precautions must be taken when working with these compounds:

i) Do not use halogenated solvents (e.g. DCM or $CHCl_3$) in the presence of NaN_3
ii) Avoid acids in the presence of NaN_3
iii) Do not handle NaN_3 with metal spatulas
iv) Avoid working on large scales (ideally < 1 g)
v) Avoid using sealed flasks
vi) Avoid concentrating organic azides in large quantities through rotary evaporation and use large flasks and a blast shield when performing such procedures
vii) Never heat NaN_3 or organic azides

Column chromatography was carried out using Merck Silica Gel 60 Å, 230-400 mesh, 40-63 µm particle size. TLC was carried out on Merck silica gel 60 F254 Al plates. Preparative TLC was performed using 20 × 20 cm plates with 1 cm silica thickness.

NMR spectroscopy measurements were recorded using a Bruker AVIII400 or AV Neo 400 instrument and peaks were referenced to the residual solvent peak. Coupling constants (J) are reported in Hertz (Hz). Standard abbreviations indicating multiplicity were used as follows: m = multiplet, quint = quintet, q = quartet, t = triplet, d = doublet, s = singlet, app. = apparent, br = broad, sept = septet. Signal assignment was carried out using 2D NMR methods (COSY, NOESY, HSQC or HMBC) where necessary. In some cases, complex multiplets with multiple contributing proton signals, exact assignment was not possible.

MALDI-TOF MS spectra were recorded on a 2022 UltrafleXtreme MALDI-TOF/TOF spectrometer using trans-2-[3-(4- tert-butylphenyl)-2-methyl-2-propenylidene]-malononitrile as the matrix. MALDI MS data was acquired in negative reflectron mode with the matrix trans-2-[3-(4-tert-Butylphenyl)-2-methyl-2-propenylidene]malononitrile (DCTB) (Insight Biotechnology, Welwyn Garden City, UK), on a Bruker ultrafleXtreme MALDI-TOF/TOF mass spectrometry (Bruker Daltonics, Bremen, Germany). The instrument was externally calibrated using the Bruker Peptide Calibration Standard (Bruker Daltonics), in negative ion mode. Mass spectra were acquired in the range m/z 800 - 3000. FlexControl software v3.4 (Bruker Daltonics) was used to control the data acquisition, and data processed in the FlexAnalysis software v3.4 (Bruker Daltonics).

The synthesis of macrocycles **1** and **2** was carried out according to Scheme **S1.1**. Compounds **9**[2] and **4**[3] were prepared following literature procedures. Compound **9** was initially isolated as a mixture of 1,6 and 1,7 PDI regioisomers which were separated via HPLC using a literature procedure.[4] The 1,7 regioisomer was exclusively used to synthesise compound **4**. Preparation of compounds **7**[5] and **8**[5] were prepared following literature procedures.

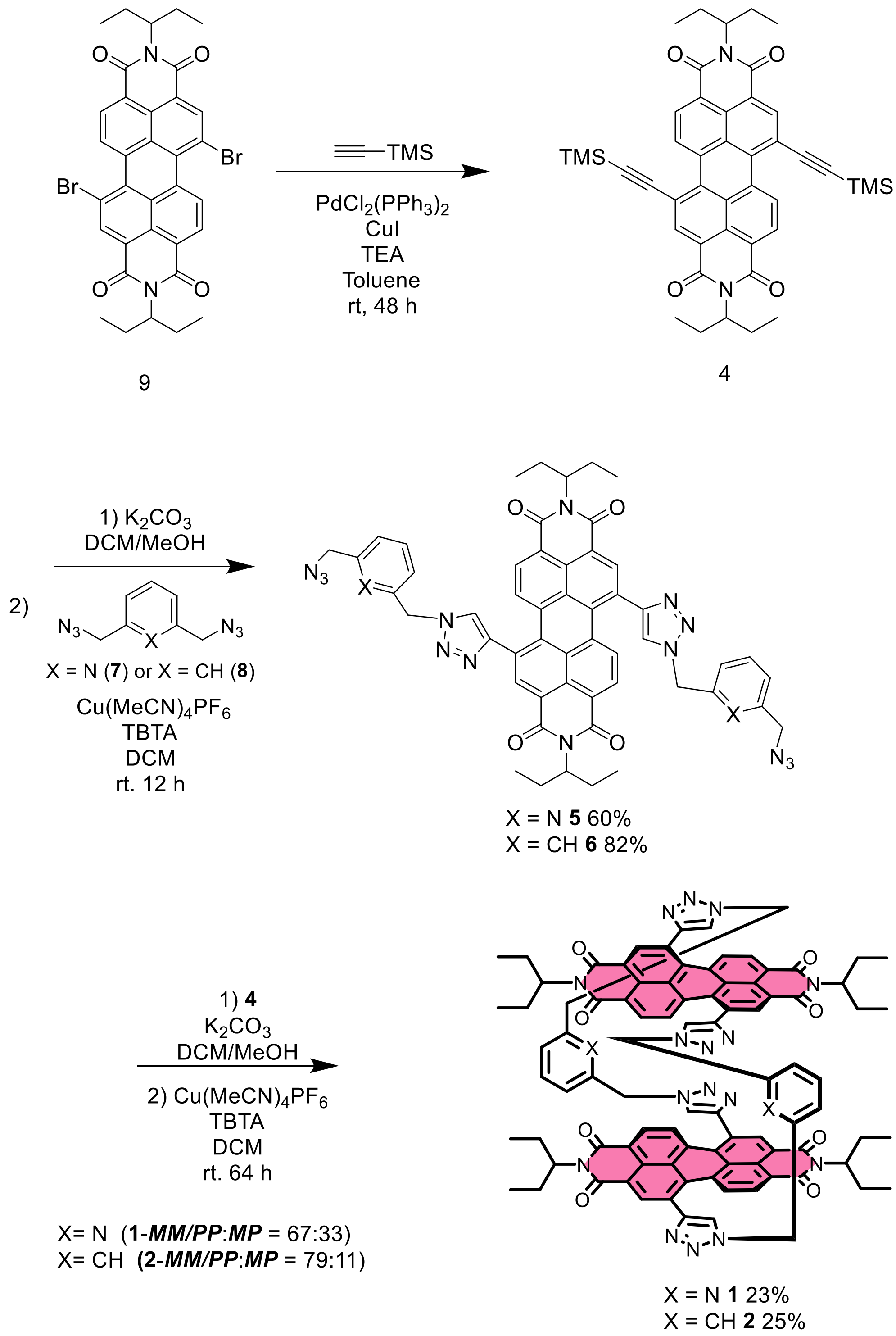

Scheme S1.1: Multistep synthesis of bis-PDI macrocycles **1** and **2**.

TMS-protected bis-alkyne PDI 4

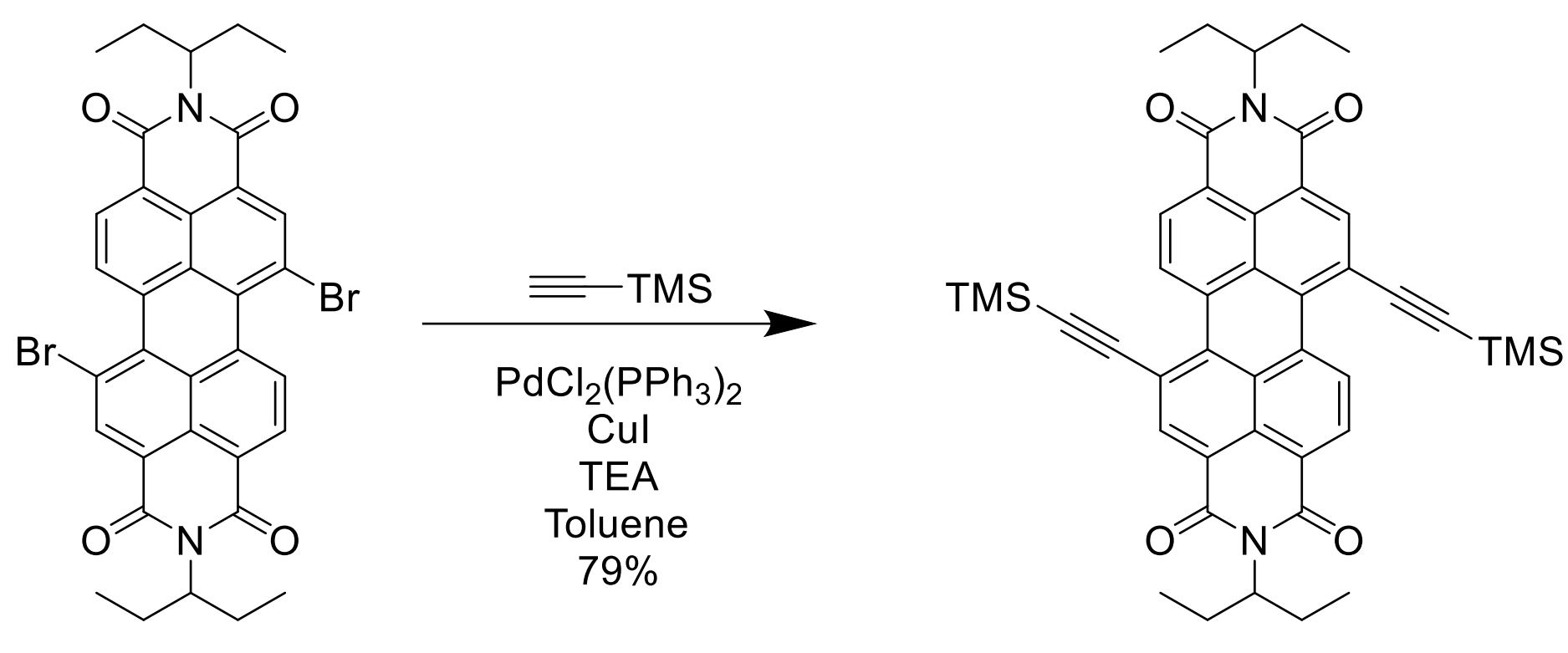

9 4

To a solution of 1,7 di-bromo PDI **9**[2] (729 mg, 1.06 mmol) in 1:1 TEA/toluene (100 mL) was added $PdCl_2(PPh_3)_2$ (45 mg, 64 μmol, 0.06 equiv.), CuI (22 mg, 117 μmol, 0.11 equiv.) and trimethylsilylacetylene (520 mg, 750 μL, 5.3 mmol, 5 equiv.) The mixture was thoroughly degassed with nitrogen and stirred at room temperature for 48 h. The solvent mixture was then removed in vacuo and the resulting residue was purified via silica gel flash chromatography (1:1 *n*-hexane:DCM). The title compound was afforded as a dark red solid (601 mg, 831 μmol, 79%). The ^{1}H NMR spectroscopic data is in agreement with the literature.[3]

^{1}H NMR (400 MHz, $CDCl_3$) δ/ppm 10.21 (d, *J* = 8.2 Hz, 2H), 8.82 (s, 2H), 8.64 (d, *J* = 8.3 Hz, 2H), 5.12 – 5.01 (m, 2H), 2.35 – 2.20 (m, 4H), 2.00 – 1.87 (m, 4H), 0.93 (t, *J* = 7.4 Hz, 13H), 0.39 (s, 18H).

2,6-bis(azidomethyl)pyridine 7

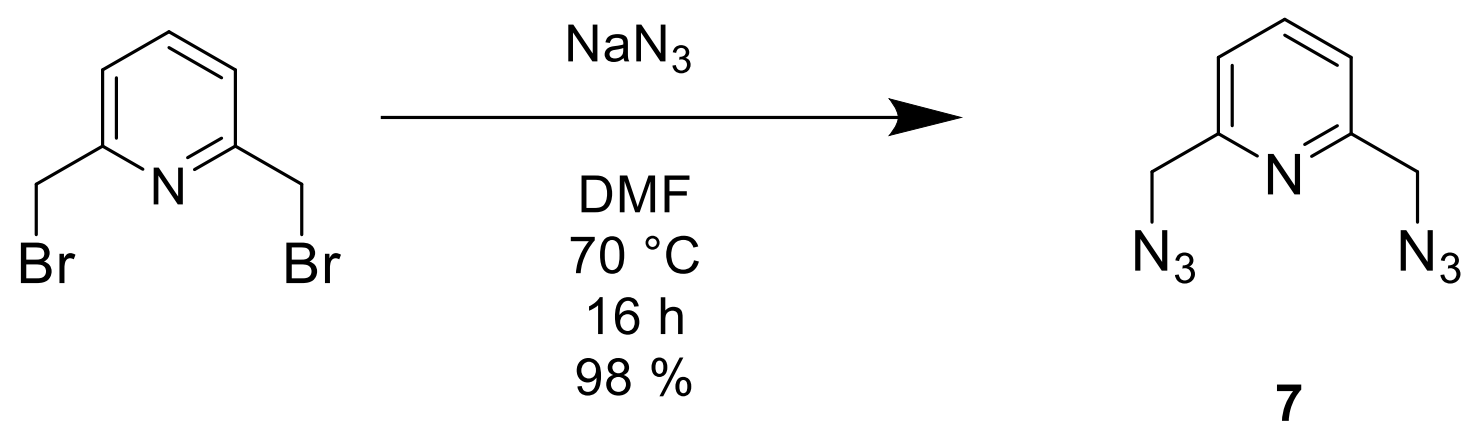

7

Warning: Organic azides are potentially unstable and explosive. Therefore, please follow the precautions listed on pg. S2 when working with these compounds.

To a solution of 2,6-bis-(bromomethyl)pyridine (1 g, 3.8 mmol) in DMF (25 mL) was added NaN_3 (0.7 g, 11 mmol, 3 equiv.). The mixture was stirred at 70 °C for 16 h. The reaction was then concentrated, and the compound was extracted using water (3 x 20 mL) and EtOAc (3 x 20 mL). The organic phases were then combined and washed with brine (2 x 30 mL), dried over $MgSO_4$, filtered and finally concentrated *through rotary evaporation at room temperature and with the use of a blast shield*, to yield a pale-yellow oil (0.7 g, 98%) as compound **7**. The compound was *stored in the freezer* until required. The ^{1}H NMR spectroscopic data is in agreement with the literature.[5]

^{1}H NMR (400 MHz, $CDCl_3$) δ/ppm 7.74 (t, *J* = 7.8 Hz, 1H), 7.29 (d, *J* = 7.8 Hz, 2H), 4.47 (s, 4H)

2,6-bis(azidomethyl)xylyl 8

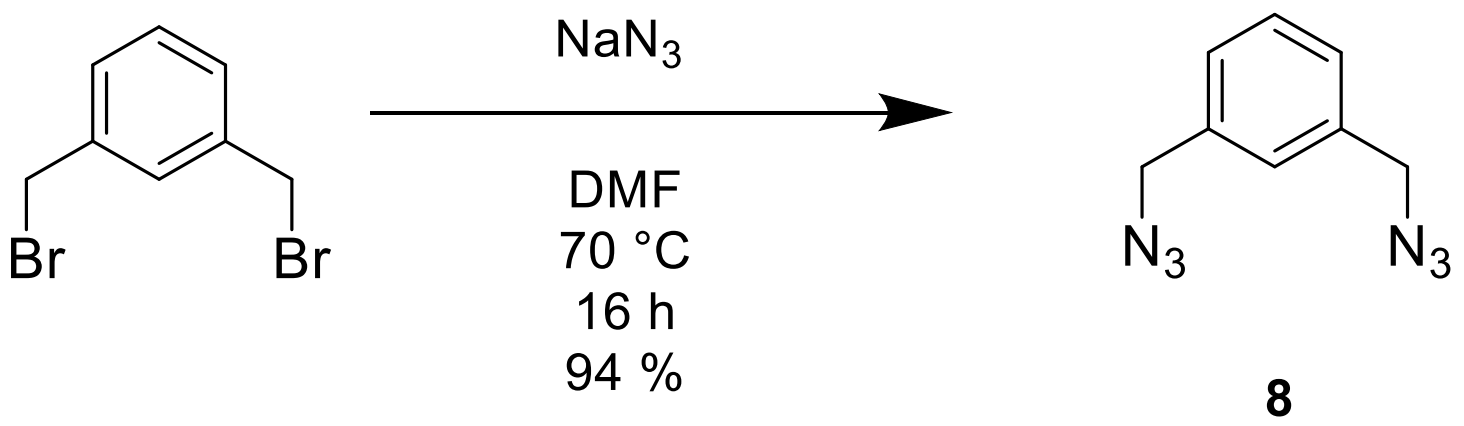

8

Warning: Organic azides are potentially unstable and explosive. Therefore, please follow the precautions listed on pg. S2 when working with these compounds.

To a solution of 1,3-bis(bromomethyl)benzene (1 g, 3.6 mmol) in DMF (20 mL) was added NaN_3 (0.7 g, 11 mmol, 3 equiv.). The mixture was stirred at 70 °C for 16 h. The reaction was then concentrated, and the compound was extracted using water (3 x 20 mL) and EtOAc (3 x 20 mL). The organic phases were then combined and washed with brine (2 x 30 mL), dried over $MgSO_4$, filtered and finally concentrated *through rotary evaporation at room temperature and with the use of a blast shield* to yield a pale-yellow oil (0.7 g, 94%) as compound **8**. The compound was *stored in the freezer* until required. The ^{1}H NMR spectroscopic data is in agreement with the literature.[5]

^{1}H NMR (400 MHz, $CDCl_3$) δ/ppm 7.85 – 7.76 (br, s, 1H), 7.30 – 7.12 (br, m, 3H), 4.16 – 4.14 (br, s, 4H).

Acyclic pyridyl bis-triazole PDI 5

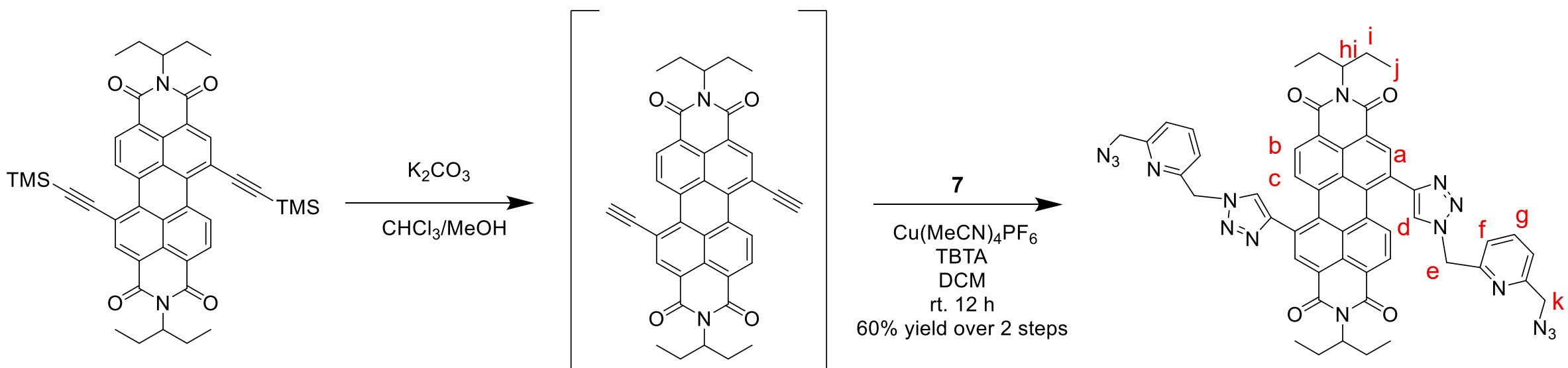

To a solution of TMS-protected PDI **4** (190 mg, 263 μmol) in $CHCl_3$ (200 mL) was added dropwise a saturated solution of K_2CO_3 in MeOH. The mixture was stirred at rt for 40 mins, and complete TMS-deprotection was confirmed by TLC. 10 mL of H_2O was added to the mixture. The mixture was then washed with brine (30 ml), and the organic phases were dried over anhydrous $MgSO_4$ and concentrated in vacuo to afford the crude bis-alkyne PDI, which was immediately used for the next step, due to its tendency to aggregate over time. This bis-alkyne PDI was dissolved in DCM (50 mL) and compound **7** (497 mg, 2.6 mmol, 10 equiv.) was added with tris((1-benzyl-4- triazolyl)methyl)amine (28 mg, 53 μmol, 0.2 equiv.). The solution was then degassed with nitrogen. The copper (I) catalyst $Cu(MeCN)_4 \cdot PF_6$ (20 mg, 34 μmol, 0.2 equiv.) was then added and the solution was once again degassed with nitrogen. The reaction was stirred at rt for 12 h. The solvent was then removed in vacuo. The resulting residue was purified by silica gel flash column chromatography (0.5:99.5 MeOH:DCM to 1.5:98.5 MeOH:DCM) affording the title compound as a purple solid (152 mg, 158 μmol, 60%).

^{1}H NMR (400 MHz, $CDCl_3$) δ/ppm 8.69 (s, $2H_a$), 8.07 (d, *J* = 8.1 Hz, $2H_b$), 7.95 (s, $2H_d$), 7.89 – 7.80 (m, $4H_{g,c}$), 7.36 (dd, *J* = 7.7, 3.9 Hz, $4H_f$), 5.76 (s, $4H_e$), 5.09 – 4.97 (m, $2H_h$), 4.42 (s, $4H_k$), 2.32 – 2.16 (m, $4H_i$), 1.97 – 1.82 (m, $4H_i$), 0.90 (t, *J* = 7.4 Hz, $12H_j$)

^{13}C NMR (101 MHz, $CDCl_3$) δ/ppm 156.46, 154.01, 148.22, 138.64, 134.50, 133.47, 129.10, 128.99, 128.42, 122.68, 122.17, 121.90, 57.64, 55.80, 54.99, 24.99, 11.26.

MS (MALDI-TOF) found m/z = 956.37 [$M^{\cdot-}$], $C_{52}H_{44}N_{16}O_4$ requires 956.62

Acyclic xylyl bis-triazole PDI 6

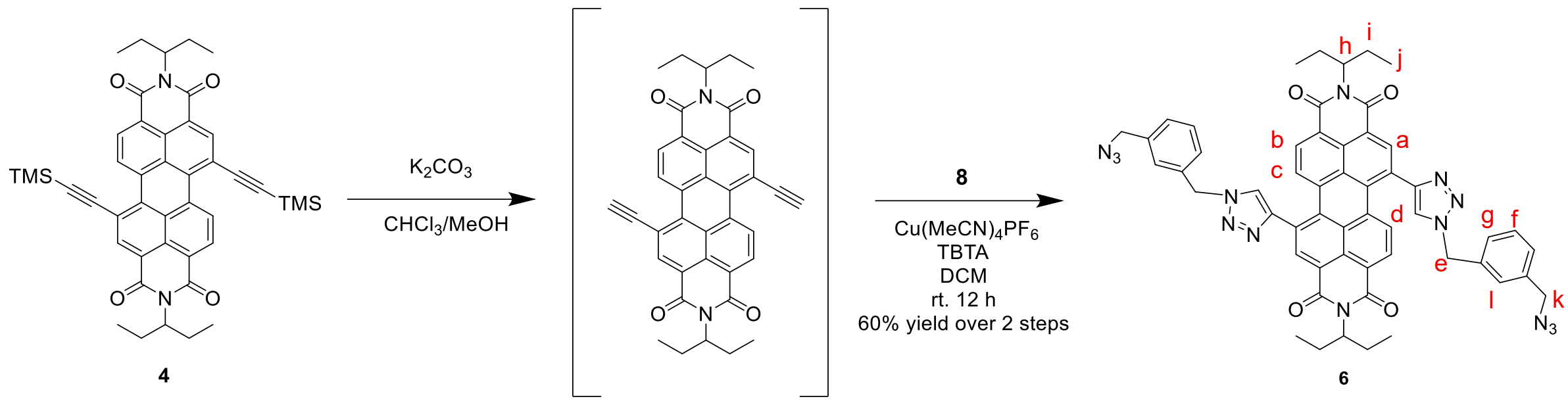

To a solution of TMS-protected PDI **4**[3] (275 mg, 380 µmol) in $CHCl_3$ (200 mL) was added dropwise a saturated solution of K_2CO_3 in MeOH. The mixture was stirred at rt for 40 mins, and complete TMS-deprotection was confirmed by TLC. 10 mL of H_2O was added to the mixture. The mixture was then washed with brine (30 ml), and the organic phases were dried over anhydrous $MgSO_4$ and concentrated in vacuo to afford the crude bis-alkyne PDI, which was immediately used for the next step, due to its tendency to aggregate over time. This bis-alkyne PDI was dissolved in DCM (50 mL) and to this was added compound **8** (716 mg, 3.8 mmol, 10 equiv.) and tris((1-benzyl-4- triazolyl)methyl)amine (40 mg, 53 µmol, 0.2 equiv.). The solution was then degassed with nitrogen. The copper (I) catalyst $Cu(MeCN)_4 \cdot PF_6$ (28 mg, 34 µmol, 0.2 equiv.) was then added and the solution was once again degassed with nitrogen. The reaction was stirred at rt for 12 h. The solvent was then removed in vacuo. The resulting residue was purified by silica gel flash column chromatography (0.5:99.5 MeOH:DCM to 1.5:98.5 MeOH:DCM) affording the title compound as a purple solid (296 mg, 310 µmol, 82%).

^{1}H NMR (400 MHz, $CDCl_3$) δ/ppm 8.66 (s, $2H_a$), 8.12 (d, *J* = 8.1 Hz, $2H_b$), 7.84 (d, *J* = 8.1 Hz, $2H_c$), 7.63 (s, $2H_d$), 7.50 – 7.45 (m, $2H_f$), 7.38 – 7.31 (m, $6H_{f,g,l}$), 5.66 (s, $4H_e$), 5.08 – 4.99 (m, $2H_h$), 4.41 (s, $4H_k$), 2.30 – 2.20 (m, 4Hi), 1.97 – 1.87 (m, $4H_i$), 0.91 (t, *J* = 7.4 Hz, $12H_j$).

^{13}C NMR (101 MHz, $CDCl_3$) δ/ppm 148.41, 136.90, 135.12, 134.40, 133.42, 130.01, 129.11, 128.96, 128.92, 128.85, 128.43, 127.93, 127.61, 121.80, 57.66, 54.42, 54.32, 24.99, 11.25.

MS (MALDI-TOF) found m/z = 954.38 [$M^{\cdot-}$], $C_{54}H_{46}N_{14}O_4$ requires 954.64

Bis-PDI *m*-pyridyl macrocycle 1

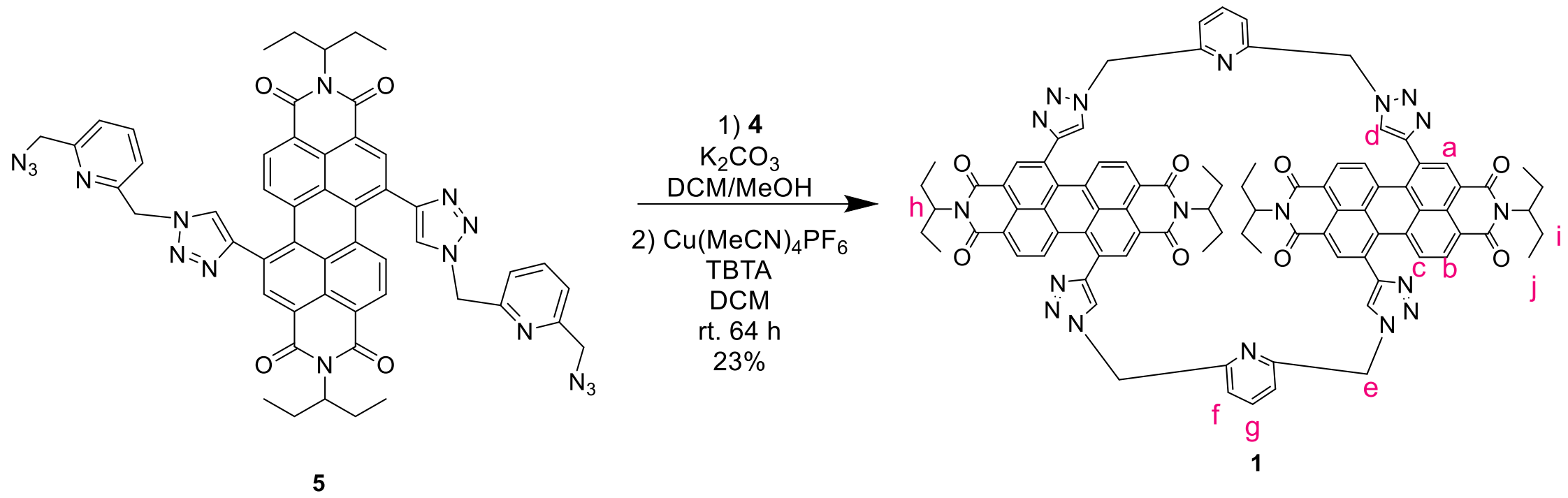

To a solution of TMS-protected PDI **4**[3] (115 mg, 159 µmol) in $CHCl_3$ (50 mL) was added dropwise a saturated solution of K_2CO_3 in MeOH. The reaction was monitored by TLC (99:1 $CHCl_3$:MeOH) and upon complete TMS-deprotection the reaction mixture was washed with water (3 x 50 mL), dried with $MgSO_4$ and concentrated in vacuo to yield the crude bis-alkyne PDI, which was immediately used for the next step due to its tendence to aggregate over time. This bis-alkyne PDI (159 µmol, 1 equiv.) was dissolved in DCM (250 mL) and compound **5** (152 mg, 159 µmol, 1 equiv.) was added with tris((1-benzyl-4- triazolyl)methyl)amine (34 mg, 64 µmol, 0.4 equiv.). The solution was then degassed with nitrogen. The copper catalyst $Cu(MeCN)_4{\cdot}PF_6$ (24 mg, 64 µmol, 0.4 equiv.) was then added and the solution was once again degassed with nitrogen. The reaction was stirred at rt for 64 h and monitored by TLC (0.5:99.5 MeOH:DCM). The solvent was then removed in vacuo. The resulting residue was purified by silica gel flash column chromatography (0.5:99.5 MeOH:DCM to 1.5:98.5 MeOH:DCM), affording the title compound as a purple solid (56 mg, 36 µmol, 23%) The macrocycle diastereoisomers **1-*MM/PP*** and **1-*MP*** were isolated via preparative silica TLC. This was done by an initial run of 100% hexane to help product loading, followed by a series of runs from 0.5:99.5 MeOH:DCM to 1.5:98.5 MeOH:DCM.

1-*MM/PP* **^{1}H NMR** (TCE-*d*$_2$, 373 K, 400 MHz) δ/ppm 8.37 (s, J = 1.3 Hz, 4H$_a$), 8.03 (t, J = 7.7 Hz, 2H$_g$), 7.94 (d, J = 1.5 Hz, 4H$_b$), 7.67 (d, J = 7.7 Hz, 4H$_f$), 7.63 (d, J = 8.1 Hz, 4H$_c$), 6.03 (s, 4H$_d$), 5.96 (d, J = 15.7 Hz, 4H$_e$), 5.54 (d, J = 15.7 Hz, 4H$_e$), 5.01 – 4.92 (m, 4H$_h$), 2.25 – 2.07 (m, 8H$_i$), 2.05 – 1.94 (m, 8H$_i$), 0.93 – 0.84 (m, 24H$_j$).

1-*MP* **^{1}H NMR** (TCE-*d*$_2$, 373 K, 400 MHz) δ/ppm 8.22 (s, 4H$_a$), 8.13 (s, 4H$_d$), 8.02 (t, J = 7.7 Hz, 2H$_g$), 7.87 (d, J = 8.1 Hz, 4H$_f$), 7.74 (dd, J = 7.9, 5.5 Hz, 8H$_{b,c}$), 5.95 (s, 4H$_e$), 5.69 (d, J = 14.6 Hz, 4H$_e$), 4.83 – 4.76 (m, 4H$_h$), 1.95 – 1.82 (m, 8H$_i$), 1.76 – 1.57 (m, 8H$_i$) 1.54 – 1.50 (m, 24H$_{j,}$).

1-*MM/PP* **^{13}C NMR** (TCE-*d*$_2$, 373 K, 101 MHz) δ/ppm 163.27, 162.22, 153.74, 146.05, 138.69, 134.91, 133.55, 131.54, 130.38, 129.23, 128.75, 128.32, 128.14, 127.79, 123.14, 122.63, 122.55, 120.58, 74.06, 73.78, 73.51, 58.05, 54.45, 24.95, 24.59, 10.95.

For **1**-***MP***, a ^{13}C NMR spectrum could not be recorded due to stereoisomer interconversion (to **1**-***MM***/***PP***) under the conditions required for data collection (long collection times and elevated temperatures).

MS (MALDI-TOF) found m/z = 1534.59 [$M^{\cdot-}$], $C_{90}H_{74}N_{18}O_4$ requires 1535.64

Bis-PDI *m*-xylyl macrocycle 2

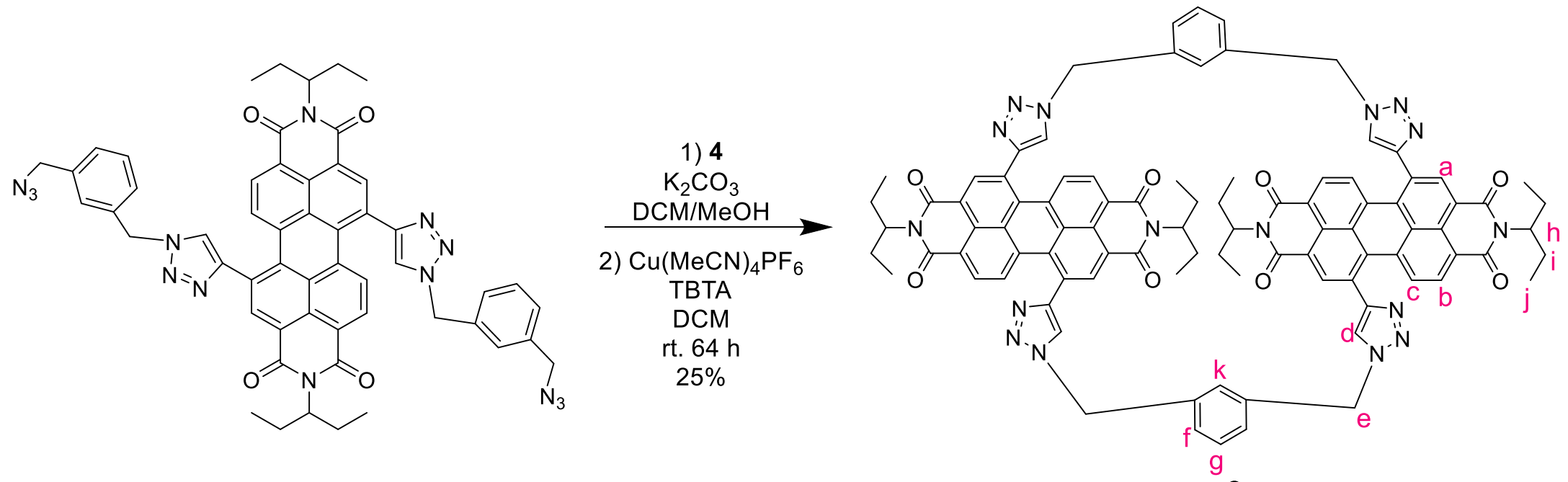

To a solution of TMS-protected PDI **4**[3] (185 mg, 256 μmol) in $CHCl_3$ (75 mL) was added dropwise a saturated solution of K_2CO_3 in MeOH. The reaction was monitored by TLC (99:1 $CHCl_3$:MeOH) and upon complete TMS-deprotection the reaction mixture was washed with water (3 x 75 mL), dried with $MgSO_4$ and concentrated in vacuo to yield the crude bis-alkyne PDI, which was immediately used for the next step due to its tendency to aggregate over time. This bis-alkyne PDI (256 μmol, 1 equiv.) was dissolved in DCM (250 mL) and compound **6** (245 mg, 256 μmol) was added with tris((1-benzyl-4- triazolyl)methyl)amine (55 mg, 102 μmol, 0.4 equiv.). The solution was then degassed with nitrogen. The copper catalyst $Cu(MeCN)_4{\cdot}PF_6$ (38 mg, 102 μmol, 0.4 equiv.) was then added and the solution was once again degassed with nitrogen. The reaction was stirred at rt for 64 h and monitored by TLC (0.5:99.5 MeOH:DCM). The solvent was then removed in vacuo. The resulting residue was purified by silica gel flash column chromatography (0.5:99.5 MeOH:DCM to 1.5:98.5 MeOH:DCM), affording the title compound as a purple solid (102 mg, 66 μmol, 25%) The macrocycle diastereoisomers **2-*MM/PP*** and **2-*MP*** were isolated via preparative silica TLC. This was done by an initial run of 100% hexane to help product loading, followed by a series of runs from 0.5:99.5 MeOH:DCM to 1.5:98.5 MeOH:DCM.

2-*MM/PP* **^{1}H NMR** (TCE-*d*$_2$, 373 K, 400 MHz) δ/ppm 8.63 (s, 4H_a), 8.20 (d, *J* = 8.1 Hz, 4H_c), 7.93 (d, *J* = 8.1 Hz, 4H_b), 7.43 (t, *J* = 7.8 Hz, 2H_g), 7.34 (s, 4H_d), 7.16 (dd, *J* = 7.9, 1.7 Hz, 4H_f), 6.91 (d, *J* = 1.9 Hz, 2H_k), 5.68 (d, *J* = 15.5 Hz, 4H_e), 5.55 (d, *J* = 15.5 Hz, 4H_e), 4.98 – 4.89 (m, 4H_h), 2.19 – 2.10 (m, 8H_i), 1.91 – 1.84 (m, 8H_i), 0.81 (s, 24Hj).

2-*MP* **^{1}H NMR** (TCE-*d*$_2$, 323 K, 400 MHz) δ/ppm 8.55 (s, 4H_a), 8.19 (d, *J* = 8.0 Hz, 4H_c), 7.85 (d, *J* = 8.1 Hz, 4H_b), 7.44 (d, *J* = 7.5 Hz, 2H_g), 7.34 (s, 4H_d), 7.32 – 7.25 (d, 6$H_{f,k}$), 5.63 (d, *J* = 15.0 Hz, 4H_e), 5.53 (d, *J* = 15.0 Hz, 4H_e), 4.94 (t, *J* = 5.7 Hz, 4H_h), 2.27 – 2.09 (m, 8H_i), 1.85 (q, *J* = 6.7 Hz, 8H_i), 0.81 (s, 24H_j).

2-*MM/PP* **^{13}C NMR** (TCE-*d*$_2$, 373 K, 101 MHz) δ/ppm 147.93, 135.65, 134.08, 133.22, 130.33, 129.18, 128.79, 128.65, 128.20, 128.06, 125.93, 121.67, 120.44, 57.80, 53.99, 25.02, 11.51, 11.48.

For **2-*MP***, a ^{13}C NMR spectrum could not be recorded due to stereoisomer interconversion (to **2-*MM*/*PP***) under the conditions required for data collection (long collection times and elevated temperatures).

MS (MALDI-TOF) found m/z = 1532.60 [$M^{\cdot-}$], $C_{90}H_{74}N_{18}O_4$ requires 1533.15

2. Further ^{1}H NMR spectroscopy

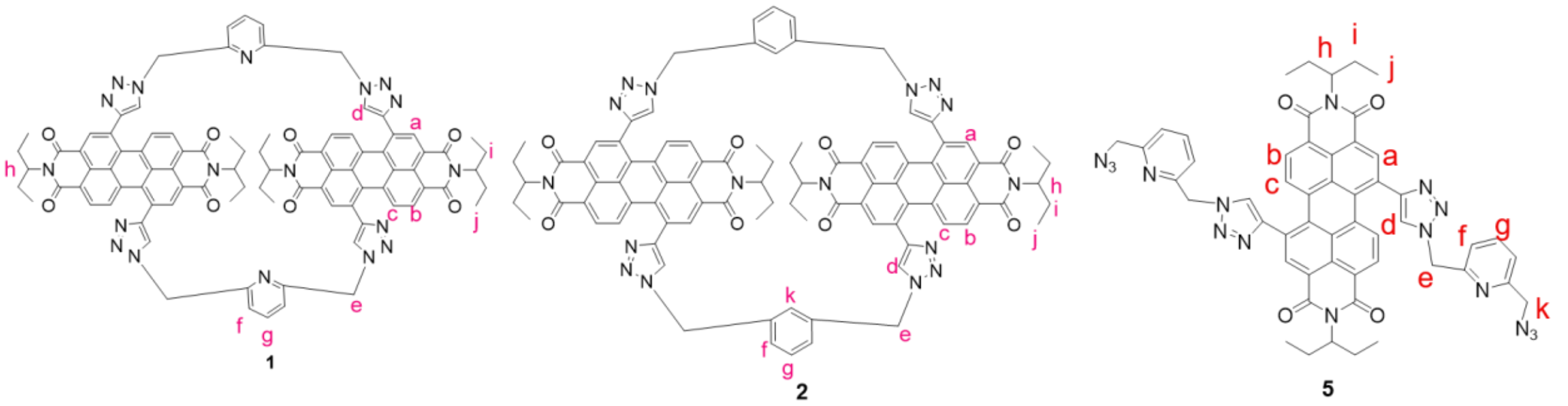

Figure S2.1: Proton labels used for bis-PDI macrocycles **1** and **2** and PDI monomer **5** throughout the manuscript and the SI.

a) 1D ^{1}H NMR spectroscopy

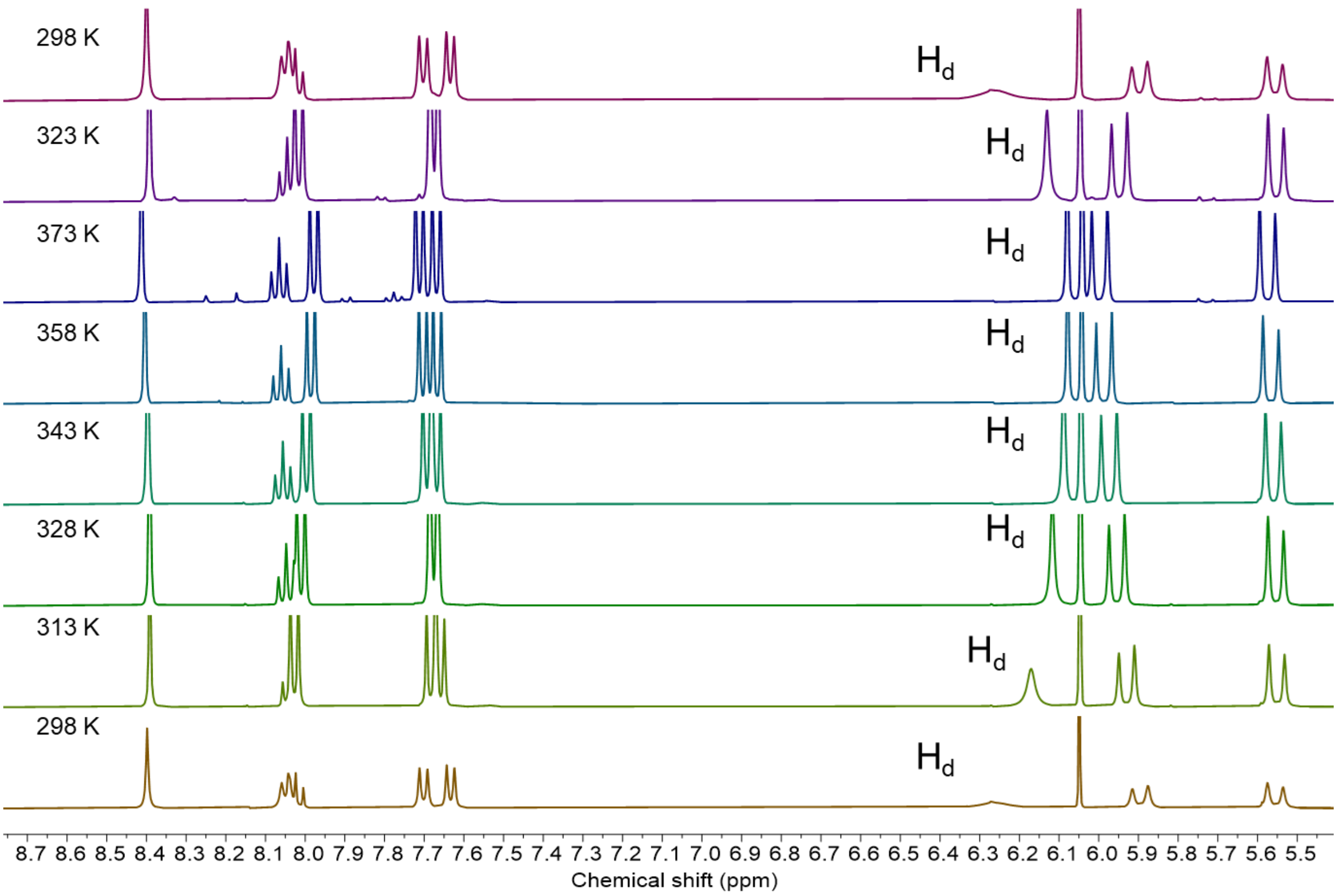

Figure S2.2: Truncated ^{1}H NMR spectra of **1-*MM/PP*** (TCE-d_2, 400 MHz) at various temperatures ranging from 298 K to 373 K. The spectra showing how the triazole (H_d) proton is broad at room temperature and sharpens upon heating.

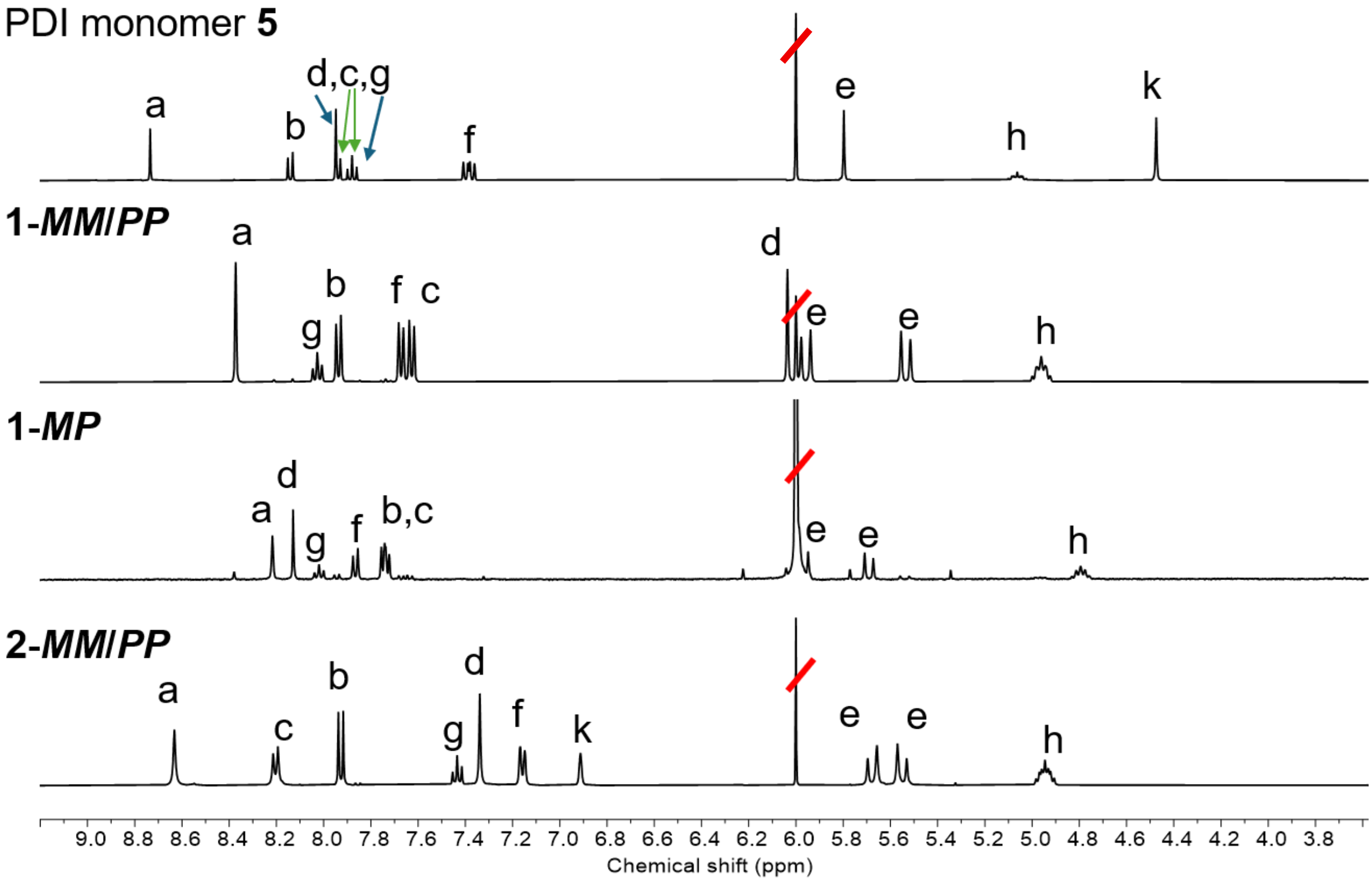

Figure S2.3: Truncated ^{1}H NMR spectra of PDI monomer **5** and bis-PDI macrocycles **1-*MM/PP***, **1-*MP*** and **2-*MM/PP*** (TCE-d_2, 400 MHz) at 373 K.

The distinct ^{1}H NMR spectra of **1-*MM/PP*** and **1-*MP*** support different dimer geometries (Fig S2.3). Notably, the triazole proton of **1-*MM/PP*** is substantially upfield shifted relative to **1-*MP*** ($\Delta\delta$ = 1.3 ppm for H_d), consistent with shielding by the neighbouring PDI π-surface. This interpretation agrees with both the crystal structure of **1-*MM/PP*** (Fig. 3) and previous literature reports of co-facial π-stacked systems.[6] In contrast, the triazole protons of **1-*MP*** are located further from the PDI π-surface (Fig. 3) and therefore resonate at chemical shifts comparable to those of monomer **5** ($\Delta\delta$ = 0.2 ppm). The ^{1}H NMR spectra also show that imide group proton H_h is shifted further upfield in **1-*MP*** compared to **1-*MM/PP***. This would be expected from their respective crystal structures (Fig. 3), since the imide groups are more susceptible to PDI ring-currents in **1-*MP*** due to the slipped stack arrangement, whilst in **1-*MM/PP*** the imide groups are directed away from these π-surfaces.

The ^{1}H NMR spectra above (Fig. S2.3) provide further support for the calculated structure of **2-*MM/PP*** (SI, section 10). As for **1-*MM/PP***, the PDI resonances $H_{a,b}$ are shifted upfield relative to those of monomer **5**, consistent with π–π stacking interactions between the two PDI units.[6] The imide group proton H_h is similarly shifted in both **1-*MM/PP*** and **2-*MM/PP*** as well. Finally, although both macrocycles exhibit evidence of π-stacking, the PDI resonances ($H_{a\text{-}c}$) are less strongly shifted in **2-*MM/PP*** than in **1-*MM/PP*** (Fig. S2.3), suggesting a greater average distance between π-surfaces. This supports our assessment that **2-*MM/PP*** is more conformationally flexible than **1-*MM/PP***.

b) ^{1}H–^{1}H ROESY NMR spectroscopy

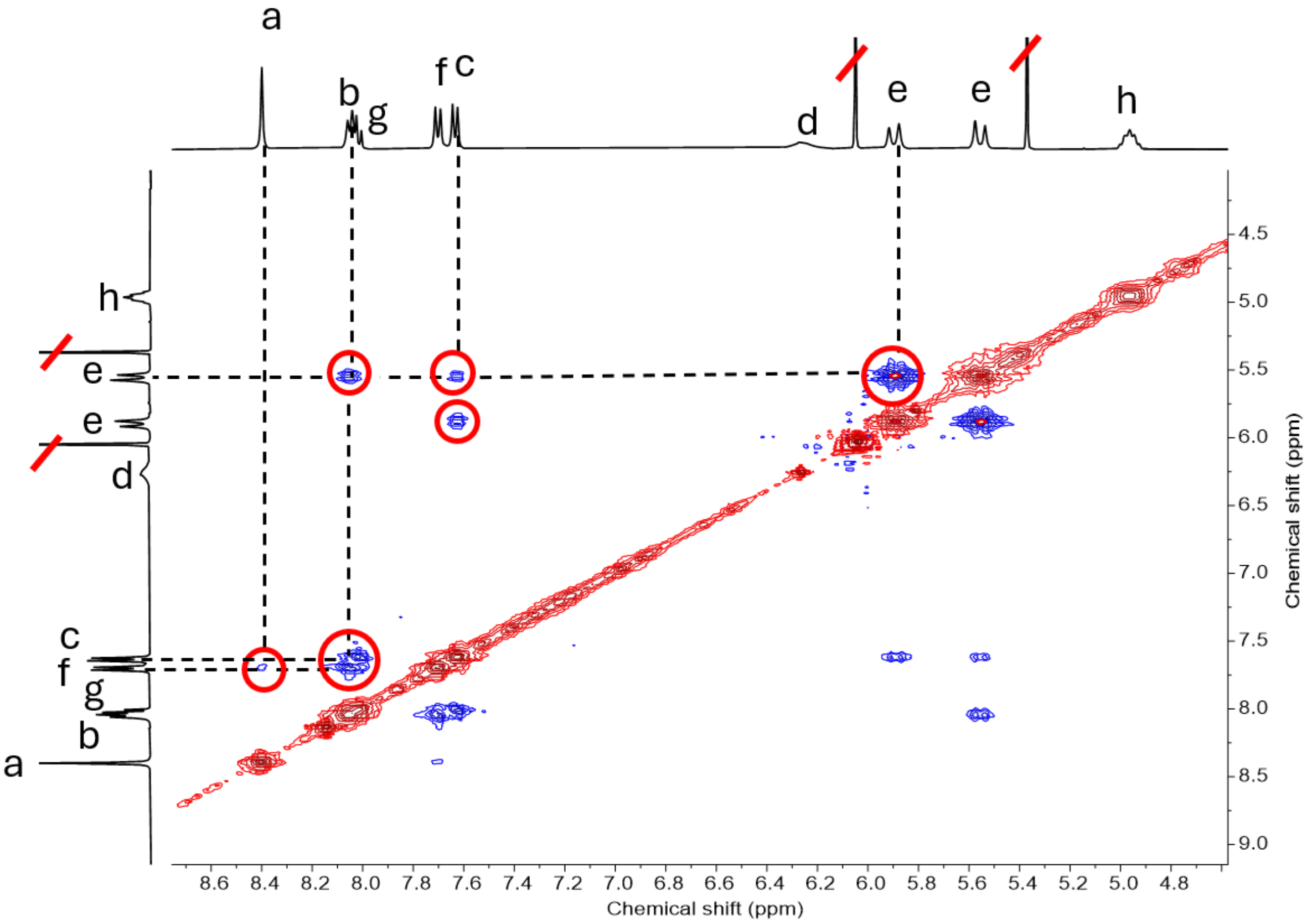

Figure S2.4: ^{1}H–1H ROESY NMR spectrum of bis-PDI macrocycle **1-*MM*/*PP*** (TCE-d_2, 400 MHz, 298 K) with key through-space proton correlations labelled.

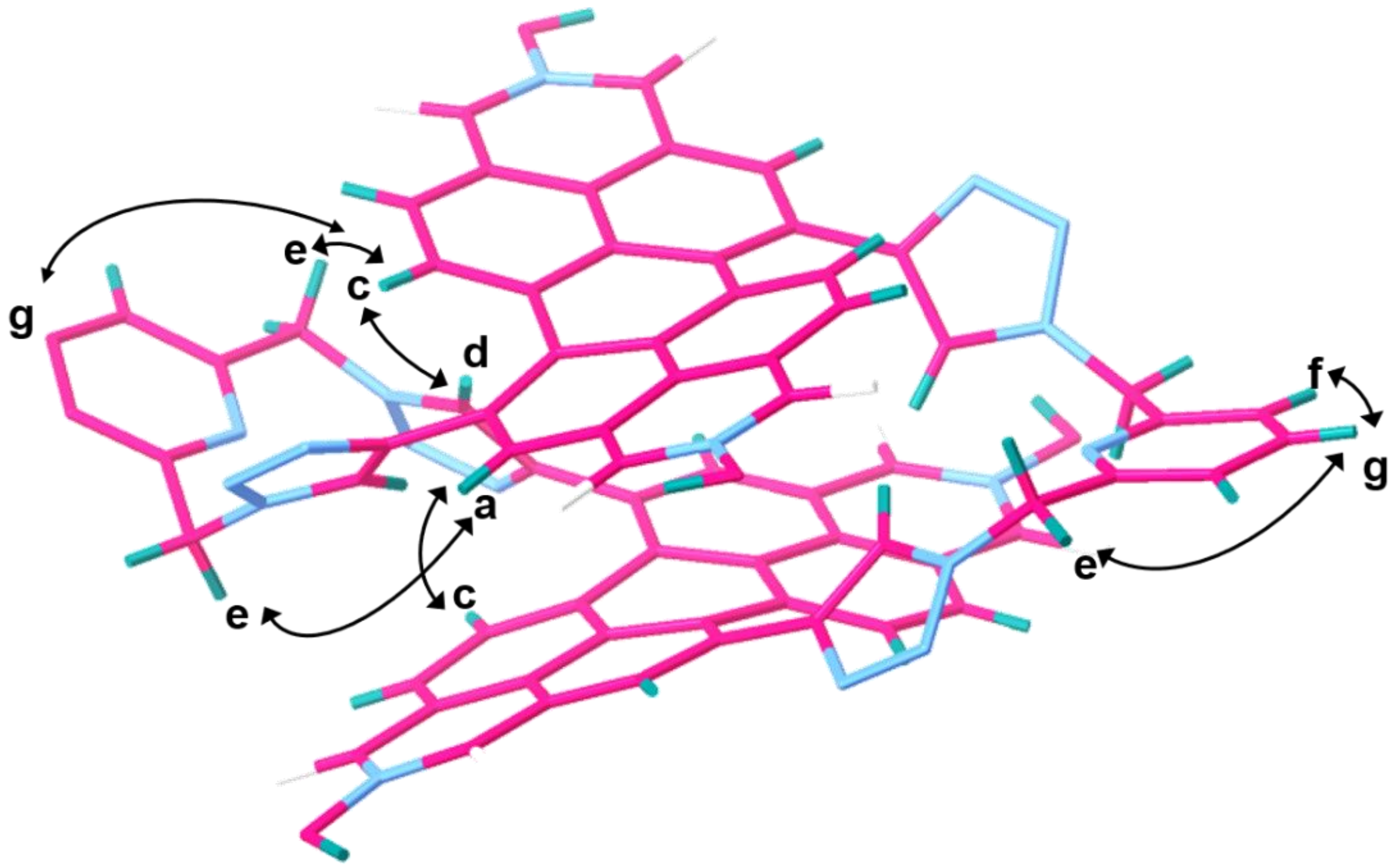

Figure S2.5: The through-space proton correlations from ROESY NMR are labelled on the X-ray crystal structure of bis-PDI macrocycle **1-*MM*/*PP***.

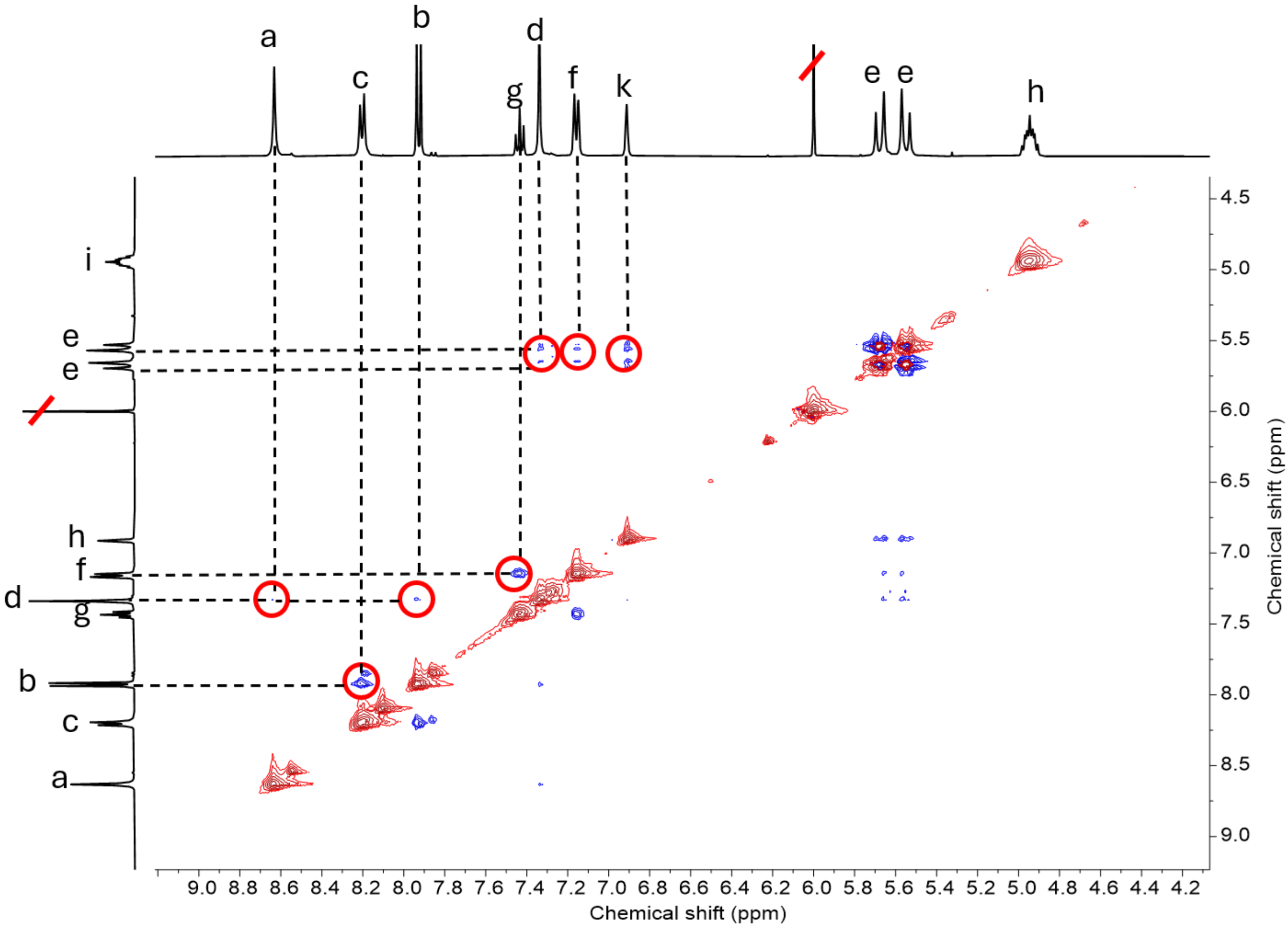

Figure S2.6: ^{1}H–1H ROESY NMR spectrum of bis-PDI macrocycle **2**-***MM***/***PP*** (TCE-d_2, 400 MHz, 298 K) with key through-space proton correlations labelled.

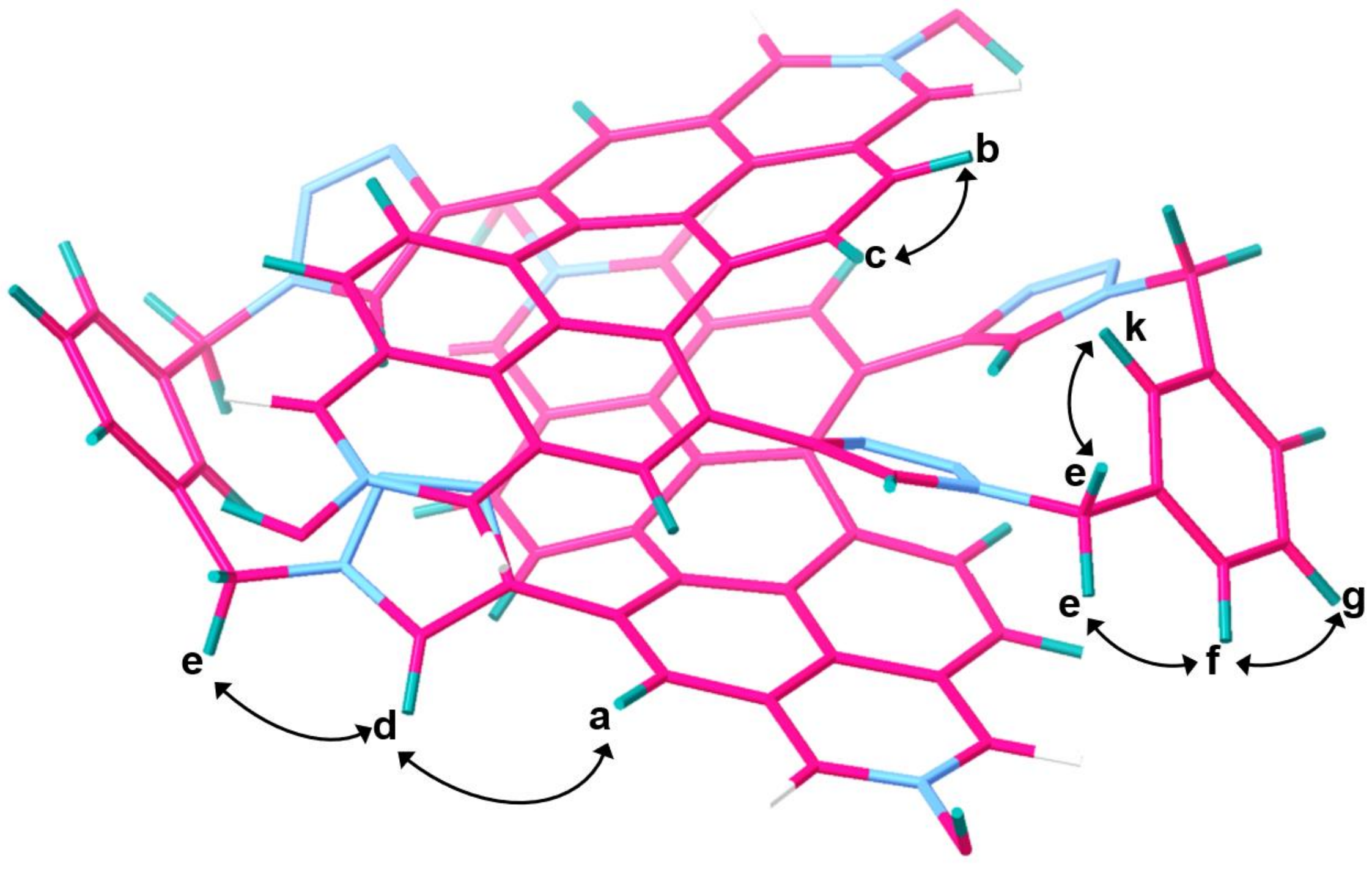

Figure S2.7: The through-space proton correlations from ROESY NMR are labelled on the calculated structure (SI, section 10) of bis-PDI macrocycle **2**-***MM***/***PP***.

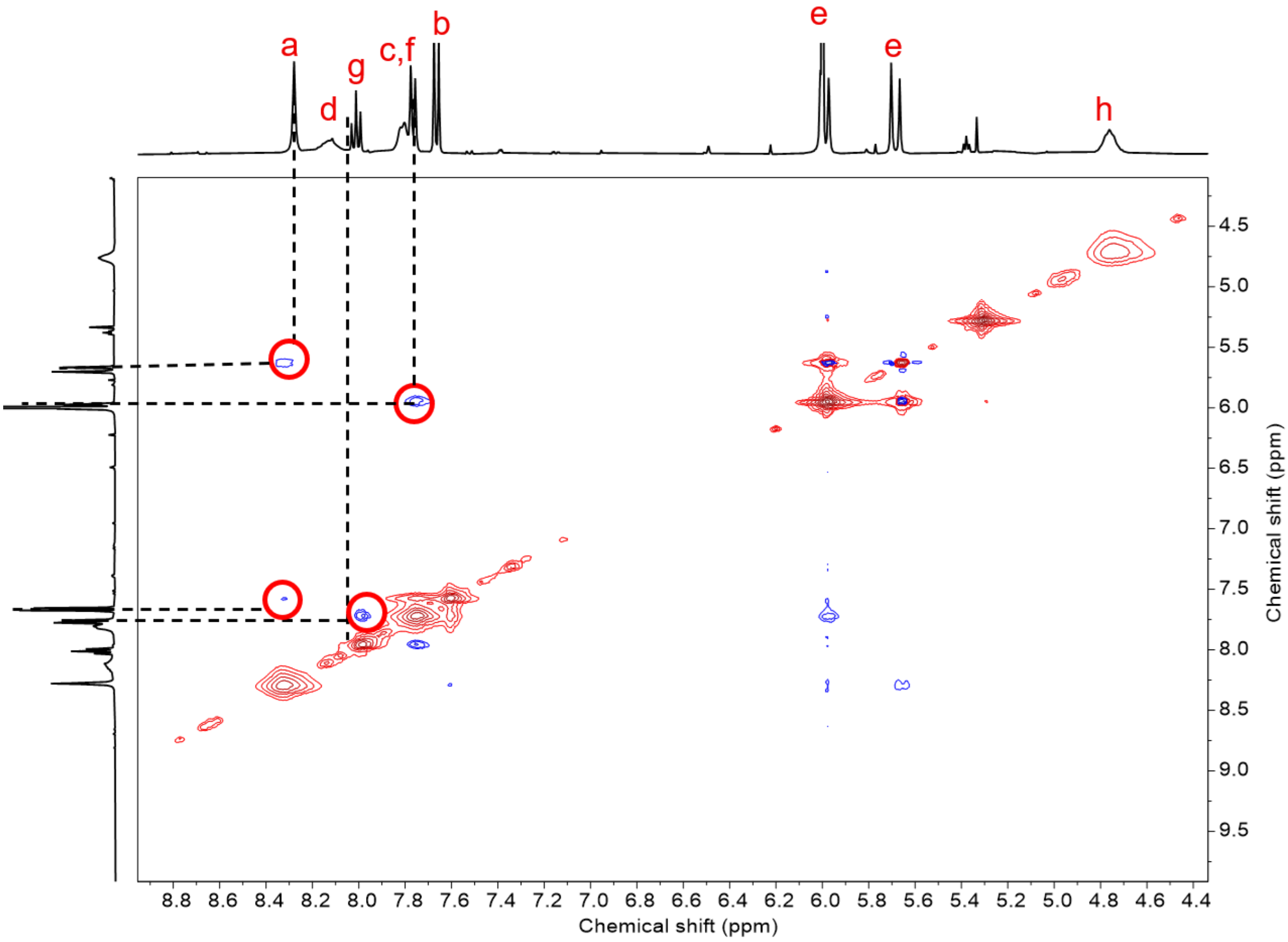

Figure S2.8: ^{1}H–1H ROESY NMR spectrum of bis-PDI macrocycle **1-*MP*** (TCE-d_2, 400 MHz, 298 K) with key through-space proton correlations labelled.

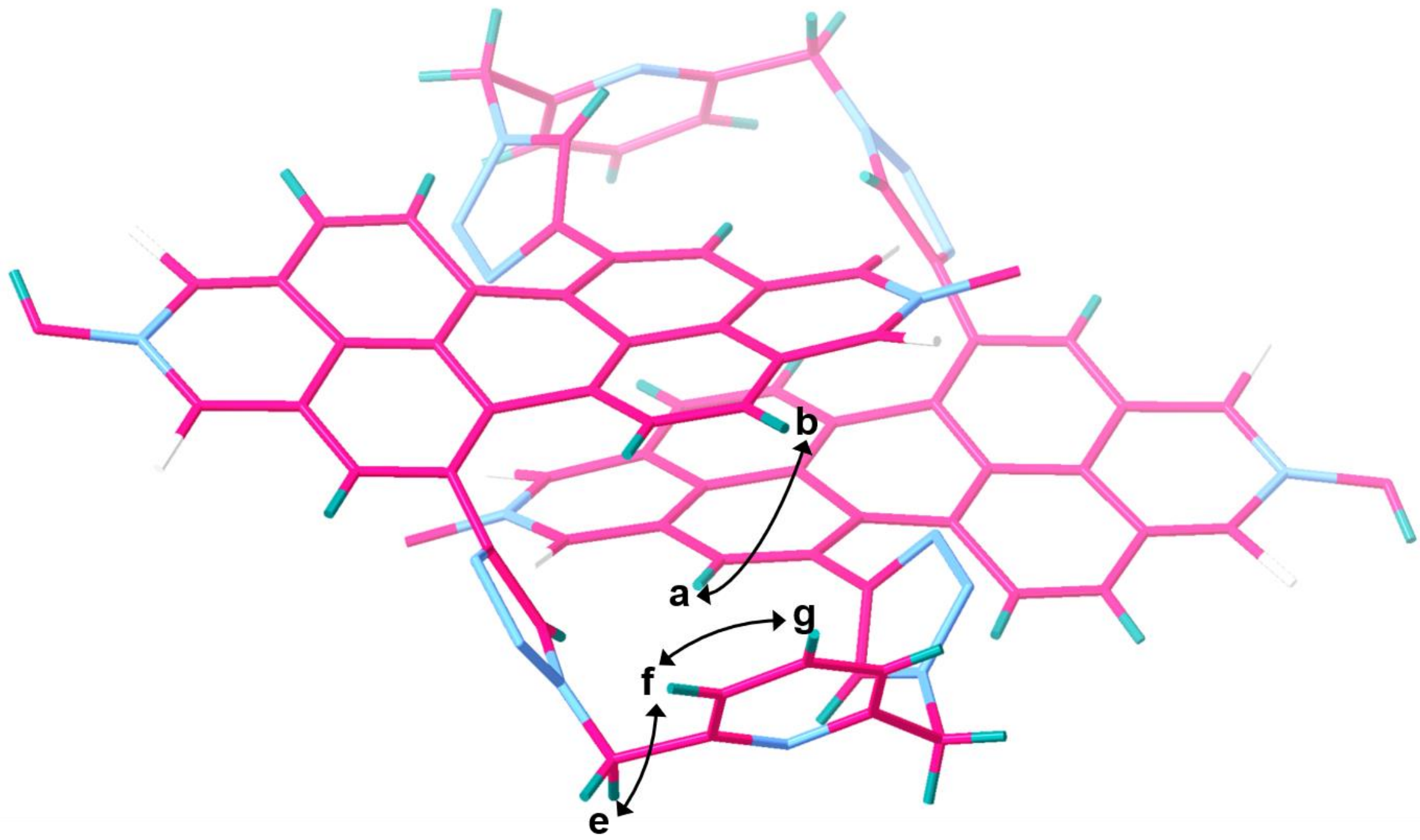

Figure S2.9: The through-space proton correlations from ROESY NMR are labelled on the crystal structure of bis-PDI macrocycle **1-*MP***.

Table S2.1: A summary of through-space proton correlations from the ROESY NMR spectra of bis-PDI macrocycles.

Through-space correlation ($H_x \leftrightarrow H_y$)		Dimer 1-*MP*	Dimer 1-*MM*/*PP*	Dimer 2-*MM*/*PP*
**PDI–PDI**	a ↔ b	✓	-	-
	a ↔ c	-	✓	-
	b ↔ c	-	-	✓
**PDI–linker**	a ↔ d	-	-	✓
	a ↔ e	✓	✓	-
	c ↔ d	-	✓	-
	c ↔ e	-	✓	-
	c ↔ g	-	✓	-
**linker–linker**	d ↔ e	-	-	✓
	e ↔ f	✓	-	✓
	e ↔ g	-	✓	-

The PDI–PDI correlations that are not possible on the same PDI unit are $H_a \leftrightarrow H_{b/c}$. Therefore, the existence of these correlations provides strong evidence for proximal PDI units in the macrocycle dimers. For macrocycle **1-*MM*/*PP***, an $H_a \leftrightarrow H_c$ cross-peak is observed which supports the compact π-stack from the crystal structure (Fig. 3), whereby rotational displacement of the PDI units positions these two protons closer in space. Instead, for macrocycle **1-*MP***, an $H_a \leftrightarrow H_b$ cross-peak is seen. This is in line with the slipped π-stack in the crystal structure (Fig. 3), since the long axis displacement of the PDI units places these two protons in closer proximity.

For PDI-linker correlations, an $H_a \leftrightarrow H_d$ correlation likely occurs due to rotation of the triazole unit around the PDI–triazole C–C bond. This is the only PDI-linker correlation seen for **2-*MM*/*PP***, and is consistent with the predicted structure (SI, section 10), where H_d protons are positioned away from the macrocycle cavity and in closer proximity to PDI protons H_a. However, the $H_a \leftrightarrow H_d$ correlation is not seen for **1-*MM*/*PP***, instead replaced by a $H_c \leftrightarrow H_d$ correlation, consistent with a distinct orientation of the triazole groups in which the triazole proton is directed towards the macrocycle cavity for intramolecular hydrogen bonding, as seen in the crystal structure (Fig. 3). Indeed, this cross peak was characteristic of the co-facial π-stacked structure of our previous bis-PDI macrocycle.[7] The existence of many PDI-linker correlations ($H_a \leftrightarrow H_e$, $H_c \leftrightarrow H_e$, $H_c \leftrightarrow H_g$) that are unique to **1-*MM*/*PP***, indicate a well-defined macrocycle conformation that is consistent with the crystal structure (Fig. 3).

While the $H_d \leftrightarrow H_e$ linker-linker correlation is seen for the PDI monomer and dimer **2-*MM*/*PP***, its absence for **1-*MM*/*PP*** provides further evidence for the orientation of the triazole protons into the macrocycle cavity for hydrogen bonding. Instead, **1-*MM*/*PP*** exhibits a unique $H_e \leftrightarrow H_g$ correlation, indicating a distinct geometry of the aryl group in the linker. This is consistent with a rotation of the pyridyl ring into a position that facilitates intramolecular hydrogen bonding, providing further support for the conformation seen in the crystal structure (Fig. 3). Macrocycle **2-*MM*/*PP*** exhibits an $H_e \leftrightarrow H_f$ correlation as expected from an orientation of the phenyl ring that is more orthogonal relative to the plane of the PDI dimer in line with its calculated structure (SI, section 10).

c) DOSY NMR spectroscopy

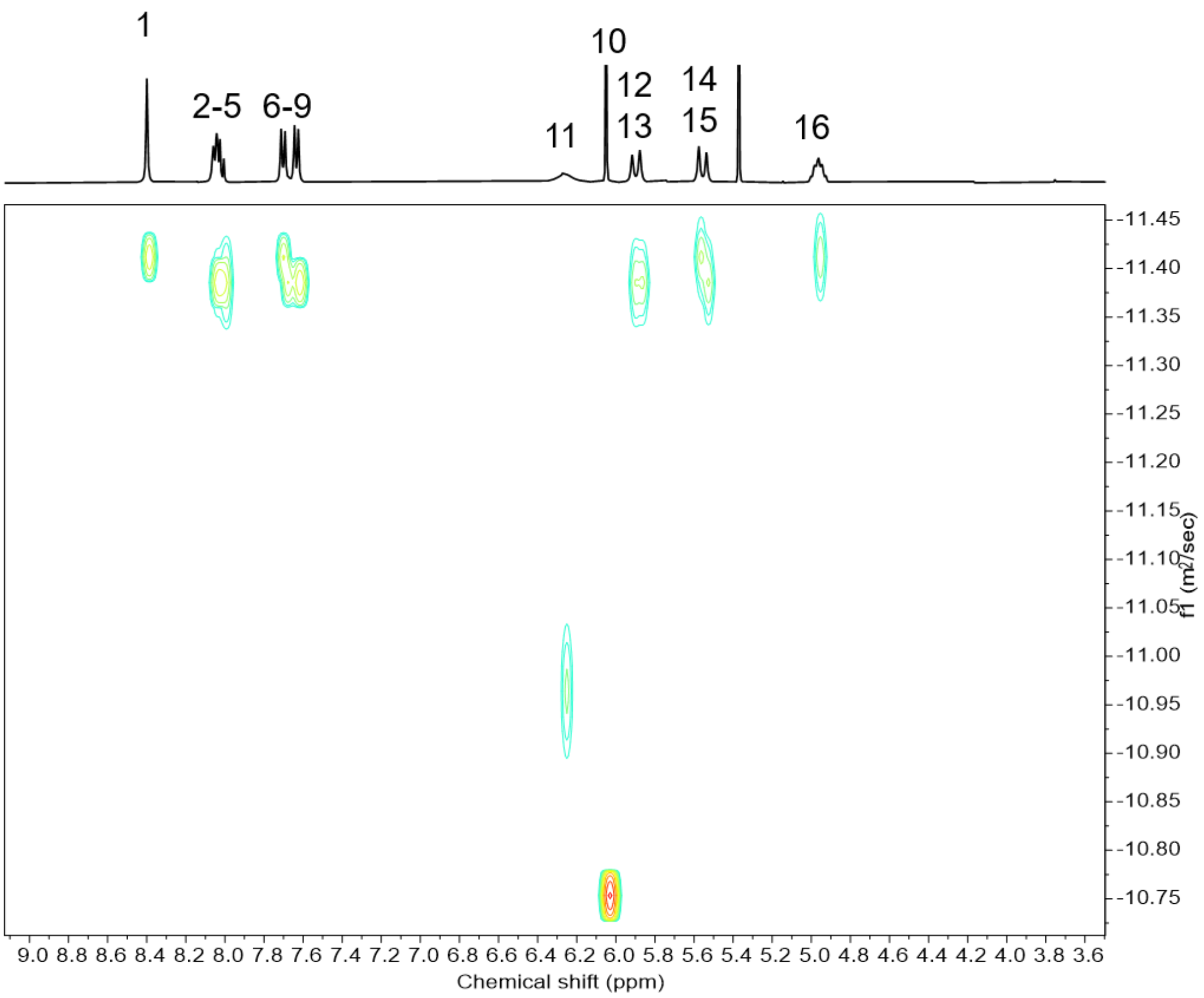

Figure S2.10: DOSY NMR spectrum of bis-PDI macrocycle **1-*MM*/*PP*** (TCE-d_2, 400 MHz, 298 K) with proton peaks labelled 1-16.

Table S2.2: Diffusion coefficients (*D*) for proton peaks 1-16 and their weighted mean obtained from the analysis of the DOSY NMR spectrum of bis-PDI macrocycle **1-*MM*/*PP***. We note that the solvent peak (10) and triazole peak (11) are not included in the Table, the latter being due to peak broadness, which is characteristic of intramolecular hydrogen bonding.

**Peaks**	***D*** **[m^2/s]**	***D*** **error [m^2/s]**
**1**	1.47E-10	4.67E-13
**2-5**	1.49E-10	1.77E-12
**6-9**	1.49E-10	2.59E-12
**12-13**	1.5E-10	2.31E-12
**14-15**	1.47E-10	2.06E-12
**16**	1.38E-10	5.89E-12
**Weighted mean**	1.47E-10	4.26E-13

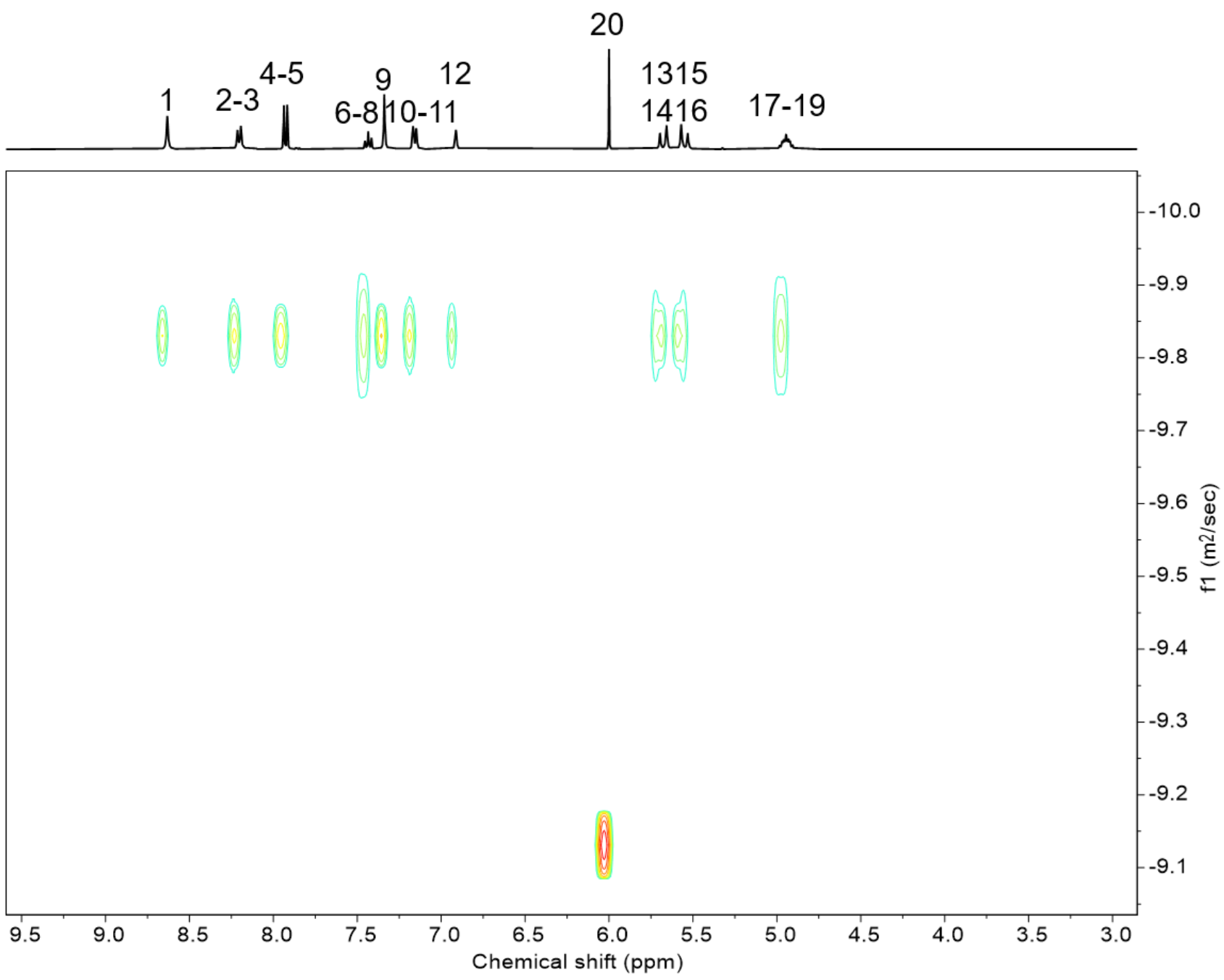

Figure S2.11: DOSY NMR spectrum of bis-PDI macrocycle **2-*MM*/*PP*** (TCE-d_2, 400 MHz, 298 K) with proton peaks labelled 1-20.

Table S2.3: Diffusion coefficients (*D*) for proton peaks 1-19 and their weighted mean obtained from the analysis of the DOSY NMR spectrum of bis-PDI macrocycle **2-*MM*/*PP***. We note that the solvent peak (20) is not included in the Table.

**Peaks**	***D* [m^2/s]**	***D* error [m^2/s]**
**1**	1.37E-10	6.39E-13
**2-3**	1.38E-10	1.38E-12
**4-5**	1.38E-10	9.17E-13
**6-8**	1.38E-10	3.83E-12
**9**	1.38E-10	3.10E-13
**10-11**	1.38E-10	1.40E-12
**12**	1.38E-10	1.08E-12
**13-14**	1.37E-10	1.80E-12
**15-16**	1.38E-10	1.80E-12
**17-19**	1.37E-10	3.60E-12
**Weighted mean**	1.38E-10	2.45E-13

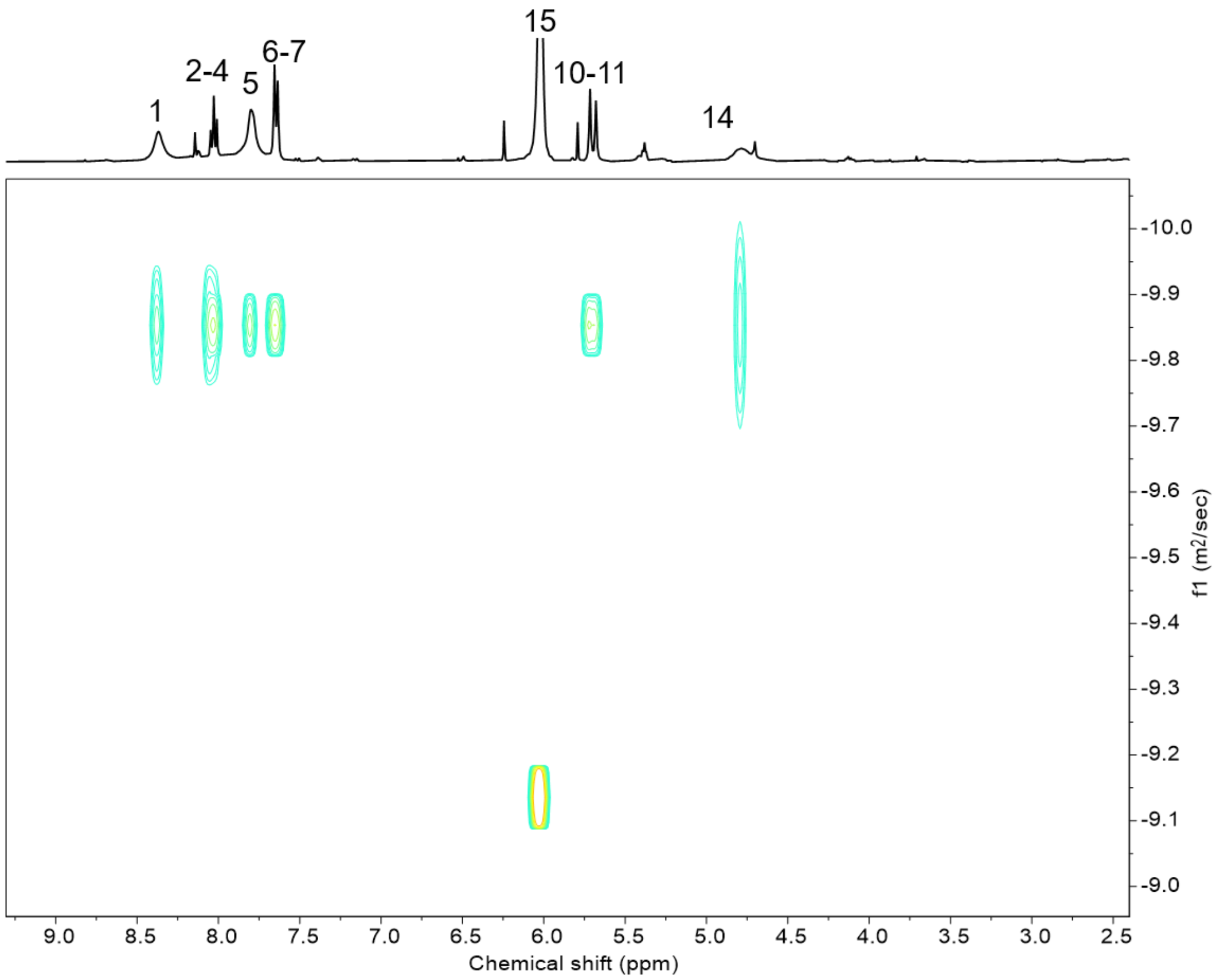

Figure S2.12: DOSY NMR spectrum of bis-PDI macrocycle **1-*MP*** (TCE-d_2, 400 MHz, 298 K) with proton peaks labelled 1-15.

Table S2.4: Diffusion coefficients (*D*) for proton peaks 1-14 and their weighted mean obtained from the analysis of the DOSY NMR spectrum of bis-PDI macrocycle **1-*MP***. We note that the solvent peak (15) is not included in the Table.

**Peaks**	***D* [m²/s]**	***D* error [m²/s]**
**1**	1.29E-10	2.62E-12
**2-4**	1.27E-10	3.60E-12
**5**	1.32E-10	1.56E-12
**6-7**	1.30E-10	1.30E-12
**10-11**	1.30E-10	1.69E-12
**14**	1.30E-10	6.18E-12
**Weighted mean**	1.30E-10	7.90E-13

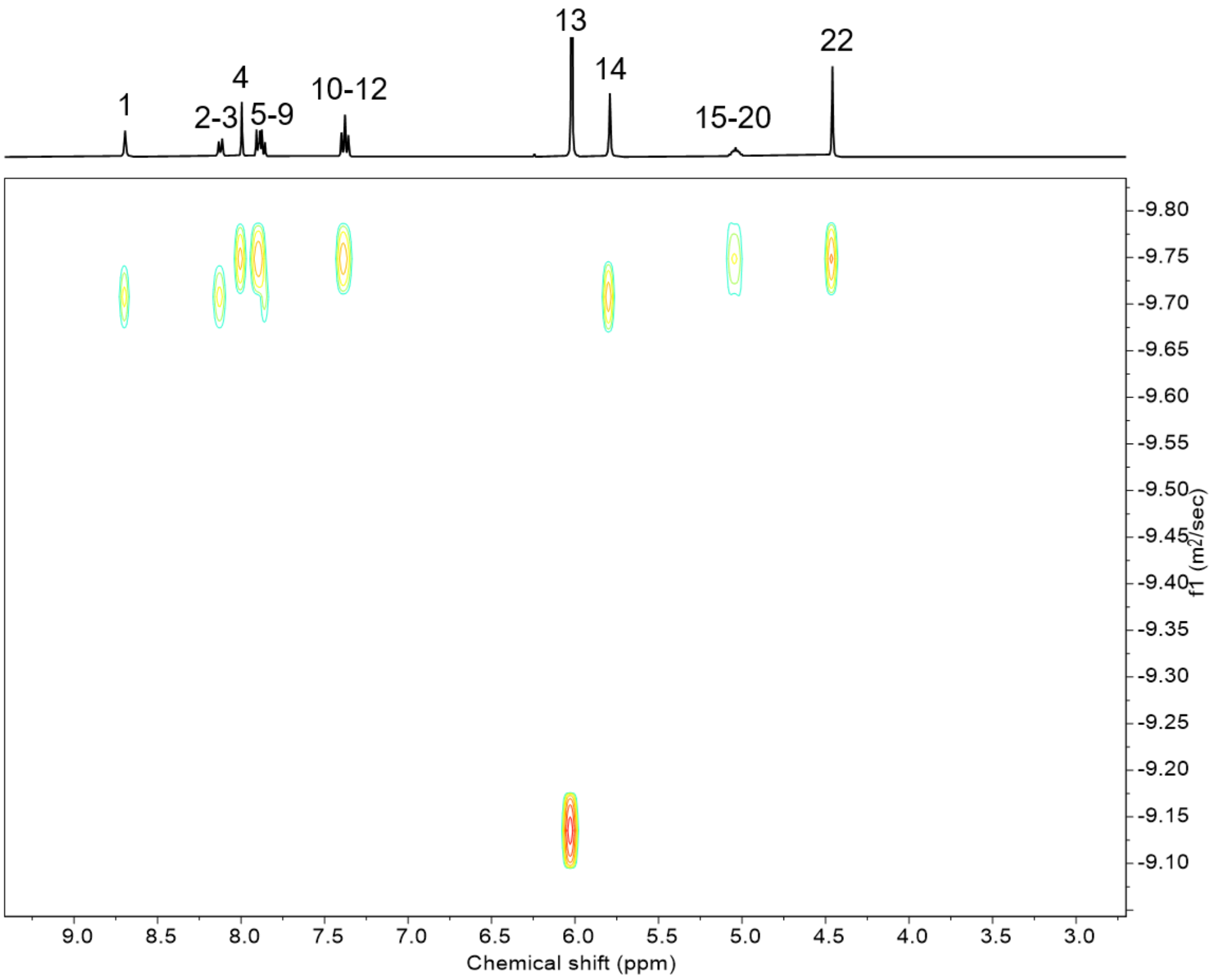

Figure S2.13: DOSY NMR spectrum of PDI monomer **5** (TCE-d_2, 400 MHz, 298 K) with proton peaks labelled 1-22.

Table S2.5: Diffusion coefficients (*D*) for proton peaks 1-22 and their weighted mean obtained from the analysis of the DOSY NMR spectrum of PDI monomer **5**. We note that the solvent peak (13) is not included in the Table.

Peaks	*D* [m²/s]	*D* error [m²/s]
**1**	1.71E-10	1.09E-12
**2-3**	1.71E-10	2.42E-12
**4**	1.68E-10	5.16E-13
**5-9**	1.68E-10	3.22E-12
**10-12**	1.69E-10	1.86E-12
**14**	1.77E-10	4.66E-13
**15-20**	1.67E-10	1.49E-11
**22**	1.69E-10	3.20E-13
**Weighted mean**	1.71E-10	2.26E-13

Table S2.6: A summary of the weighted average diffusion coefficients (*D*) from analysis of DOSY NMR spectra for PDI monomer **5** and bis-PDI macrocycles **1**-***MM/PP***, **1**-***MP*** and **2**-***MM/PP***.

**Compound**	***D*** $\times 10^{-10}$ ($m^2\,s^{-1}$)
**1**-***MM/PP***	1.4723 ± 0.0043
**1**-***MP***	1.3028 ± 0.0079
**2**-***MM/PP***	1.3783 ± 0.0024
Monomer **5**	1.7079 ± 0.0023

All the macrocycles have *D* that are lower than PDI monomer **5**, due to the larger size of these dimeric structures. Amongst the macrocycles, **1**-***MM/PP*** is the smallest, which is explained by the intramolecular hydrogen bonding that holds the co-facial π-stacked dimer together, with less conformational freedom and hence a more compact structure. Macrocycle **2**-***MM/PP*** is larger due to the absence of intramolecular hydrogen bonding, which leads to greater conformational flexibility. Heterochiral macrocycle **1**-***MP*** has a smaller *D* than both homochiral macrocycles due to the slipped stack, which leads to a larger structure.

3. Further photophysics

a) Beer-Lambert plots

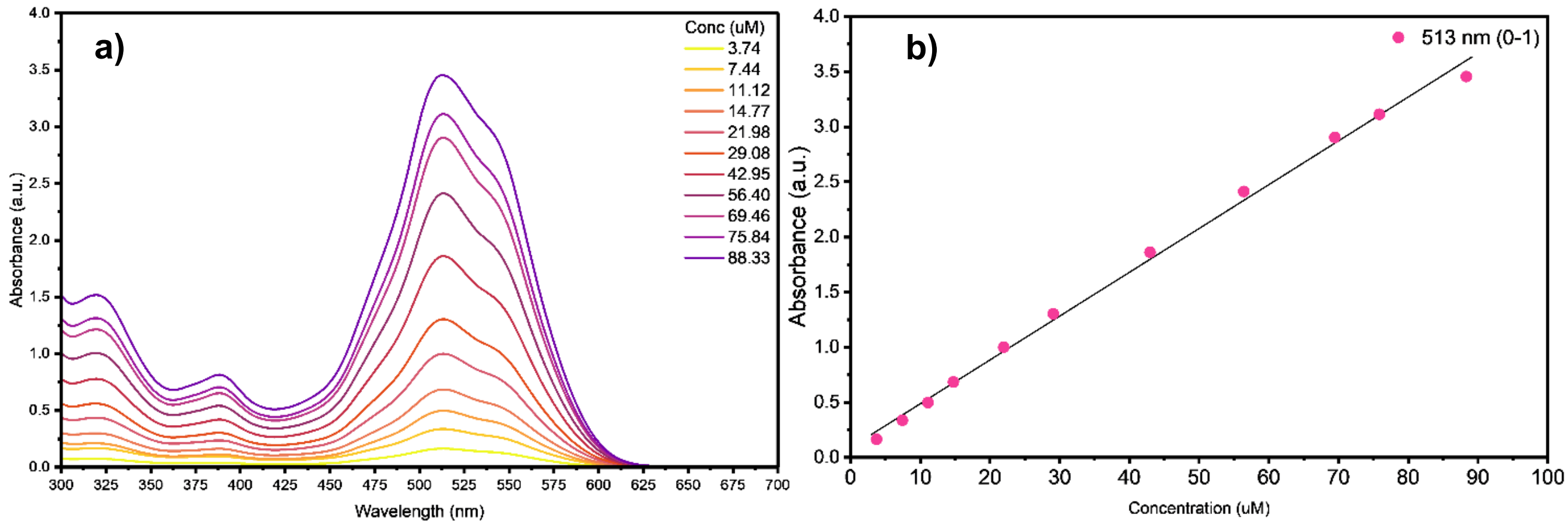

Figure S3.1: Absorption spectrum of macrocycle **1-*MM/PP*** in toluene at different concentrations (up to saturation of the instrument's detector). b) The dependence of λ_{max} = 513 nm on concentration for macrocycle **1-*MM/PP*** in toluene is linear, confirming Beer-Lambert behaviour.

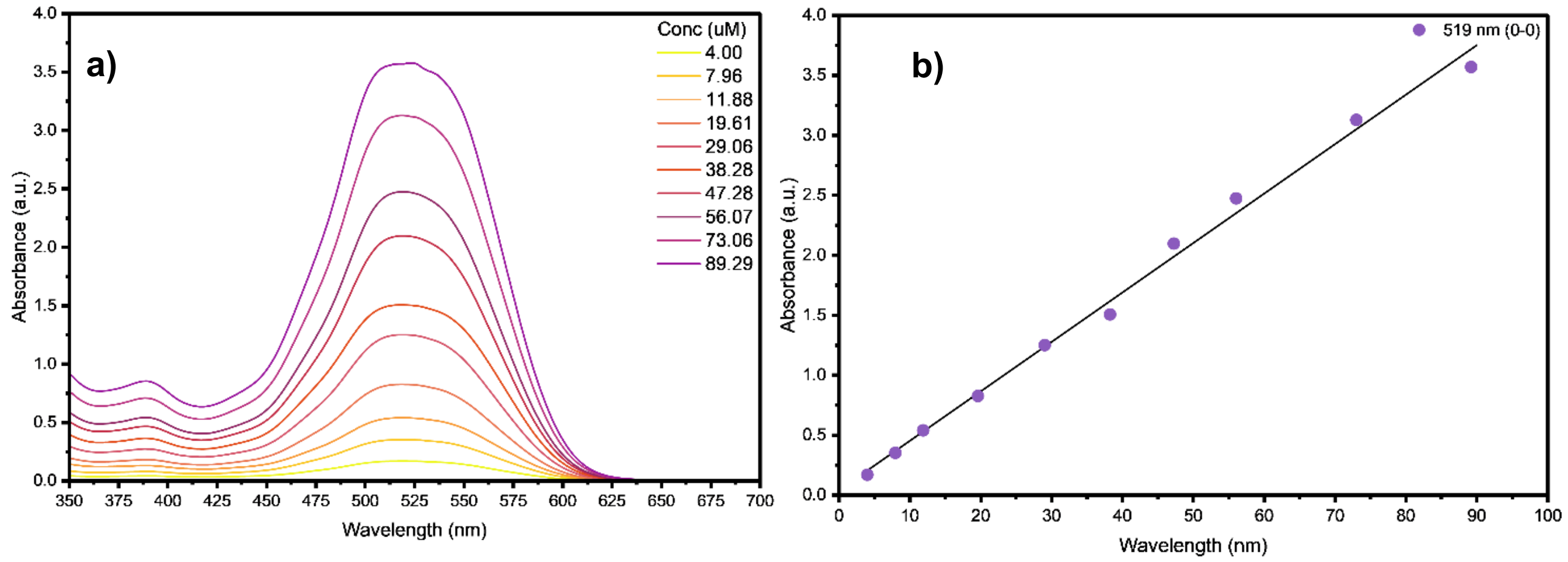

Figure S3.2: a) Absorption spectrum of macrocycle **2-*MM/PP*** in toluene at different concentrations (up to saturation of the instrument's detector). b) The dependence of λ_{max} = 519 nm on concentration for macrocycle **2-*MM/PP*** in toluene is linear, confirming Beer-Lambert behaviour.

b) Further absorption, excitation and fluorescence spectra

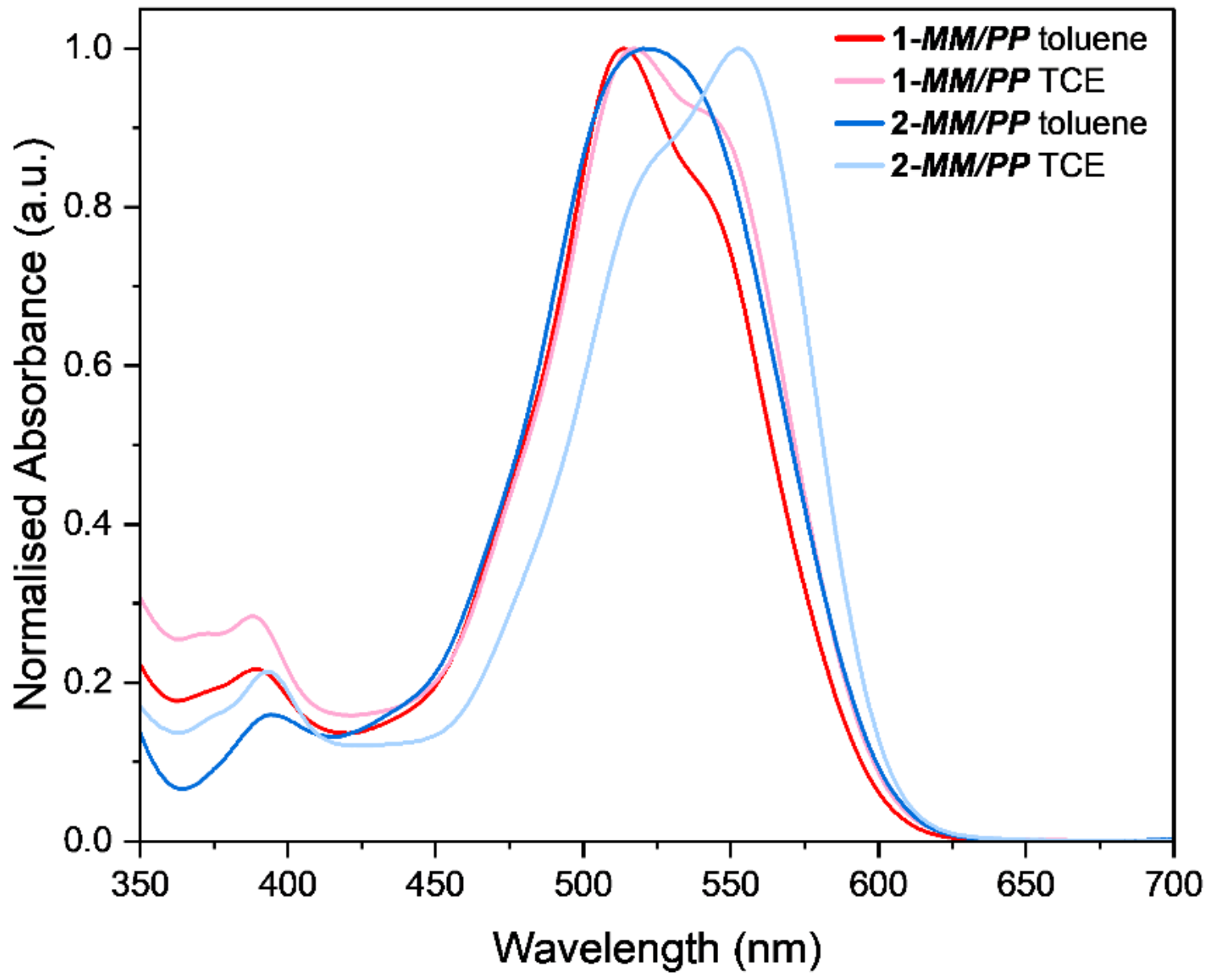

Figure S3.3: Normalised UV-vis spectra (11 μM) of **1-*MM/PP*** and **2-*MM/PP*** in toluene and 1,1,2,2-tetrachloroethane (TCE).

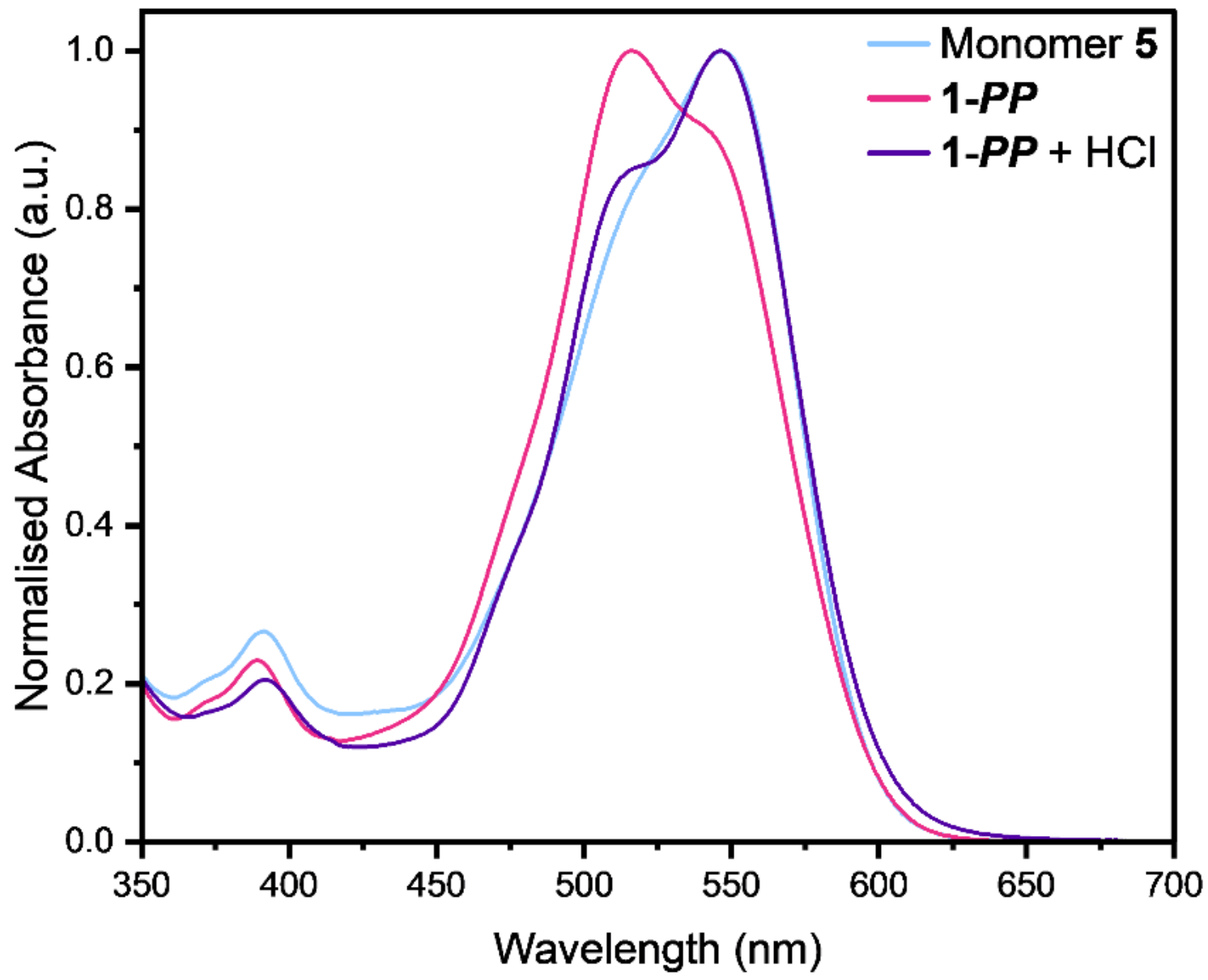

Figure S3.4: Normalised UV-vis spectra (11 μM) of PDI monomer **5**, **1-*PP*** and **1-*PP*** + HCl in 1,1,2,2-tetrachloroethane. Upon protonation, the UV-vis spectrum of macrocycle **1-*PP*** resembles that of monomer **5**

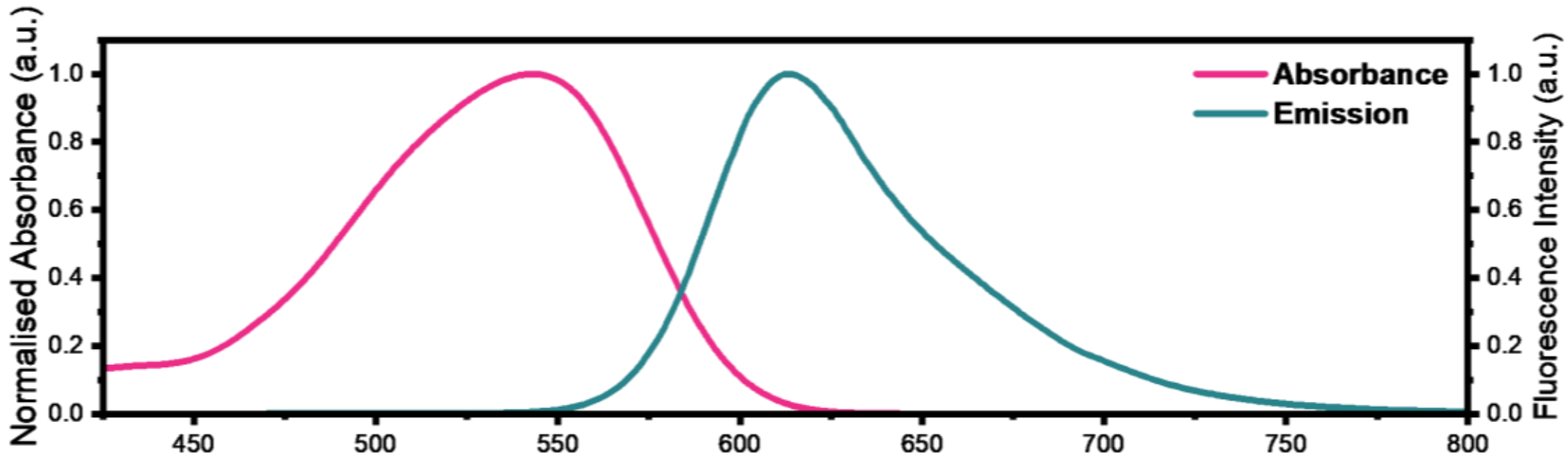

Figure S3.5: Normalised UV-vis (11 μM) and fluorescence emission spectra (11 μM, λ_{ex} = 450 nm) for **2-*MP*** in toluene

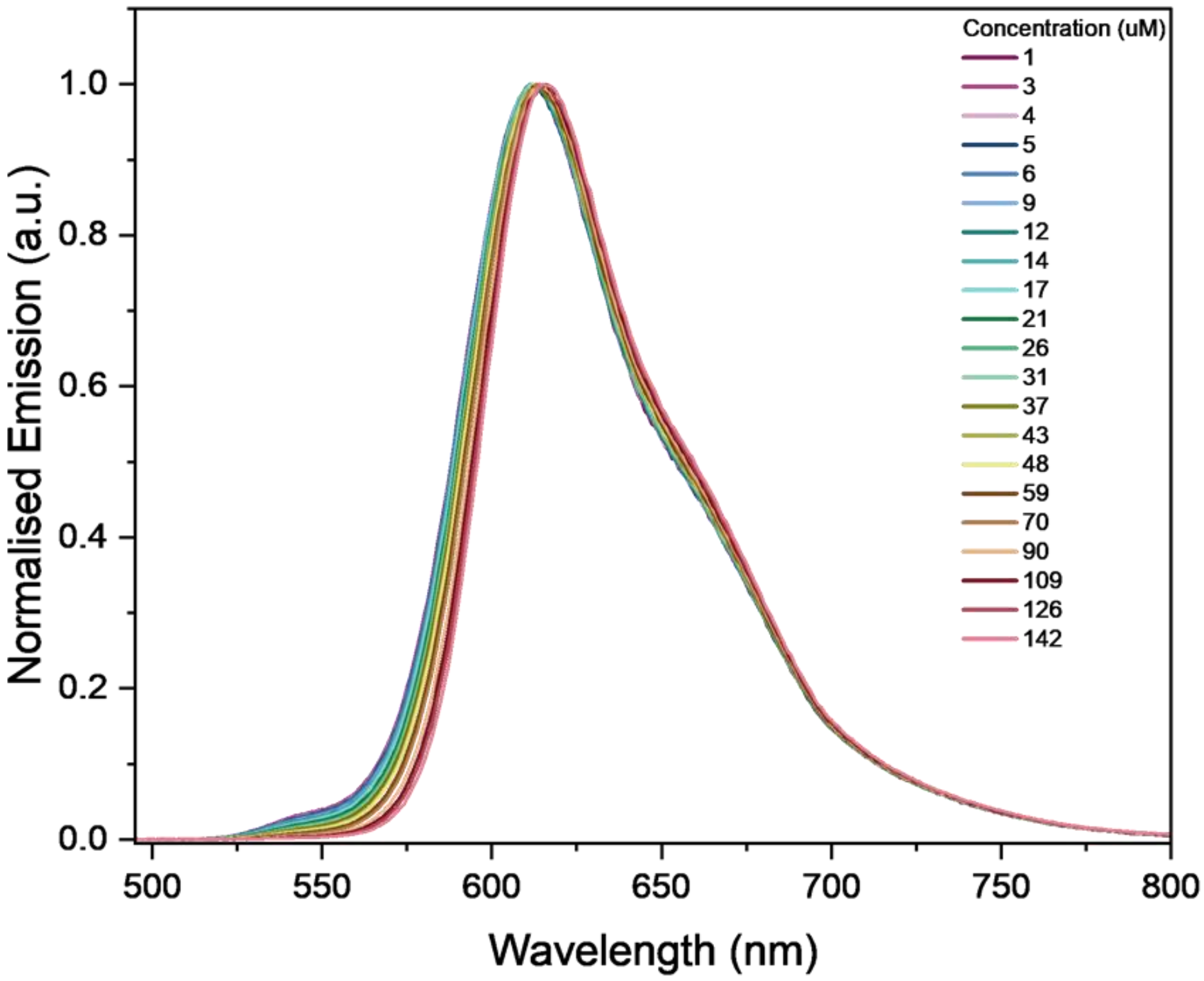

Figure S3.6: Normalised fluorescence emission spectrum of **1-*PP*** in toluene at varying concentrations spanning two orders of magnitude (1-142 μM, λ_{ex} = 450 nm). As the absorption spectrum remains unchanged over the investigated concentration range (Fig. S3.1), the concentration-dependent redshift/narrowing of the fluorescence spectrum is due to secondary inner filter effect i.e. self-absorption. Specifically, overlap between the absorption tail and the blue side of the emission results in preferential reabsorption of higher-energy fluorescence photons, producing an apparent narrowing and redshift of the emission without requiring any change in the intrinsic emissive state.

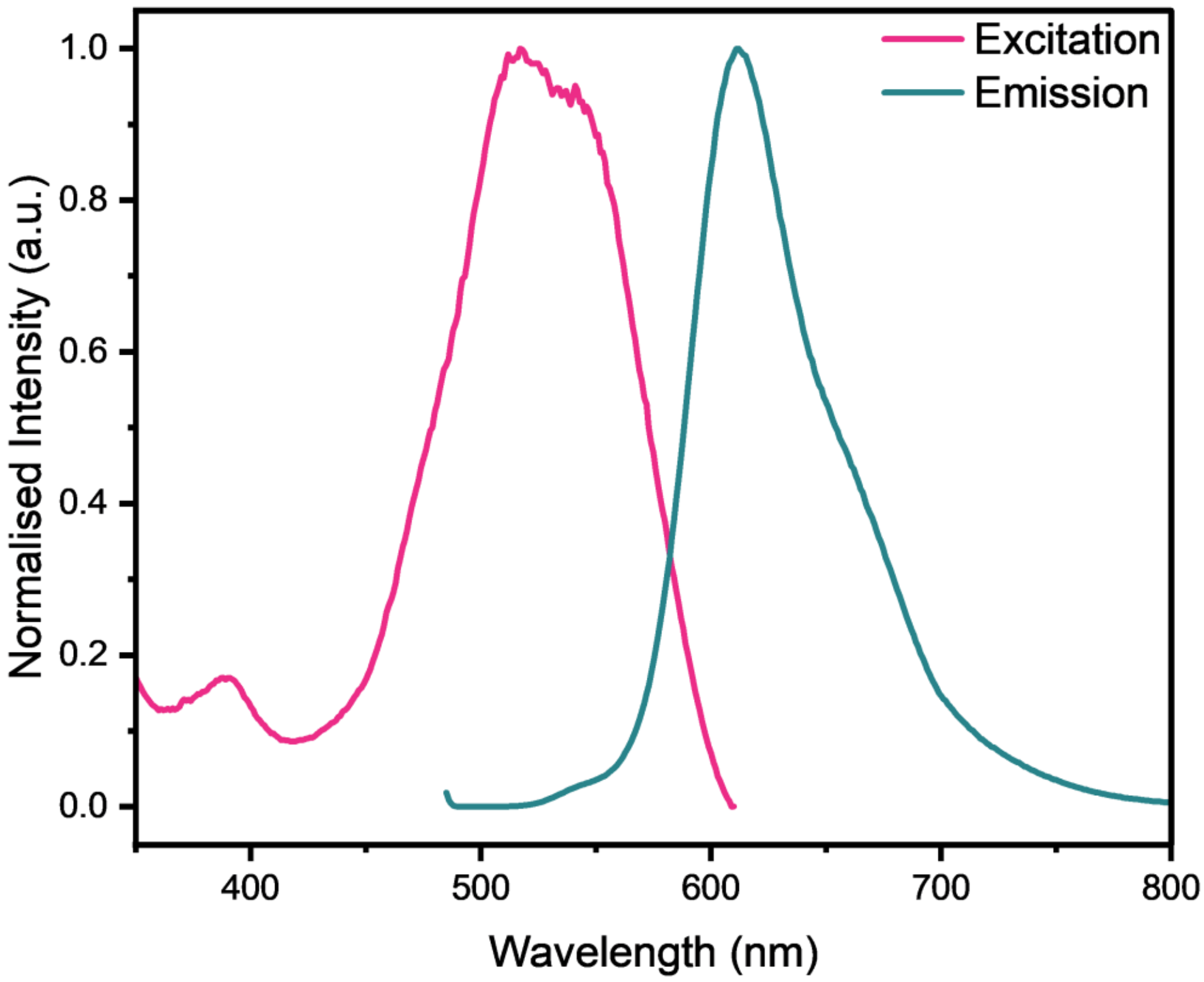

Figure S3.7: Excitation (7 μM, λ_{em} = 613 nm) and fluorescence emission (7 μM, λ_{ex} = 450 nm) spectra in toluene of bis-PDI macrocycle **1**-***MM/PP***. The excitation spectrum is consistent with the absorption spectrum of **1**-***MM/PP***.

4. X-ray diffraction

a) Single crystal X-ray diffraction

Crystals of racemic **1-*MM/PP*** were obtained by slow diffusion of MeOH into a solution of macrocycle in $CHCl_3$. Glass vials for crystallisation were coated with Sigmacote® siliconizing agent (Merck, SL2) prior to use. Diffraction experiments for the resulting reddish-purple lath-shaped crystals were performed by the UK EPSRC National Crystallography Service at the University of Southampton[8] on a Rigaku 007HF diffractometer with HF Varimax confocal mirrors, an UG2 goniometer and HyPix 6000HE detector at 100 K using Cu Kα radiation and processed using CrysAlisPro 1.171.43. The structure was solved by direct methods using ShelXT[18] and refined with ShelXL[19] using a least squares method. Olex2 software was used as the solution, refinement and analysis program.[9] Hydrogen atoms were placed geometrically and refined using a riding model. Constraints and restraints were applied as seen fit. The CCDC deposition number is: 2558754.

Crystal Data for $C_{92}H_{82}N_{18}O_{10}$ (M =1599.75 g/mol): orthorhombic, space group $Pna2_1$ (no. 33), a = 16.1627(8) Å, b = 19.9482(14) Å, c = 23.9220(16) Å, V = 7712.9(8) Å^3, Z = 4, T = 99.99(10) K, μ(Cu Kα) = 0.751 mm^{-1}, $Dcalc$ = 1.378 g/cm^3, 34415 reflections measured (5.768° ≤ 2Θ ≤ 133.17°), 11622 unique (R_{int} = 0.1606, R_{sigma} = 0.1849) which were used in all calculations. The final R_1 was 0.1176 (I > 2σ(I)) and wR_2 was 0.3566 (all data).

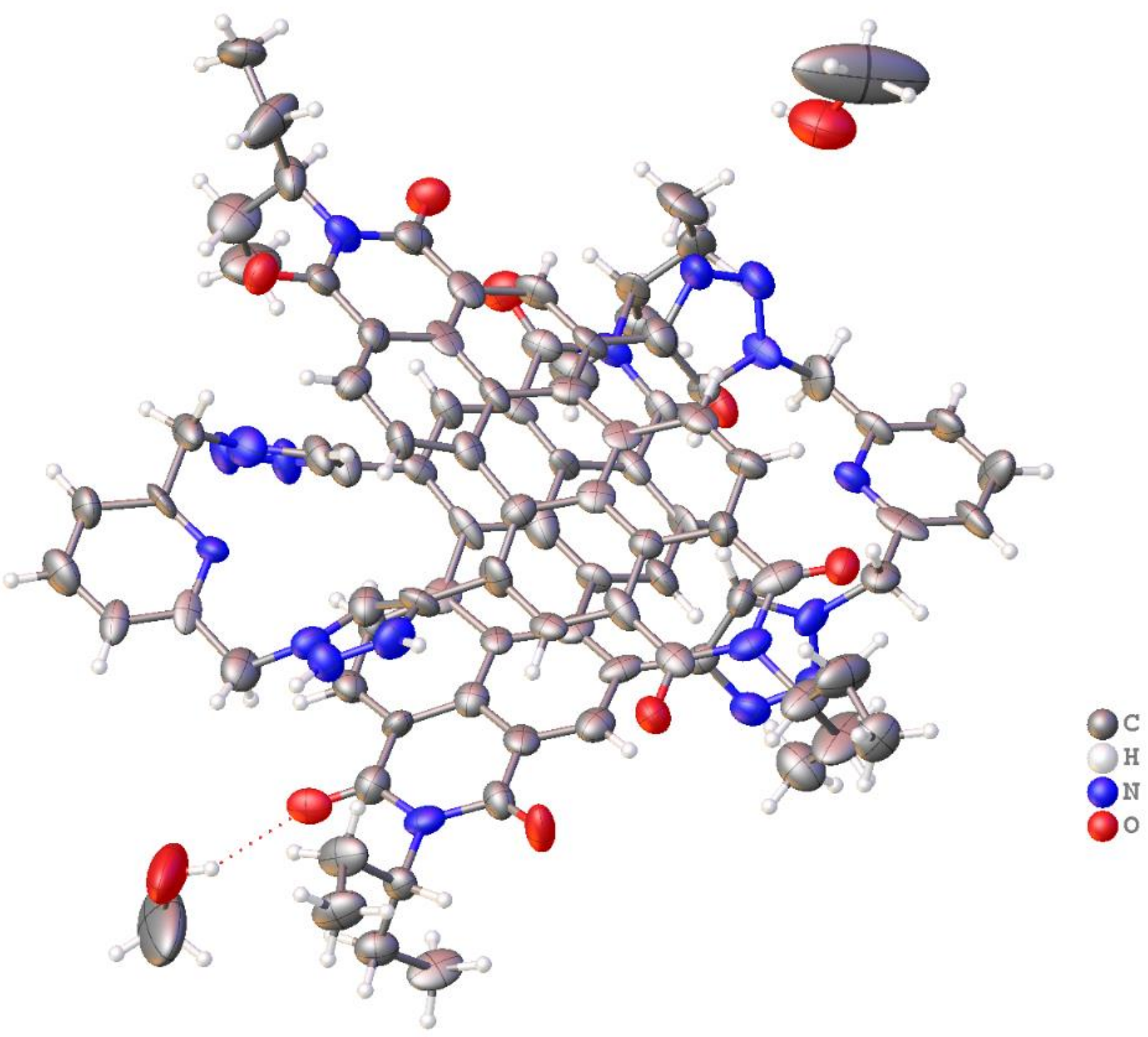

Figure S4.1: Asymmetric unit of racemic crystals of (homochiral) **1-*MM/PP***. Thermal ellipsoids indicate 50% probability.

Similarly, crystals of **1-*MP*** were obtained by slow diffusion of MeOH into a solution of macrocycle in $CHCl_3$. Glass vials for crystallisation were coated with Sigmacote® siliconizing agent (Merck, SL2) prior to use. Diffraction experiments for the resulting reddish-purple lath-shaped crystals were performed by the UK EPSRC National Crystallography Service on a Rigaku 007HF diffractometer with HF Varimax confocal mirrors, an UG2 goniometer and HyPix 6000HE detector at 100 K using Cu Kα radiation and processed using CrysAlisPro 1.171.43. The structure was solved by direct methods using ShelXT[18] and refined with ShelXL[19] using a least squares method. Olex2 software was used as the solution, refinement and analysis program.[7] Hydrogen atoms were placed geometrically and refined using a riding model. Constraints and restraints were applied as seen fit.

Data quality for **1-*MP*** was poor, with negligible diffraction past 1 Å. Despite multiple attempts, better crystals could not be obtained. Several atoms had to be refined isotropically as they appeared NPD when refined anisotropically. A solvent mask also had to be applied to the structure. It was found that there was residual electron density corresponding to $CHCl_3$ at <1 occupancy and with disorder, which could not be accurately modelled due to the quality of the data. Using the mask, the residual electron density (192 e-) was found to correspond to 3.5 $CHCl_3$/unit cell (203 e-). Due to the data quality issues, this structure is only used to confirm connectivity and geometry of the molecule. The CCDC deposition number is: 2558753.

Crystal Data for $C_{181.75}H_{147.75}Cl_{5.25}N_{36}O_{16}$ (M =3278.21 g/mol): monoclinic, space group P2$_1$/c (no. 14), a = 15.543(2) Å, b = 14.6761(18) Å, c = 34.718(4) Å, β = 91.431(15)°, V = 7917.1(17) Å^3, Z = 2, T = 100.00(10) K, μ(Cu Kα) = 1.523 mm^{-1}, $Dcalc$ = 1.375 g/cm^3, 29272 reflections measured (5.092° ≤ 2Θ ≤ 100.866°), 8207 unique (R_{int} = 0.2032, R_{sigma} = 0.2681) which were used in all calculations. The final R_1 was 0.2131 (I > 2σ(I)) and wR_2 was 0.5535 (all data).

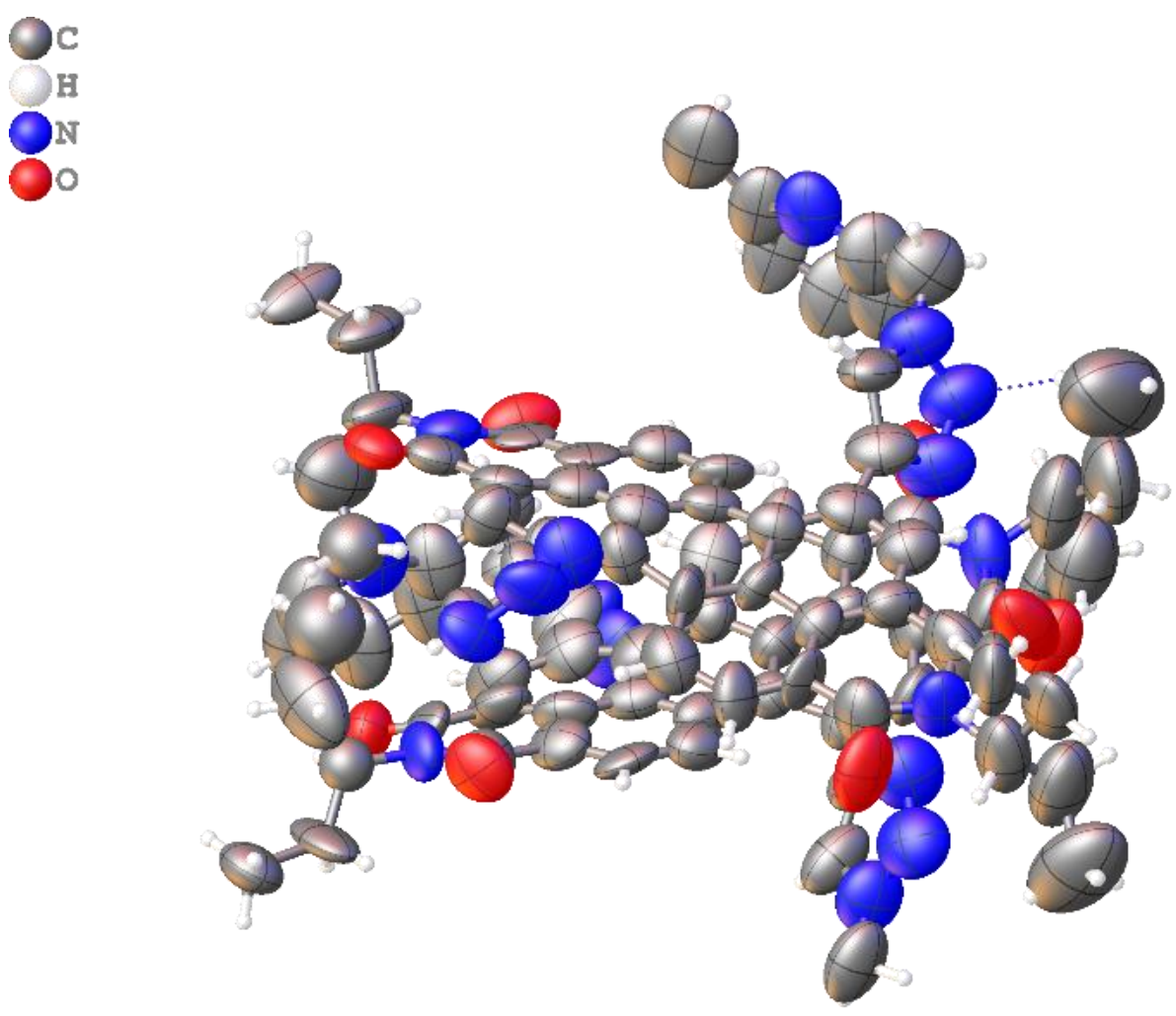

Figure S4.2: Asymmetric unit of crystals of (heterochiral) **1-*MP***. Thermal ellipsoids indicate 50% probability.

Crystals of enantiopure **1-*PP*** were obtained by slow diffusion of MeOH into a solution of macrocycle in $CHCl_3$. The obtained purple crystals were measured on a Rigaku Synergy-S dual source diffractometer with a PhotonJet-S X-ray source and a HyPix-6000 detector. Data were collected at 100 K through the use of an Oxford Cryosystems cryostream device using Cu Kα radiation. Data were collected and processed using CrysAlisPro 1.171.43. The structure was solved by direct methods using ShelXT[18] and refined with ShelXL[19] using a least squares method. Olex2 software was used as the solution, refinement and analysis program.[9] Hydrogen atoms were placed geometrically and refined using a riding model. Constraints and restraints were applied as seen fit. A solvent mask was applied to the structure, with 102 e^- residual electron density per asymmetric unit corresponding to ~5 MeOH molecules (90 e^-). The CCDC deposition number is: 2558750.

Crystal Data for $C_{192}H_{190}Cl_6N_{36}O_{26}$ (M =3630.49 g/mol): monoclinic, space group $P2_1$ (no. 4), a = 8.3690(2) Å, b = 24.9347(5) Å, c = 21.3995(5) Å, β = 90.479(2)°, V = 4465.46(17) $Å^3$, Z = 1, T = 100.00(10) K, μ(Cu Kα) = 1.544 mm^{-1}, $Dcalc$ = 1.350 g/cm^3, 16720 reflections measured (8.208° ≤ 2Θ ≤ 158.326°), 10350 unique (R_{int} = 0.0486, R_{sigma} = 0.0682) which were used in all calculations. The final R_1 was 0.0641 (I > 2σ(I)) and wR_2 was 0.1845 (all data).

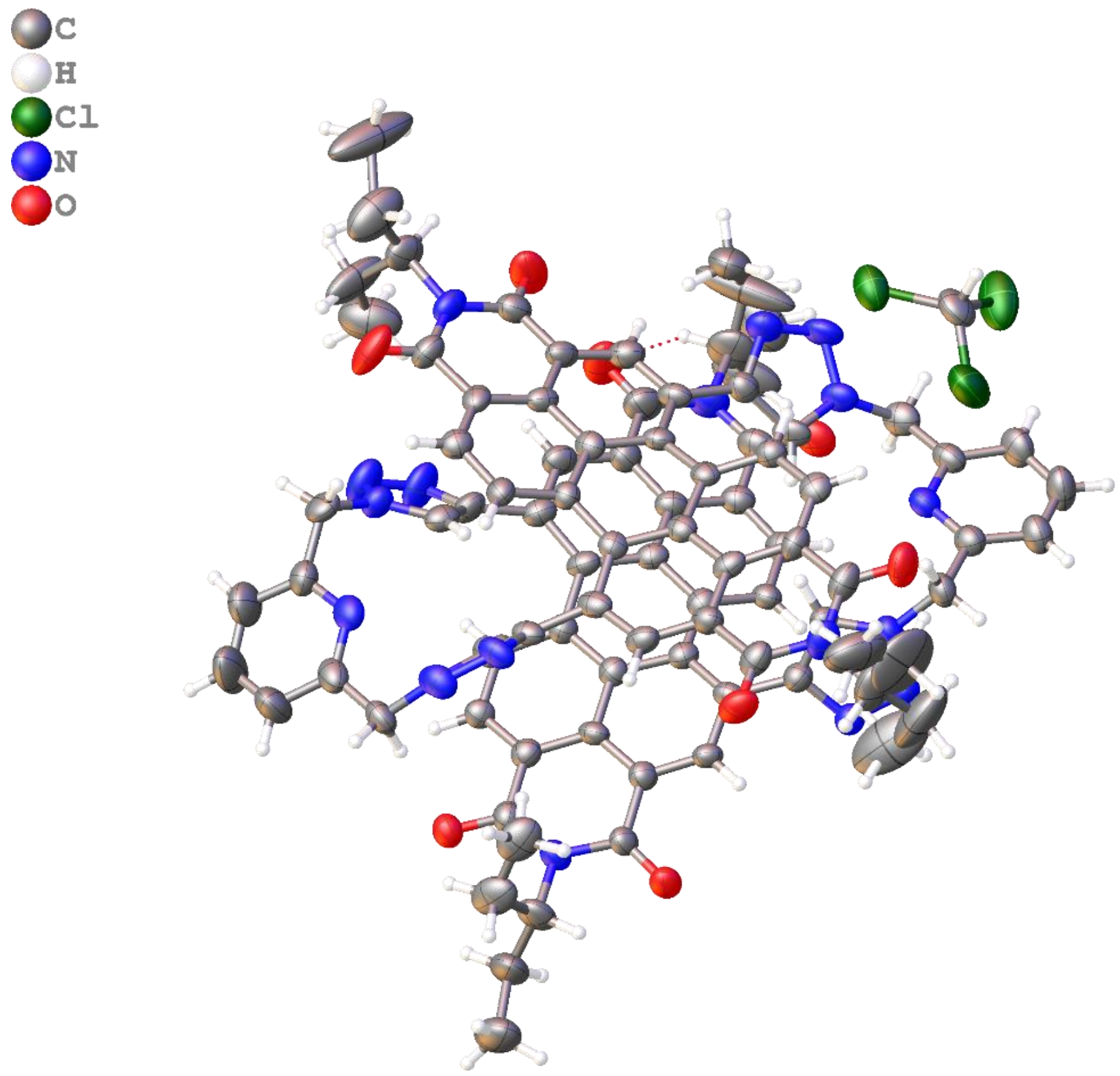

Figure S4.3*:* Asymmetric unit of crystals of enantiopure **1-*PP***. Thermal ellipsoids indicate 50% probability.

A second set of crystals of enantiopure **1-*PP*** were also obtained from the same conditions. Diffraction experiments for the resulting violet needles were performed by the UK EPSRC National Crystallography Service on a Rigaku 007HF diffractometer with HF Varimax confocal mirrors, an UG2 goniometer and HyPix 6000HE detector at 100 K using Cu Kα radiation and processed using CrysAlisPro 1.171.43. The structure was solved by direct methods using ShelXT[18] and refined with ShelXL[19] using a least squares method. Olex2 software was used as the solution, refinement and analysis program.[9] Hydrogen atoms were placed geometrically and refined using a riding model. Constraints and restraints were applied as seen fit.

The quality of these crystals was poor, with only negligible diffraction past 1 Å. A solvent mask was applied, with the residual electron density per asymmetric unit (162 e^-) corresponding to 9 MeOH molecules (162 e^-). Disorder was also seen in the structure for both the refined chloroform and the imide chains, which could not be accurately modelled due to a lack of electron density. The resulting data is therefore only used to confirm connectivity. The CCDC deposition number is: 2558751.

Crystal Data for $C_{100}H_{111}Cl_3N_{18}O_{17}$ (M =1943.41 g/mol): monoclinic, space group I2 (no. 5), a = 19.5509(10) Å, b = 14.1182(6) Å, c = 34.201(2) Å, β = 94.268(5)°, V = 9414.1(8) Å^3, Z = 4, T = 100.00(10) K, μ(Cu Kα) = 1.532 mm^{-1}, $Dcalc$ = 1.371 g/cm^3, 54556 reflections measured (5.05° ≤ 2Θ ≤ 96.91°), 9021 unique (R_{int} = 0.1589, R_{sigma} = 0.0807) which were used in all calculations. The final R_1 was 0.0742 (I > 2σ(I)) and wR_2 was 0.2103 (all data).

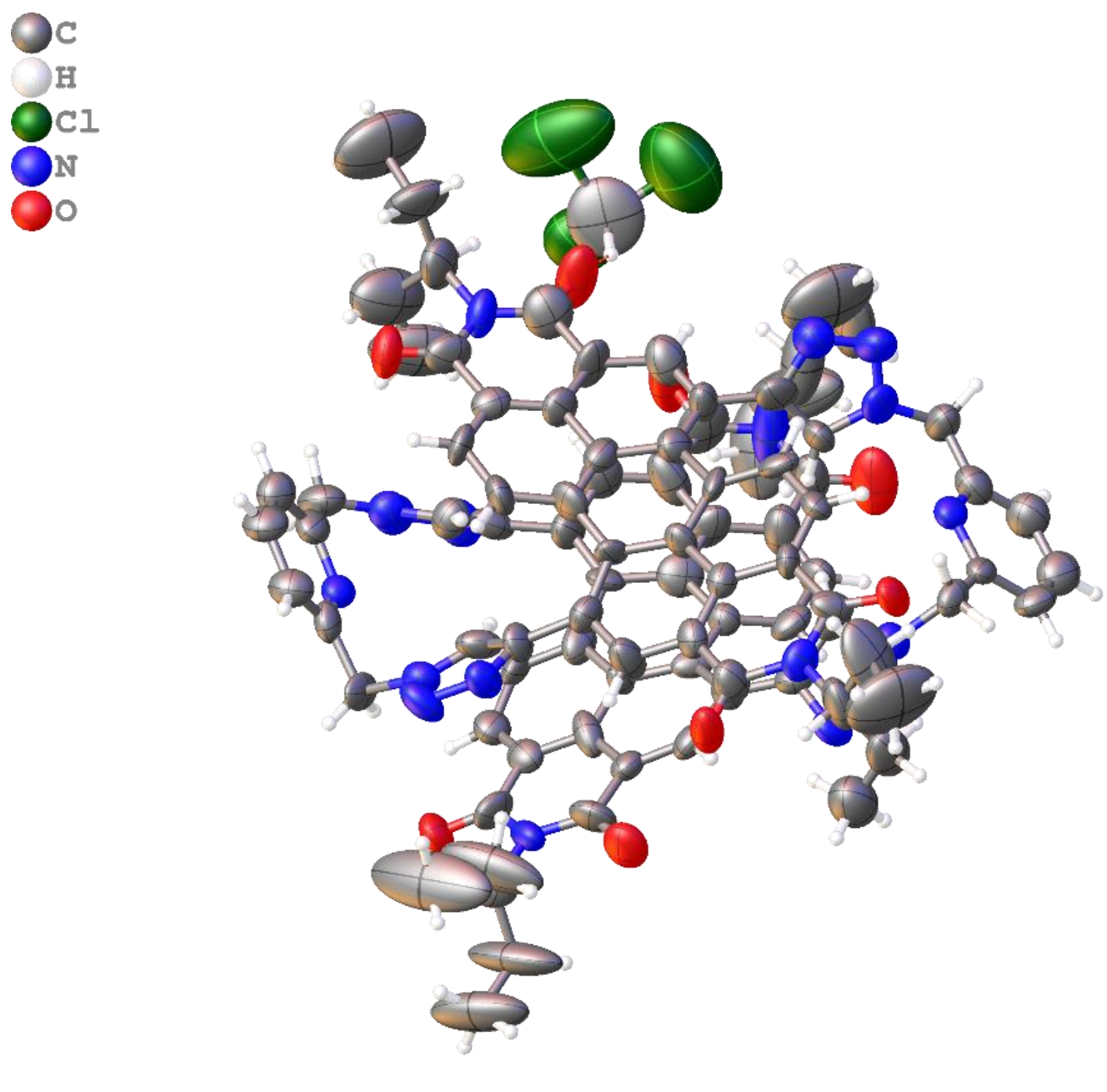

Figure S4.4: Asymmetric unit of crystals of enantiopure **1-*PP***. Thermal ellipsoids indicate 50% probability.

In both cases, enantiopure crystals of the opposite enantiomer, **1-*MM***, were obtained using similar conditions. These were confirmed to have the same unit cell parameters.

Crystals of **2-*MP*** were obtained by slow diffusion of MeOH into a solution of macrocycle in $CHCl_3$. The obtained purple plates were measured on beamline I19 at the UK Diamond Light Source on a 3-circle diffractometer (λ = 0.6889 Å).[10–12] A suitable single crystal was selected and mounted using fomblin film on a MiTeGen micromount. Data were collected on a Dectris PILATUS 2M detector, with the detector distance set at 300 mm. The crystals were kept at 100 K during data collection (single omega sweep). The structure was solved by direct methods using ShelXT[18] and refined with ShelXL[19] using a least squares method. Olex2 software was used as the solution, refinement and analysis program. [9] Hydrogen atoms were placed geometrically and refined using a riding model. Despite multiple attempts at crystal growth, better crystals could not be obtained. Negligible diffraction was observed past 1 Å for the crystals, which affected the data quality. The crystals were also found to be twinned (twin law -1 0 0 | 0 1 0 | 0.5 0 1) and the resulting data was therefore processed with the HKLF 5 program.[13] Furthermore, disorder was observed in the structure, and a solvent mask had to be applied, with the residual electron density per asymmetric unit (30 e^-) corresponding to 1.75 MeOH molecules (32 e^-) and the refined chloroform atom at <1 occupancy. The overall quality of the structure is low and is only used for confirmation of connectivity. The CCDC deposition number is: 2558752.

Crystal Data for $C_{187.899}H_{165.051}Cl_{3.311}N_{32}O_{19.5}$ (M =3300.72 g/mol): monoclinic, space group $P2_1/c$ (no. 14), a = 15.8160(5) Å, b = 14.6958(3) Å, c = 35.6043(8) Å, β = 96.018(3)°, V = 8229.9(4) $Å^3$, Z = 2, T = 100 K, μ(Synchrotron) = 0.132 mm^{-1}, $Dcalc$ = 1.332 g/cm^3, 8632 reflections measured (2.23° ≤ 2Θ ≤ 40.366°), 8632 unique (R_{sigma} = 0.0444) which were used in all calculations. The final R_1 was 0.1363 (I > 2σ(I)) and wR_2 was 0.4015 (all data).

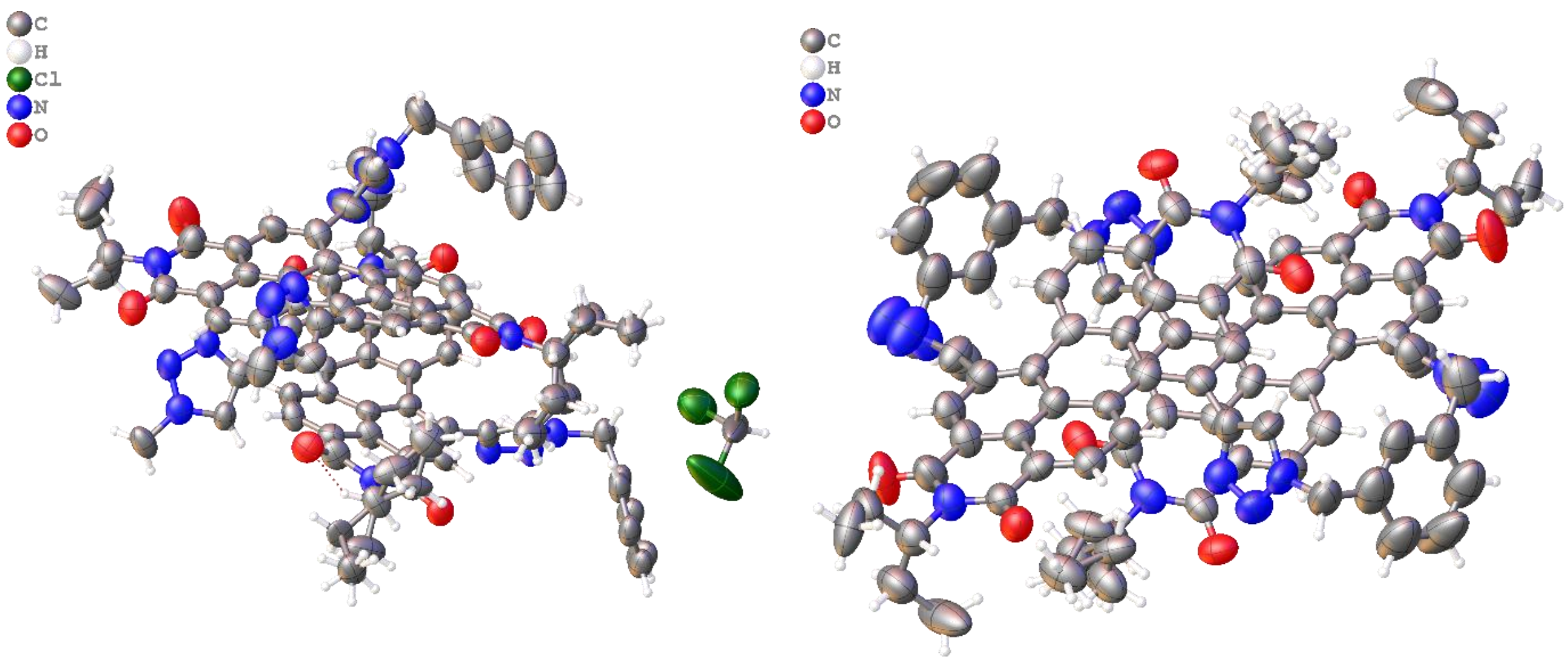

Figure S4.5: Asymmetric unit and complete macrocyclic structure of crystals of (heterochiral) **2-*MP***. Thermal ellipsoids indicate 50% probability

b) Powder X-ray diffraction

Powder X-ray diffraction experiments for the macrocycle **1** thin films samples were performed using a Rigaku Synergy-S dual source with a PhotonJet-S X-ray source and a HyPix-6000 detector using Cu radiation (λ = 1.5406 Å). The film was removed from the substrate with a spatula and the powder mounted on a MiTeGen micromount. The sample was kept at 100(2) K during data collection through the use of an Oxford Cryosystems cryostream device. Data were collected and processed using CrysAlis PRO171.42.49.

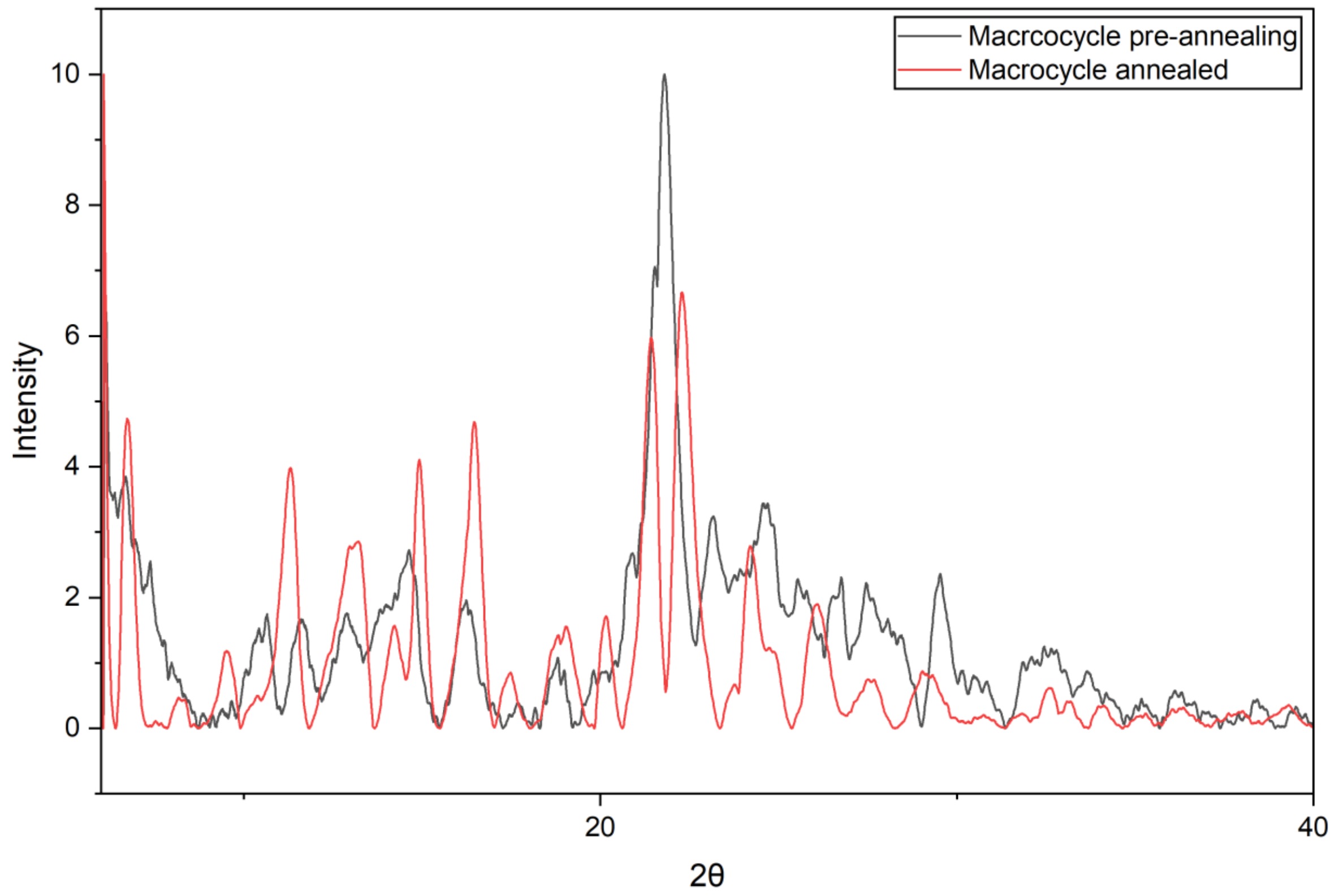

Figure S4.6: Powder X-ray diffractograms of bis-PDI macrocycle **1**-***PP*** thin films both prior to, and post, thermal annealing.

The diffractograms (Fig. S4.6) confirm that multiple crystalline phases are present in the pre-annealed sample, and that annealing results in a more uniformly crystalline sample as evidenced by the presence of fewer, more well-defined peaks. These observations are consistent with the formation of a more ordered molecular packing arrangement upon thermal treatment.

5. Chiral HPLC

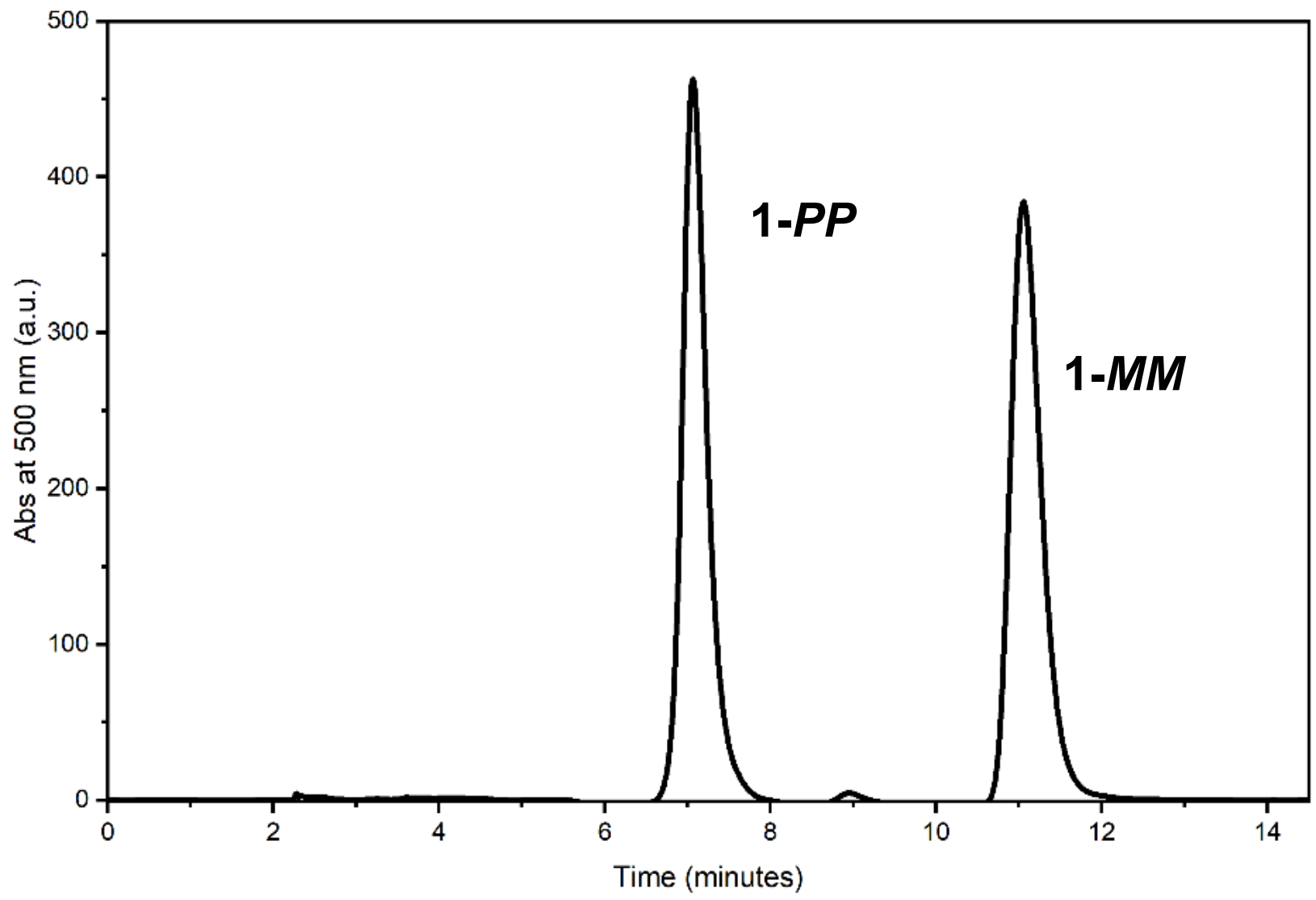

Figure S5.1: Chiral HPLC chromatogram (Phenomenex i-Amylose-1, 250 x 4.6 mm) of **1-*MM*/*PP*** dissolved in 1:1 DCM:n-hexane eluted with a gradient of 38:2:60 to 78:3:19 of DCM:IPA:n-hexane over eight minutes.

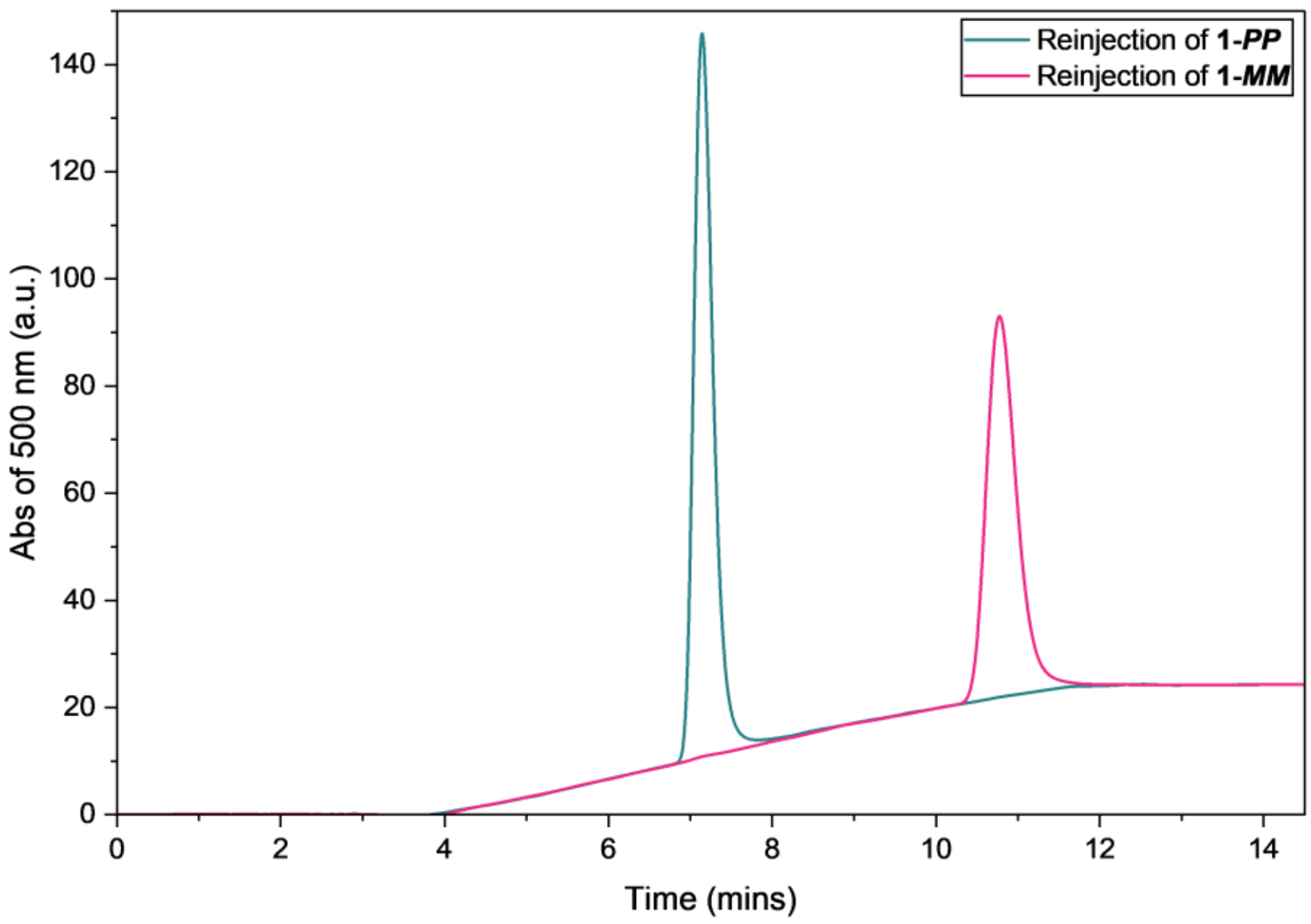

Figure S5.2: Chiral HPLC chromatogram (Phenomenex i-Amylose-1, 250 x 4.6 mm) of **1-*PP*** and **1-*MM***, previously purified by chiral HPLC. The enantiomers were left in solution (1:1 DCM:n-hexane) for one week at room temperature and then reinjected, revealing no racemisation.

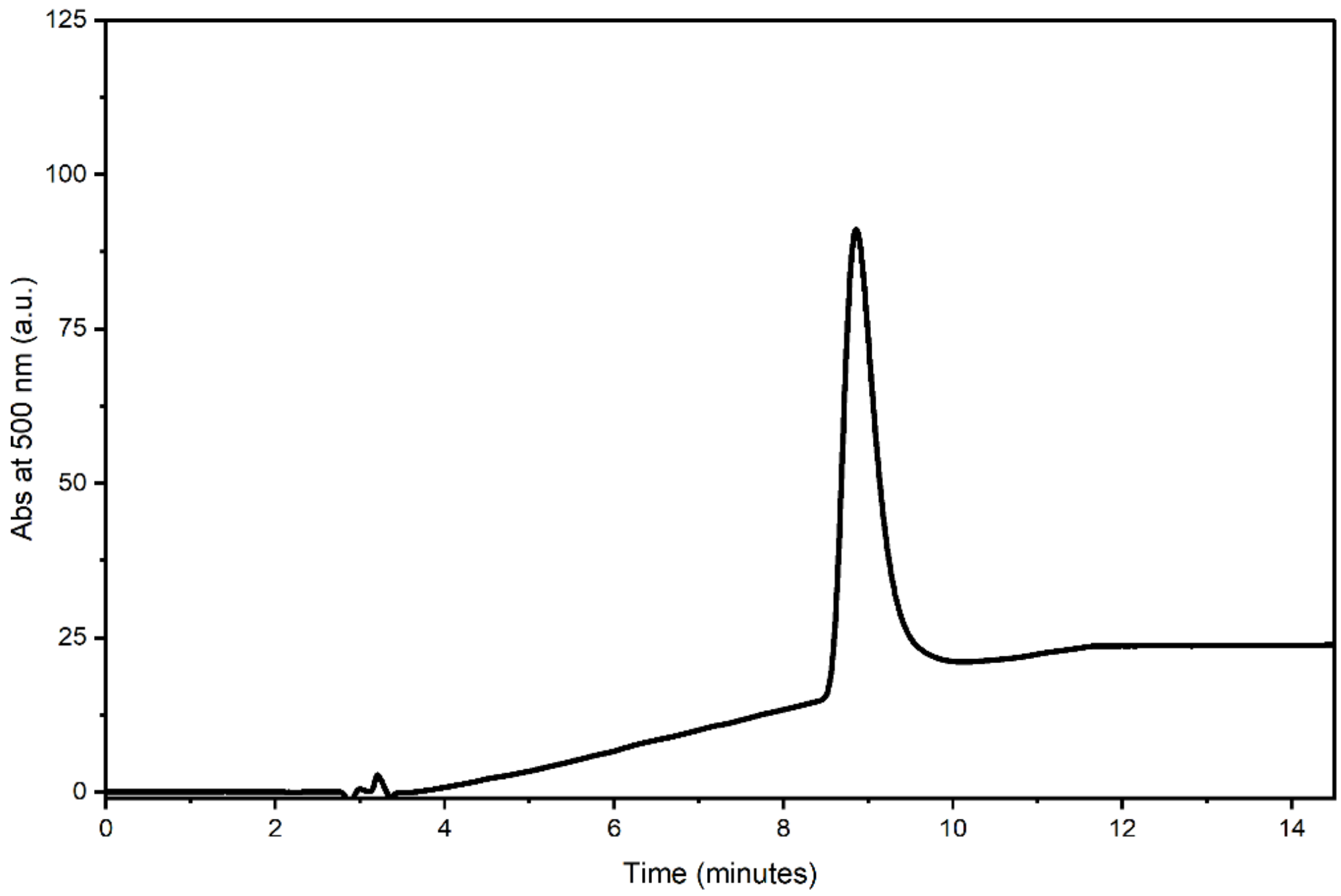

Figure S5.3: Chiral HPLC chromatogram (Phenomenex i-Amylose-1, 250 x 4.6 mm) of **1-*MP*** dissolved in 1:1 DCM:n-hexane with a gradient over 8 minutes of 38:2:60 to 78:3:19 of DCM:IPA:n-hexane. The macrocycle is achiral (the heterochiral meso isomer) and so a single peak is seen.

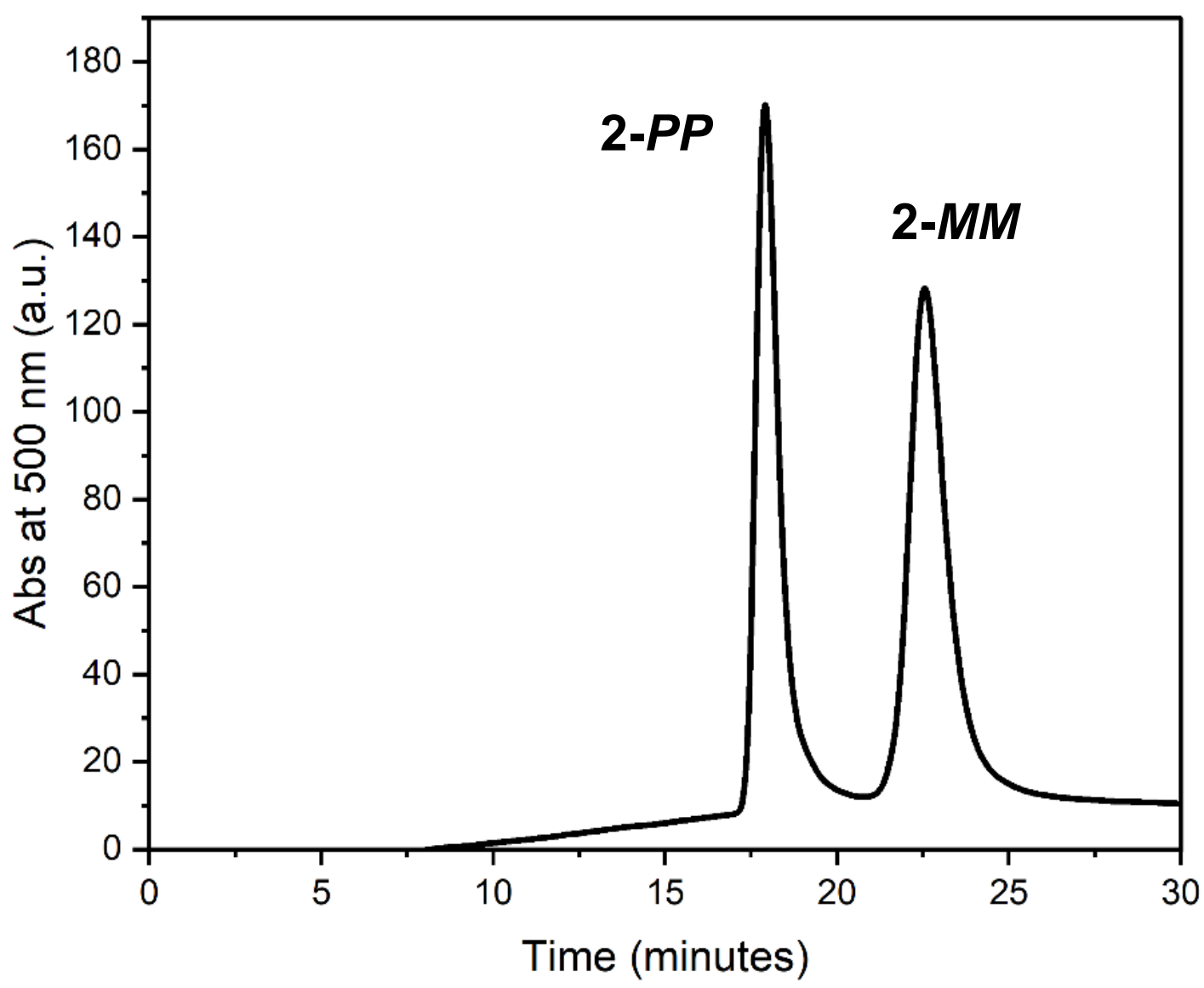

Figure S5.4: Chiral HPLC chromatogram (Phenomenex i-Amylose-1, 250 x 4.6 mm) of **2-*MM*/*PP*** dissolved in 1:1 DCM:n-hexane eluted with a gradient over 15 minutes of 38:0:62 to 58:2:40 of DCM:IPA:n-hexane.

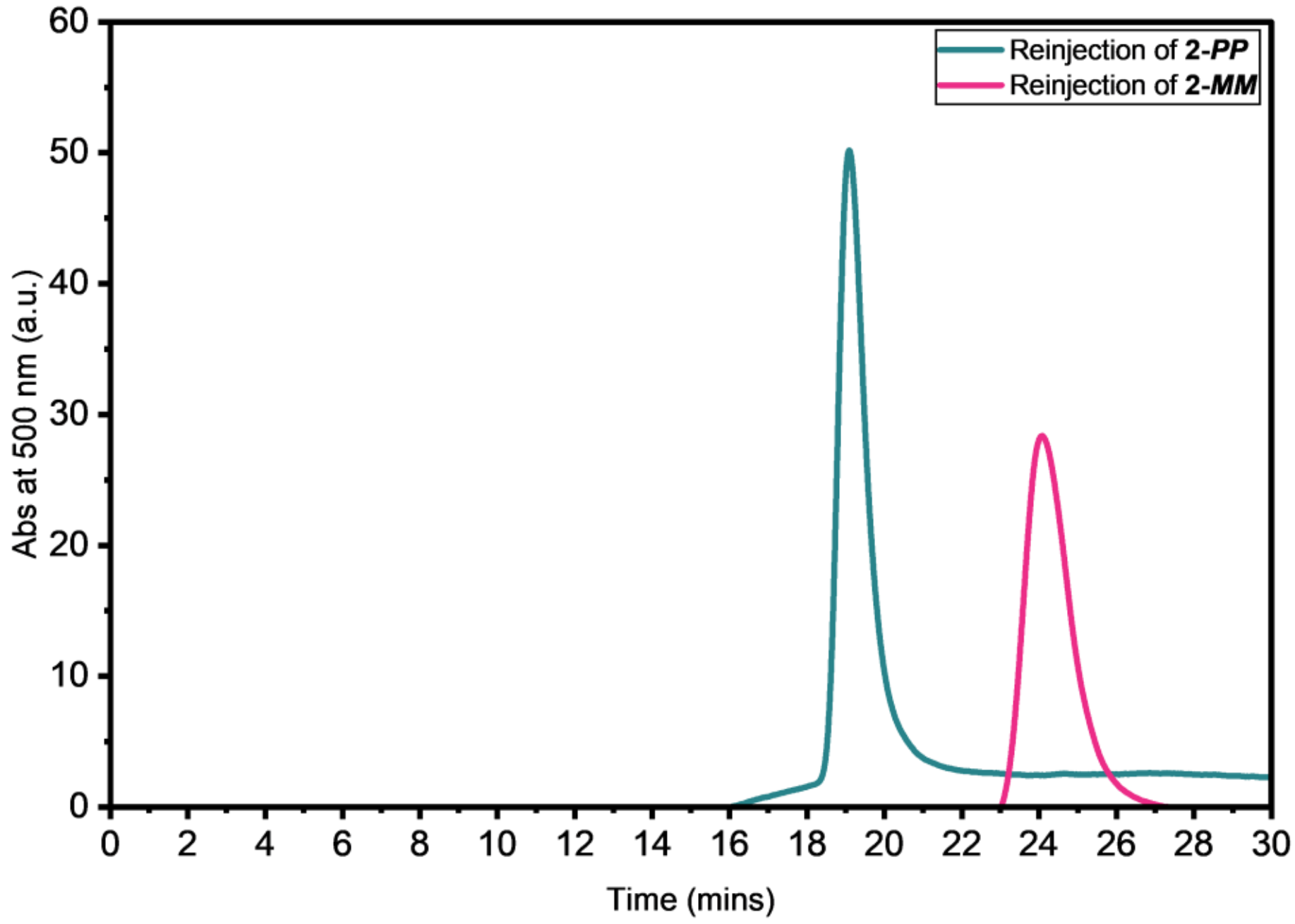

Figure S5.5: Chiral HPLC chromatogram (Phenomenex i-Amylose-1, 250 x 4.6 mm) of **2-*PP*** and **2-*MM*** dissolved in 1:1 DCM:n-hexane, previously purified by chiral HPLC. Enantiomers were left in solution (1:1 DCM:n-hexane) for one week at room temperature and then reinjected, revealing no racemisation.

6. Circular dichroism

To determine the racemisation rate constant (k_{rac}), an enantiopure sample of a macrocycle (e.g., **1-*PP***) was dissolved in 1,1,2,2-tetrachloroethane (TCE). The sample was placed in the CD spectrometer and kept at a constant temperature (112 °C = 385 K) and the CD spectrum was recorded at regular time intervals. The decay in intensity of the strongest CD band (325 nm) was monitored over time. The resulting data was fitted to the following equation:

$$\ln(CD_0/CD_t) = 2kt$$

Where CD_0 is the CD signal in intensity at t = 0 for the given peak (325 nm), CD_t is the CD signal intensity at time t for the given peak and k is the enantiomerisation rate constant. The racemisation rate constant, k_{rac}, is determined from $k_{rac} = 2k$.

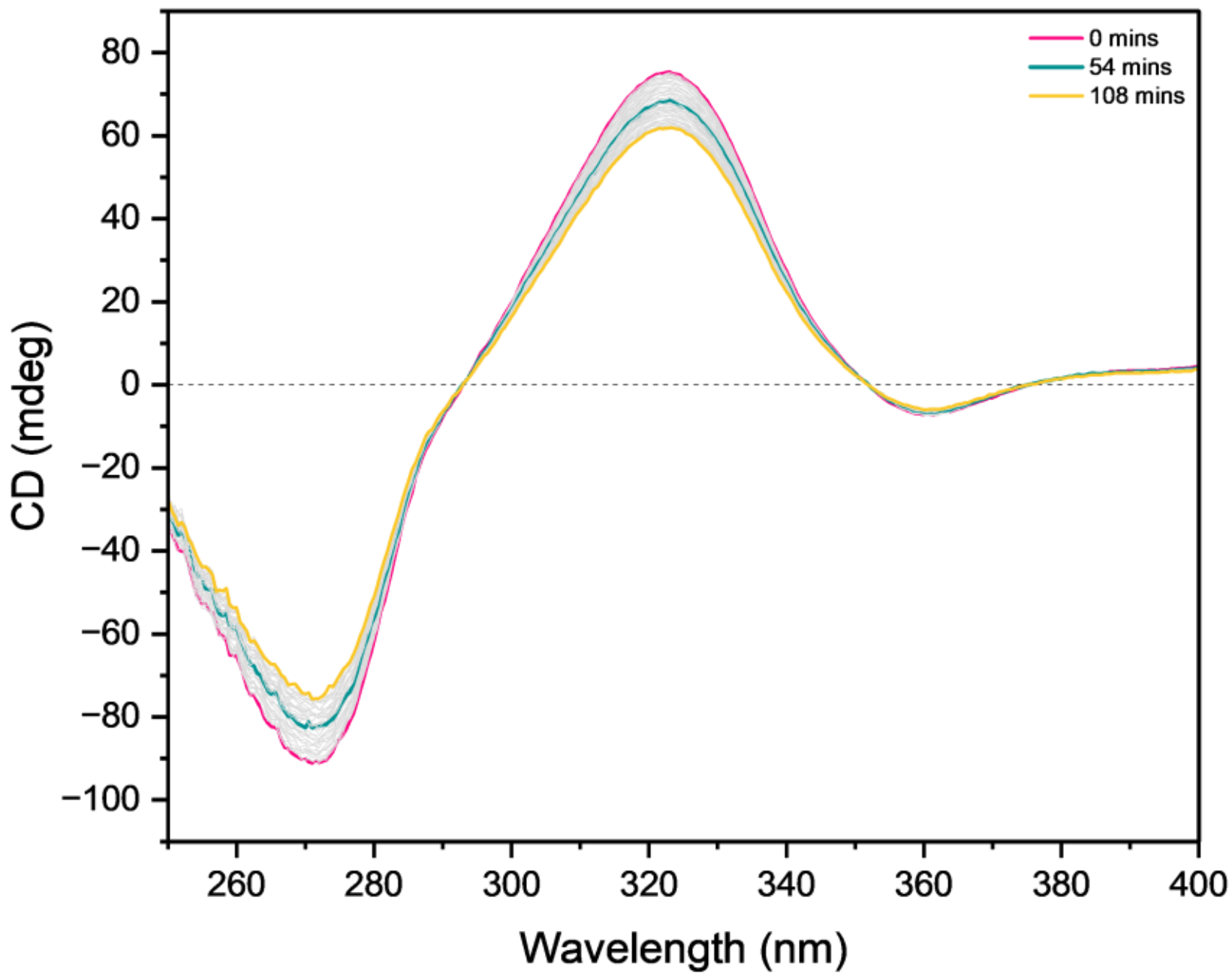

Figure S6.1: Time-course CD spectra for an enantiopure sample of **1-*PP*** in TCE at 385 K (12 μM).

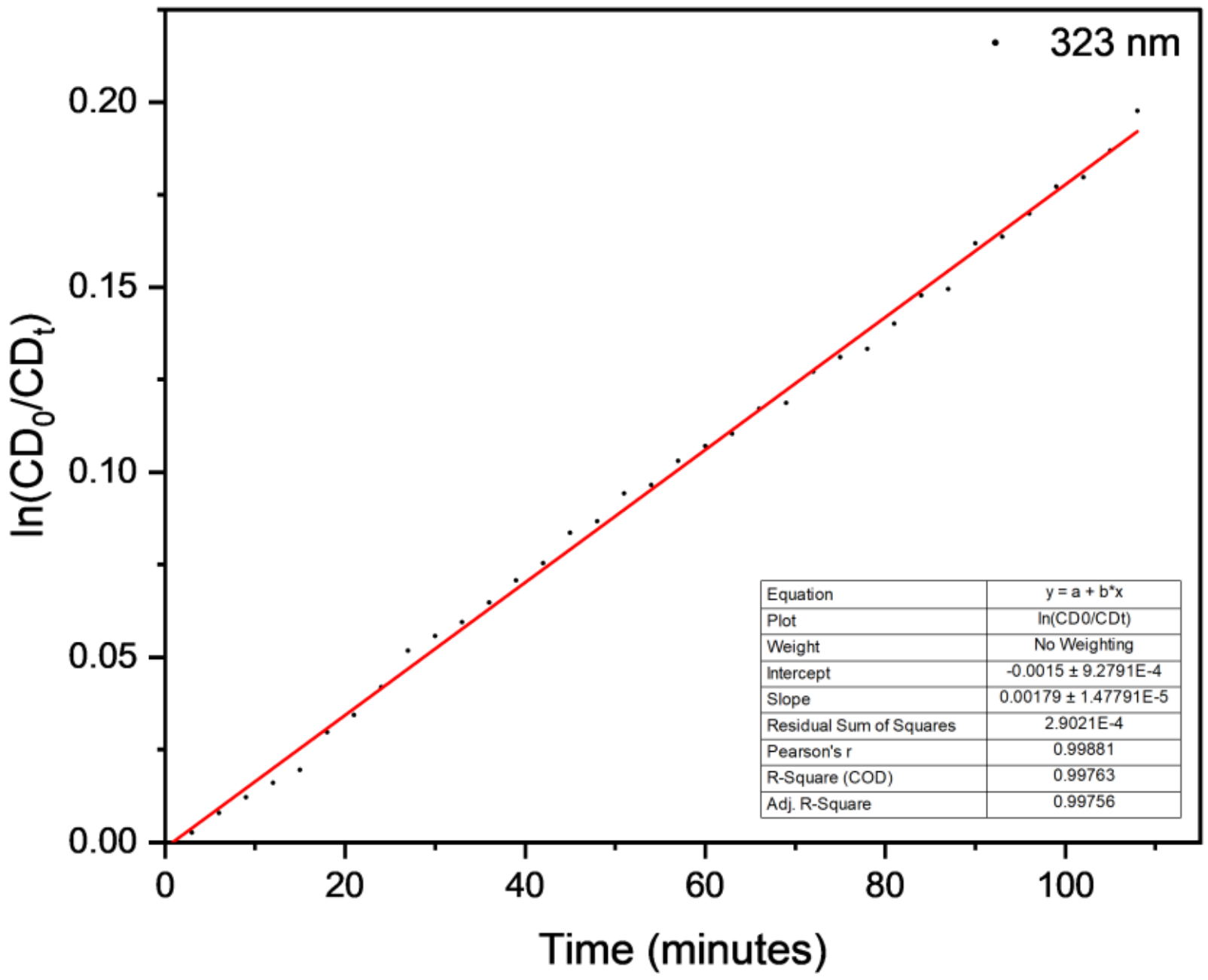

Figure S6.2: Plot and linear fit of ln(CD_0/CD_t) for **1-*PP***

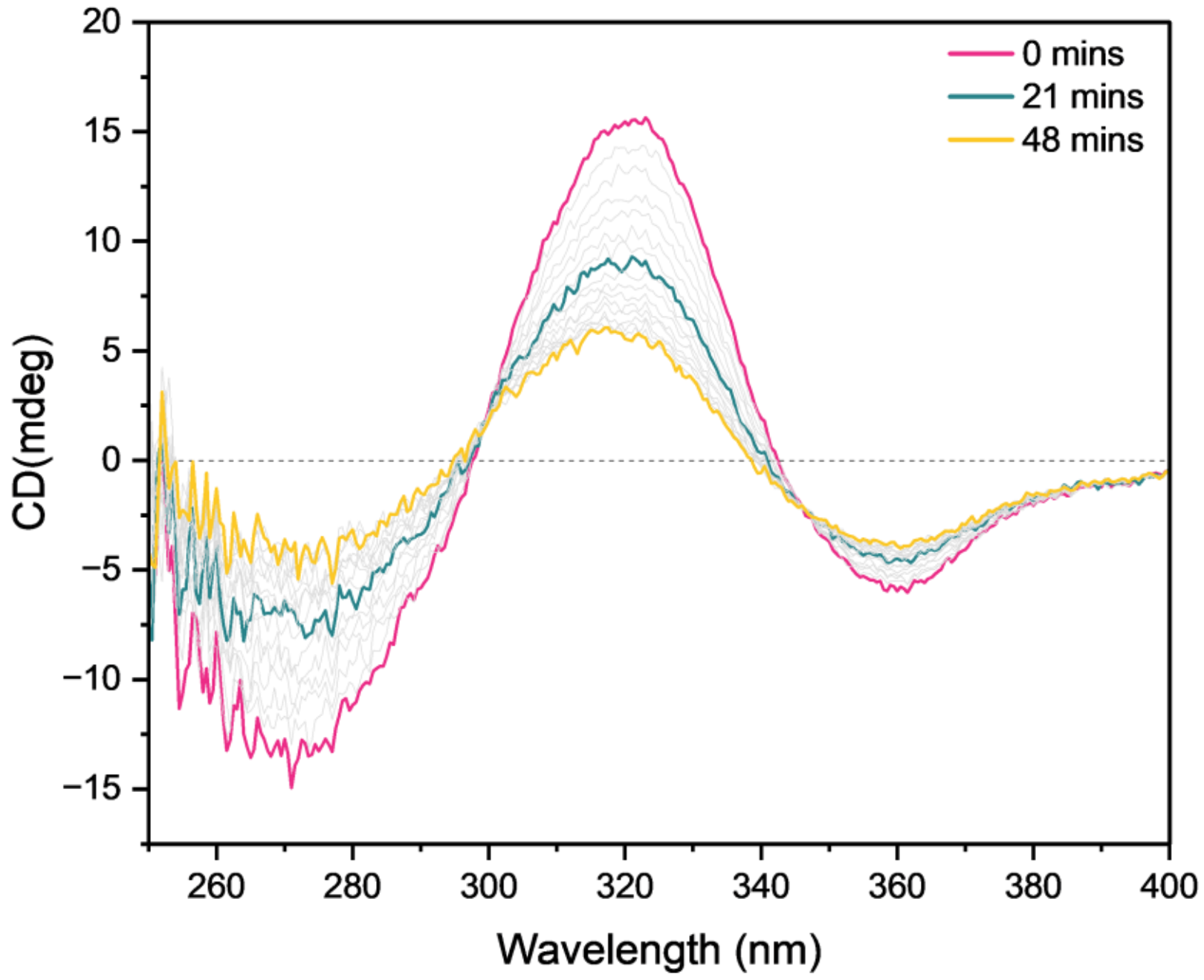

Figure S6.3: Time-course CD spectra for an enantiopure sample of **2-*PP*** in TCE at 385 K (2 μM).

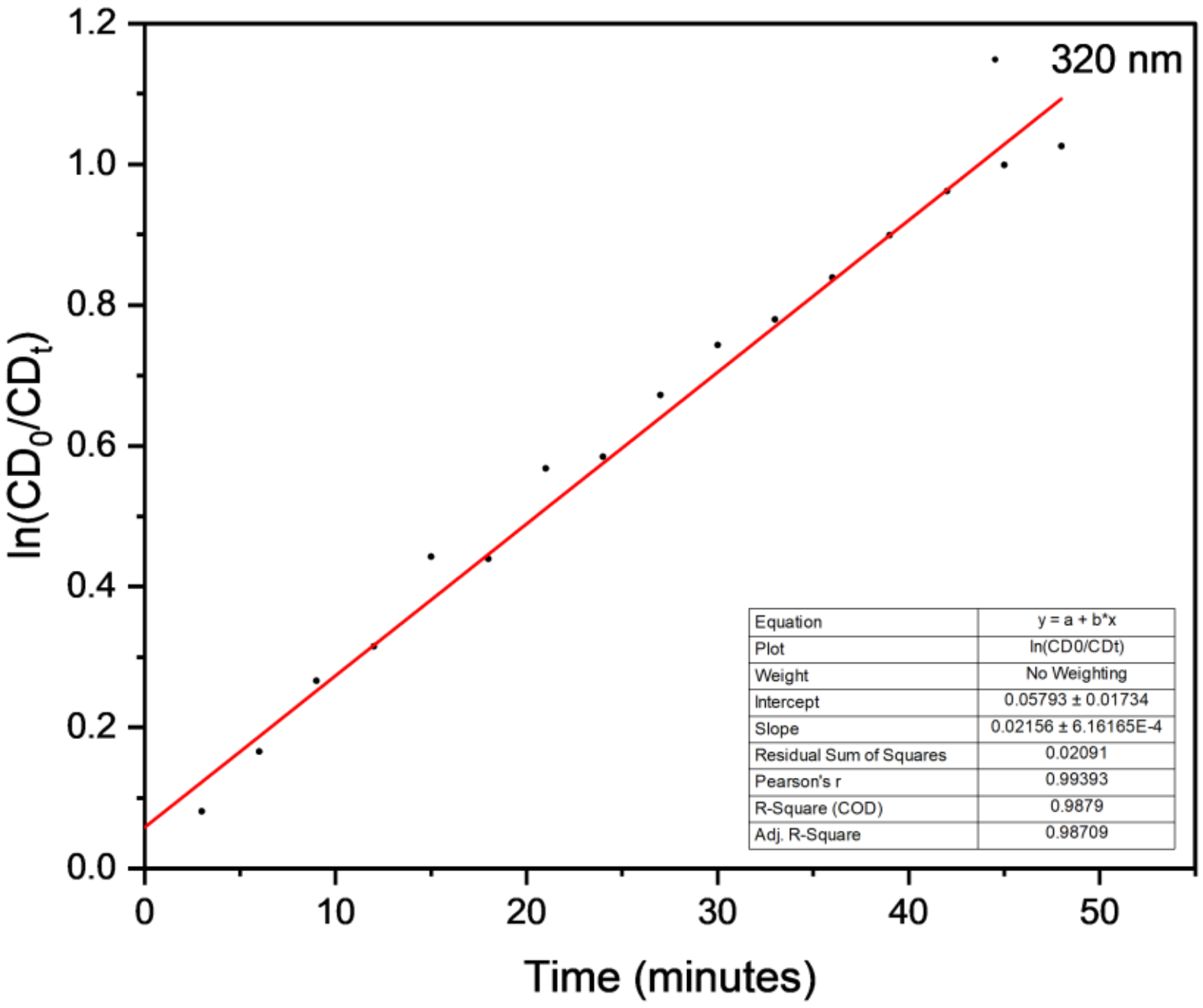

Figure S6.4*:* Plot and linear fit of $\ln(CD_0/CD_t)$ of **2-*PP*** in TCE at 385 K (2 μM).

Table S6.1: Kinetic and thermodynamic parameters for macrocycle stereoisomer interconversion in TCE at 385 K, as determined by time-course CD spectroscopy.

**Macrocycle**	k (s^{-1})	k_{rac} (s^{-1})	$t_{1/2}$ (hours)	$\Delta G^‡$ ($kJ\ mol^{-1}$)
**1**	1.5×10^{-5}	3.0×10^{-5}	6.5	131
**2**	1.8×10^{-4}	3.6×10^{-4}	0.5	122

Further CD spectroscopy studies were conducted on **1-*PP*** to obtain the enthalpic and entropic barrier to macrocycle stereoisomer interconversion. These were carried by time course CD measurements at five different temperatures using an enantiopure sample of **1-*PP*** in TCE. The enantiomerisation rate constant (k) is therefore obtained as a function of temperature (Table S6.2), which is then fitted (Fig. S6.5) to the linear form of the Van 't Hoff equation:

$$\ln\frac{k}{T} = \frac{-\Delta H^{\neq}}{R}\frac{1}{T} + ln\frac{k_B}{h} + \frac{\Delta S^{\neq}}{R}$$

where k_B is the Boltzmann constant, R is the gas constant and h is Planck's constant.

Table S6.2: Kinetic parameters for the interconversion of macrocycle **1**-***PP*** at different temperatures.

**Temp. (K)**	***k*** (s^{-1})
375	1.99×10^{-6}
383	1.41×10^{-5}
385	1.49×10^{-5}
388	2.49×10^{-5}
390	3.00×10^{-5}

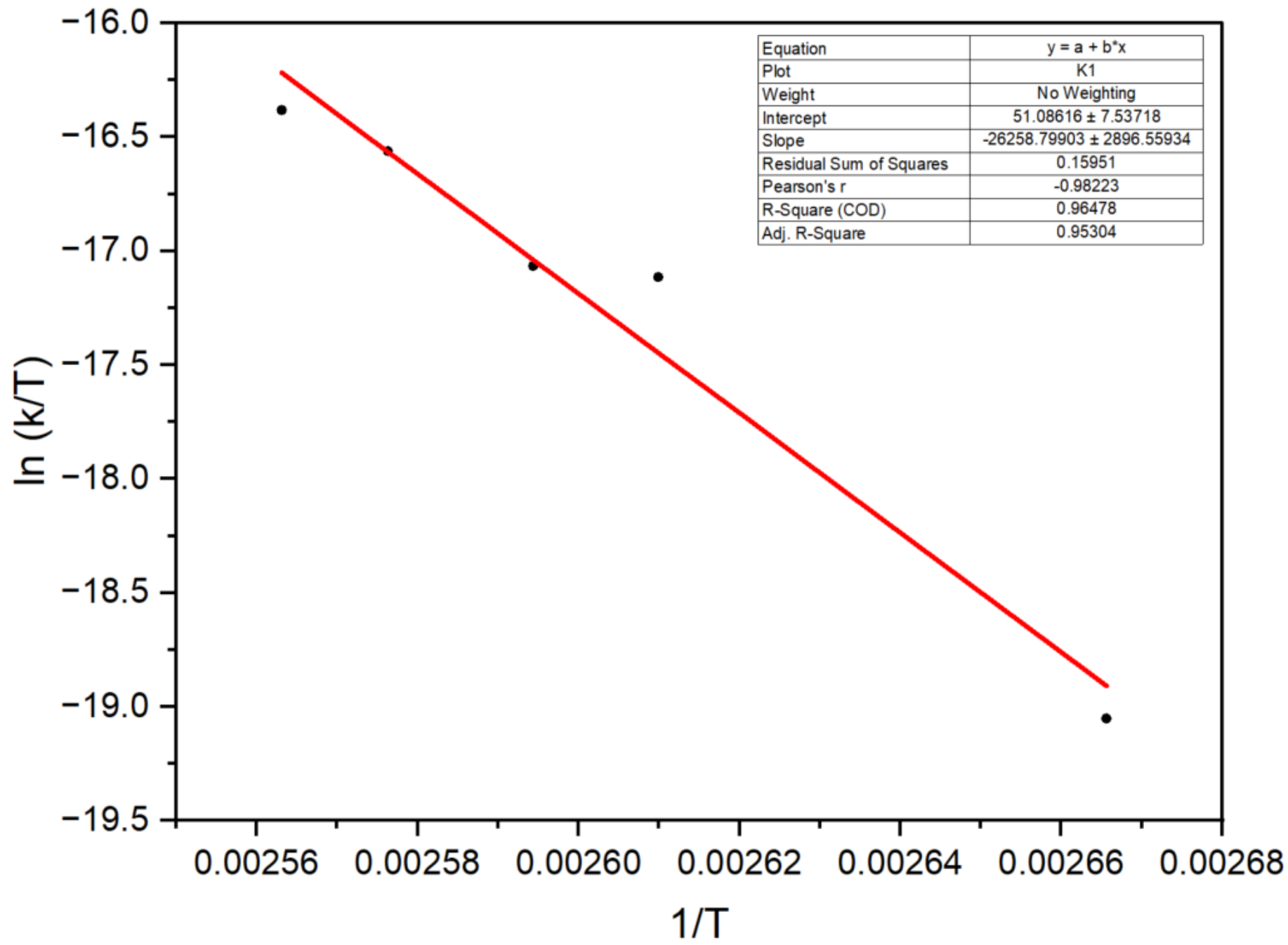

Figure S6.5*:* Van 't Hoff plot for the stereoisomer interconversion of macrocycle **1,** obtained from time course CD spectroscopy measurements at different temperatures. The fit of the data yields $\Delta H^{\ddagger}$ = 218 kJ mol^{-1} and $\Delta S^{\ddagger}$ = 227J K^{-1} mol^{-1}.

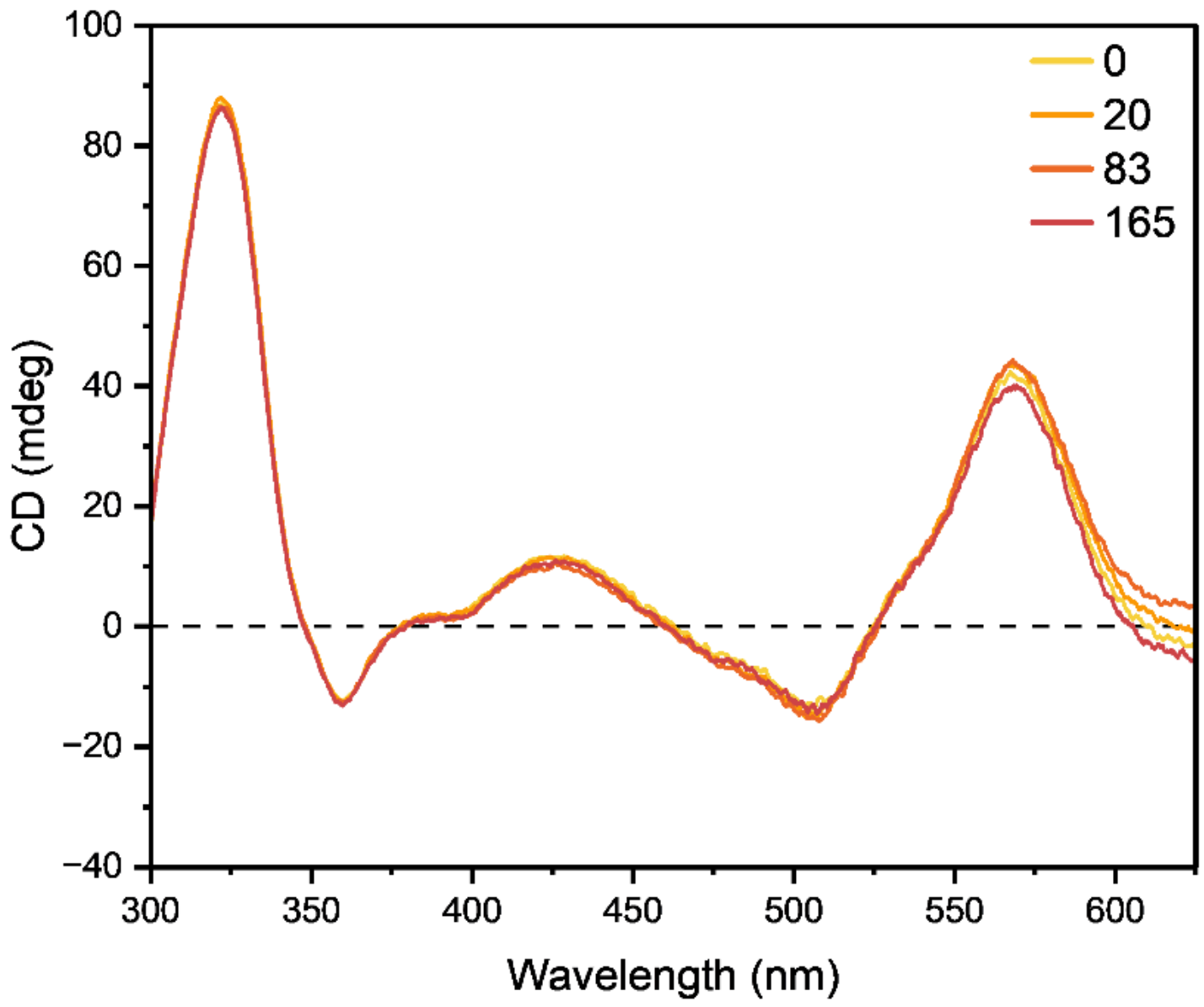

Figure S6.6: CD spectra of **1-*PP*** in TCE measured at day 0, day 20, day 83 and day 165. The sample was kept in solution in a sealed cuvette at room temperature (10 µM).

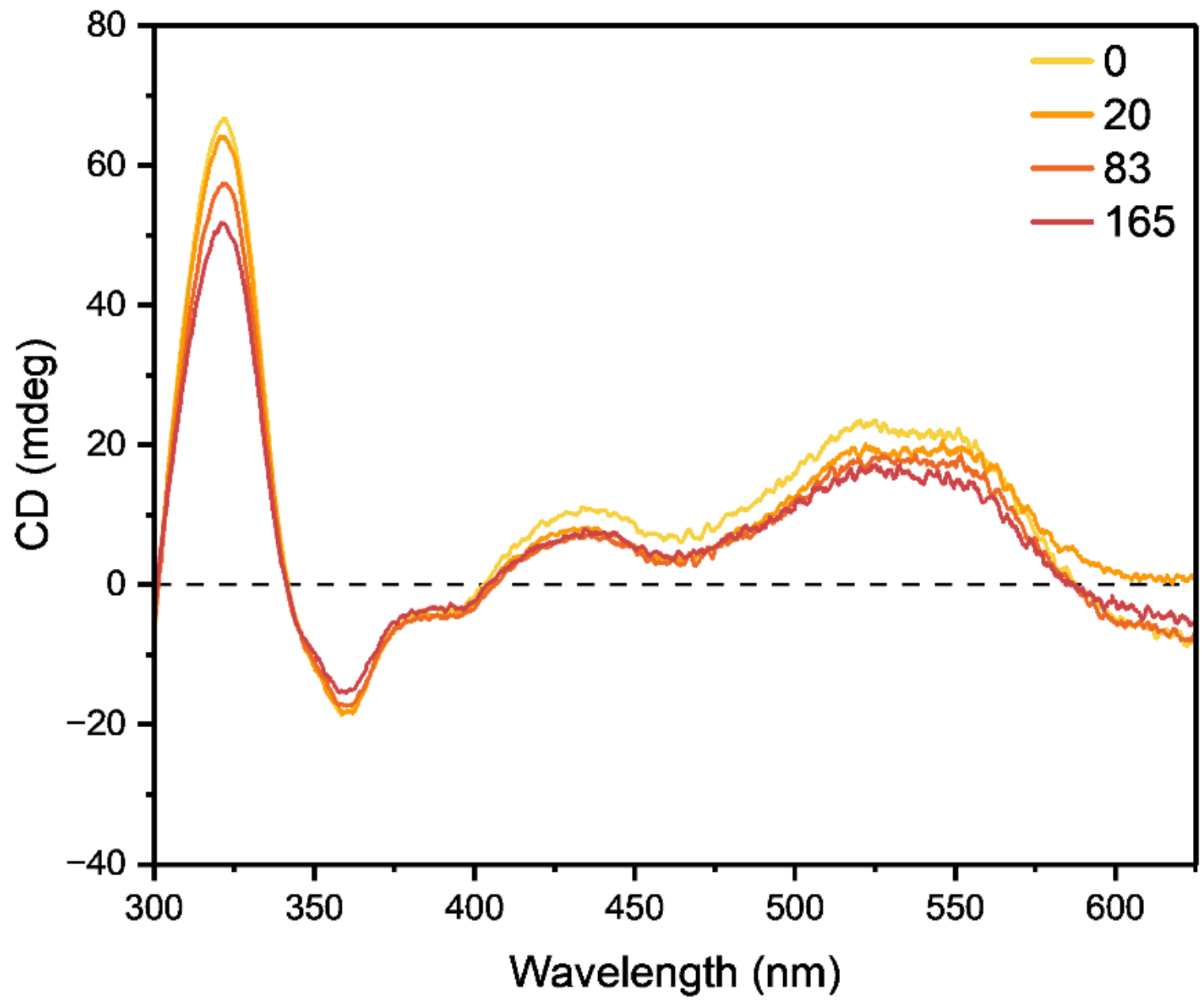

Figure S6.7: CD spectra of ***2-PP*** in TCE measured at day 0, day 20, day 83 and day 165. The sample was kept in solution in a sealed cuvette at room temperature (10 µM).

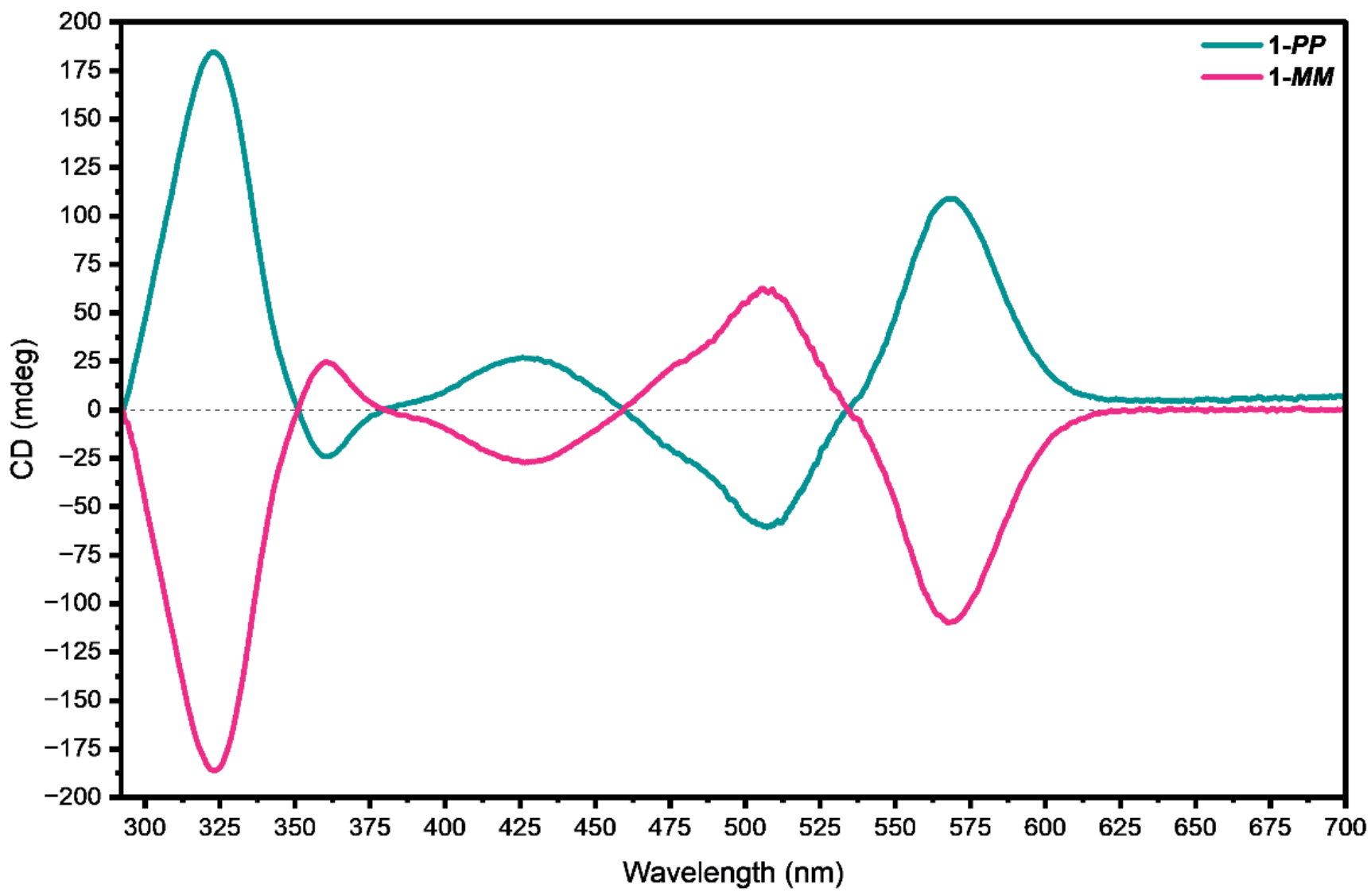

Figure S6.8: CD spectra for **1-*MM*** and **1-*PP*** enantiomers in toluene (25 μM).

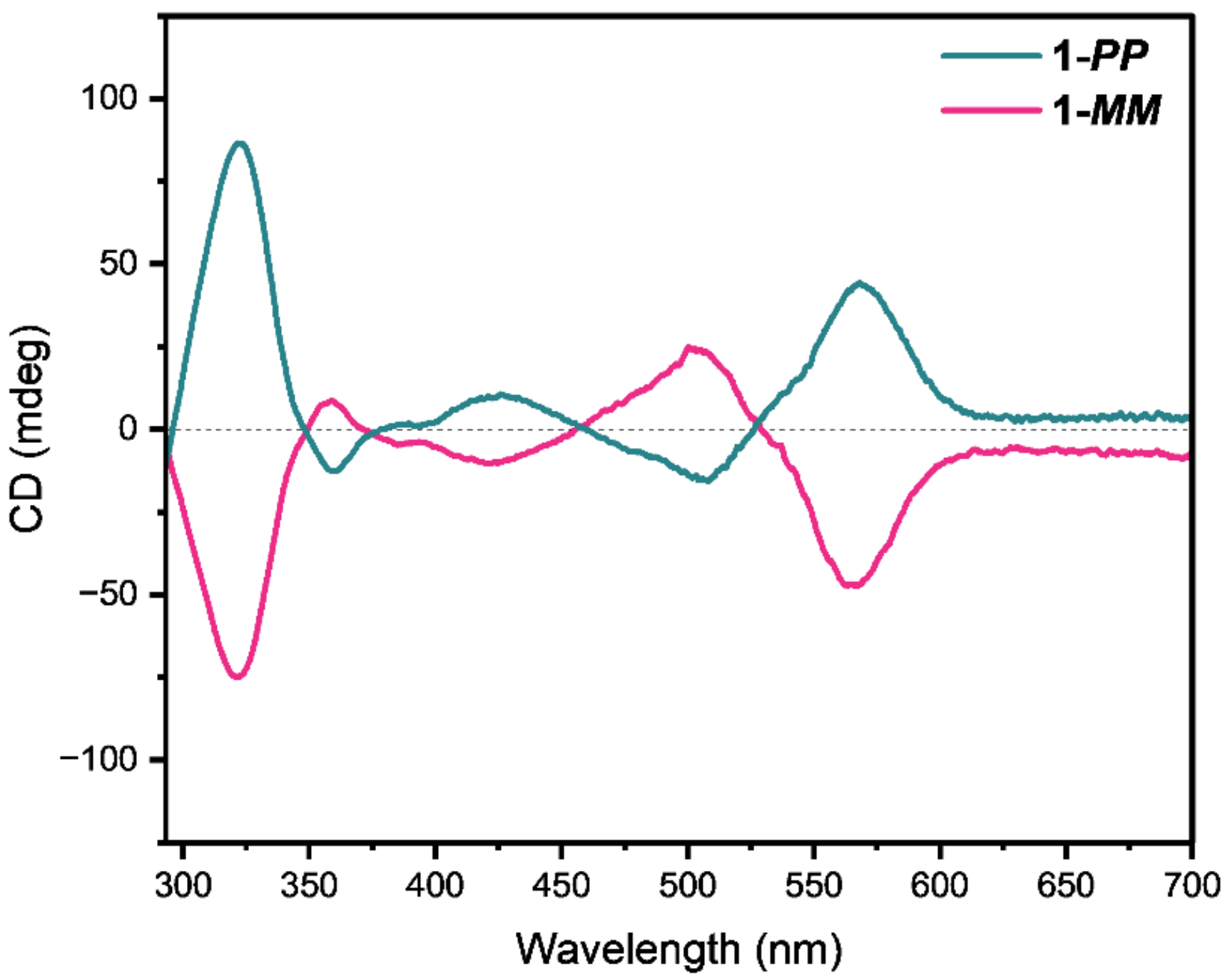

Figure S6.9: CD spectra for **1-*MM*** and **1-*PP*** enantiomers in TCE (12 μM).

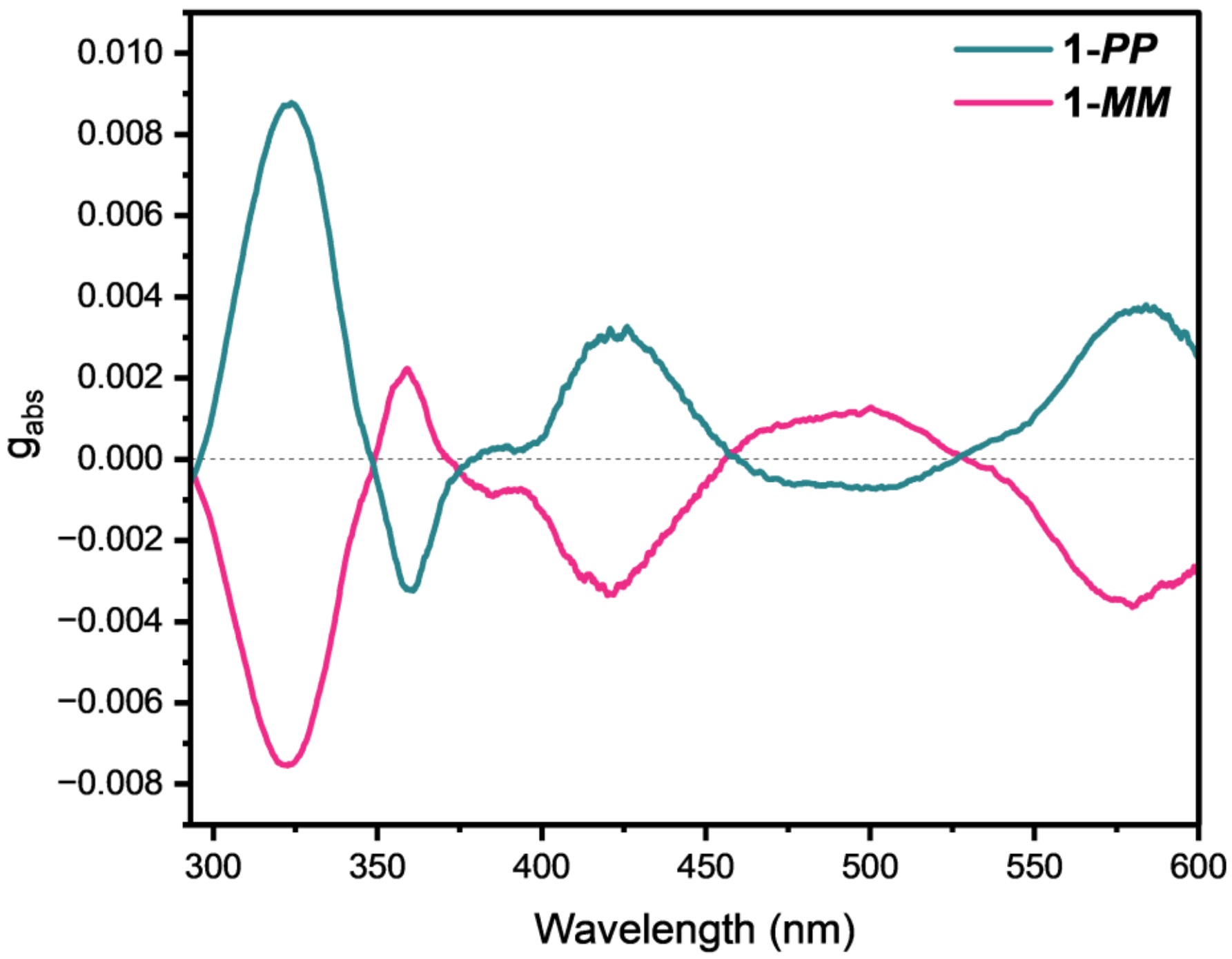

Figure S6.10: CD spectra for **1-*MM*** and **1-*PP*** enantiomers in TCE showing the variation in g_{abs}

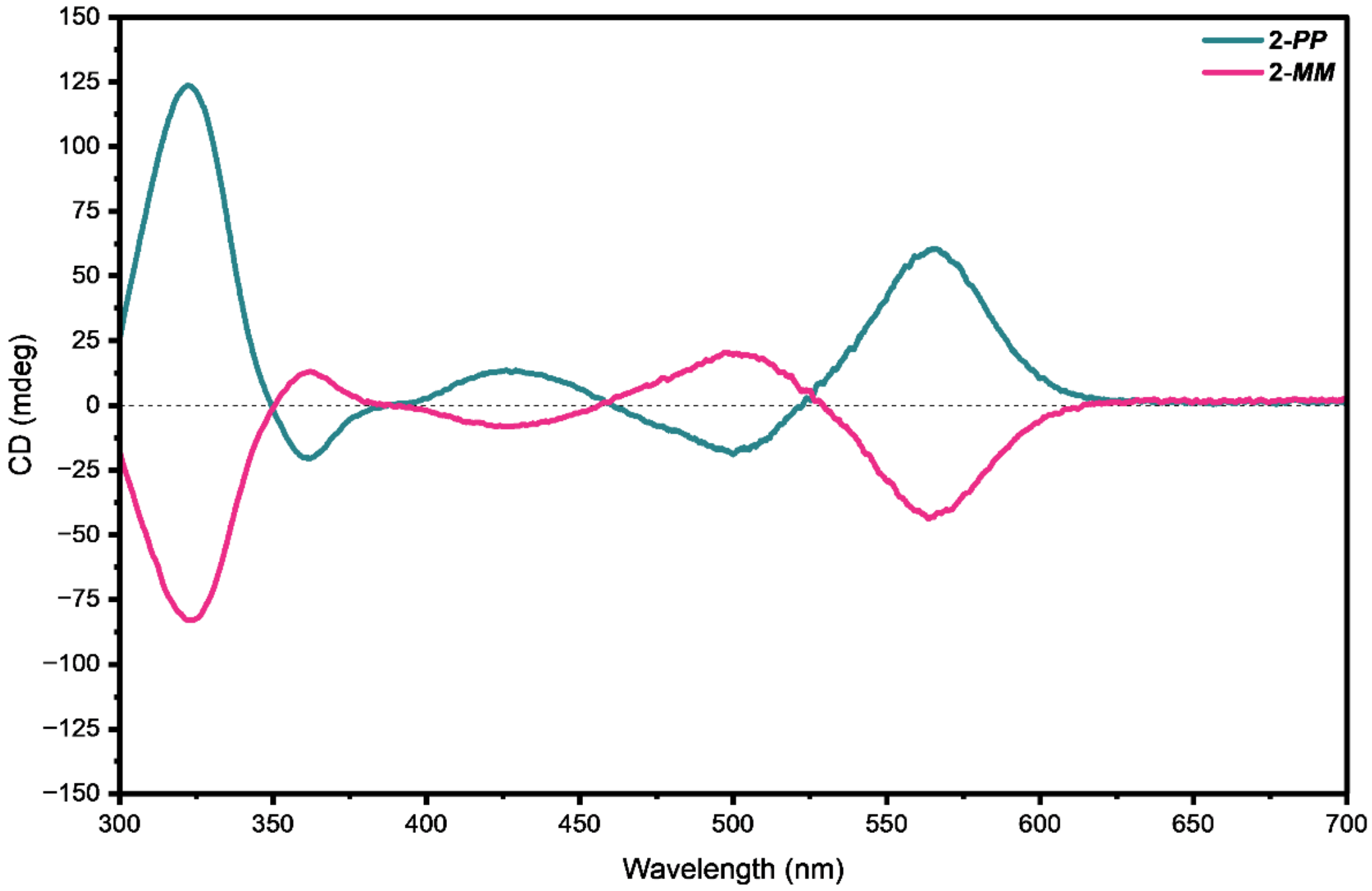

Figure S6.11: CD spectra for **2-*MM*** and **2-*PP*** enantiomers in toluene (18 µM).

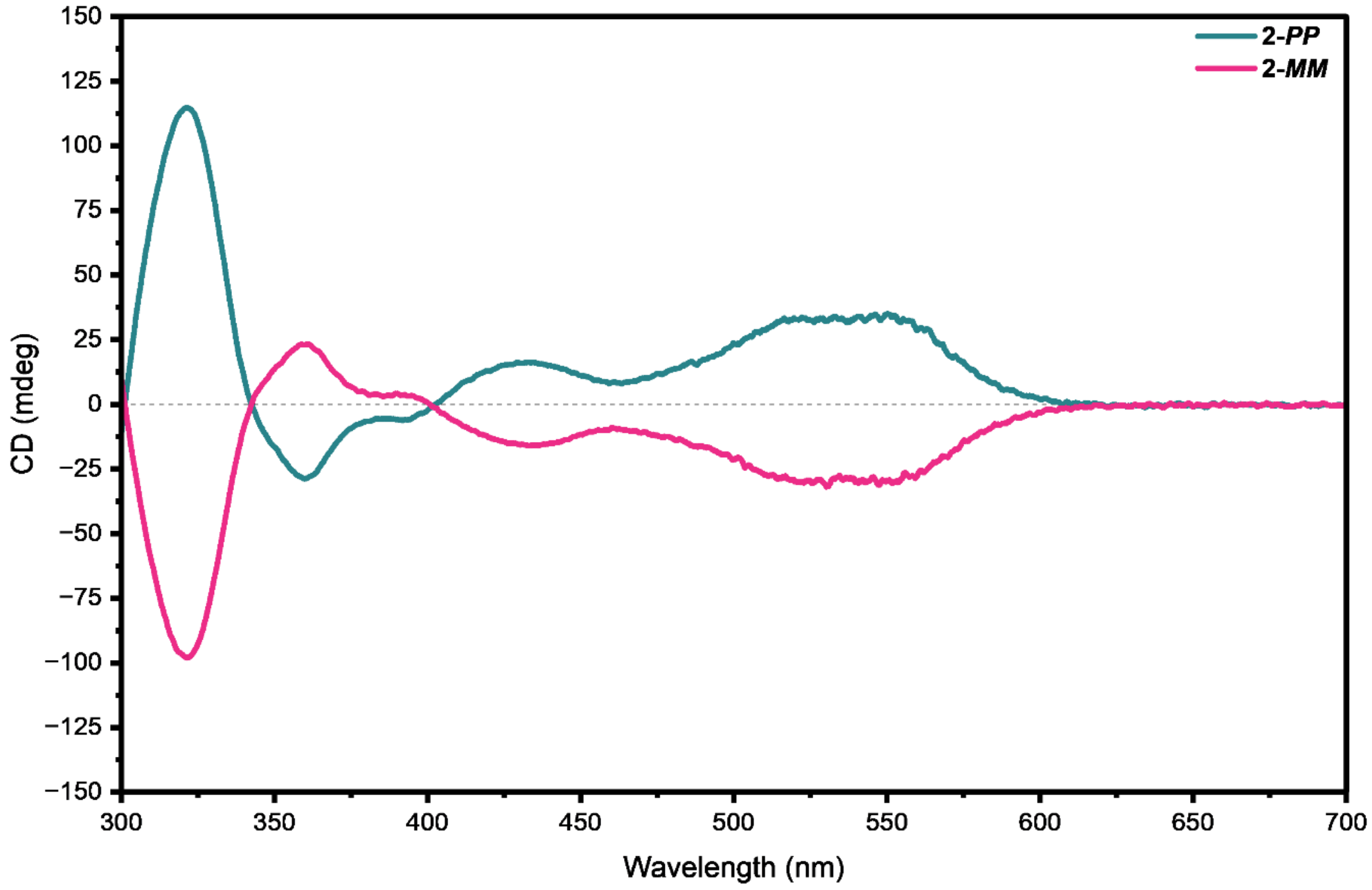

Figure S6.12*:* CD spectra for **2-*MM*** and **2-*PP*** enantiomers in TCE (17 µM).

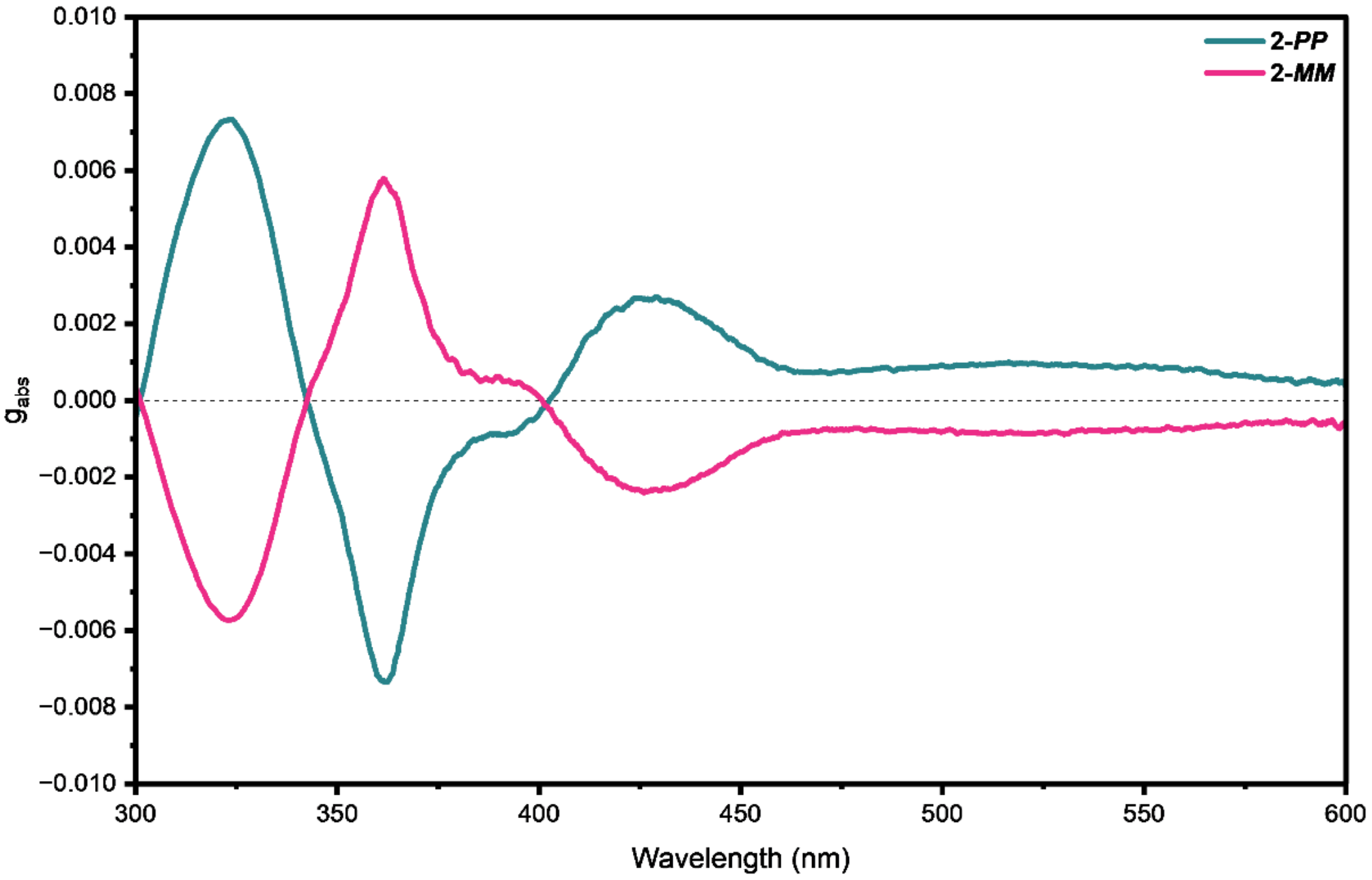

Figure S6.13: CD spectra for **2-*MM*** and **2-*PP*** enantiomers in TCE showing the variation in g_{abs}

7. Mueller–matrix polarimetry

For anisotropic samples such as thin films, linear dichroism (LD) and linear birefringence (LB) can substantially affect the spectral shape, magnitude, intensity and sign of the CD measured with benchtop and synchrotron CD instruments. Also, the residual static strain birefringence of the photoelastic modulator and polarizing prism can generate CD artifacts. For accurate chiroptical characterisation of solid-state chiral materials Muller Matrix Polarimetry (MMP) has to be employed, as each optical property can be decomposed from the others.[14,15]

MMP was performed at B23 at Diamond Light Source. Here, the custom-built MMP by Hinds Instruments is composed of four photoelastic modulators in transmission operating at different frequencies, two in the polarization state generator and two in the polarization state analyser[16,17] coupled to a double grating subtractive monochromator (Olis Instruments).[18] The wavelength range of B23 MMP is 190-700nm, which has been successfully employed to measure and quantify the CD of a wide range of chiral materials in the solid-state.[19–24] With benchtop CD instruments, the smallest area that can be measured is about 3 mm in diameter giving an average CD compared to 50 μm using the MMP instrument at B23.[16]

An important measurement derived from MMP is the depolarization index (DI) [25] that varies from 1 for non-depolarising materials to 0 for depolarising materials. For non-depolarizing materials, the natural logarithm Matrix[26] method is capable of decomposing the polarization elements CD, LD, LD', CB, LB and LB'. For materials with a depolarization index between 1 and 0.5, the Analytic Inversion method[27] can decompose the polarization properties.

The Analytic Inversion is the method used in this study, enabling the CD to be decomposed from the other optical polarizations. Based on the type of materials investigated, some areas or spectral regions may show DI > 1 (due to e.g., light scattering, iridescence and reflections), in which case our interpretation of the data is only carried out in the areas of spectral regions with $0.5 \leq DI \leq 1$, since only these will have a physical meaning. A $DI < 0.5$ is indicative of complex materials for which none of the current methods can decompose their optical properties.

The 16 elements of the 4 × 4 Mueller Matrix (spectroscopic and mapping data) are presented for each sample. All datasets were processed with the Analytic Inversion method (for DI between 0.5 and 1) calculating the CD, CB, LD, LD', LB, and LB' optical properties and presented together with the absorbance (Abs), the dissymmetry factor ($g_{abs} = \Delta Abs/Abs$), and the Depolarization Index (DI) to allow for comparison between different samples. The substrate background has been subtracted for all MMP data sets.

MMP spectra were recorded with over a wavelength range from 250 to 700 nm. For each sample, three spectra at different locations were taken and averaged to produce representative spectra of the whole sample. MMP mapping was performed at λ = 576/590 nm, chosen as this is a CD maximum for the mirror image enantiomer films prior/after annealing. Spectra and maps were taken with an integration time of 0.1 seconds over 16 iterations.

a) Pre-annealed films

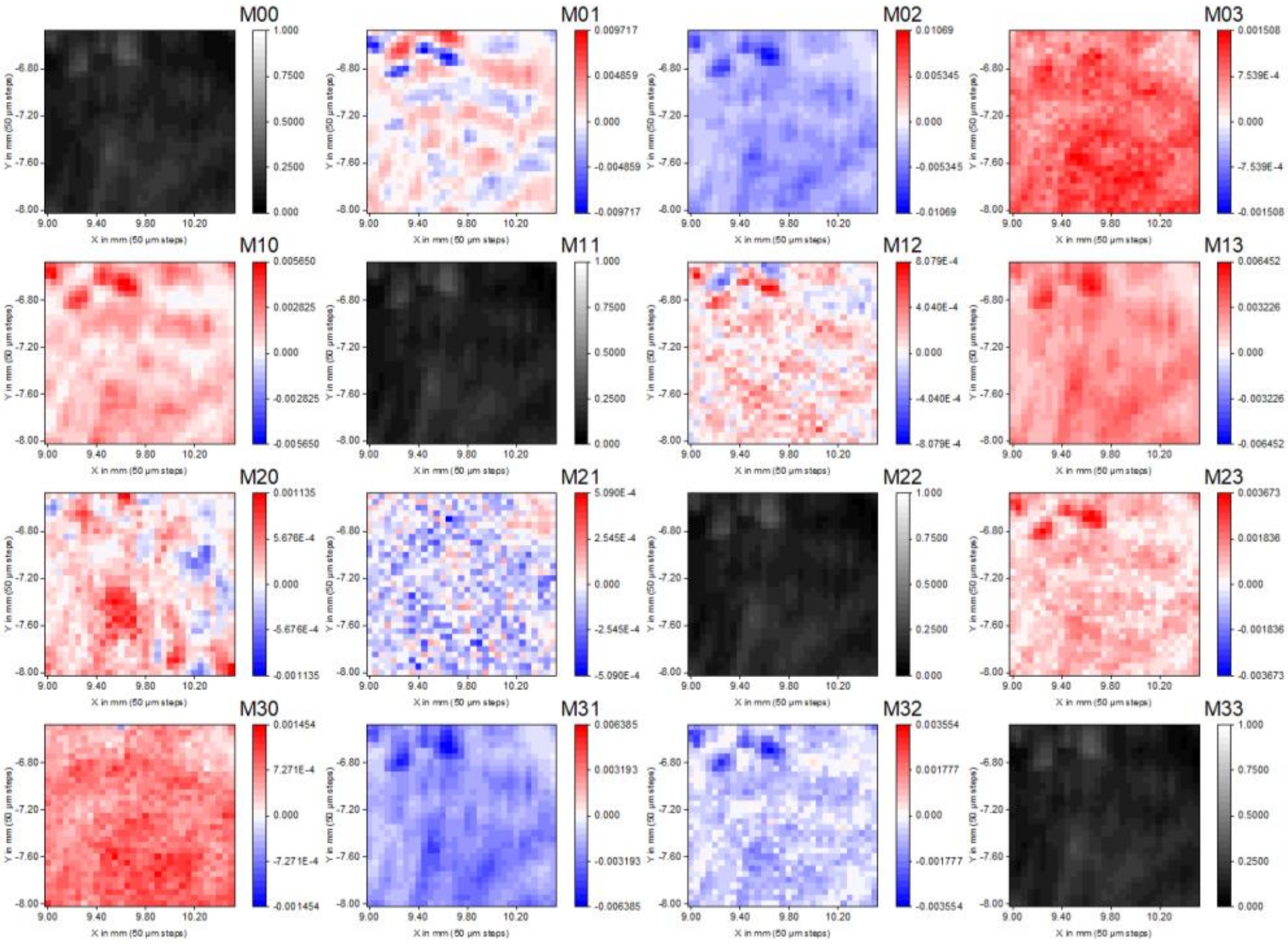

Figure S7.1: Raw data of differential 4×4 Mueller-Matrix of **1**-***PP*** macrocycle thin film.

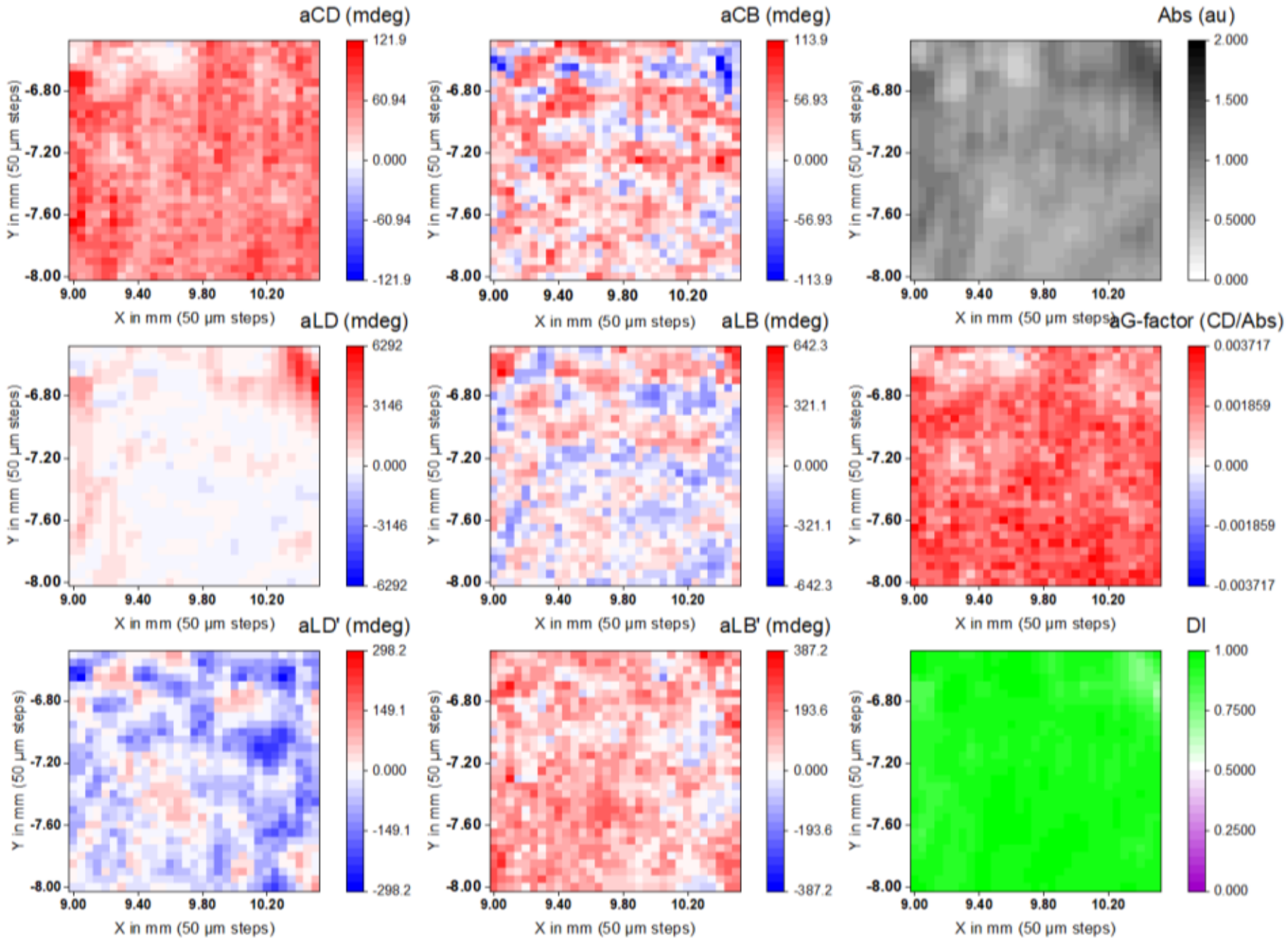

Figure S7.2: MMP map of **1**-***PP*** macrocycle thin film at 31 x 31 steps of 50 μM recorded at 576 nm, baseline corrected for the glass substrate and processed using the Analytic Inversion method.

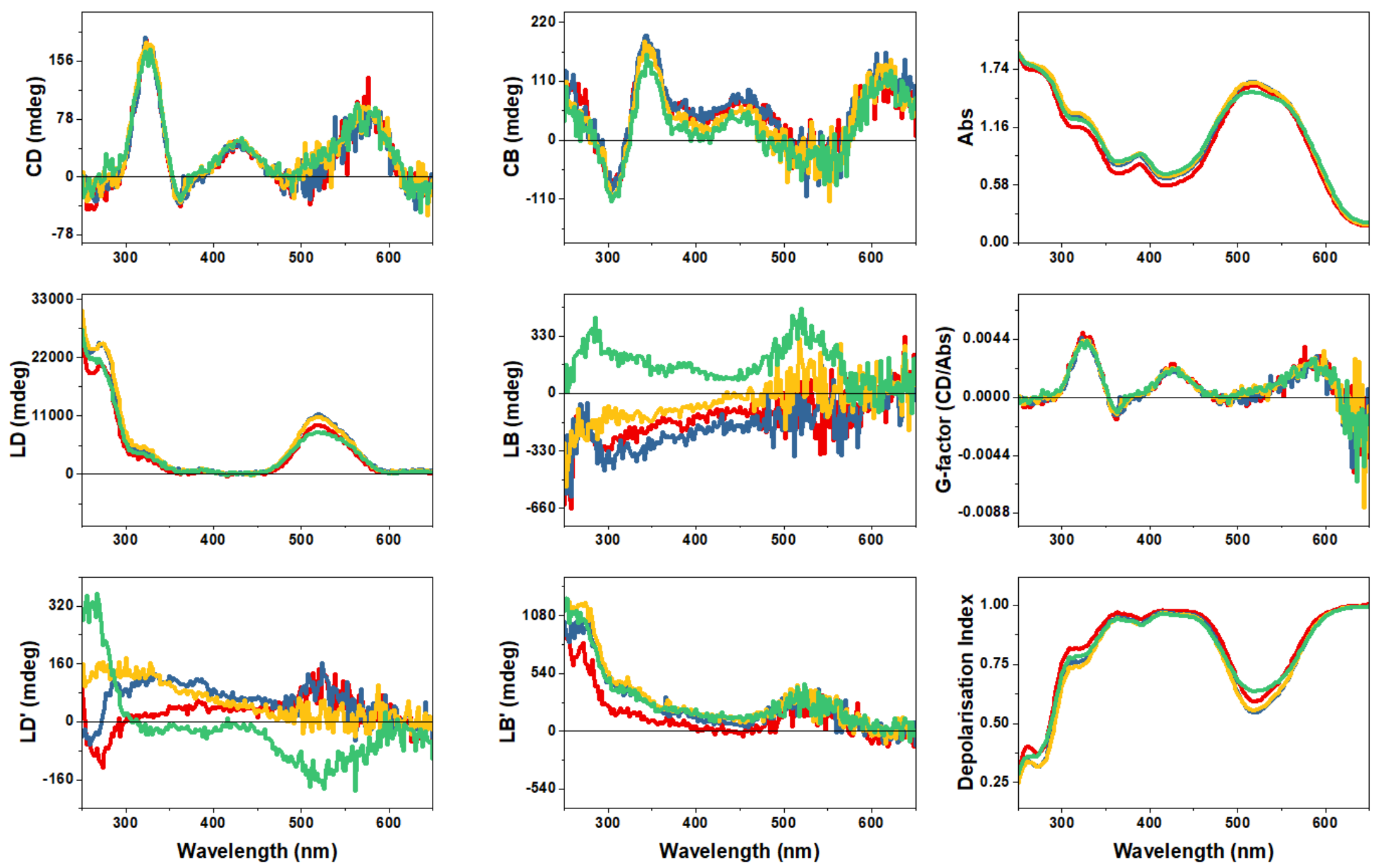

Figure S7.3: CD, CB, Abs, LD, LB, g_{abs}, LD', LB' and DI spectra of **1-*PP*** macrocycle thin film recorded at four different positions on the film processed with Analytical Inversion method. The g_{abs} at λ > 600 nm is neglected due to noise.

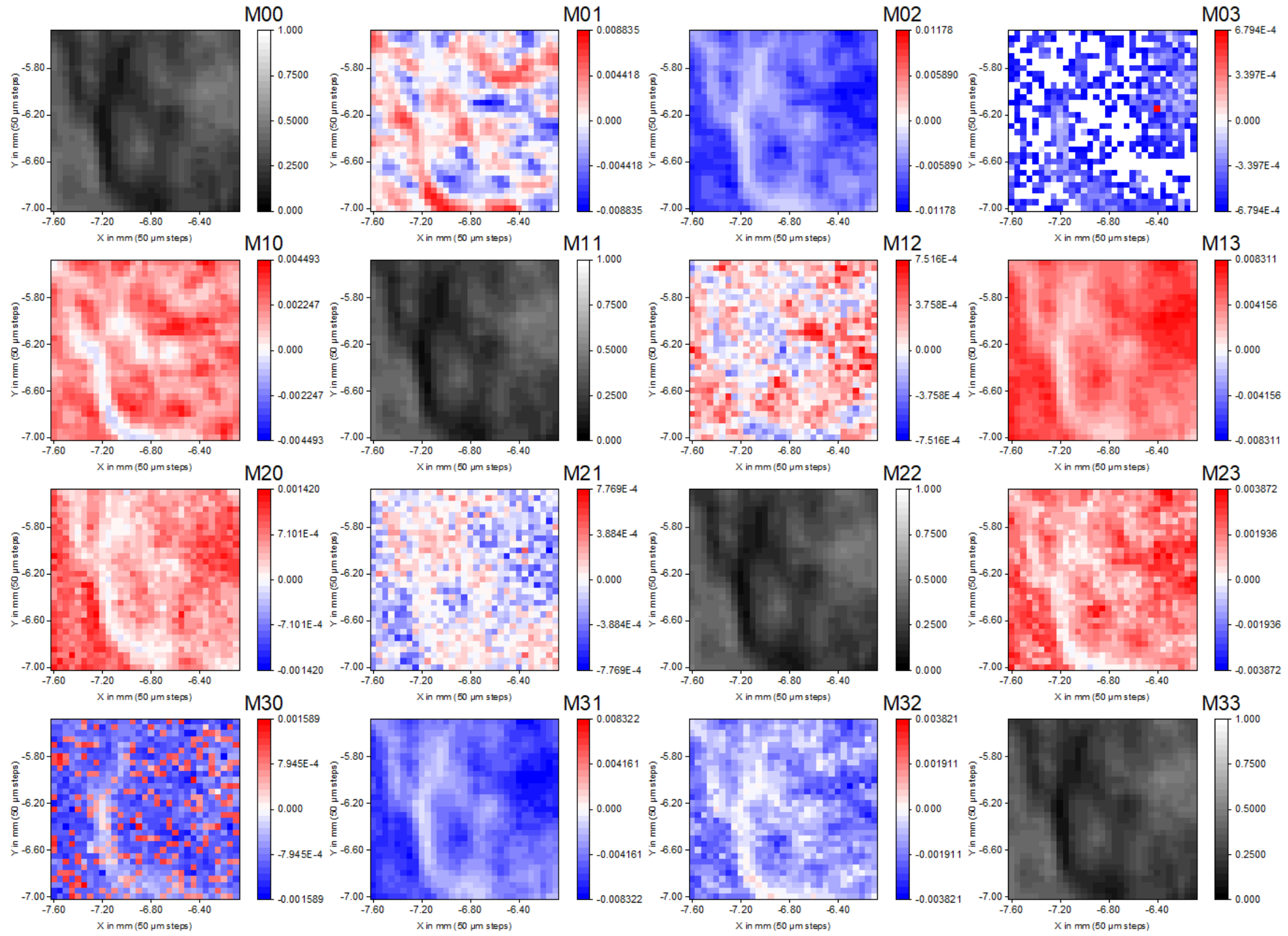

Figure S7.4: Raw data of differential 4×4 Mueller-Matrix of **1-*MM*** macrocycle thin film.

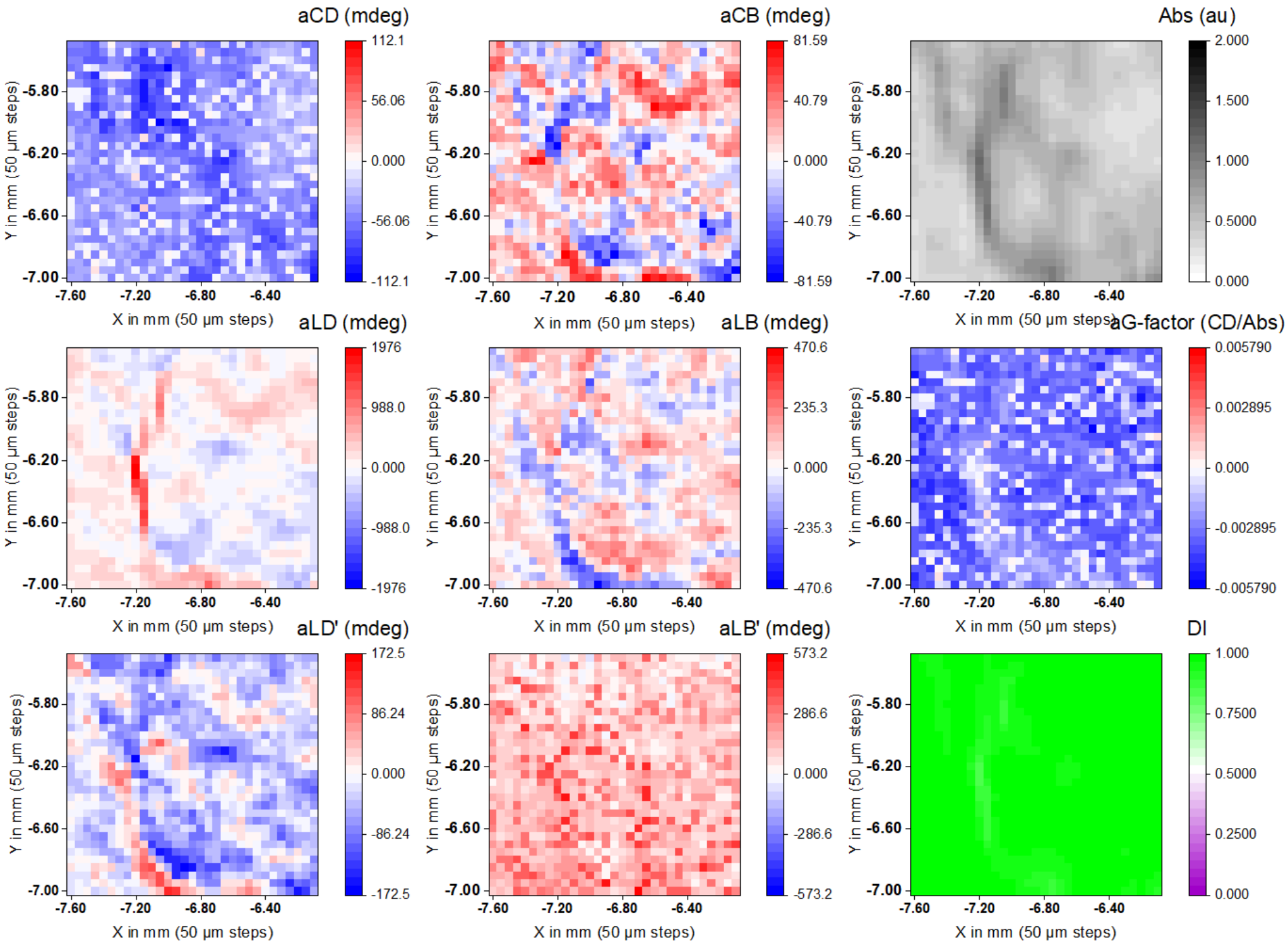

Figure S7.5: MMP map of **1**-***MM*** macrocycle thin film at 31 x 31 steps of 50 μM recorded at 576 nm, baseline corrected for the glass substrate and processed using the Analytic Inversion method.

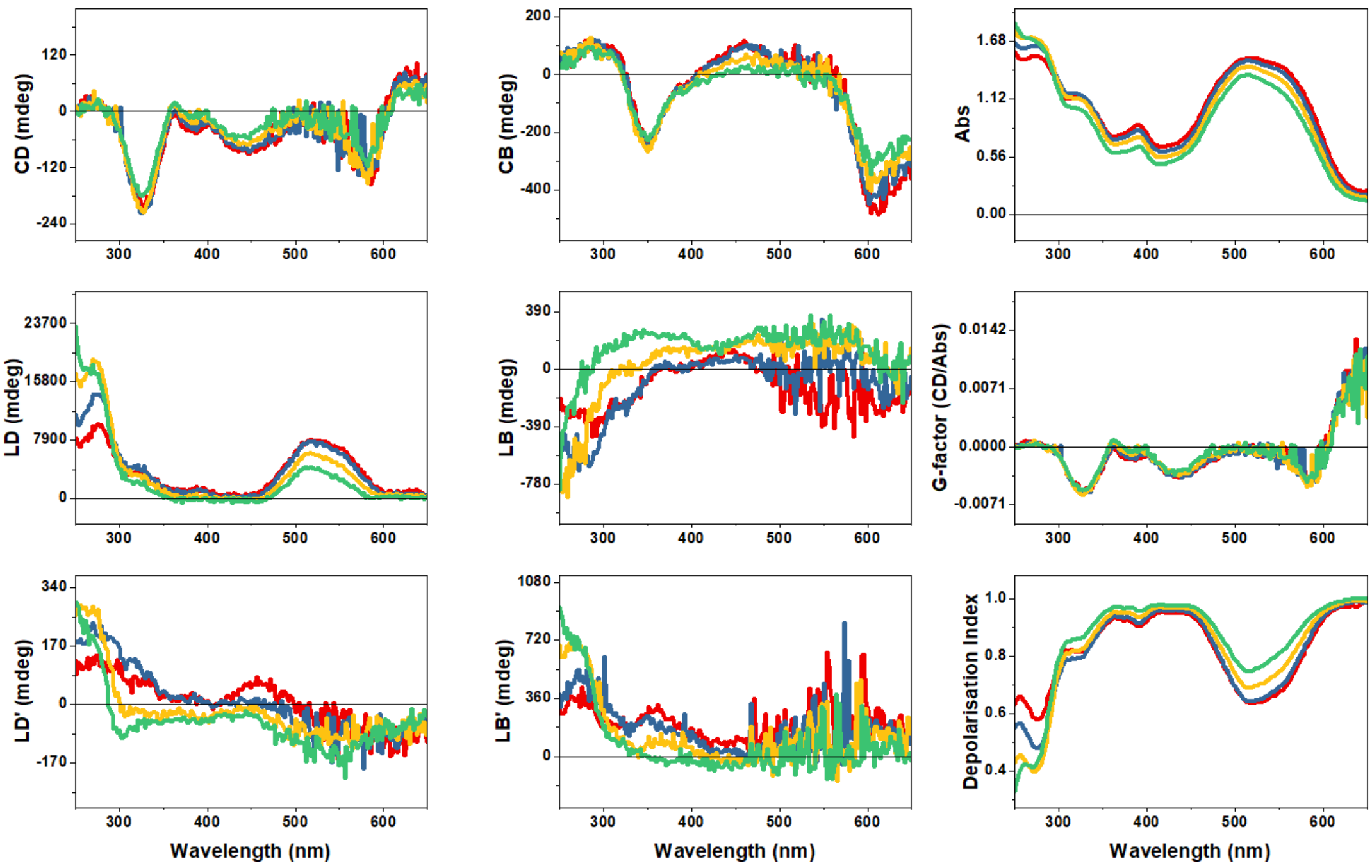

Figure S7.6: CD, CB, Abs, LD, LB, g_{abs}, LD', LB' and DI spectra of **1**-***MM*** macrocycle thin film recorded at four different positions on the film processed with Analytical Inversion method. The g_{abs} at $\lambda > 600$ nm is neglected due to noise.

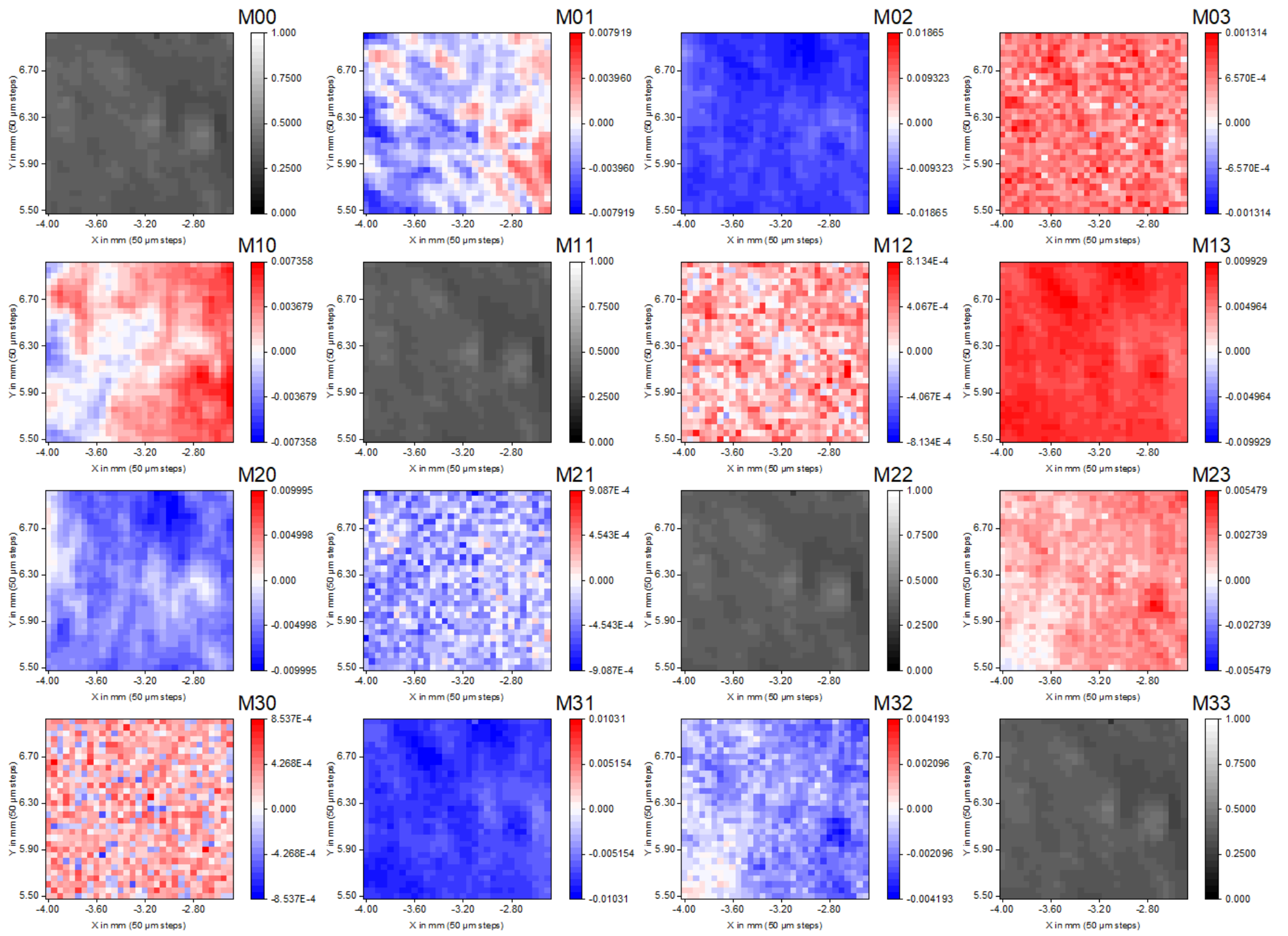

Figure S7.7: Raw data of differential 4×4 Mueller-Matrix of racemic **1-*MM*/*PP*** macrocycle thin film.

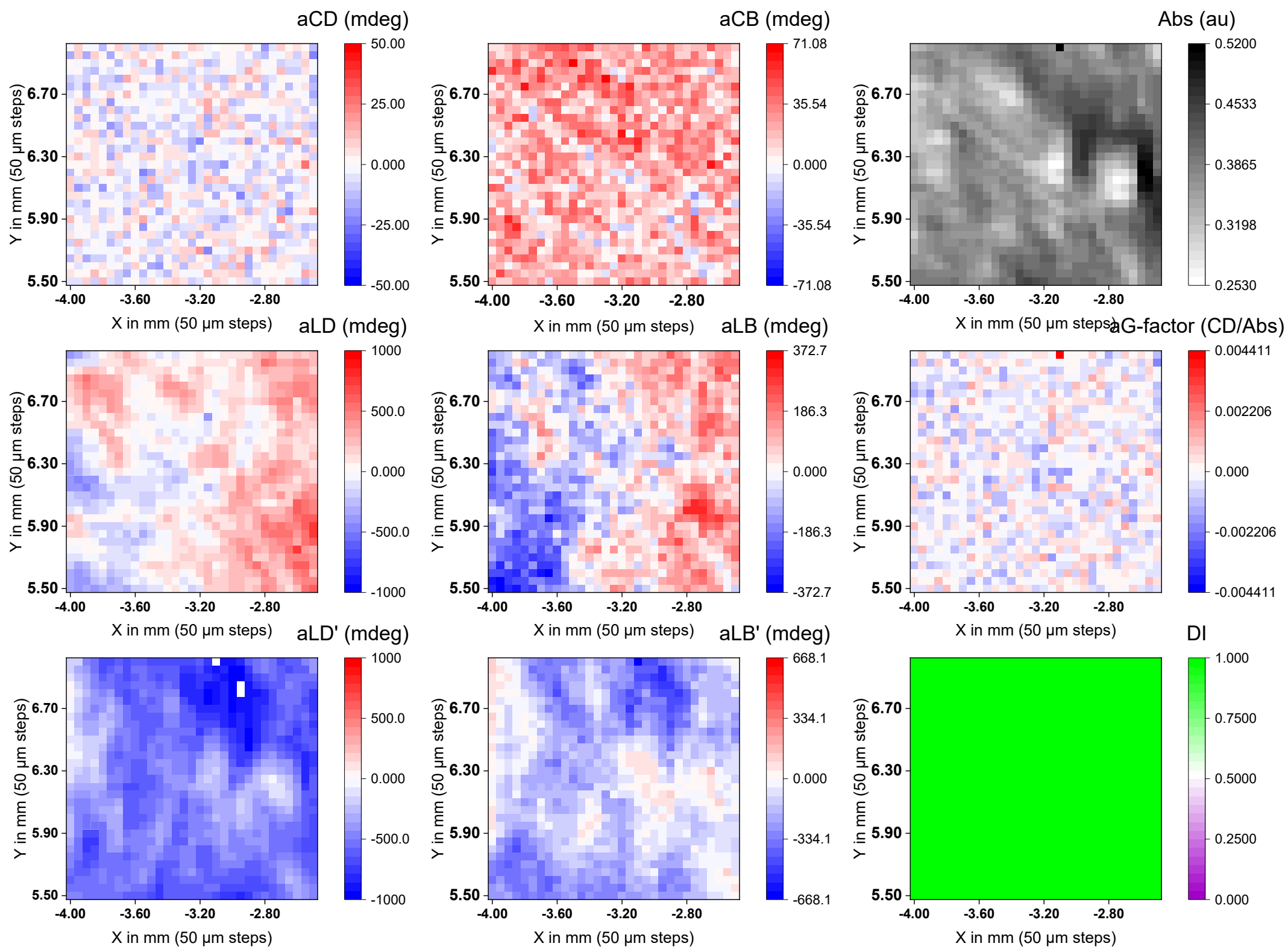

Figure S7.8: MMP map of racemic **1-*MM*/*PP*** macrocycle thin film at 31 x 31 steps of 50 µM recorded at 576 nm, baseline corrected for the glass substrate and processed using the Analytic Inversion method.

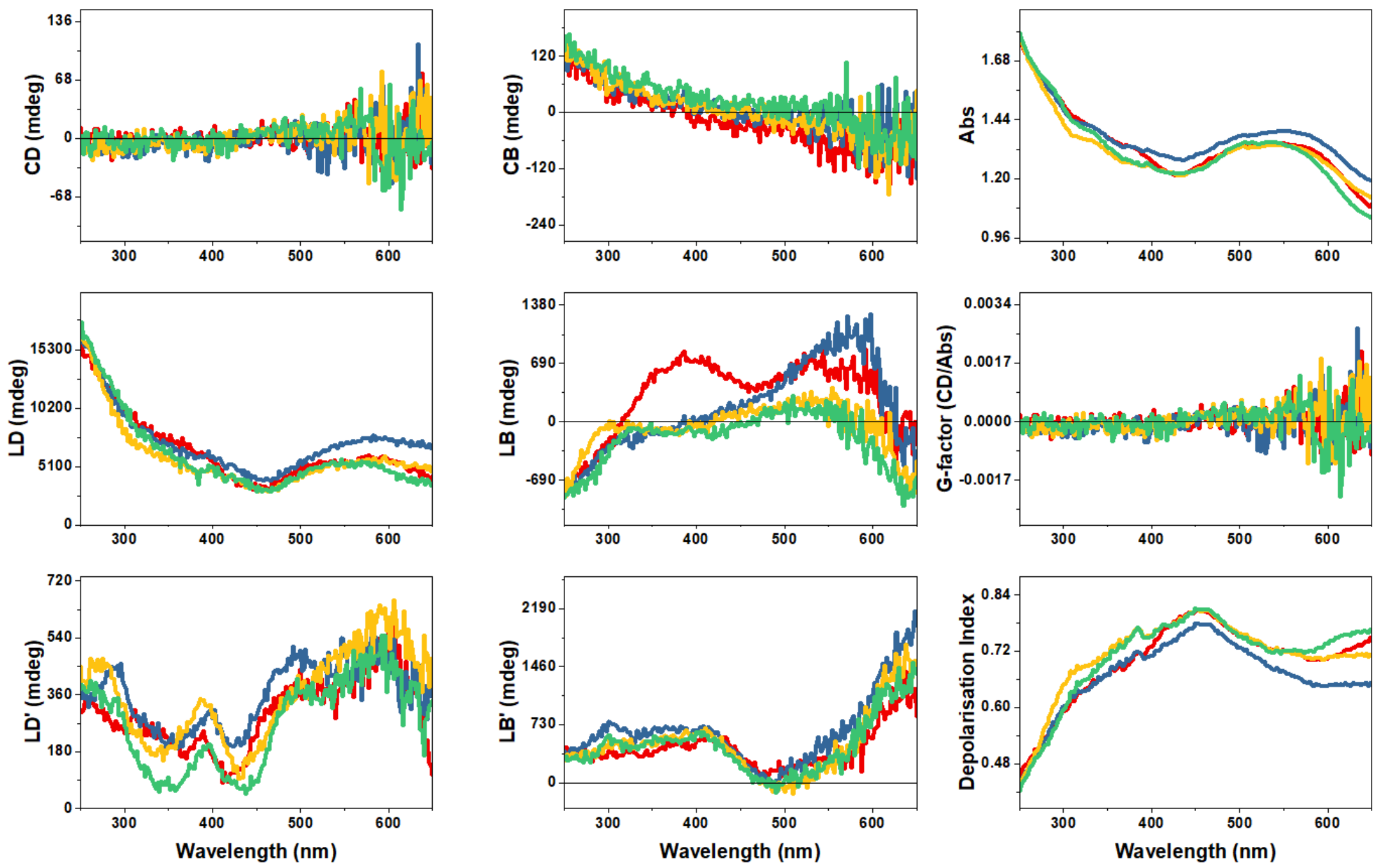

Figure S7.9: CD, CB, Abs, LD, LB, g_{abs}, LD', LB' and DI spectra of racemic **1-*MM*/*PP*** macrocycle thin film recorded at four different positions on the film processed with Analytical Inversion method.

b) Annealed films

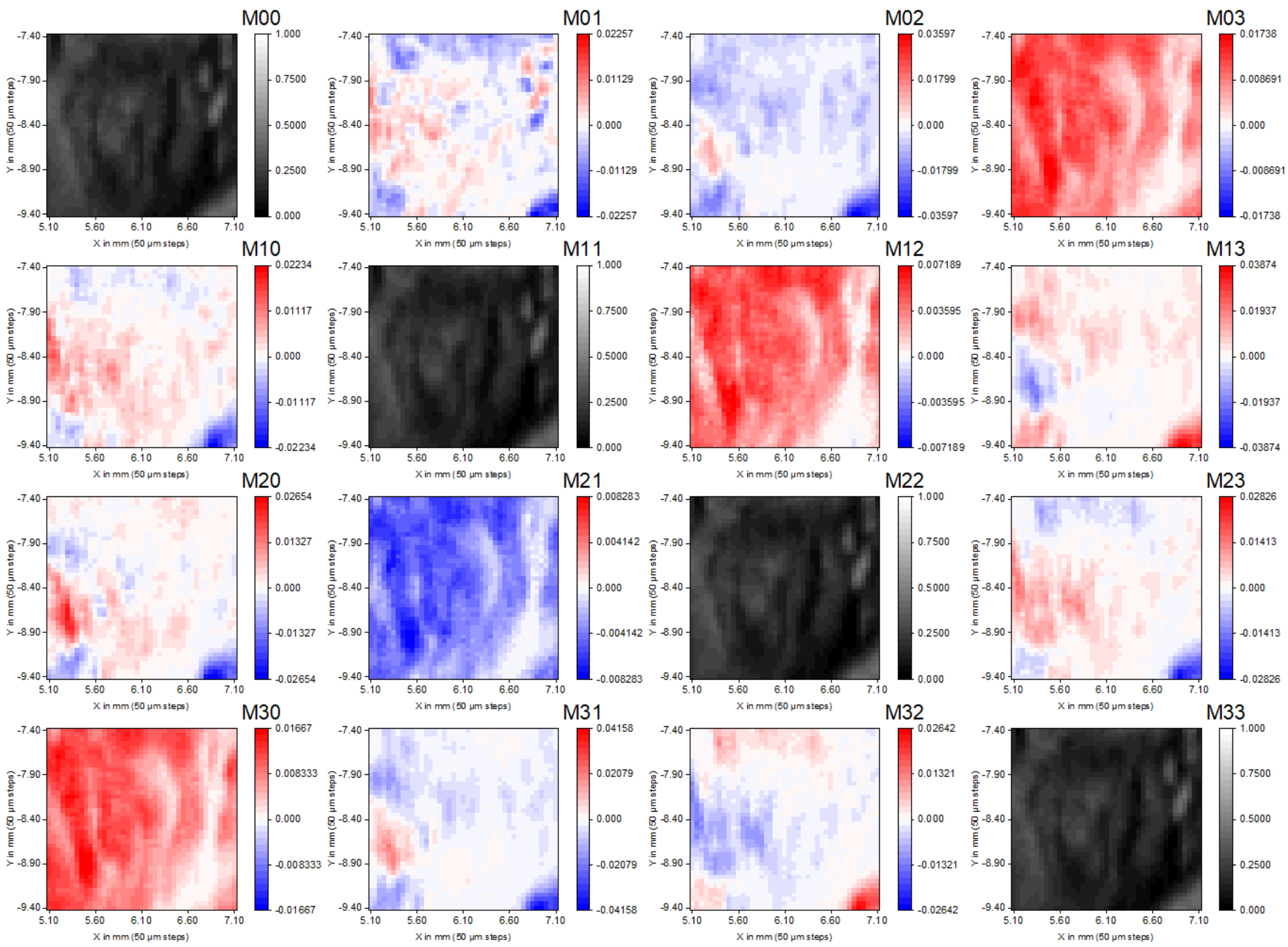

Figure S7.10: Raw data of differential 4×4 Mueller-Matrix of **1-*PP*** macrocycle thin film.

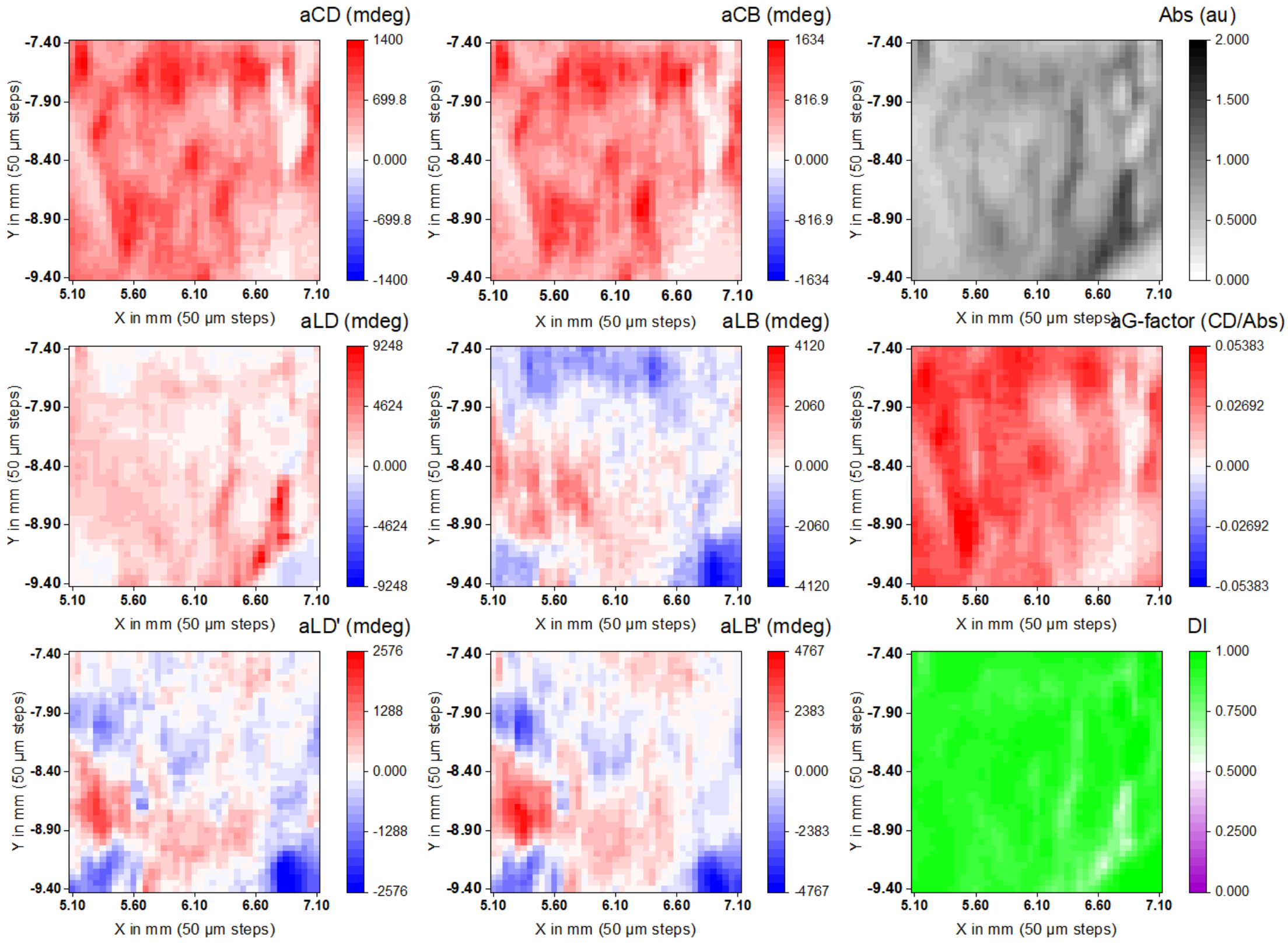

Figure S7.11: MMP map of **1**-***PP*** macrocycle thin film at 41 x 41 steps of 50 µM recorded at 590 nm, baseline corrected for the glass substrate and processed using the Analytic Inversion method.

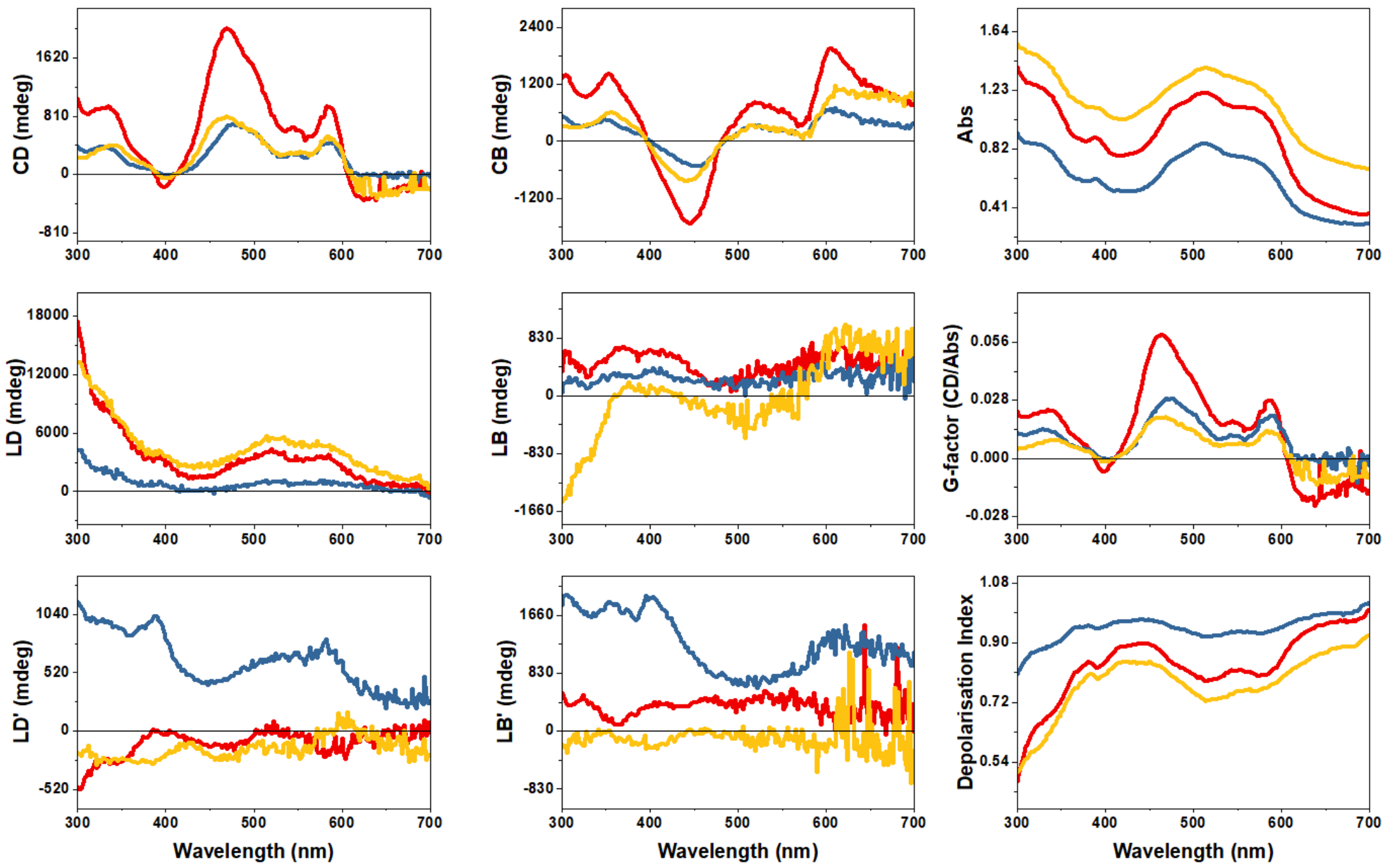

Figure S7.12: CD, CB, Abs, LD, LB, g_{abs}, LD', LB' and DI spectra of **1**-***PP*** macrocycle thin film recorded at three different positions on the film processed with Analytical Inversion method. The g_{abs} at λ > 600 nm is neglected due to noise.

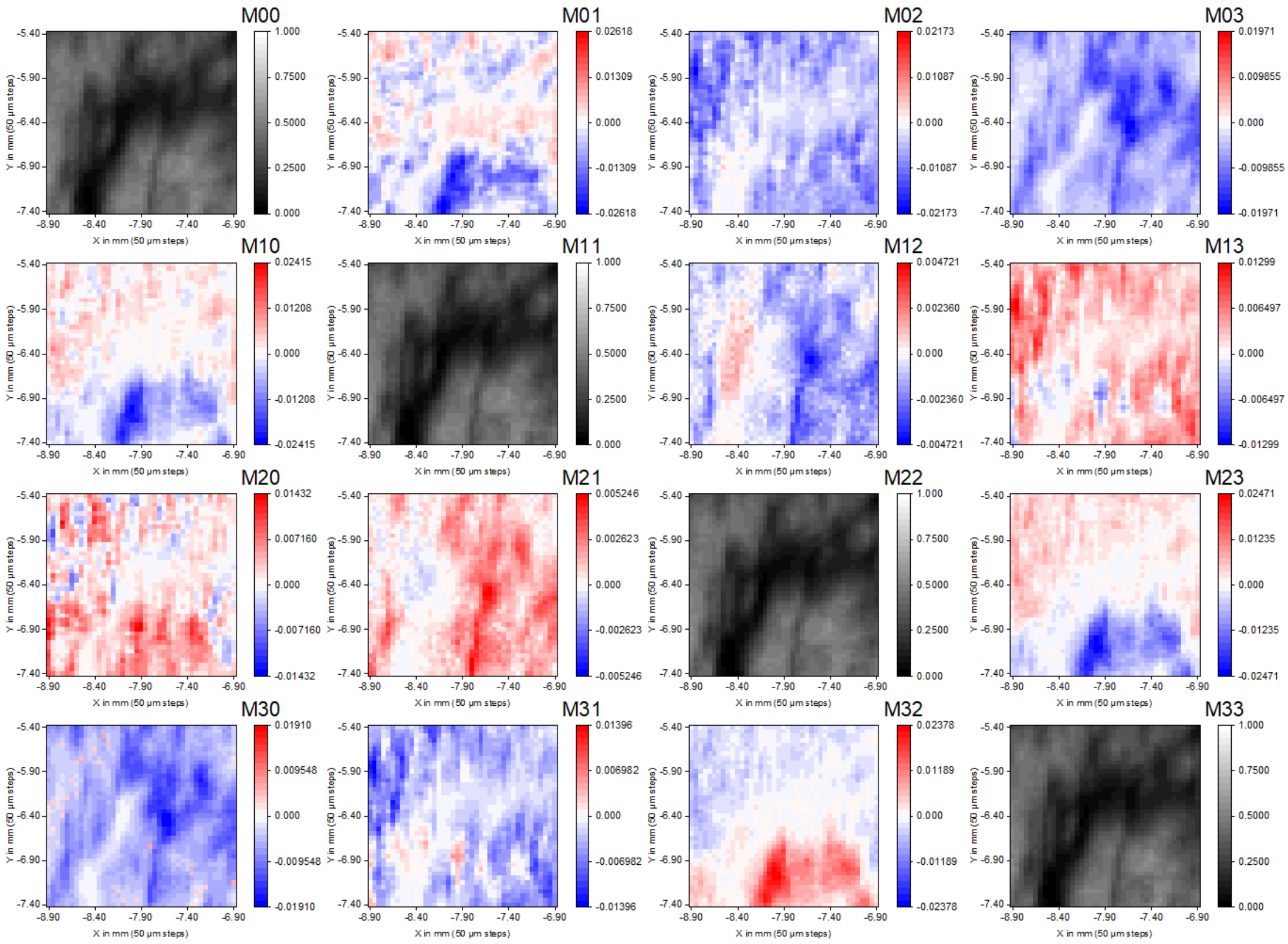

Figure S7.13: Raw data of differential 4×4 Mueller-Matrix of **1-*MM*** macrocycle thin film.

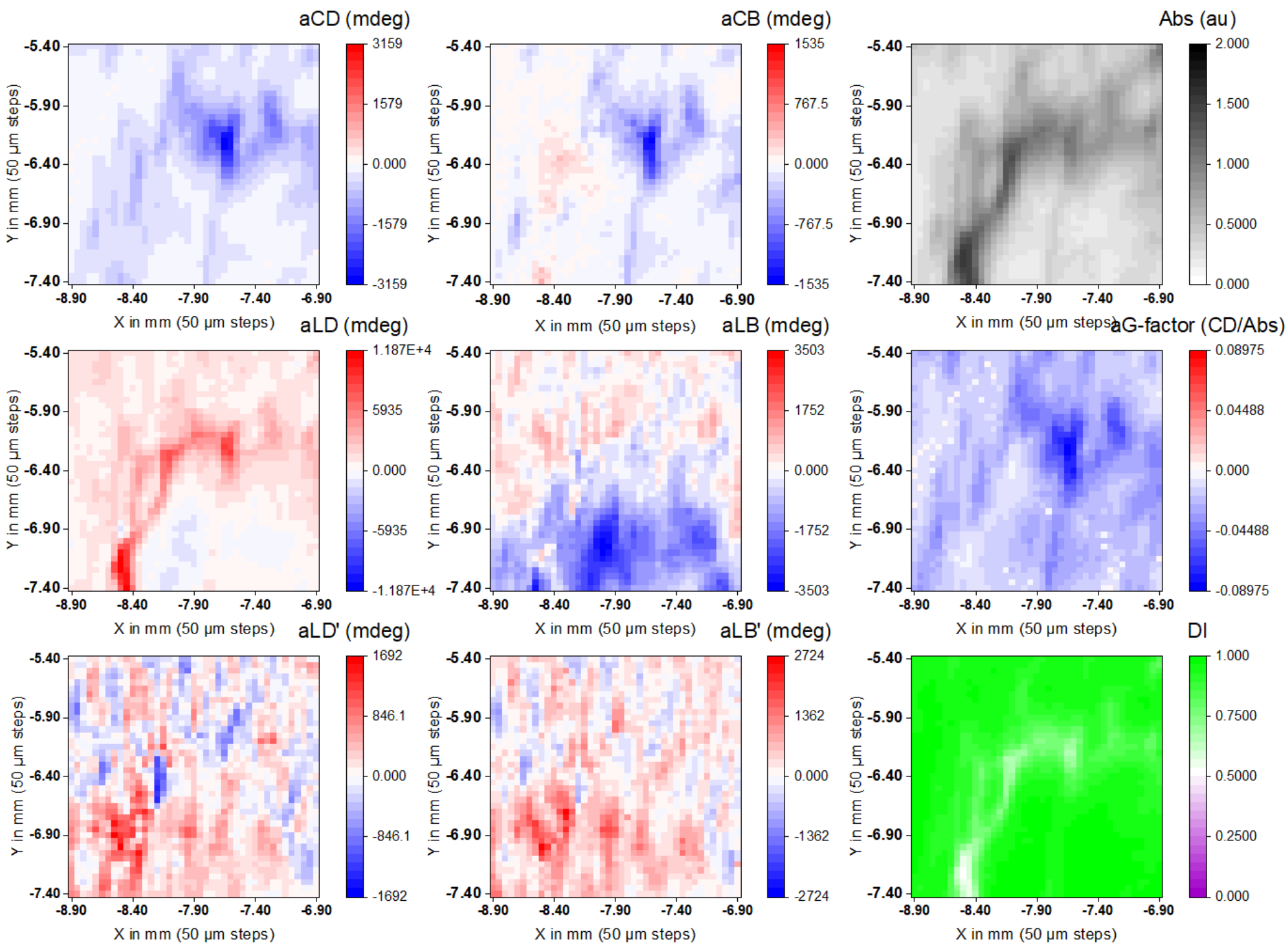

Figure S7.14: MMP map of **1-*MM*** macrocycle thin film at 41 x 41 steps of 50 μM recorded at 590 nm, baseline corrected for the glass substrate and processed using the Analytic Inversion method.

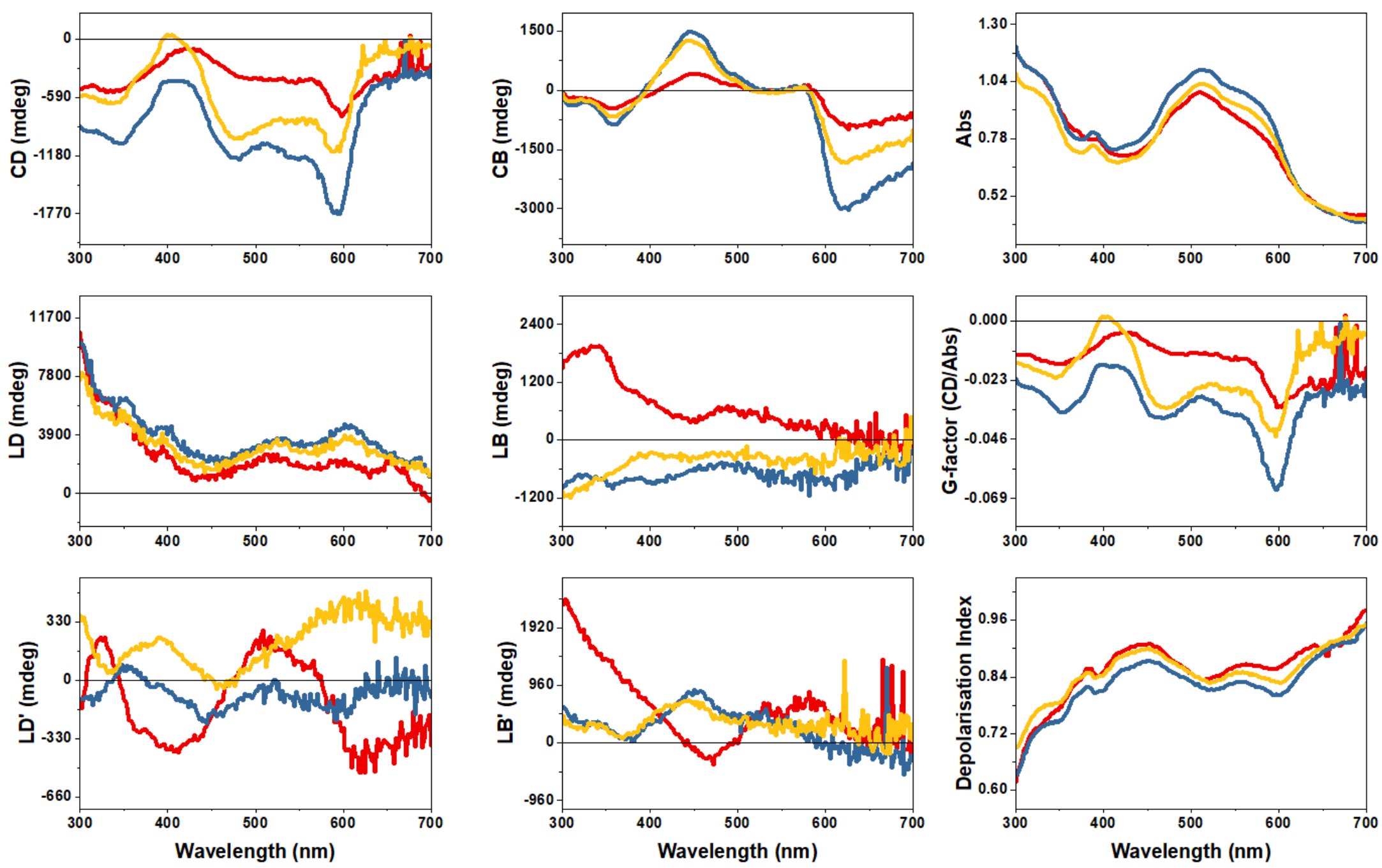

Figure S7.15: CD, CB, Abs, LD, LB, g_{abs}, LD', LB' and DI spectra of **1-*MM*** macrocycle thin film recorded at three different positions on the film processed with Analytical Inversion method.

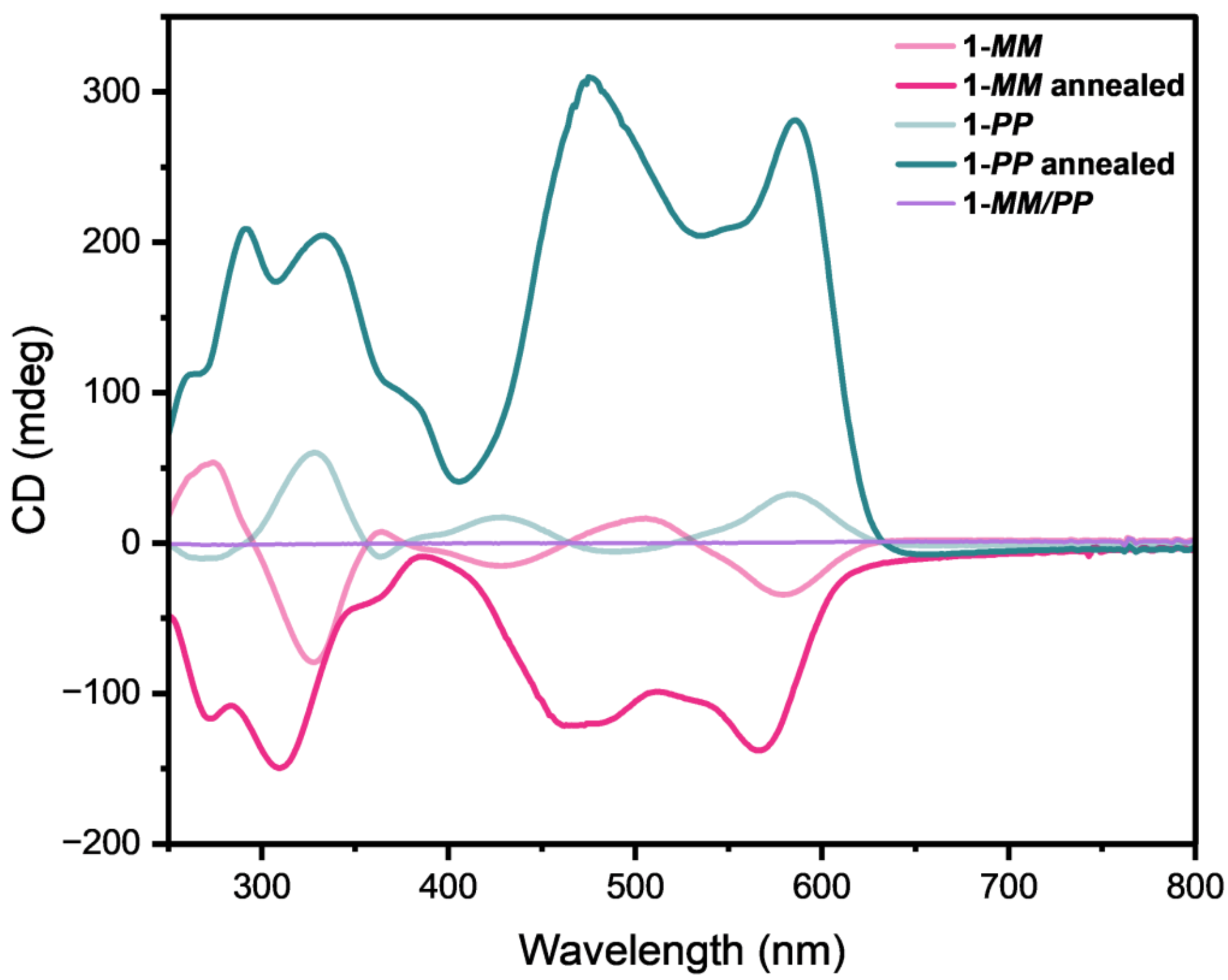

Figure S7.16: MMP CD spectra (averaged from three locations) of thin films of enantiopure **1**-***PP*** and **1-*MM*** before and after annealing, and a thin film of racemic **1-*MM/PP***.

c) Microscopy

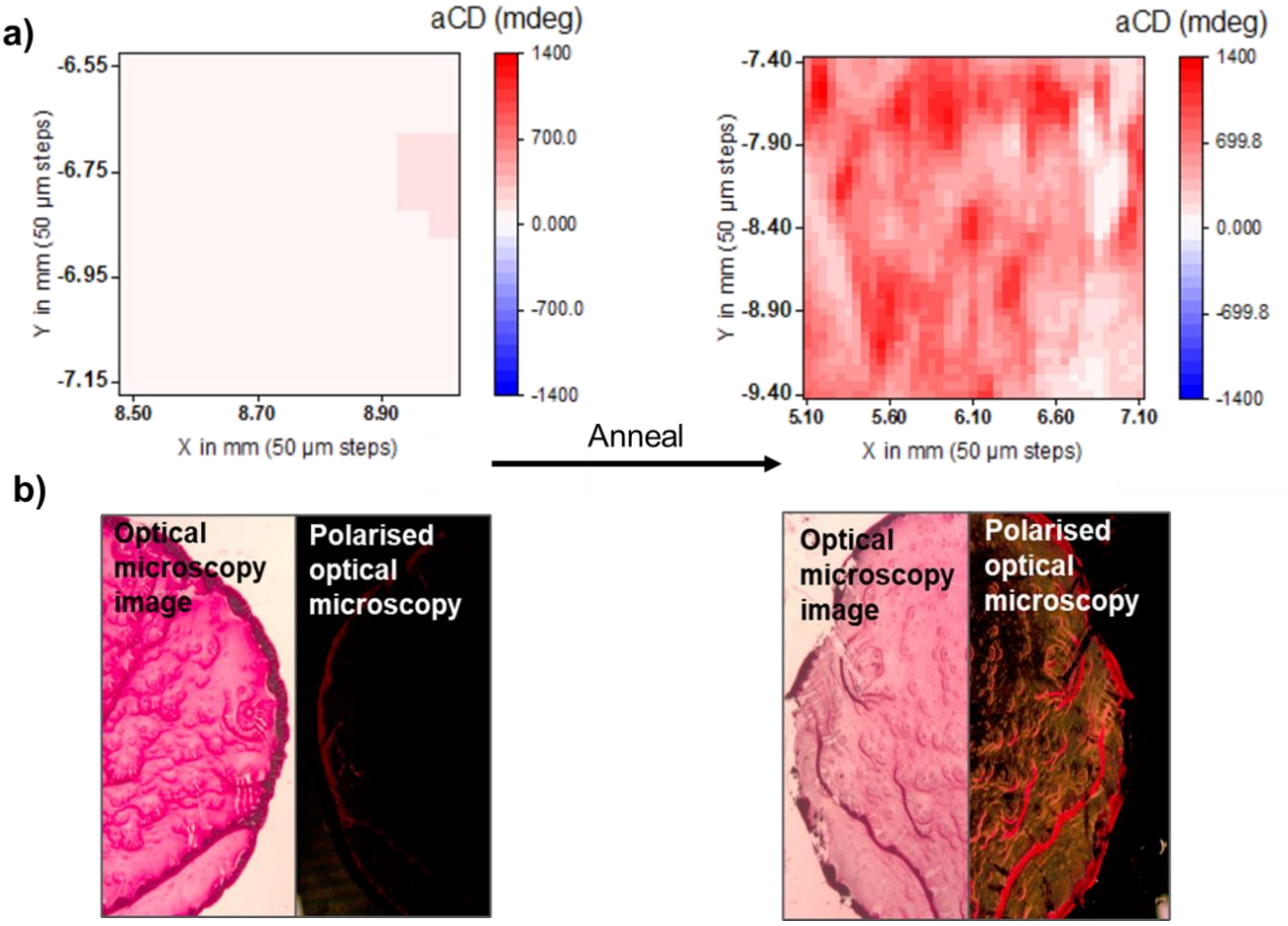

Figure S7.17: a) MMP CD maps of an area of the **1**-***PP*** thin film before (left) and after annealing (right) (50 µm spatial resolution, λ = 576/590 nm), b) Microscope images of an area of the **1**-***PP*** thin film before (left) and after annealing (right) showing thin film birefringence observed by polarised optical microscopy.

8. Circularly polarised luminescence

CPL was measured with a home-built (modular) spectrometer. The excitation source was a broad band (200 – 1000 nm) laser- driven light source EQ 99 (Elliot Scientific). The excitation wavelength was selected by feeding the broadband light into an Acton SP-2155 monochromator (Princeton Instruments, 7 μm slit); the collimated light was focused into the sample cell (1 cm quartz cuvette, temperature controlled at 20 ºC). Sample PL emission was collected perpendicular to the excitation direction with a lens (f = 150 mm). The emission was fed through a photoelastic modulator (PEM) (Hinds Series II/FS42AA) and through a linear sheet polariser (Comar). The light was then focused into a second scanning monochromator (Acton SP-2155, 7 μm slit) and subsequently on to a photomultiplier tube (PMT) (Hamamatsu H10723 series). The detection of the CPL signal was achieved using the field modulation lock-in technique. The electronic signal from the PMT was fed into a lock-in amplifier (Hinds Instruments Signaloc Model 2100). The reference signal for the lock-in detection was provided by the PEM control unit. The monochromators, PEM control unit and lock-in amplifier were interfaced to a desktop PC and controlled by a custom-written Labview graphic user interface. The lock-in amplifier provided two signals, an AC signal corresponding to (IL- IR) and a DC signal corresponding to (IL + IR) after background subtraction. The emission dissymmetry factor was therefore readily obtained from the experimental data, as 2 AC/DC. Errors of g_{lum} are ±5 x 10-5. The emission spectra were recorded with 2 nm step size, and the slits of the detection monochromator were set to a slit width corresponding to a spectral resolution of 3.5 nm. CPL spectra (as well as total emission spectra) were obtained through an averaging procedure of several scans. Measurements were performed in toluene at ~0.1 OD (~2.5 μM).

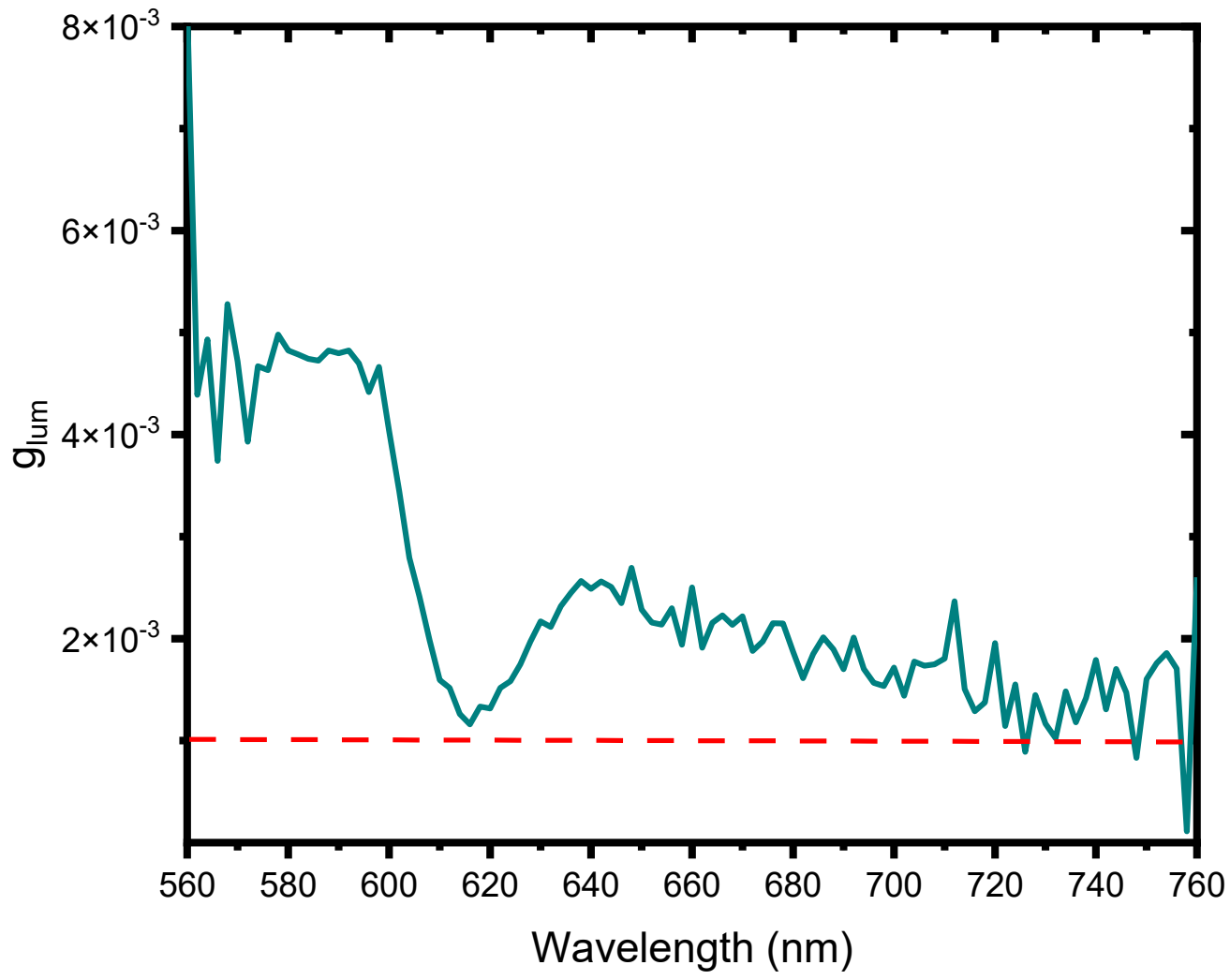

Figure S8.1: The g_{lum} spectrum for macrocycle **1-*MM/PP***, which is consistently $g_{lum} \geq 10^{-3}$ over 200 nm.

Table S8.1: A comparison of leading broadband CPL emitters (Δλ > 150 nm), with a focus on discrete small molecules (entries 2-19), akin to macrocycle **1.** Indicative polymeric materials are included in entries 20-24.

Entry	Material	Small molecule or Polymer	CPL Δλ (nm)	Distinct LE/EX CPL bands?	g_{lum} (×10^{-3})		Monosignate CPL?	Ref.
					LE band	EX band		
**1**	Perylene diimide dimer macrocycle **1**	S	200	✓	5	3	✓	This work
**2**	Pyrene dimer (acyclic pincer)	S	150	✓	1	16	✓	28
**3**	Pyrene dimer (acyclic pincer)	S	150	✓	0.7	4	-	29
**4**	Anthracene dimer macrocycle	S	200	✓	0.6	10	✓	30
**5**	Pyrene trimer (acyclic pincer)	S	200	✓	5	10	-	31
**6**	Pyrene dimer (macrocycle foldamer)	S	200	✓	7	30	-	32
**7**	Pyrene dimer (intermolecular complex)	S	250	✓	1	2	-	33
**8**	Naphthalene diimide dimer macrocycle	S	150	-	5		✓	34
**9**	Perylene dimer (in a [4]rotaxane)	S	150	-	20		✓	35
**10**	Thiazole orange dimer (ternary complex with cucurbituril)	S	150	-	10		✓	36
**11**	Pyrene dimer (acyclic pincer)	S	150	-	20		✓	37
**12**	Pyrene dimer macrocycle	S	150	-	30		✓	38
**13**	Pyrene dimer (in γ-cyclodextrin)	S	150	-	10		✓	39
**14**	Pyrene dimer (in a [8]rotaxane)	S	150	-	20		✓	40
**15**	Pyrene dimer (in a [2]catenane)	S	180	-	10		✓	41
**16**	Pyrene dimer (in a [4]rotaxane)	S	200	-	20		✓	42
**17**	Pyrene dimer macrocycle	S	200	-	20		✓	43
**18**	Pyrene dimer (in a helicene-based macrocycle)	S	250	-	40		✓	44
**19**	Guanidine–naphthalimide monomer	S	300	-	0.5		✓	45
**20**	Naphthalene diimide supramolecular polymer	P	200	-	20		✓	46
**21**	Polyacetylene liquid crystal	P	200	-	100		-	47
**22**	Pyrene–RNA polymer	P	250	✓	EX = 20<br>LE not reported		-	48
**23**	Triphenylamine supramol. polymer	P	250	-	4		✓	49
**24**	Polydibenzofulvene (conjugated polymer)	P	350	-	0.6		✓	50

Of the small molecule chiral emitters in Table S8.1 (entries 2-19), six systems are analogous to macrocycle **1** in that they exhibit distinct LE and EX CPL bands (entries 2-7, blue). In all these, the LE CPL band is substantially weaker than the EX band, while the two CPL bands are often of opposite sign (entries 3, 5, 6 and 7). In contrast, macrocycle **1** exhibits strong CPL from both LE/EX bands and retains the same sign throughout the CPL spectrum. This unique behaviour is due to the chiral twisted perylene diimide, which has intrinsic chirality in the LE state matched to that of the supramolecular EX state, while the chromophores in entries 2-7 are all planar.

9. Time-resolved fluorescence and femtosecond transient absorption spectroscopy

Fluorescence lifetime measurements

Time correlated single photon counting (TCSPC) measurements reveal clear solvent dependence across the PDI dimers. All samples were excited by ~1 ns pulses at either 485 or 570 nm and the fluorescence traces were detected at the steady-state fluorescence maxima (Fig. S9.1) or at the wavelengths indicated in the legend (630, 670 and 730 nm; Fig. S9.2), with 5 nm bandwidth. Measured fluorescence lifetimes are independent of the excitation and detection wavelengths for all samples.

The reference acyclic PDI monomer **5** displays exponential decay with lifetimes of 7.4 ns in toluene and 6.2 ns in DMSO, indicating only minor effects of solvent polarity on its excited-state dynamics. In toluene, **2-*MM*/*PP*** (8.5 ns) and **1-*MP*** (8.0 ns) dimers exhibit lifetimes comparable to that of the PDI monomer **5**. In contrast, the **1-*MM*/*PP*** shows a longer lifetime (16.5 ns), consistent with the formation of an excimer (EX) state.

In DMSO, all macrocycles show substantial lifetime shortening (3.4 ns for **1-*MM*/*PP***, 2.4 ns for **2-*MM*/*PP***, and 1.7 ns for **1-*MP***), consistent with the onset of nonradiative decay pathways. While the **1-*MM*/*PP*** dimer remains well described by exponential decay, both **2-*MM*/*PP*** and **1-*MP*** require biexponential fitting. These show a dominant fast component and a minor longer contribution (<5% amplitude), suggesting branching within the excited state potential energy surfaces. For nonexponential decays, reported lifetimes are weighted over amplitude contributions of τ_1 and τ_2.

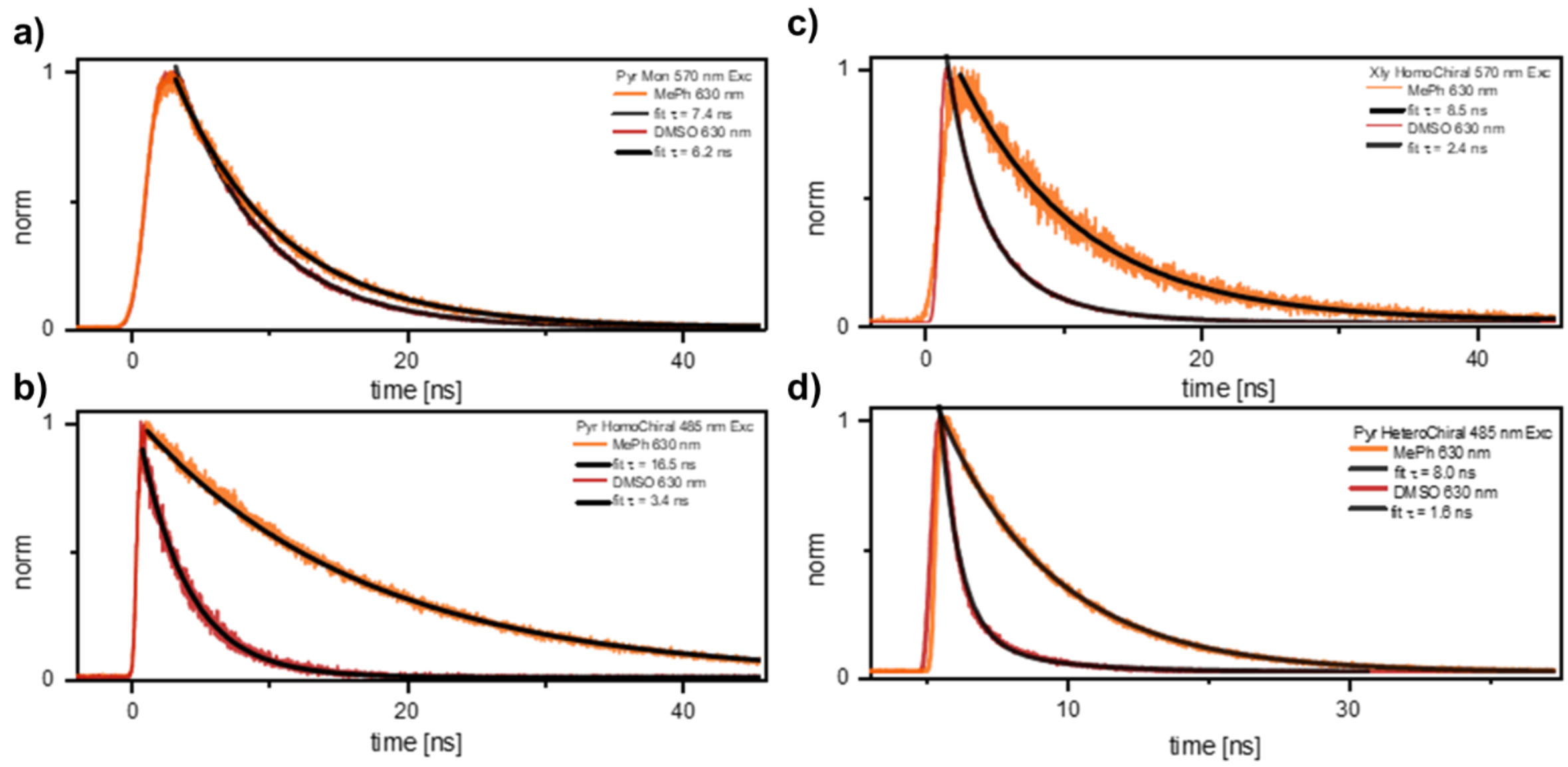

Figure S9.1: a) TCSPC traces of acyclic PDI **5** in toluene (orange) and DMSO (red) solvent, shown together with exponential fits. Fit results are shown in legend. (**b**, **c**, **d**) are same as (**a**) for **1-*MM/PP*, 2-*MM/PP*** and **1-*MP***, respectively.

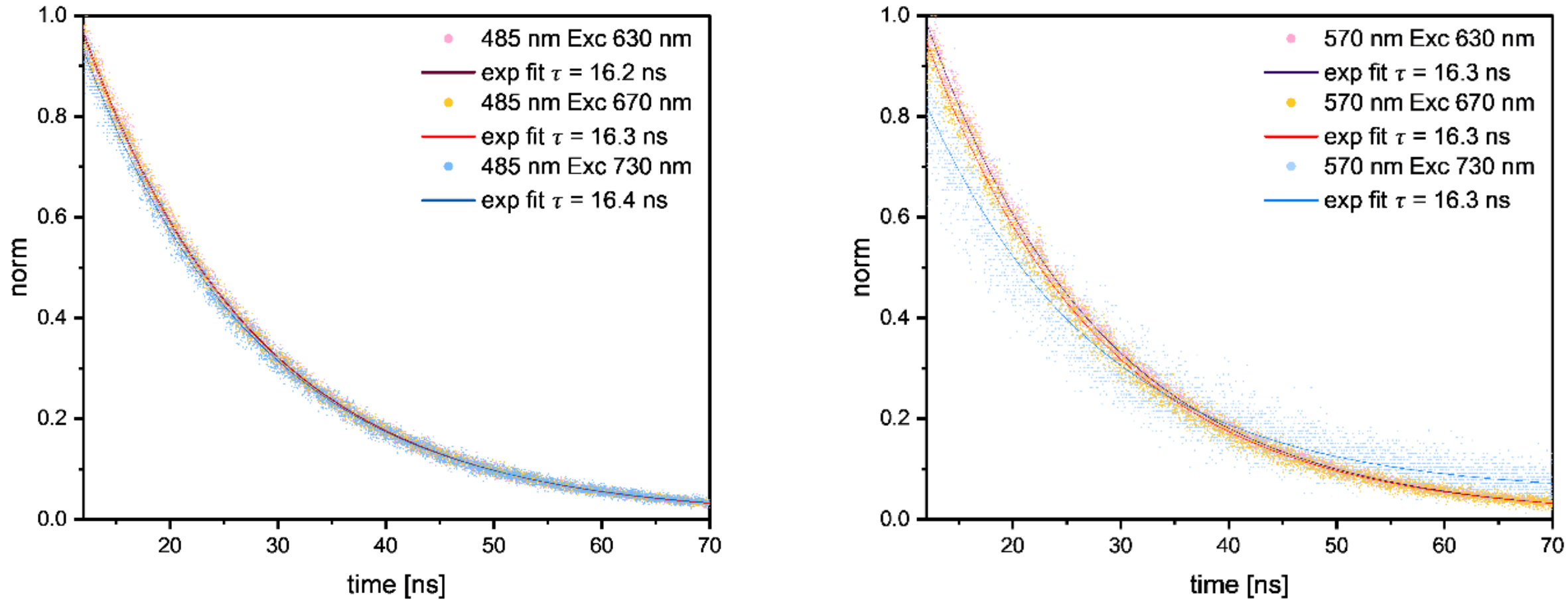

Figure S9.2: TCSPC traces of macrocycle **1-*MM*/*PP*** in toluene at different excitation wavelengths, shown together with exponential fits. Fit results are shown in legend.

fsTA spectroscopy: PDI monomer 5 in toluene

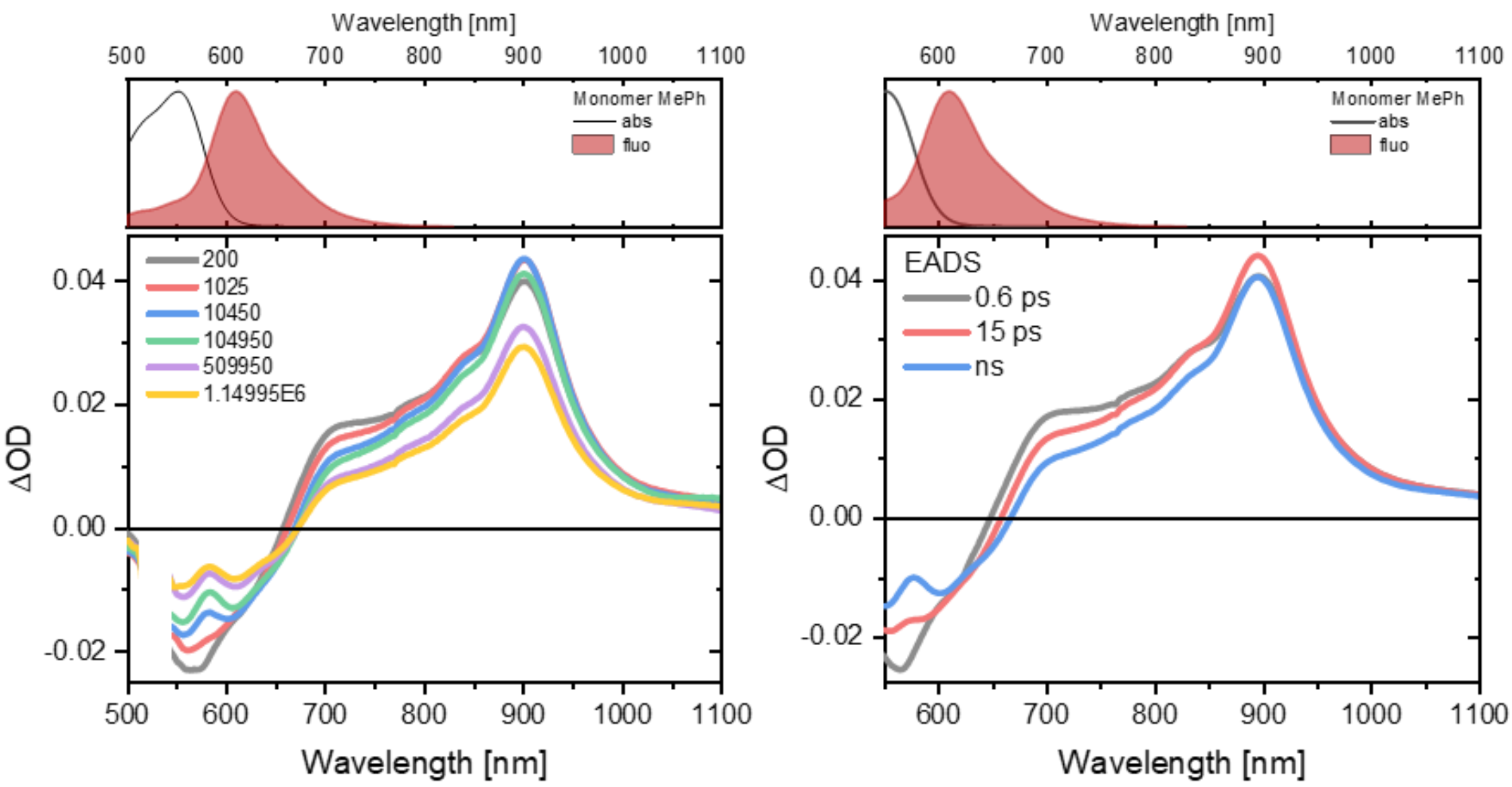

Figure S9.3: fsTA spectra of the PDI monomer **5** in toluene at selected pump probe delay times (left) and EADS from global fitting (right) in GloTarAn.[51] Three components were required to achieve a good fit, in agreement with Bressan et al.[52] ps components represent “intra-PDI/triazole” planarisation, whilst the long component was fixed to the fluorescence lifetime determined by TCSPC. Steady-state absorption (black) and emission (red shade) spectra are reproduced in the top panels as reference

fsTA spectroscopy in DMSO

In contrast to apolar toluene, the fluorescence shoulder of **1-*MM/PP*** and assigned to emission from a mixed LE/EX state is not observed in polar DMSO. Therefore, the PDI dimer macrocycles **1-*MM/PP*** and **1-*MP*** were investigated by fsTA spectroscopy in DMSO. fsTA data are shown in Figure S9.4a, c.

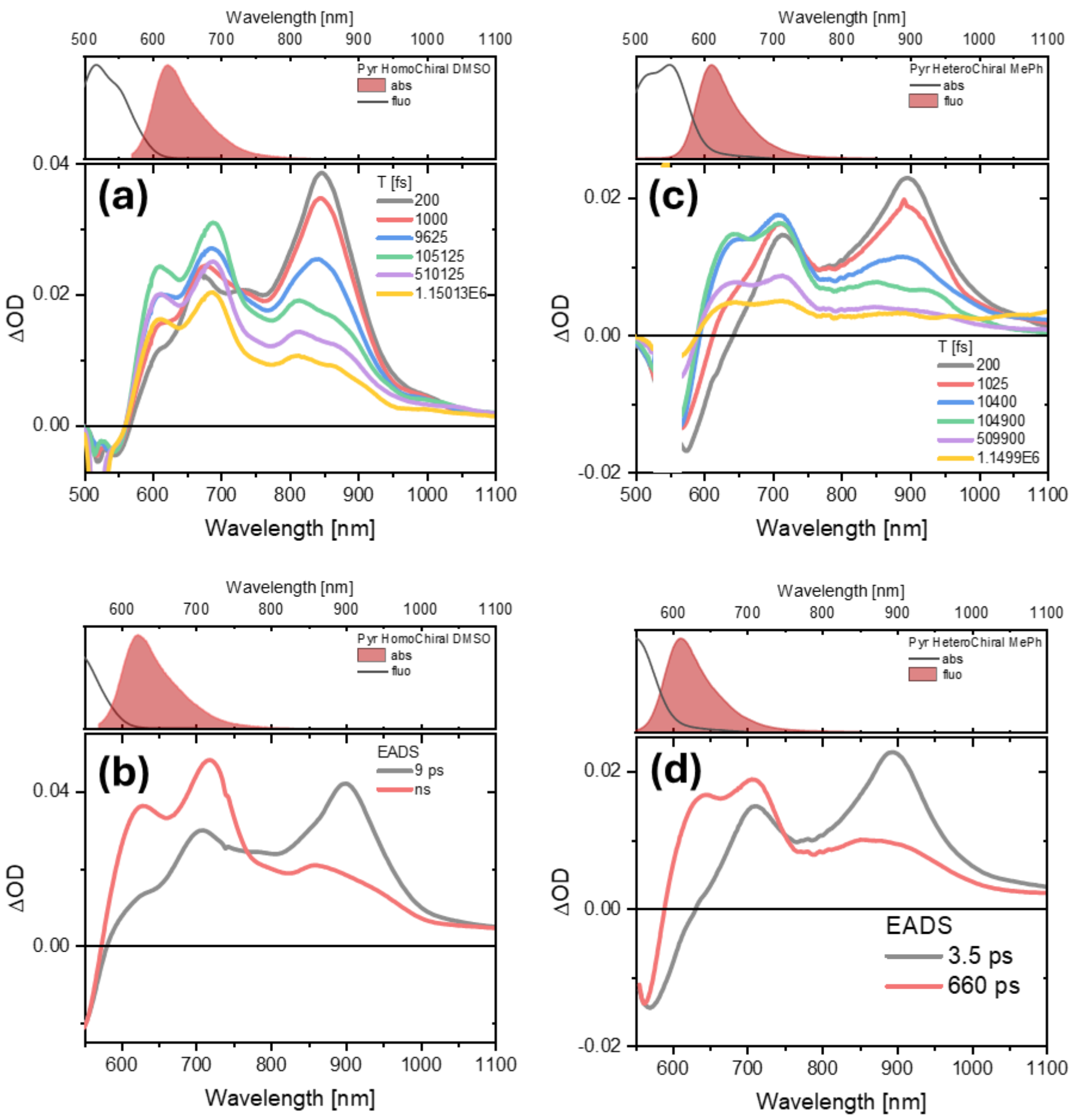

Figure S9.4: (**a**, **b**) fs transient absorption spectra at selected pump-probe delays and evolution associated differential spectra from global fitting of **1-*MM/PP*** in DMSO. (**c**, **d**) Same as (**a**, **b**) for **1-*MP***. Steady-state absorption (black solid) and fluorescence emission (red shade) spectra are reproduced in the top panels.

In DMSO, the fsTA responses differ markedly from the data in toluene: at the first measured pump-probe delay time (0.2 ps) stimulated emission (SE) is quenched, and the excited state absorption (ESA) exhibits a double maximum profile more structured than in toluene. On the picosecond timescale, all dimers show decay of the ~900 nm local excited state (LE) ESA, and the concomitant growth of new absorption bands at ~615 and ~710 nm, which persist for hundreds of ps.

The initial ESA of **1-*MM*/*PP*** (Figure S9.4b) differs from toluene, possibly reflecting enhanced charge transfer (CT) character in the region of the S_1 potential energy surface promptly accessed via vertical excitation, or evolution out of this region of the potential energy surface within the ~150 fs instrument response function. This assignment is supported by the lack of SE. Subsequently, in 9 ps, we observe decay of the ~900 nm LE ESA and the growth of bands

at ~615 and ~710 nm. These are assigned to PDI radical cation and anion absorption, respectively, indicating symmetry-breaking charge separation (SBCS) driven by the polar solvent environment, stabilising the radical pair state.[53,54] Similar behaviour is observed for ***1-MP*** whose EADS are shown in Figure S9.4d. SBCS occurs on the few ps timescale in both dimers and yields charge-separated states, persisting for 0.66-2.5 ns.

Despite clear evidence for SBCS in fsTA, fluorescence spectra in DMSO remain characteristic of LE emission (no EX shoulder) with 2-3 nanosecond lifetimes. As radical pair states are dark, this indicates that fluorescence arises from a residual LE population, while SBCS constitutes a competing, and dominant, nonradiative decay pathway. The reduced fluorescence lifetimes therefore reflect depopulation of the emissive LE state rather than emission from the charge-separated state (QY in DMSO = 5%), indicating branching within the S_1 manifold. This branching likely reflects population of multiple minima separated by barriers higher than kT in the S_1 potential. Hence, a subpopulation of photoexcited molecules is trapped in the LE state, possibly due to conformational disorder that prevents SBCS, as we have previously shown for a flexible PDI dimer in polar environment,[55] and given the near-unity fluorescence quantum yield of the LE state, even a minor such population contributes disproportionately to the observed steady-state and TCSPC fluorescence signals.

10. Computational chemistry

General

Density functional theory (DFT) calculations were performed in ORCA 6.0[56] unless otherwise indicated. All functionals and basis sets were chosen following the best-practice DFT protocols published by Grimme[57]. The following sections detail the calculations performed on macrocycle structures obtained from a) X-ray diffraction (XRD), b) DFT-optimised crystal structures in implicit toluene and c) CREST conformer searches.

a) Structures obtained directly from X-ray diffraction

Spectra prediction from single crystal structure of 1-*PP*

The absorbance and CD spectra of **1-*PP*** were predicted from the single crystal structure of the ***PP*** stereoisomer found in the racemic crystal dataset by means of TD-DFT using the TD-ωB97x[58] functional and the def2-SVP basis-set[59].

Table S10.1: Predicted vertical transitions and their oscillator and rotatory strengths (R) of **1-*PP***.

Transition	λ (nm)	f_{osc} (μ^2)	R (10^{-40} erg cm^3)
**1**	464.4	0.17344	731.5672
**2**	432.4	0.780709	-751.119
**3**	338.9	0.016149	11.6566
**4**	338.7	0.018758	74.61745
**5**	329.5	0.038437	16.26586
**6**	324.3	0.011232	58.78643
**7**	314.3	0.028076	22.3117
**8**	309.5	0.059215	-68.3361
**9**	307	0.007314	-87.9165
**10**	303.5	0.052358	-25.8065

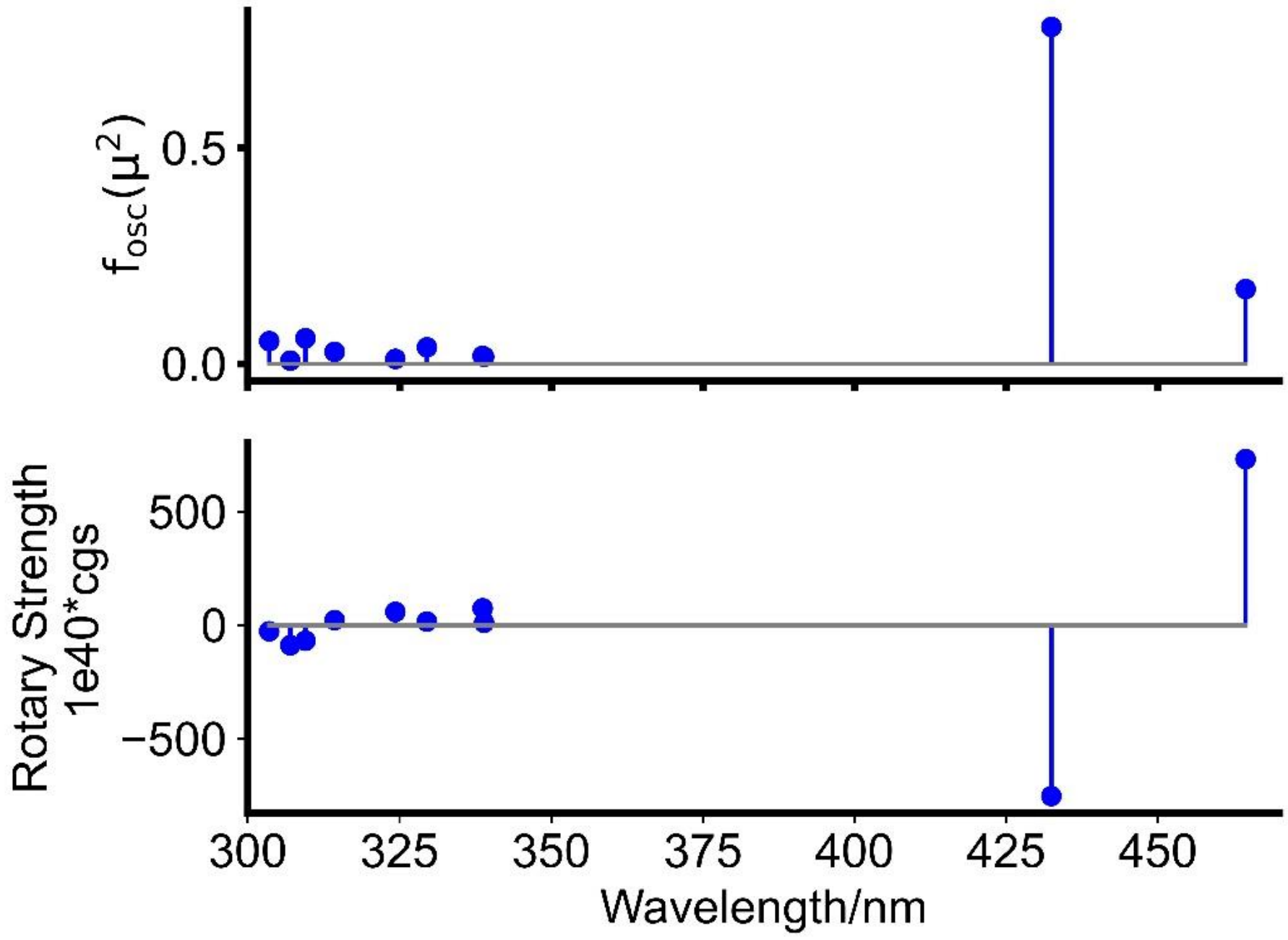

Figure S10.1: Predicted UV-vis and CD spectra of **1-*PP***.

Spectra prediction from single crystal structure of 1-*MP*

The absorbance and CD spectra of **1-*MP*** were predicted from its single crystal structure by means of TD-DFT using the TD-ωB97x[58] functional and the def2-SVP basis-set.[59]

Table S10.2: Predicted vertical transitions and their oscillator and rotatory strengths (R) of **1-*MP***.

Transition	λ (nm)	f_{osc} (μ^2)	R (10^{-40} erg cm^3)
**1**	527.6	1.08E-05	-0.50567
**2**	524.3	1.382692	0.46695
**3**	498.7	3E-09	-0.02809
**4**	498.6	0.004865	0.05607
**5**	381.4	1.38E-06	-0.08119
**6**	380.7	0.017523	0.08777
**7**	364.9	5.2E-08	0.10226
**8**	364.8	0.000201	-0.09977
**9**	360.6	0	0.00413
**10**	359.9	0.017661	-0.00727

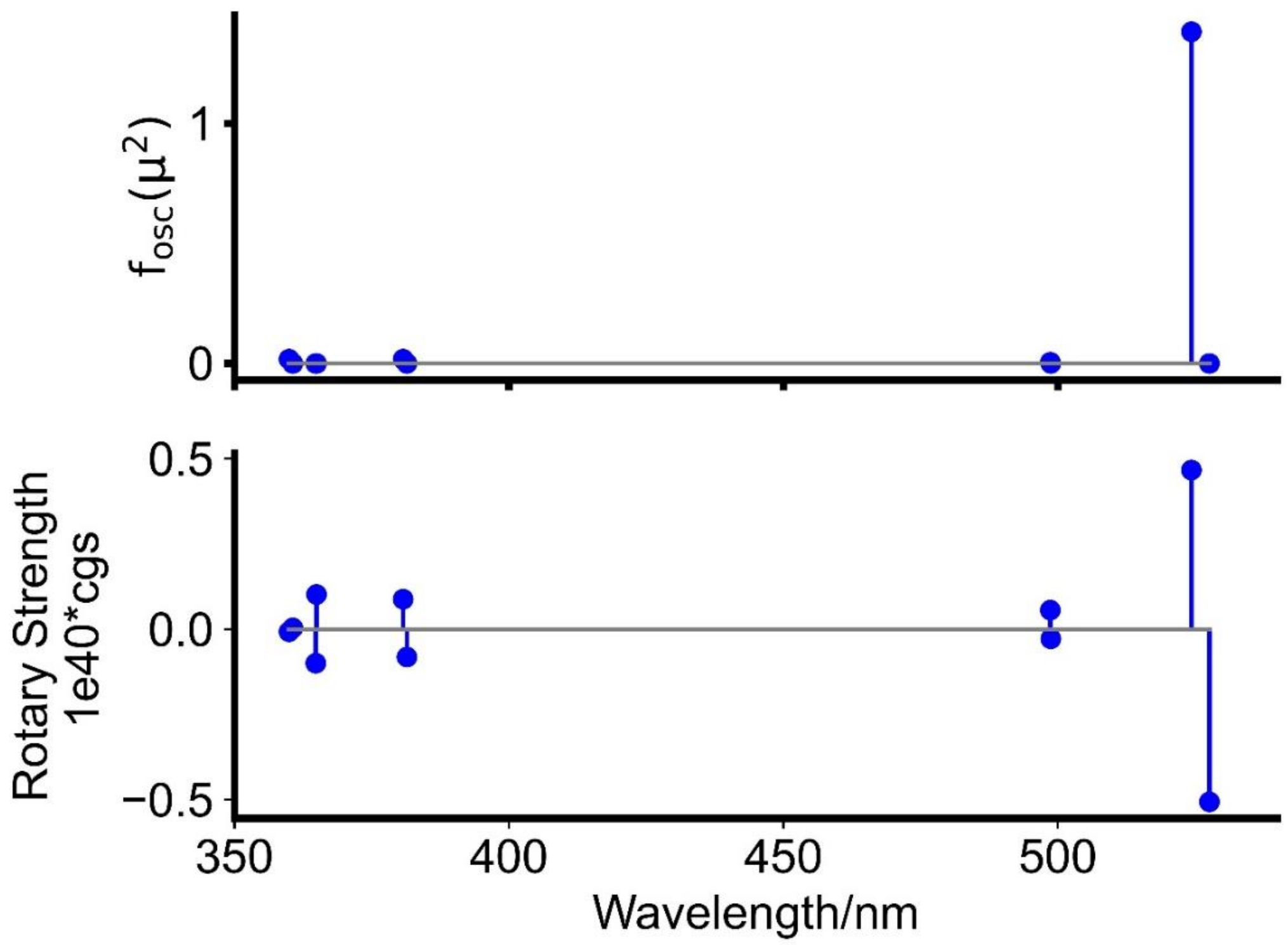

Figure S10.2: Predicted UV-vis and CD spectra of **1-*MP***

b) Structures obtained from DFT-optimisation of XRD structures with implicit solvation

DFT-optimisation of crystal structures

For both homochiral macrocycles **1** (*m*-pyridyl) and **2** (*m*-xylyl), DFT geometry optimisations (B97-3c functional with a def2-TZVP basis set and implicit solvation in toluene using the Conductor-like Polarizable Continuum Model [CPCM]) were carried out starting from the XRD structure of **1-*PP***. As single crystals of **2-*MM*/*PP*** could not be obtained, the initial XRD-derived structure of **2-*PP*** was generated by replacing the pyridyl nitrogen atoms in the XRD structure of **1-*PP*** with C–H groups. The corresponding .xyz files "1-PP_OPT_B973c-CPCM" and "2-PP_OPT_B973c-CPCM" are available in the folder "DFT_structure".

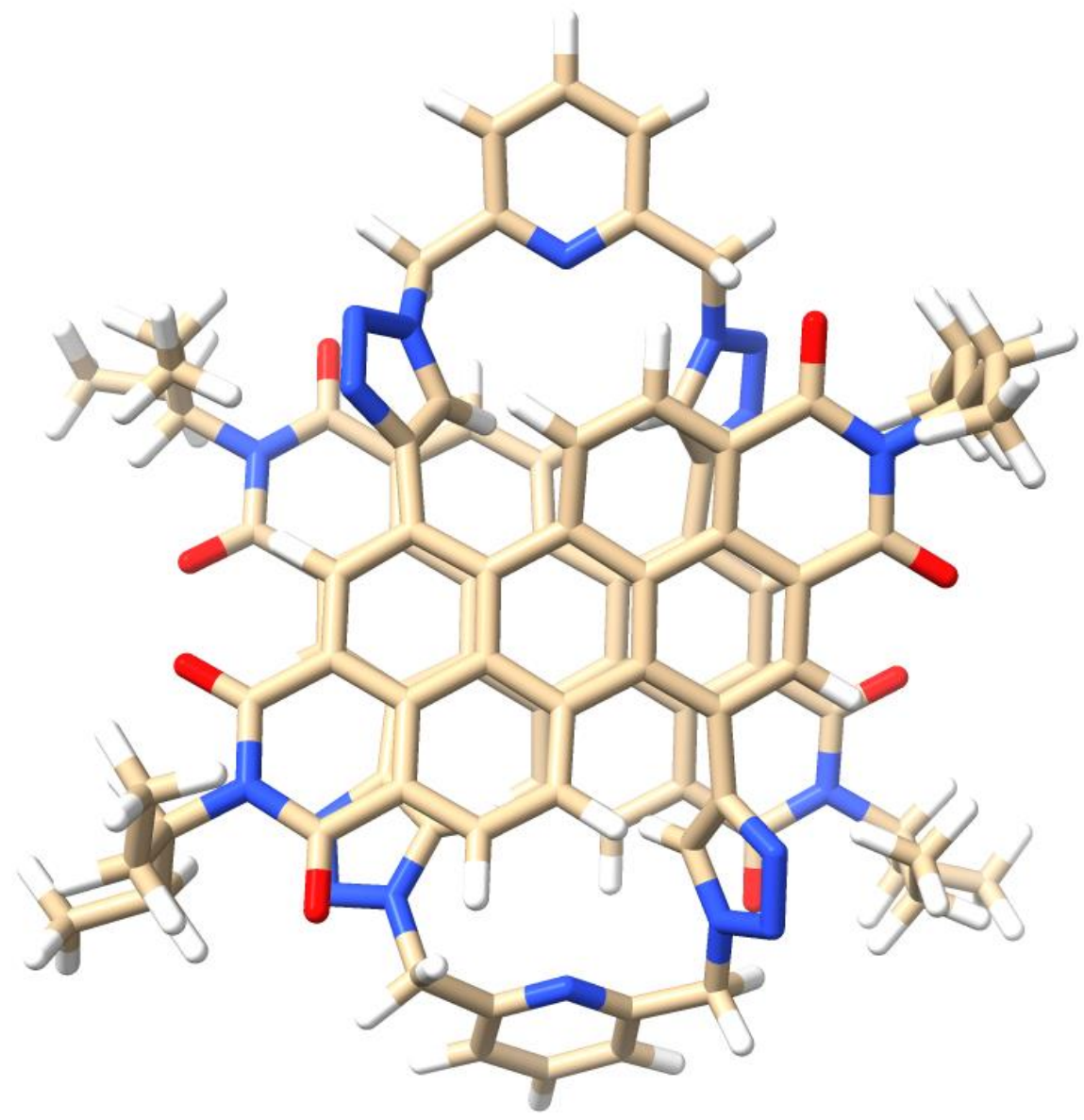

Figure S10.3: Structure of **1-*PP*** obtained by DFT geometry optimisation (B97-3c functional with a def2-TZVP basis set) of the XRD structure with implicit solvation in toluene (using the CPCM).

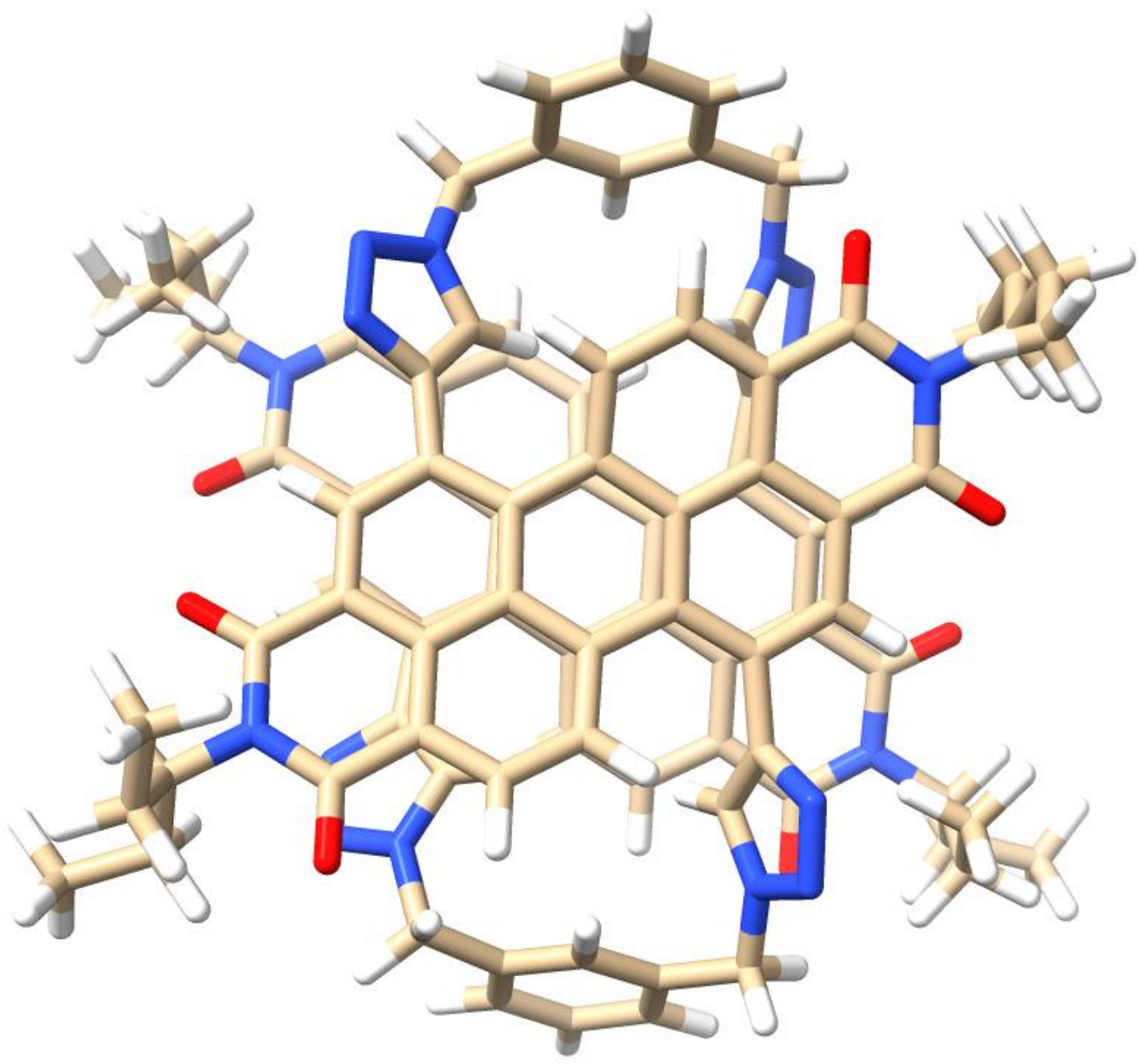

Figure S10.4: Structure of **2-*PP*** obtained by DFT geometry optimisation (B97-3c functional with a def2-TZVP basis set) of a modified XRD structure of **1-*PP*** with implicit solvation in toluene (using the CPCM).

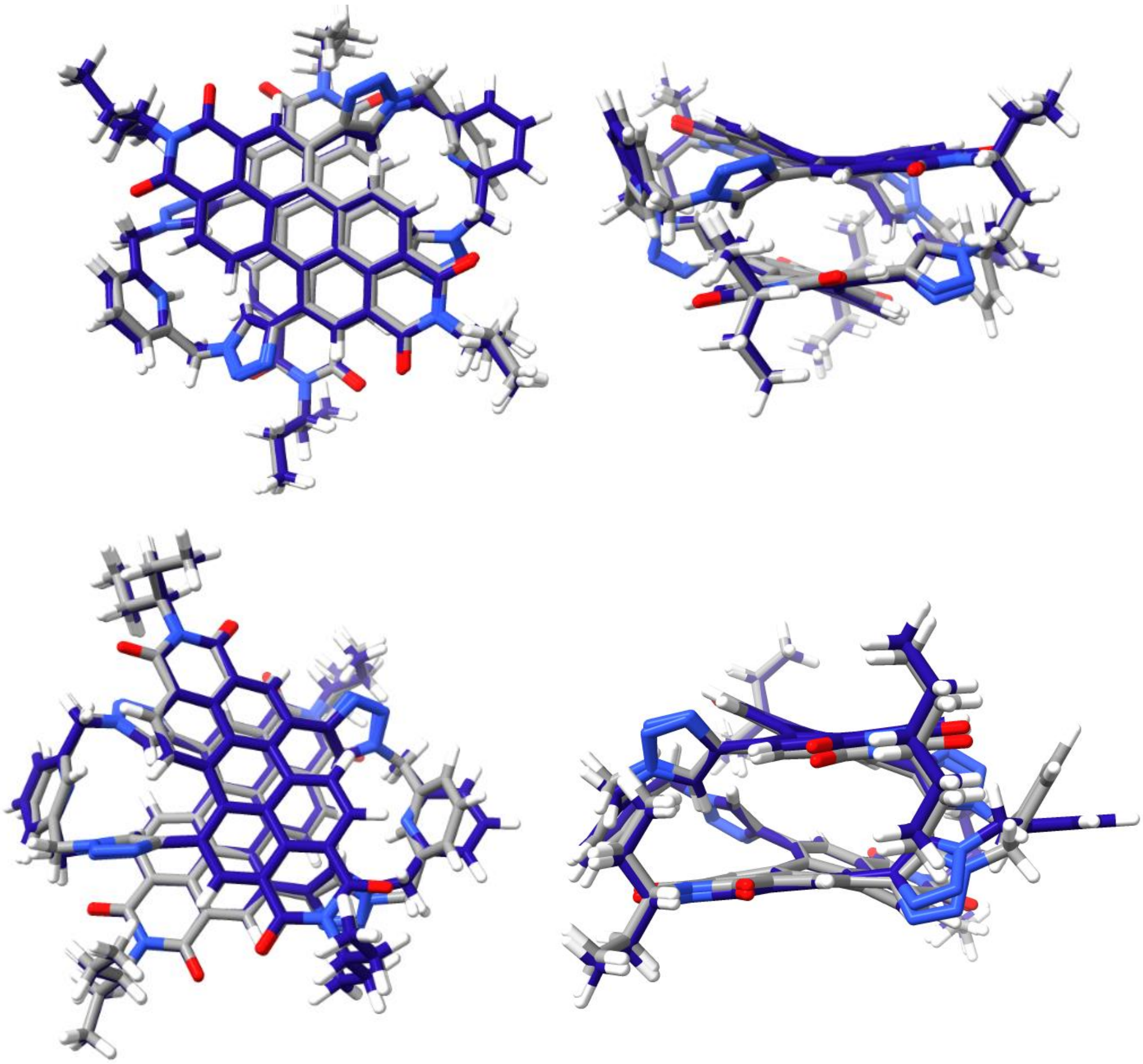

Figure S10.5: Overlays of DFT-optimised structures of **1-*PP*** (C atoms = dark blue) and **2-*PP*** (C atoms = grey). This shows a close similarity in the PDI dimer geometry; the interchromophore separation ($d_{\pi-\pi}$ = 3.9 Å), dimer tilt angle (α = 60°), PDI twist (α = 27°) and long-axis displacement (d = 0 Å) are essentially identical in both structures. However, one of the *m*-pyridyl groups in **1-*PP*** is rotated towards the ditriazole PDI units, adopting a geometry conducive to hydrogen bonding with the triazole C–H groups.

Spectra Prediction

The absorbance spectra of **1-*PP*** and **2-*PP*** were predicted from their DFT-optimised XRD-derived structures by means of TD-DFT using the TD-ωB97x[58] functional and the def2-SVP basis-set[59] and implicit solvation in toluene using CPCM.

Table S10.3: Predicted vertical transitions and their oscillator strengths of **1-*PP***.

Transition	λ (nm)	f_{osc} (μ^2)
**1**	494.6	0.24413
**2**	469	1.15885
**3**	346.8	0.02398
**4**	343.8	0.02201
**5**	339.3	0.02377
**6**	329.5	0.02662
**7**	316.3	0.08182
**8**	312.6	0.01197
**9**	311.4	0.27985
**10**	308.6	0.02457

Table S10.4: Predicted vertical transitions and their oscillator strengths of **2-*PP***.

Transition	λ (nm)	f_{osc} (μ^2)
**1**	492.1	0.18142
**2**	471.8	1.16354
**3**	351	0.01734
**4**	349.3	0.01948
**5**	338	0.01080
**6**	332.5	0.11334
**7**	315.9	0.00590
**8**	312.9	0.26351
**9**	309.5	0.01866
**10**	307.8	0.05398

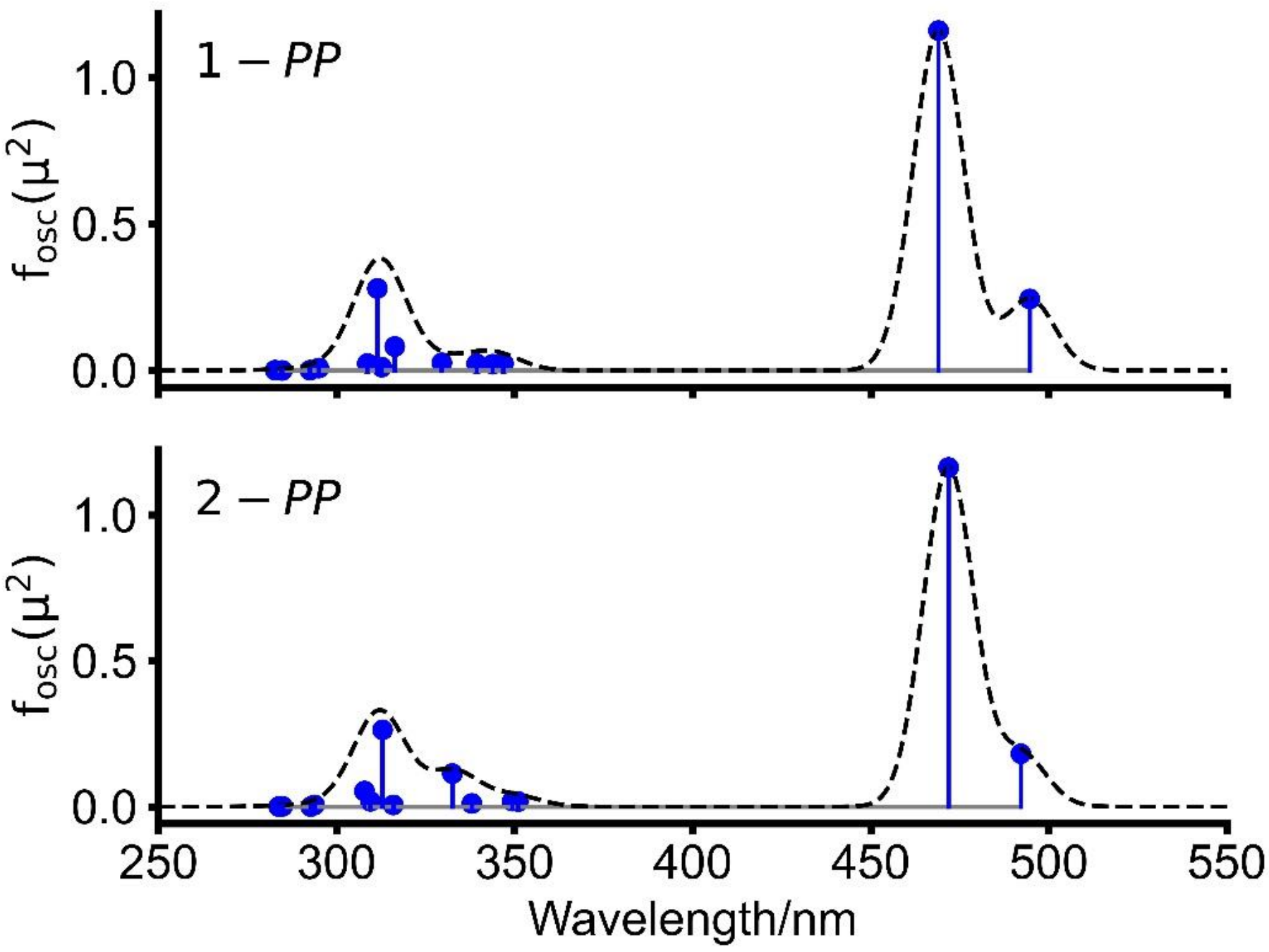

Figure S10.6: Predicted UV-vis spectra of **1**-***PP* *and*** **2**-***PP***.

Orbital diagrams

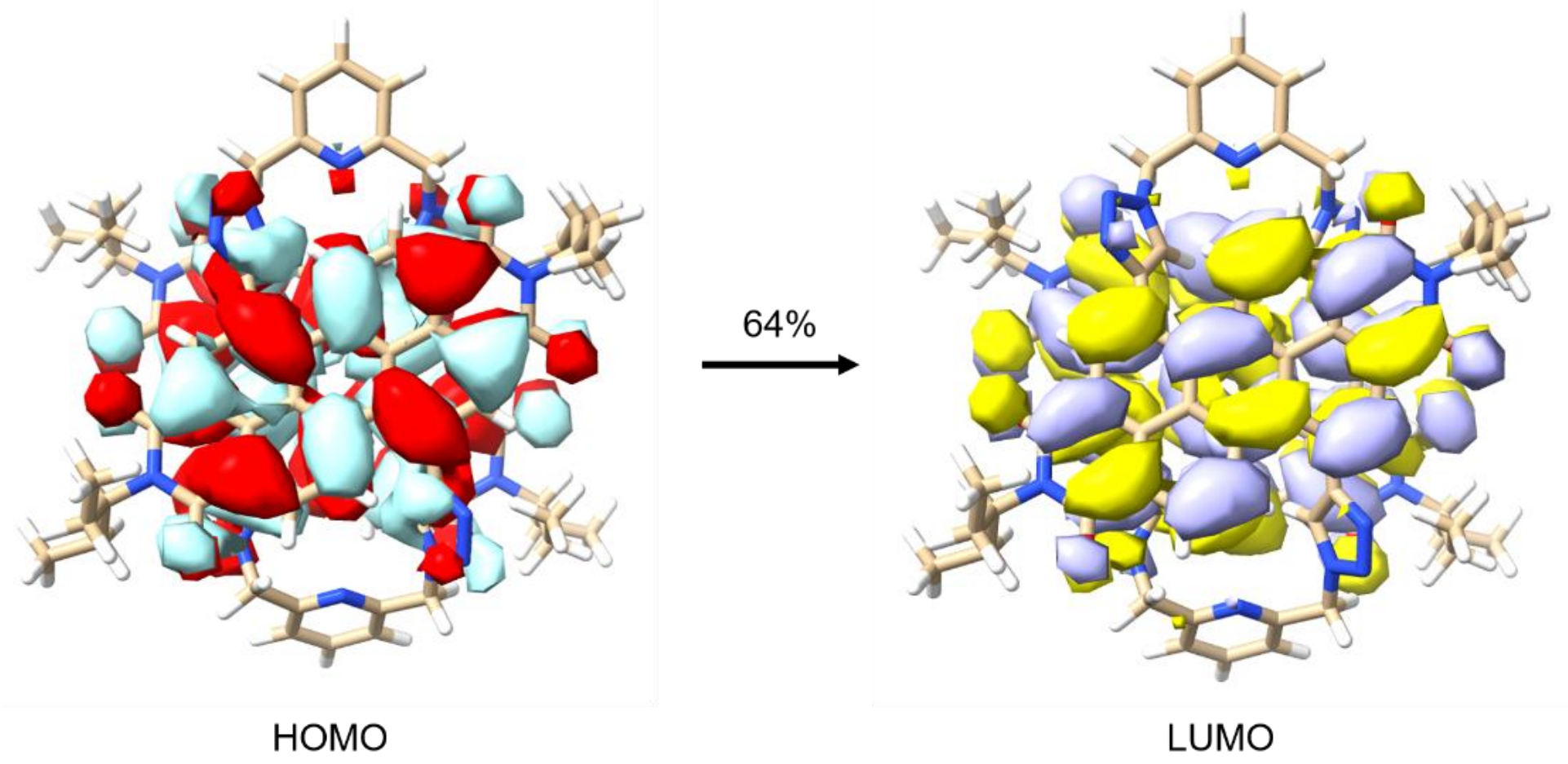

Figure S10.7: Orbital diagram of the major contribution to transition 1 for **1-*PP***. This corresponds to the HOMO-LUMO transition.

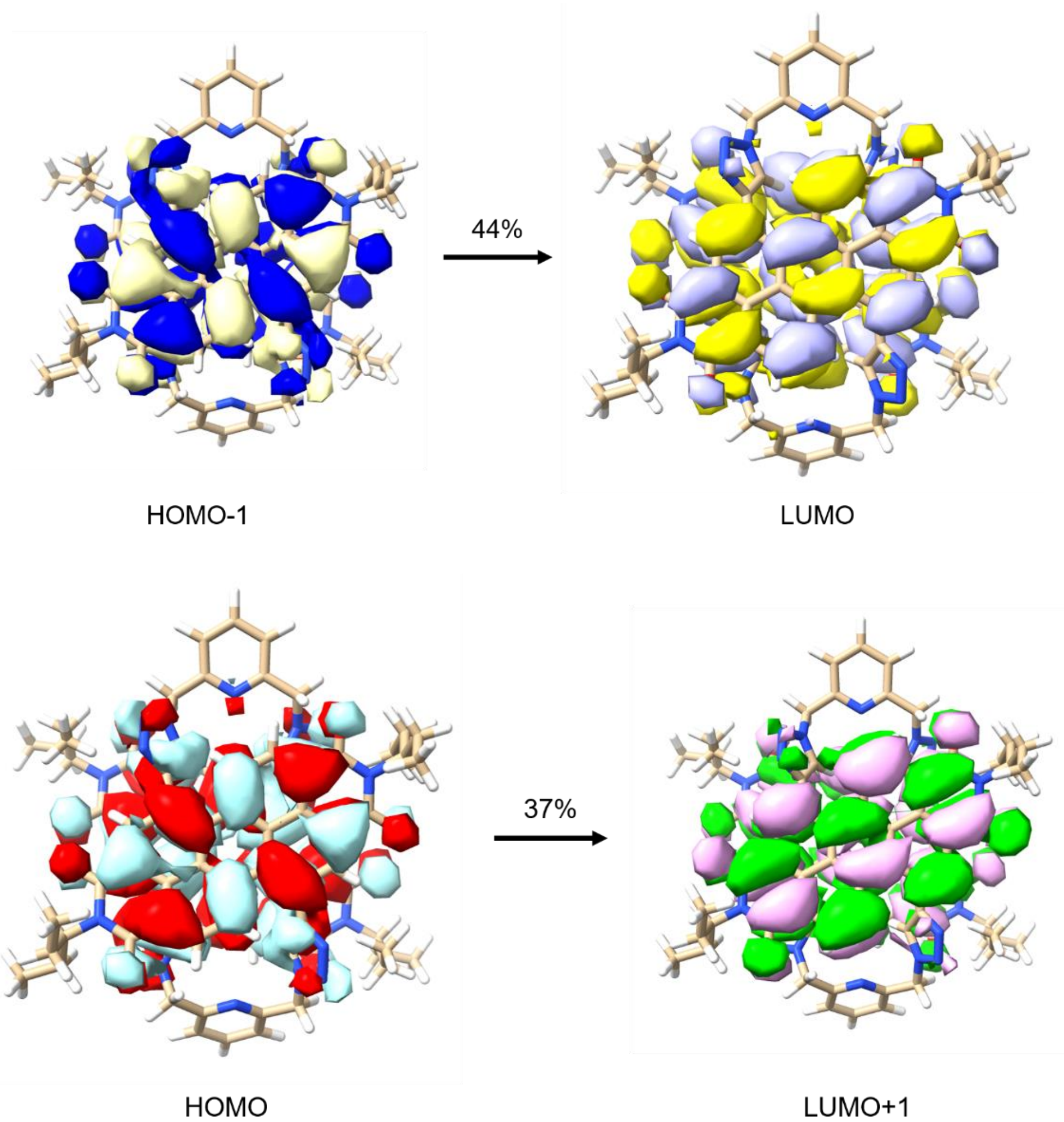

Figure S10.8: Orbital diagram of the two major contributions to transition 2 for **1-*PP***.

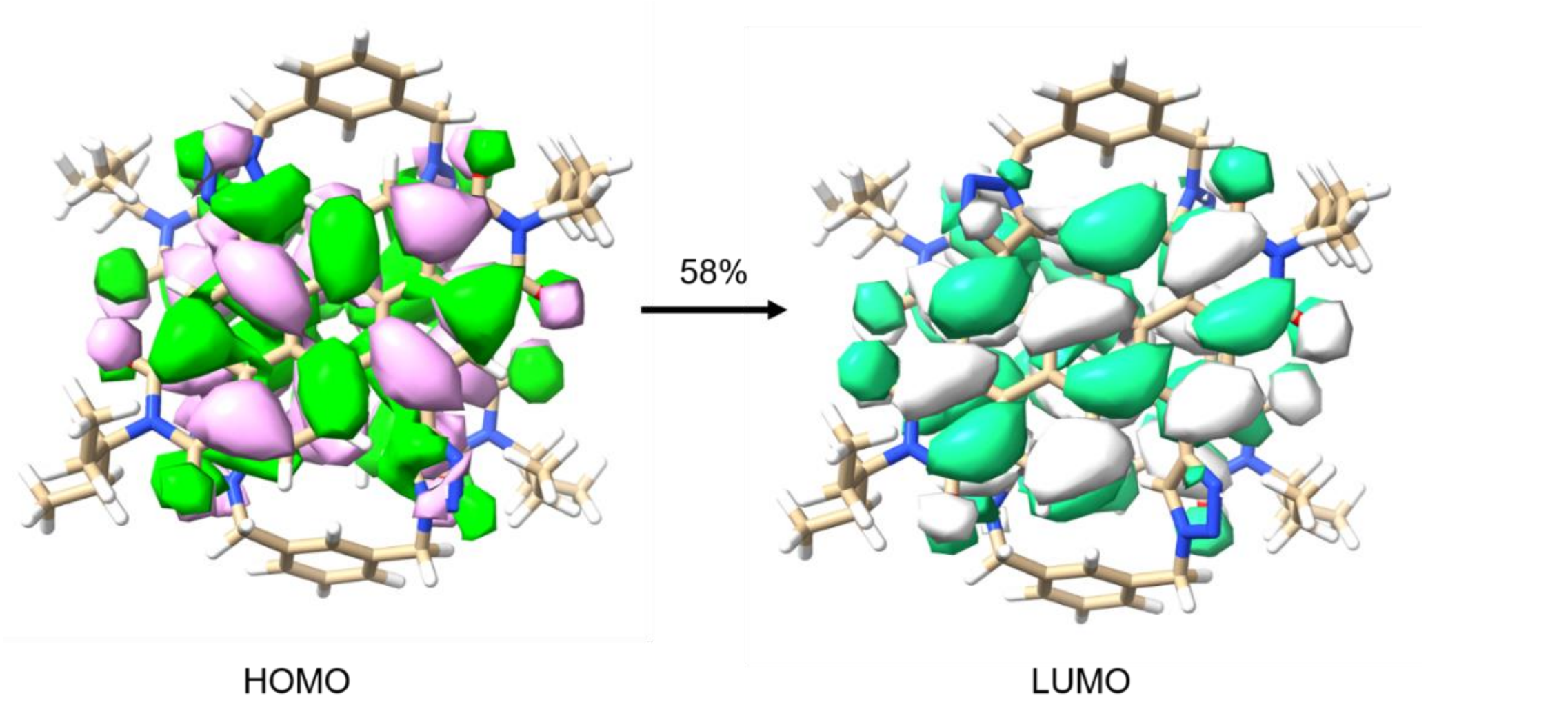

Figure S10.9: Orbital diagram of the major contribution to transition 1 for **2-*PP***. This corresponds to the HOMO-LUMO transition.

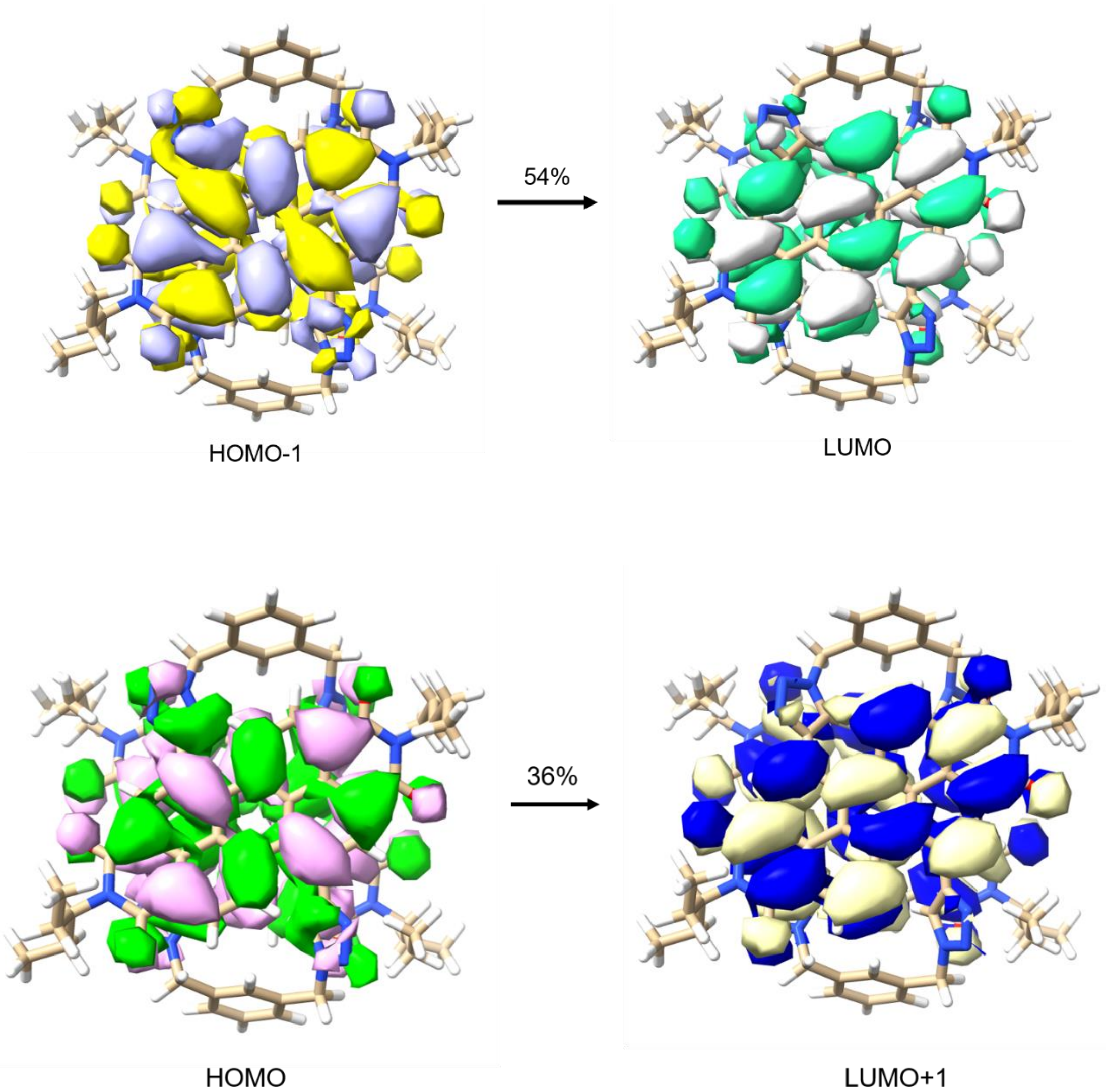

Figure S10.10: Orbital diagram of the two major contributions to transition 2 for **2-*PP***.

Non-covalent interaction analysis

The non-covalent interaction (NCI) index method[60] was used to identify interactions in **1-*PP*** and **2-*PP*** through analysis of the electron density and reduced density gradient.

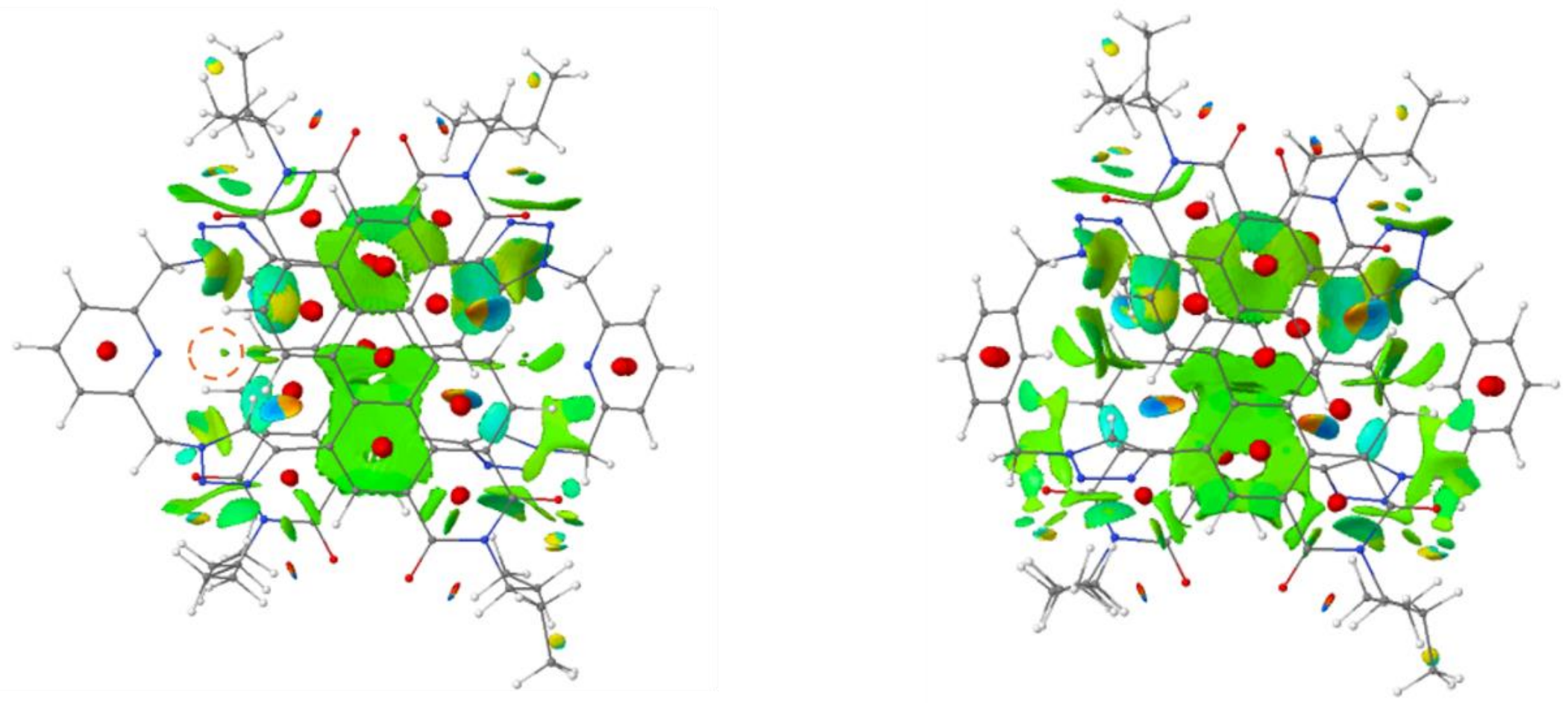

Figure S10.11: 3D representation of the intramolecular NCI regions as isosurfaces (cutoff value (*s*) = 0.25 a.u.) for **1-*PP*** (left) and **2-*PP*** (right). The large green regions between the PDI π-surfaces indicate Van der Waals interactions. Pyridyl–triazole (CH···N) intramolecular hydrogen bonding is highlighted on the **1-*PP*** structure via an orange circle.

The results of the NCI index method are also available to view online under the following links:

1-*PP*: https://lct-webtools.sorbonne-universite.fr/nciweb/to_download/6a4cb7388c7c0/

2-*PP*: https://lct-webtools.sorbonne-universite.fr/nciweb/to_download/6a4cb77527757/

c) Structure obtained from CREST conformer search

Conformer Prediction for 2-*MM*/*PP*

In the absence of a crystal structure, possible conformers of **2-*MM*/*PP*** were also predicted using a combination of the CREST[61] code and the GFN2-xTB semiempirical tight-binding method[62]. The branched alkyl chains at the imide positions were simplified to methyl groups. The resulting lowest energy structure was then optimised by means of DFT using the B97-3c functional[63] and the def2-TZVP basis set.[59] The .xyz file "2-PP_fromCREST_DFTOpt" is available in the folder "DFT_structure".

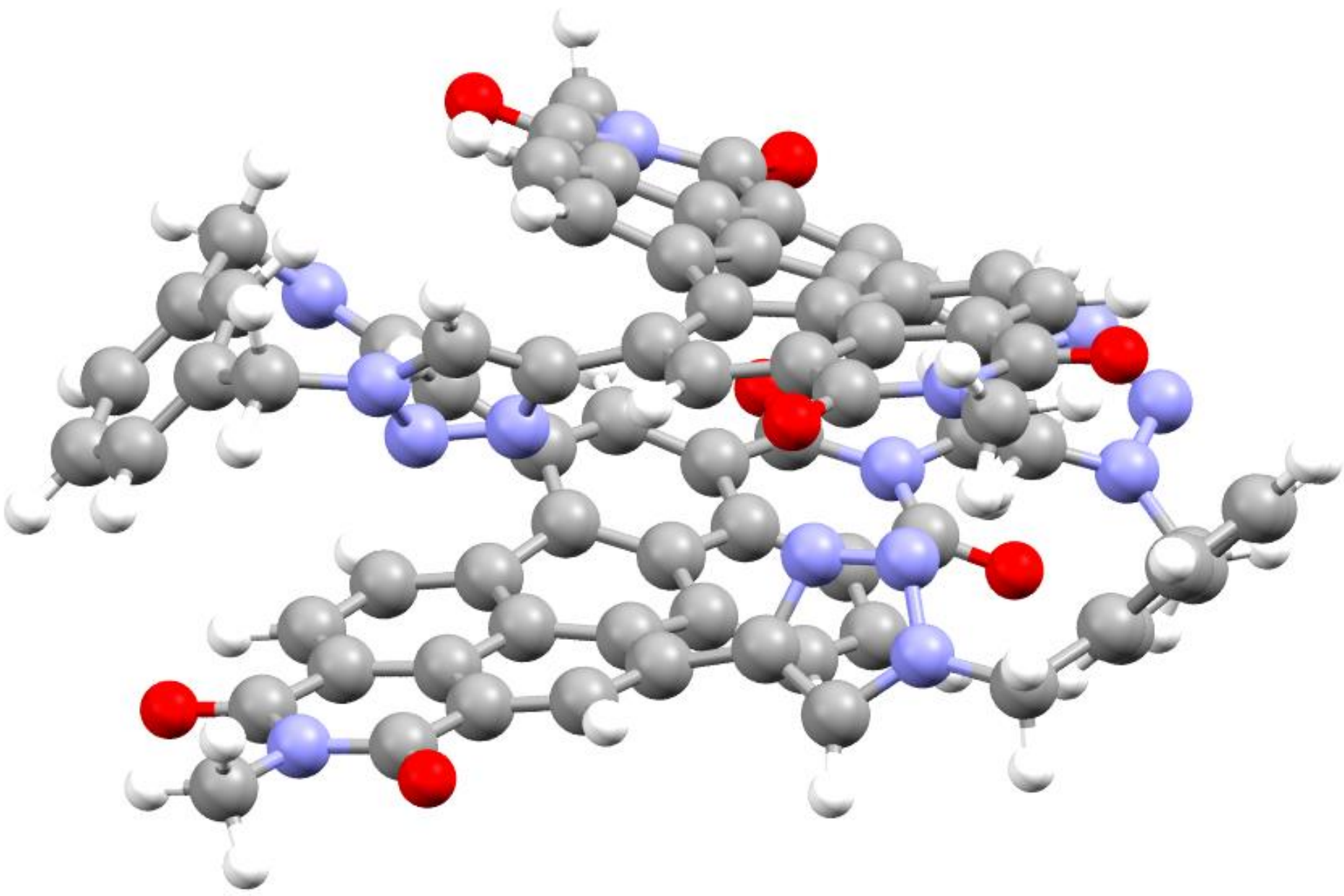

Figure S10.12: Lowest energy conformer of **2-*MM/PP*** as obtained by CREST and optimised using DFT (B97-3c/def2-TZVP). The ***PP*** enantiomer is shown.

Spectral Prediction for 2-*PP*

The spectra of **2-*PP*** were predicted from the DFT-optimised conformer by means of TD-DFT using the TD-ωB97x functional [58] and the def2-SVP basis-set [59].

Table S10.5: Predicted vertical transitions and their oscillator and rotatory strengths (R) of **2-*PP***.

Transition	λ (nm)	f_{osc} (μ^2)	R (10^{-40} erg cm^3)
**1**	503.4	0.129872	662.8844
**2**	476.4	0.628342	-588.421
**3**	359	0.008203	23.56598
**4**	356.8	0.022918	-97.5296
**5**	338.1	0.0151	-58.6309
**6**	332.6	0.029779	194.4134
**7**	321.7	0.00046	-23.8097
**8**	320.6	0.052116	-111.951
**9**	314.3	0.013173	73.7362
**10**	313	0.142145	-99.087

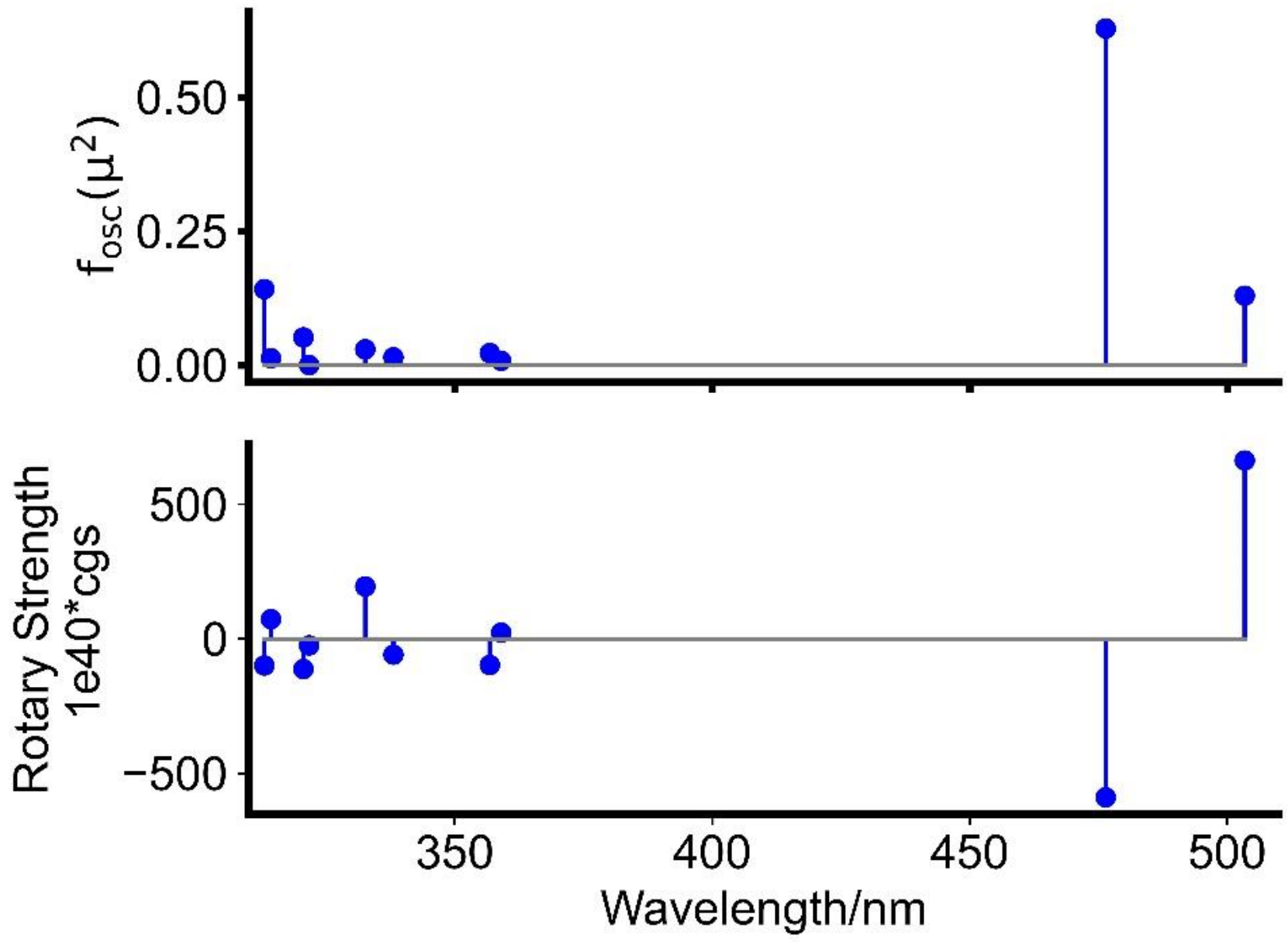

Figure S10.13: Predicted UV-vis and CD spectra for **2-*PP***

11. Excitonic coupling calculations

The excitonic coupling strength for **1-*MM*/*PP*** and **1-*MP*** in toluene were determined from their steady-state absorption spectra, using the Huang-Rhys factor $\lambda^2{}_{\mathrm{mon}}$ of the acyclic PDI monomer **5** as reference[64–66]. $\lambda^2{}_{\mathrm{mon}}$ is defined as:

$$\lambda^2{}_{mon} = (R_{0-1}/R_{0-0}) \tag{S1}$$

Where R_{0-0} and R_{0-1} are the intensities of the 0-0 and 0-1 vibronic bands of its steady-state absorption spectrum. $(\mathrm{R}_{0-1}/\mathrm{R}_{0-0}) = 0.8$ for the acyclic PDI monomer. Equation S1 can then substituted into Equation S2 which gives the total excitonic coupling (i.e. Coulombic + charge transfer) when solved for $\mathrm{J}_{\mathrm{Tot}}$:

$$R_{abs} = (\lambda^2{}_{mon})^{-1}\left[\frac{1-G(0,\lambda^2)e^{-\lambda^2{}_{mon}}J_{Tot}/\omega_0}{1-G(1,\lambda^2)e^{-\lambda^2{}_{mon}}J_{Tot}/\omega_0}\right]^2 \tag{S2}$$

Where $\mathrm{R}_{\mathrm{abs}} = (\lambda^2{}_{\mathrm{dim}})^{-1} = (\mathrm{R}_{0-0}/\mathrm{R}_{0-1})$ of **1-*MM*/*PP*** or **1-*MP*** in toluene and ω_0 is the spacing of the vibronic progression (1350 cm^{-1}). Finally, G is the vibrational function, as defined in Equation S3:

$$G(v_t;\lambda^2) = \sum_{\substack{u=0,1,..\\ u\neq v_t}} \frac{(\lambda^2)^u}{u!(u-v_t)}, v_t \in \mathbb{N}_0 \tag{S3}$$

The values of $\mathrm{J}_{\mathrm{Tot}}$ extracted using the method outlined above are reported in the main manuscript.

12. NMR spectra

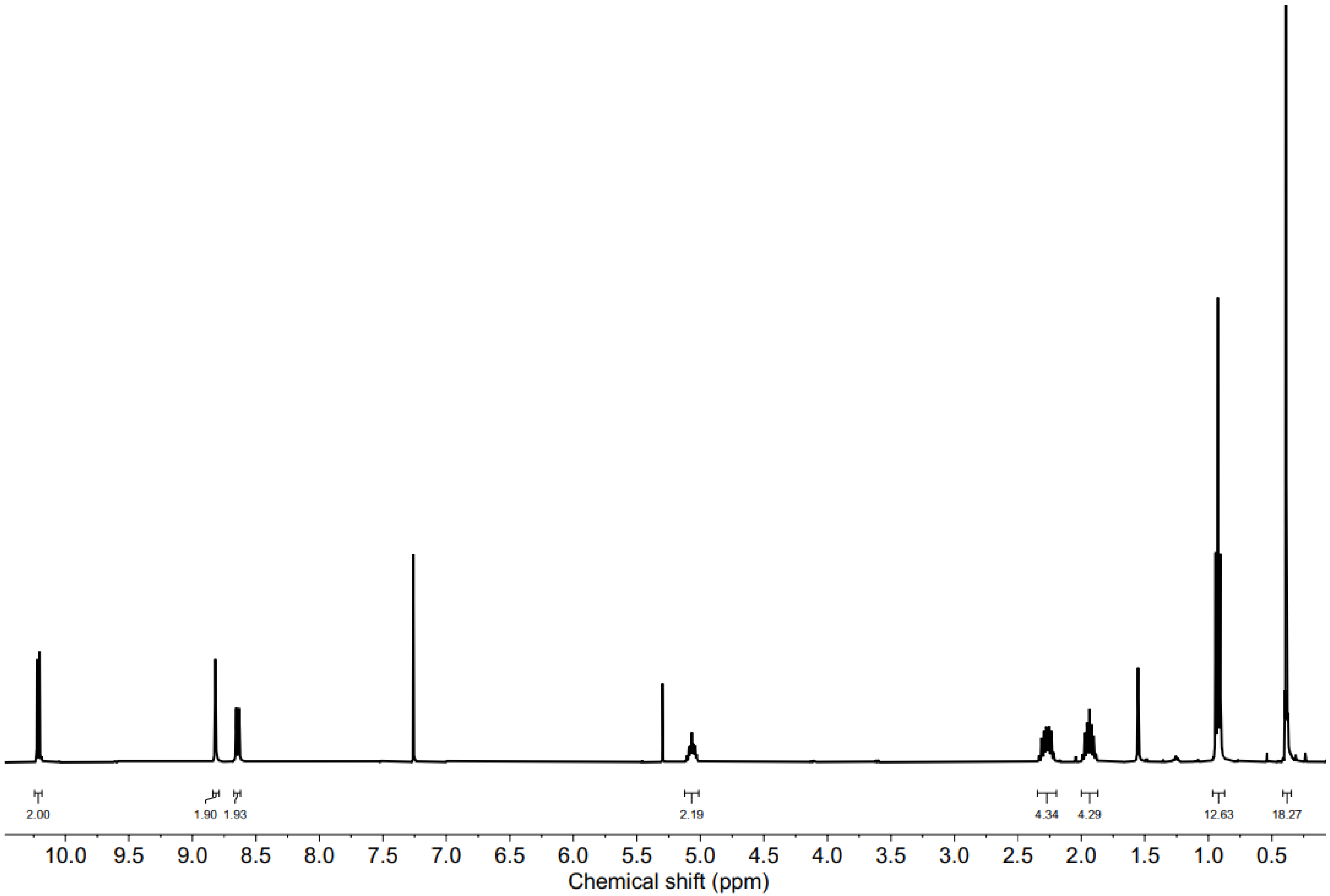

^{1}H NMR spectrum of **4** (Chloroform-*d*, 298 K, 400 MHz)

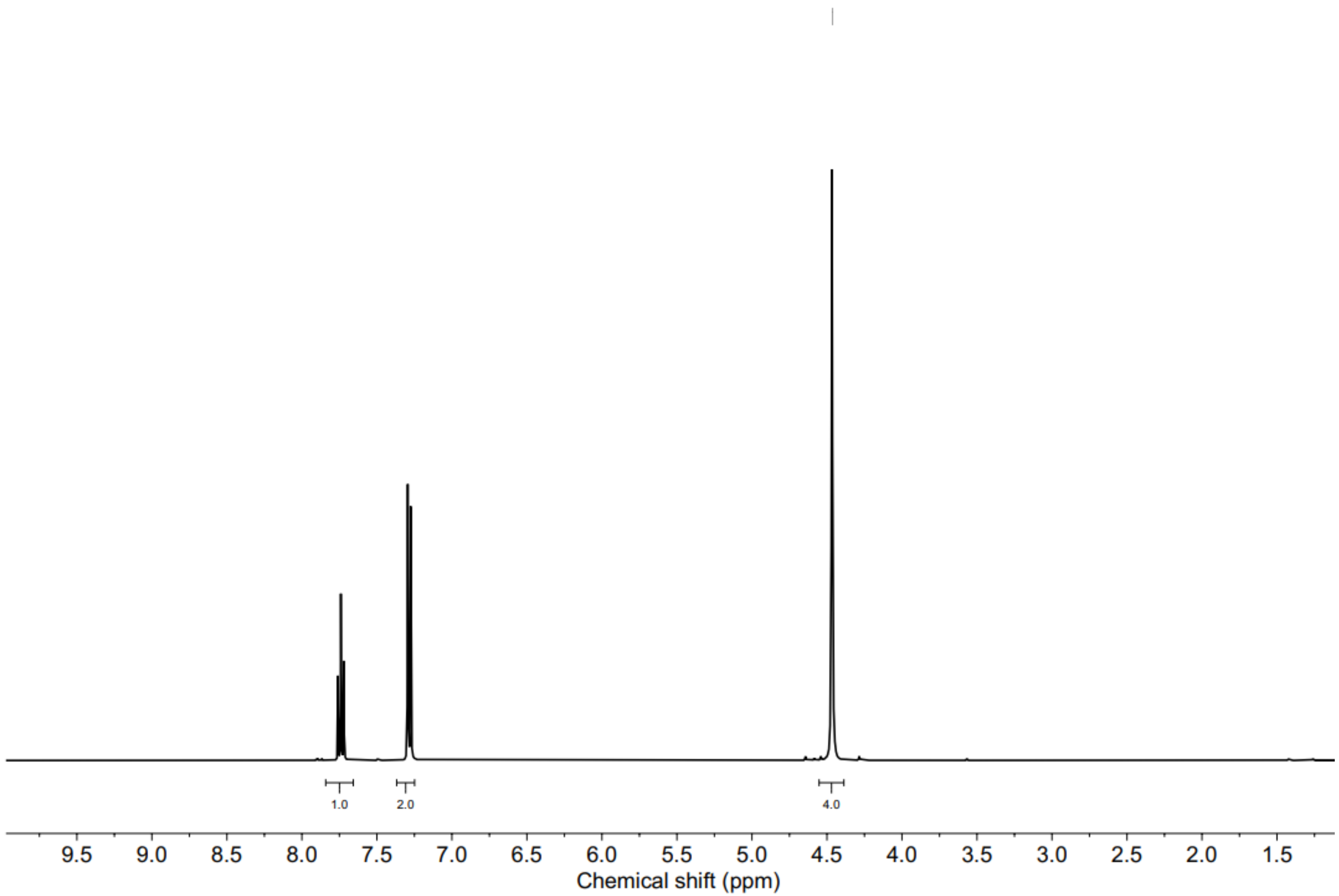

^{1}H NMR spectrum of **7** (Chloroform-*d*, 298 K, 400 MHz)

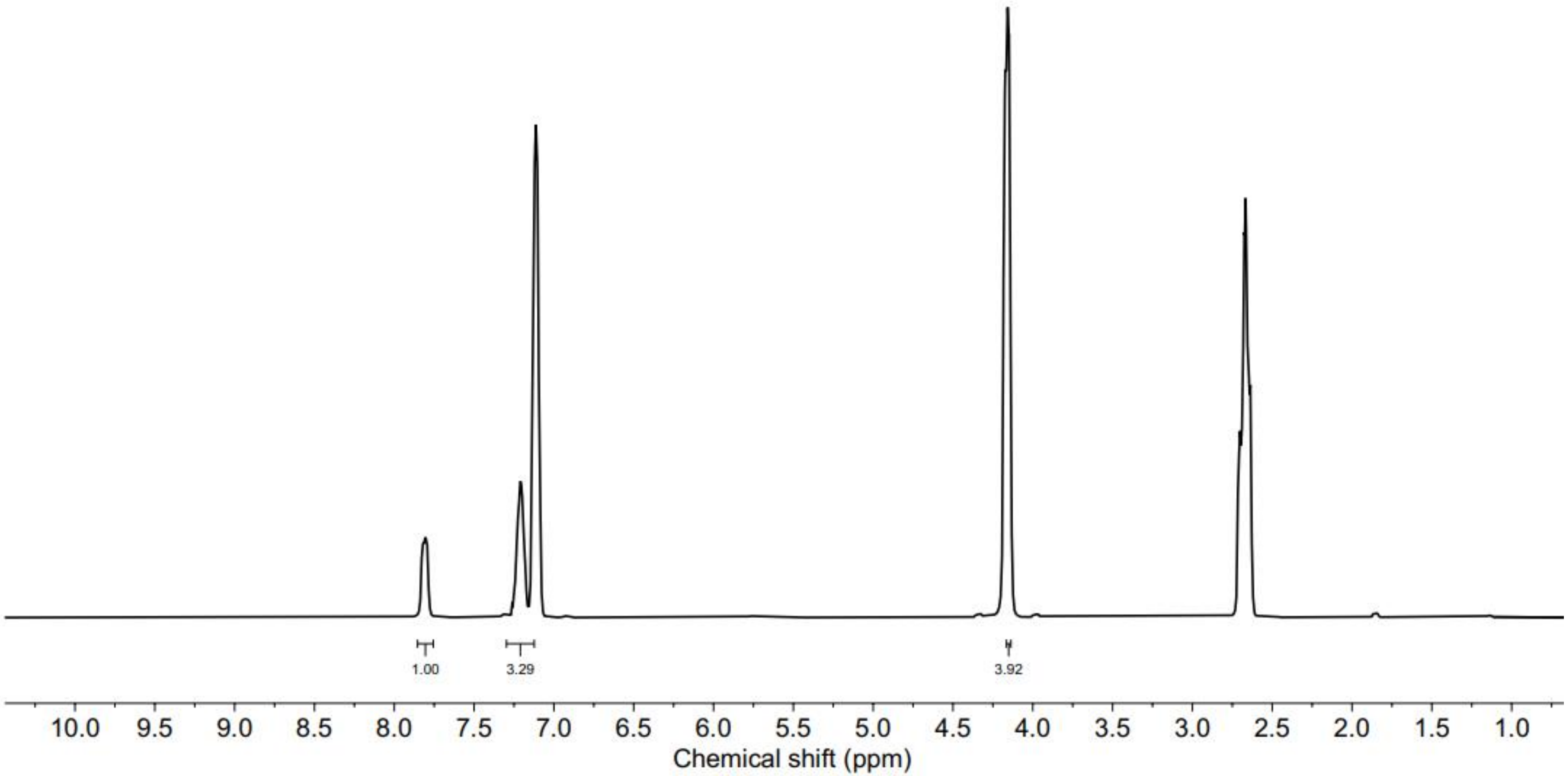

^{1}H NMR spectrum of **8** (Chloroform-*d*, 298 K, 400 MHz)

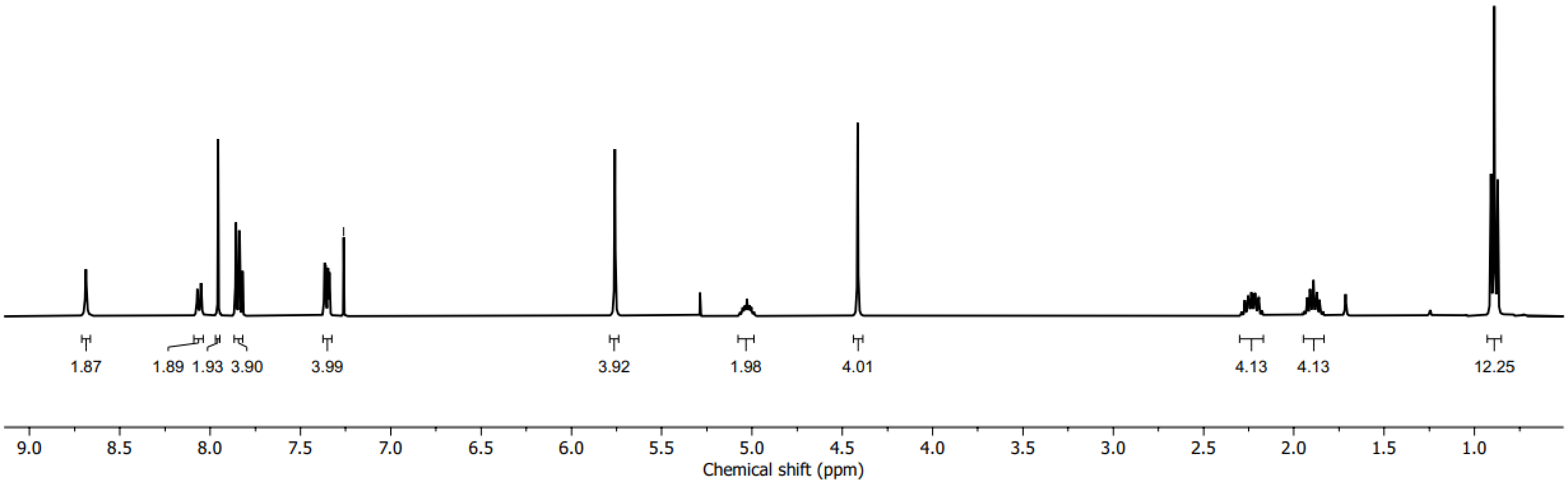

^{1}H NMR spectrum of **5** (Chloroform-*d*, 298 K, 400 MHz)

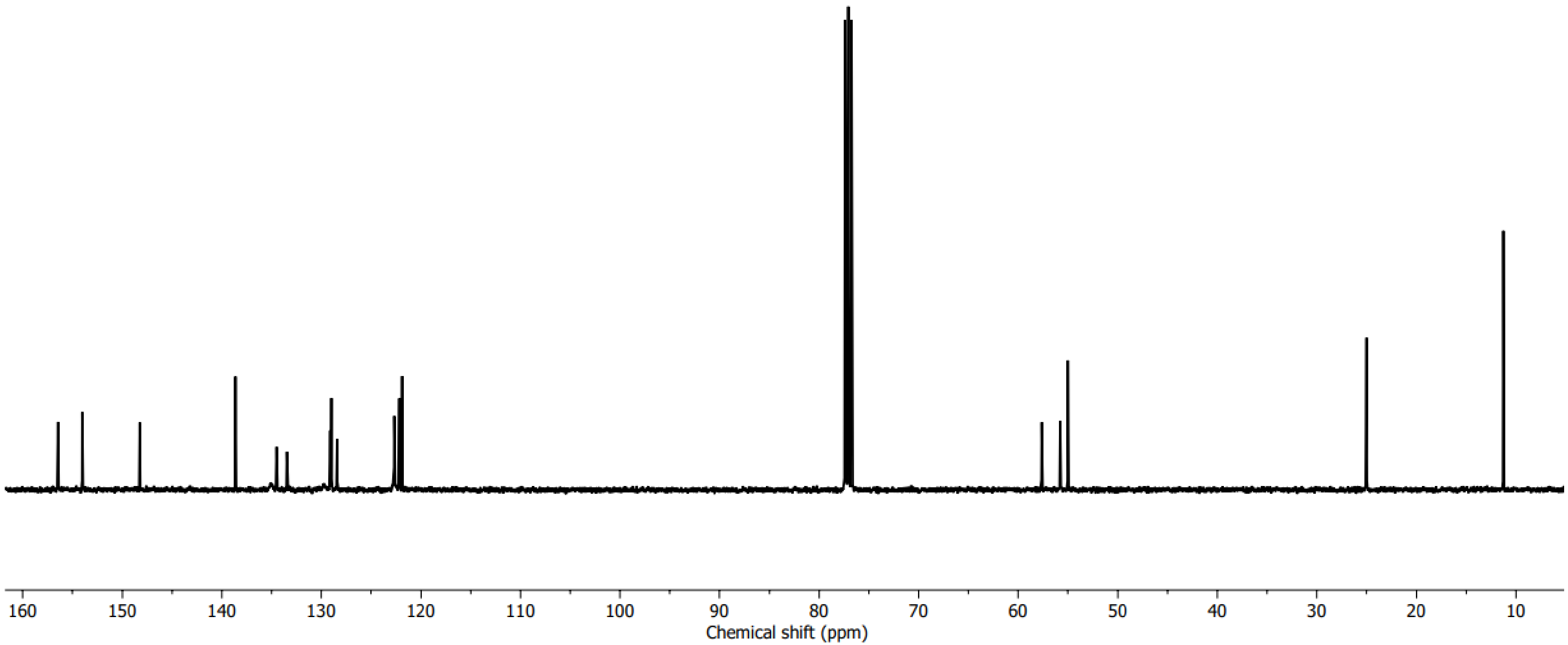

^{13}C NMR spectrum of **5** (Chloroform-*d*, 298 K, 101 MHz)

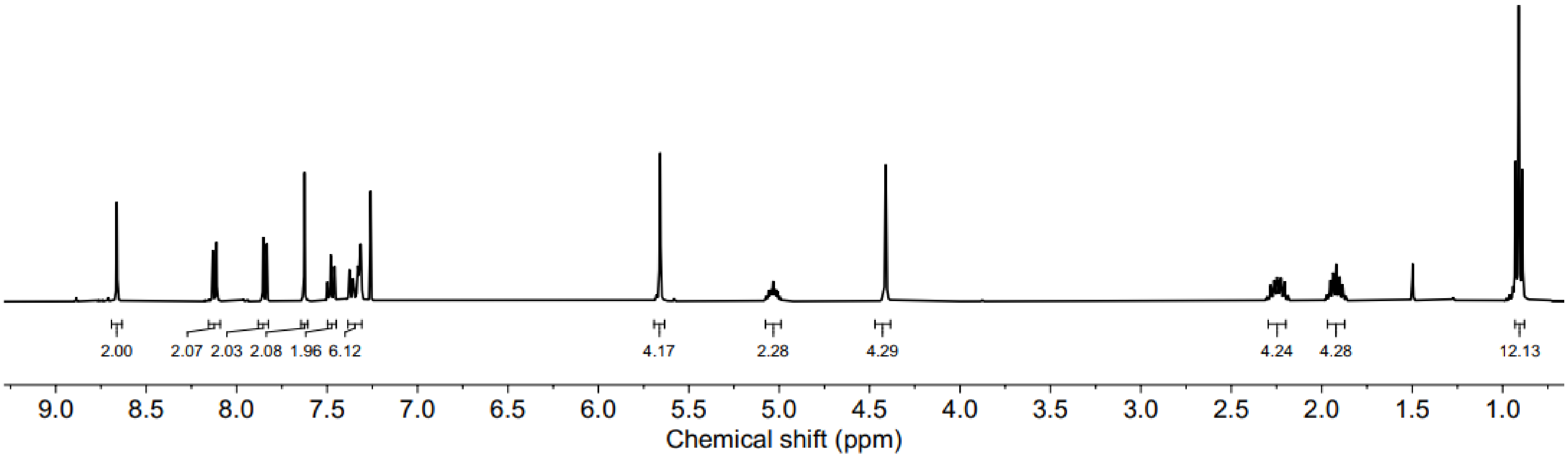

^{1}H NMR spectrum of **6** (Chloroform-*d*, 298 K, 400 MHz)

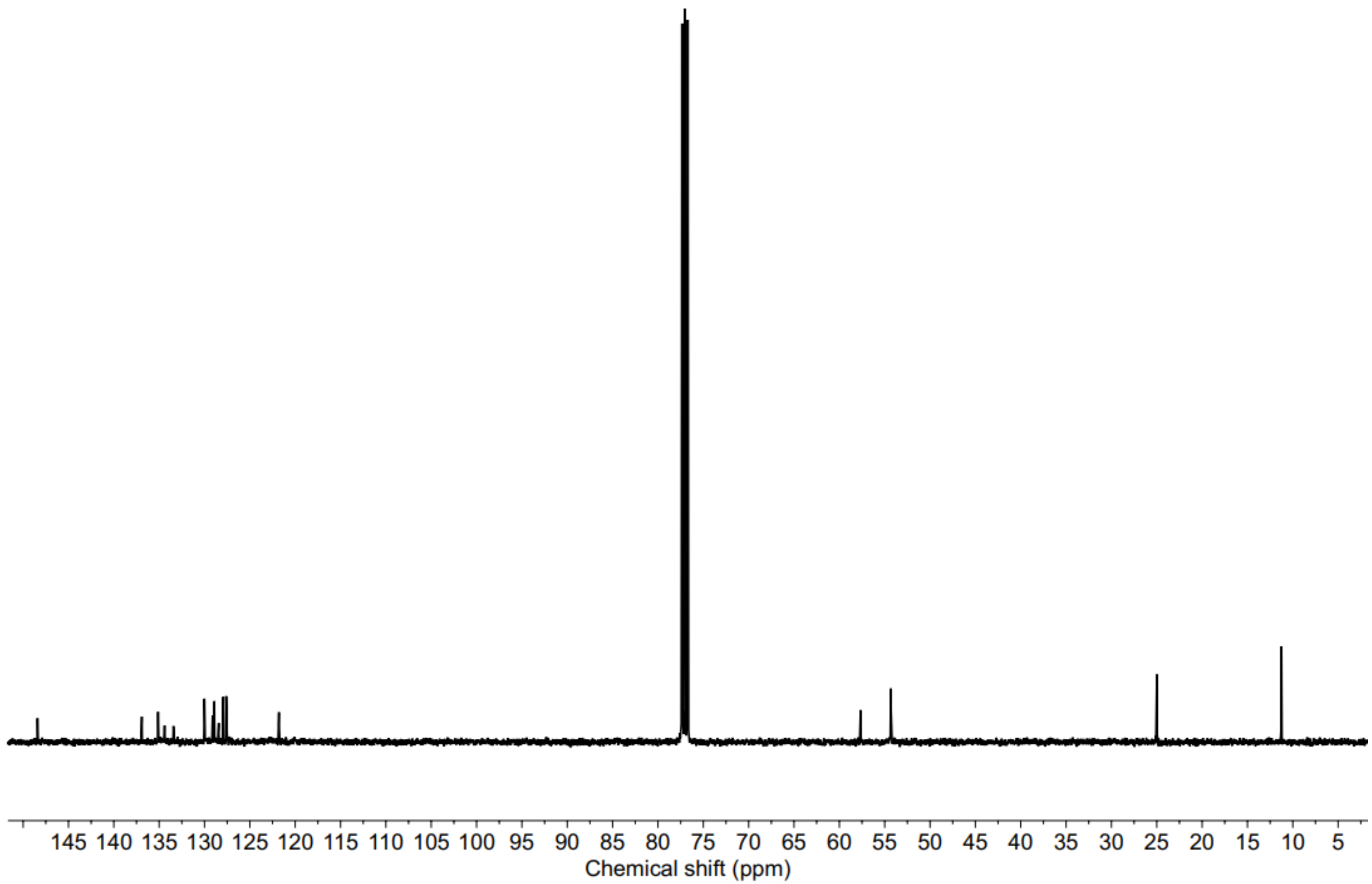

^{13}C NMR spectrum of **6** (Chloroform-*d*, 298 K, 101 MHz)

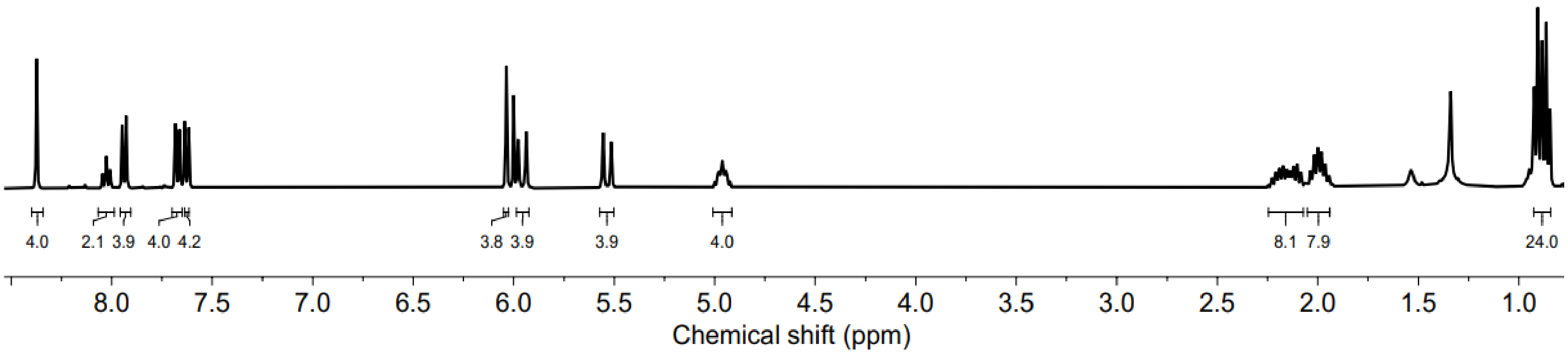

^{1}H NMR of **1-*MM/PP*** (TCE-d_2, 373K, 400 MHz)

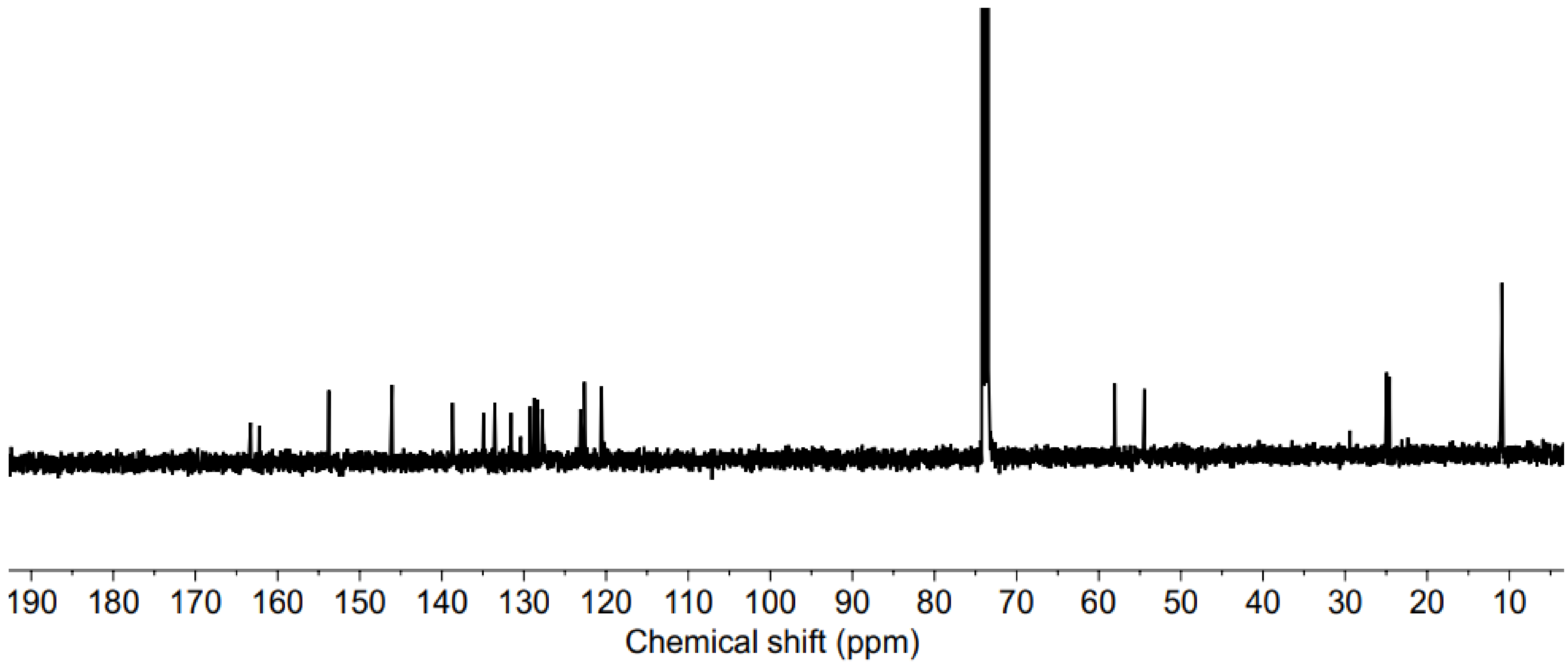

^{13}C NMR of **1-*MM/PP*** (TCE-d_2, 373K, 101 MHz)

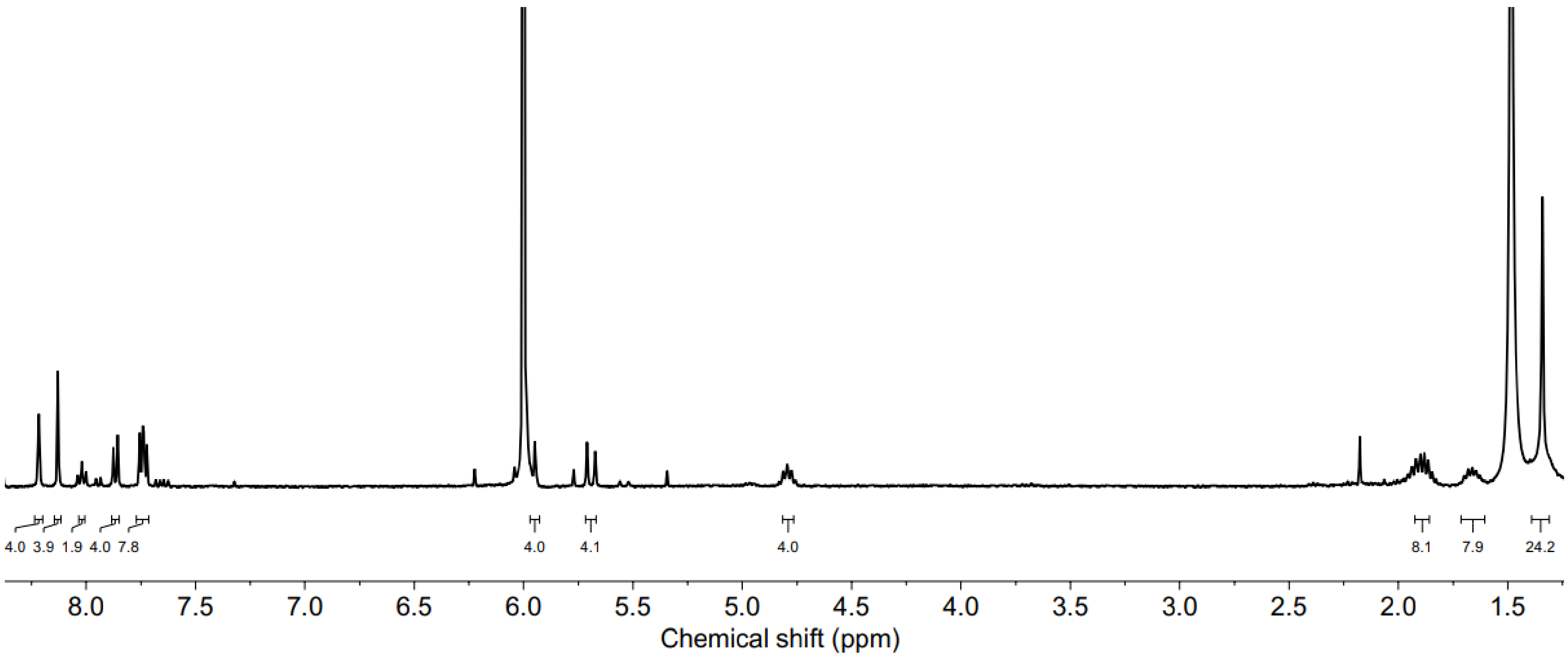

^{1}H NMR of **1-*MP*** (TCE-d_2, 373 K, 400 MHz)

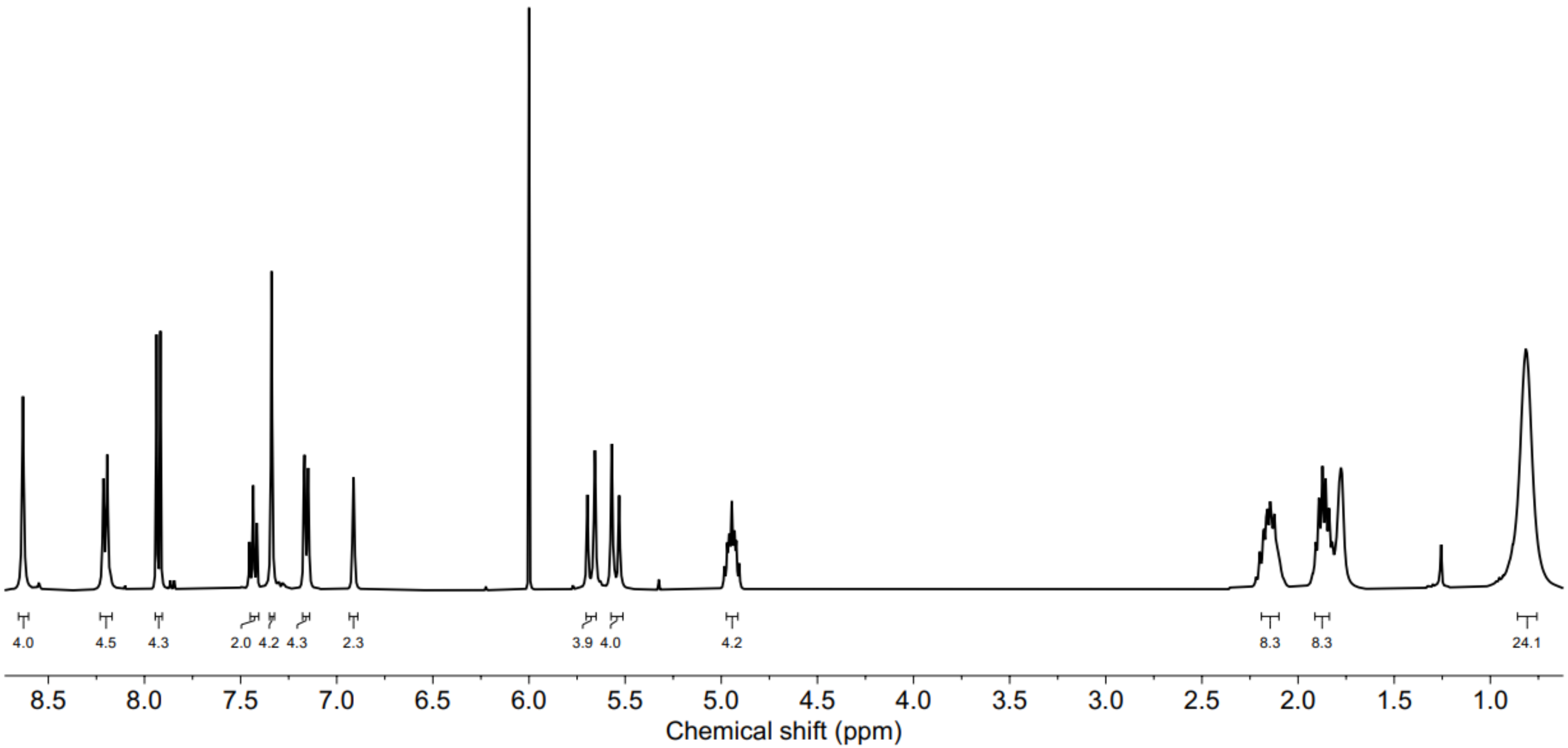

^{1}H NMR of **2-*MM/PP*** (TCE-d_2, 373 K, 400 MHz)

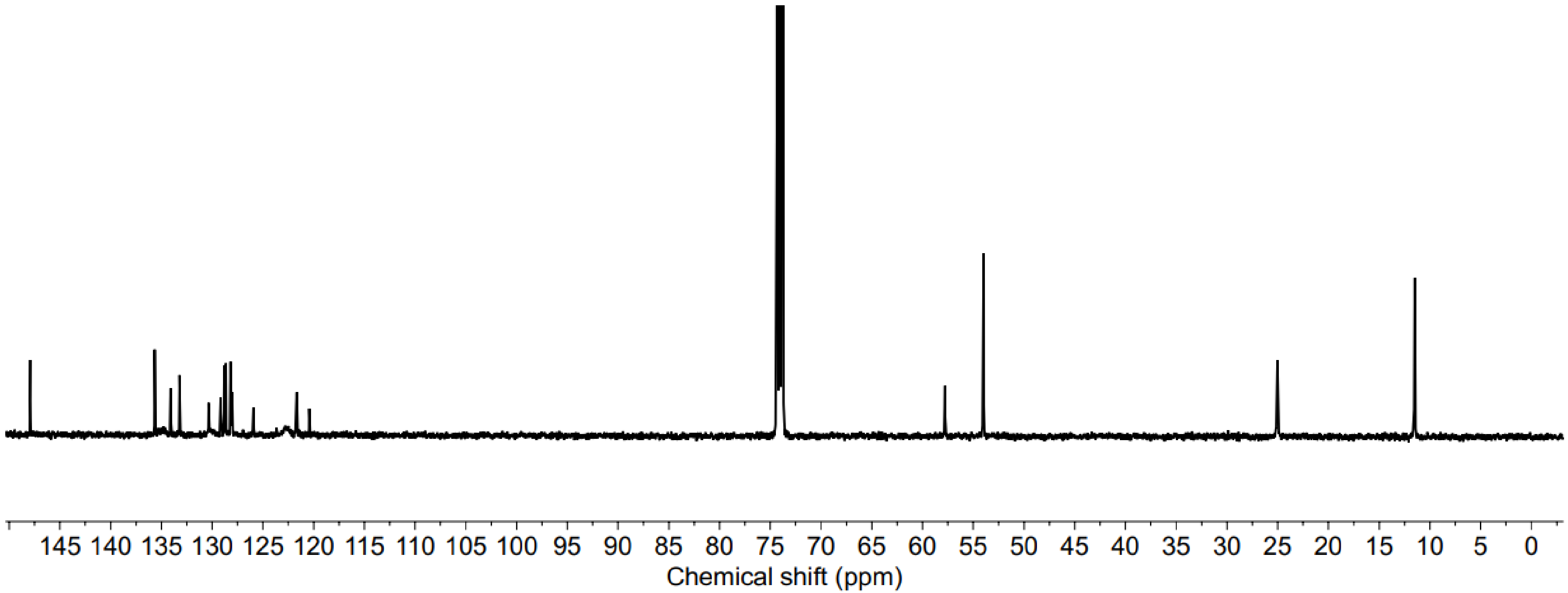

^{13}C NMR of **2-*MM/PP*** (TCE-d_2, 373 K, 101 MHz)

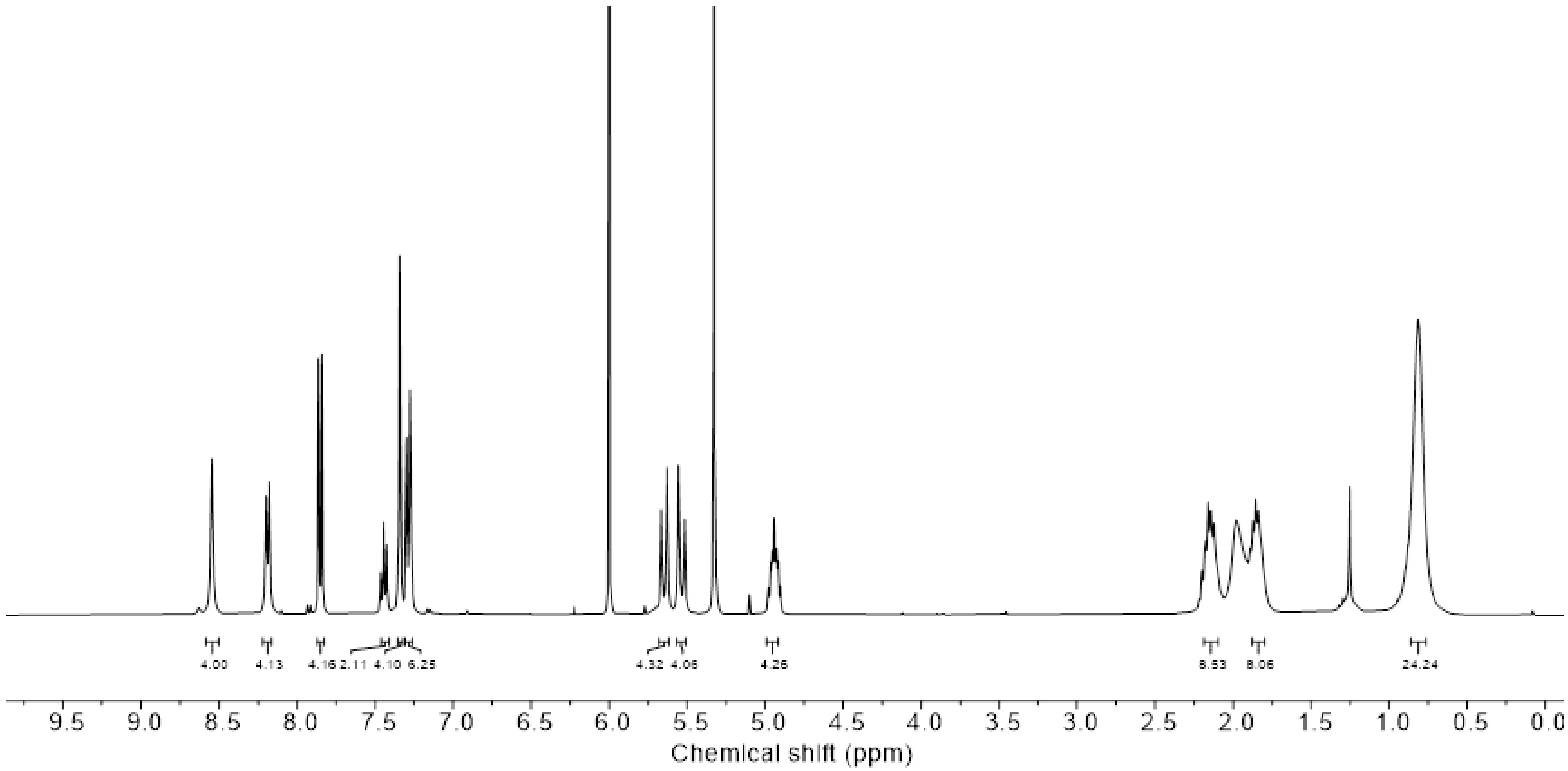

^{1}**H NMR** of **2-*M/P*** (TCE-d_2, 323 K, 400 MHz)

13. MALDI spectra

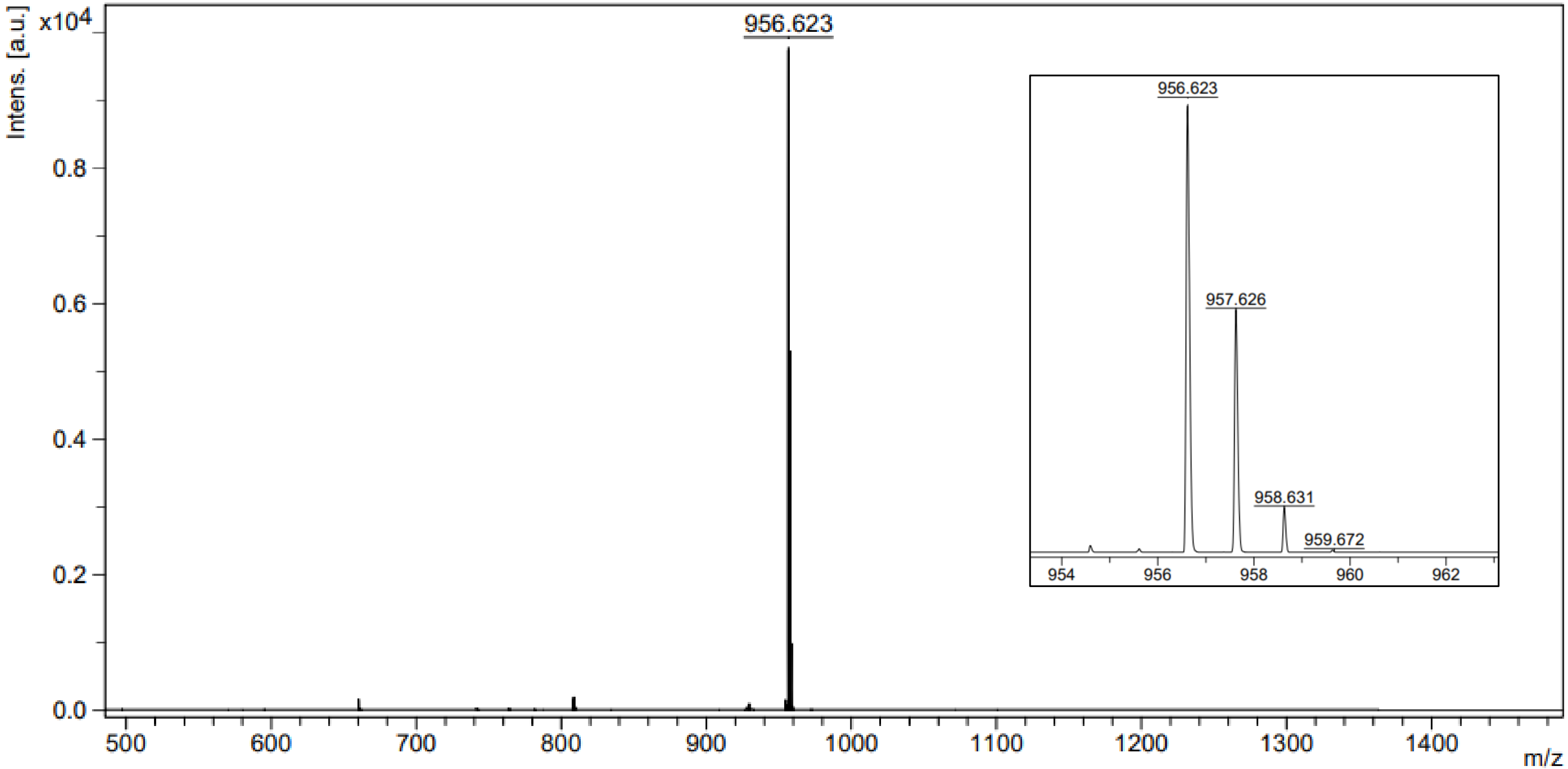

Observed MALDI (TOF) mass spectrum of **5**.

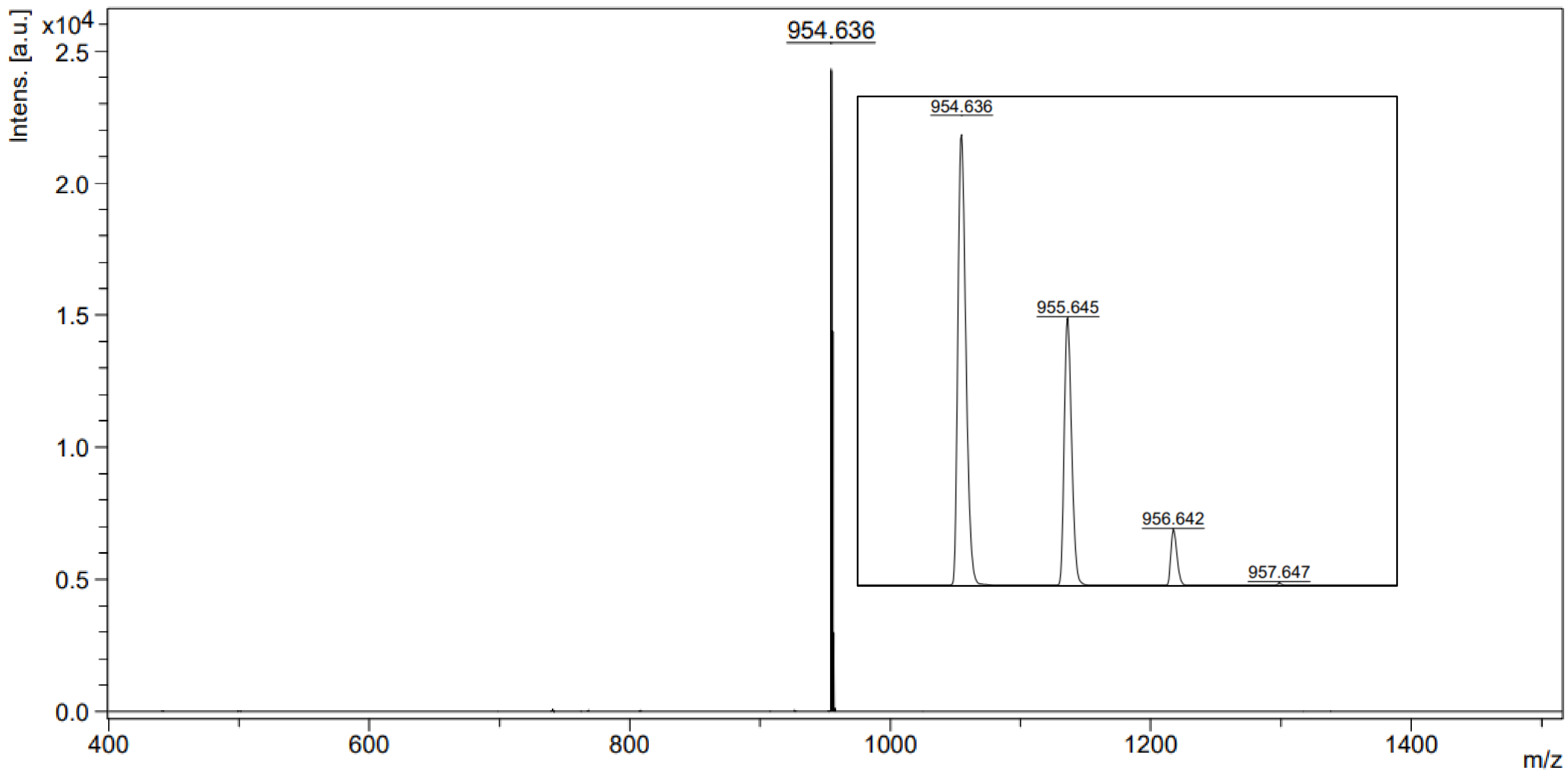

Observed MALDI (TOF) mass spectrum of **6**.

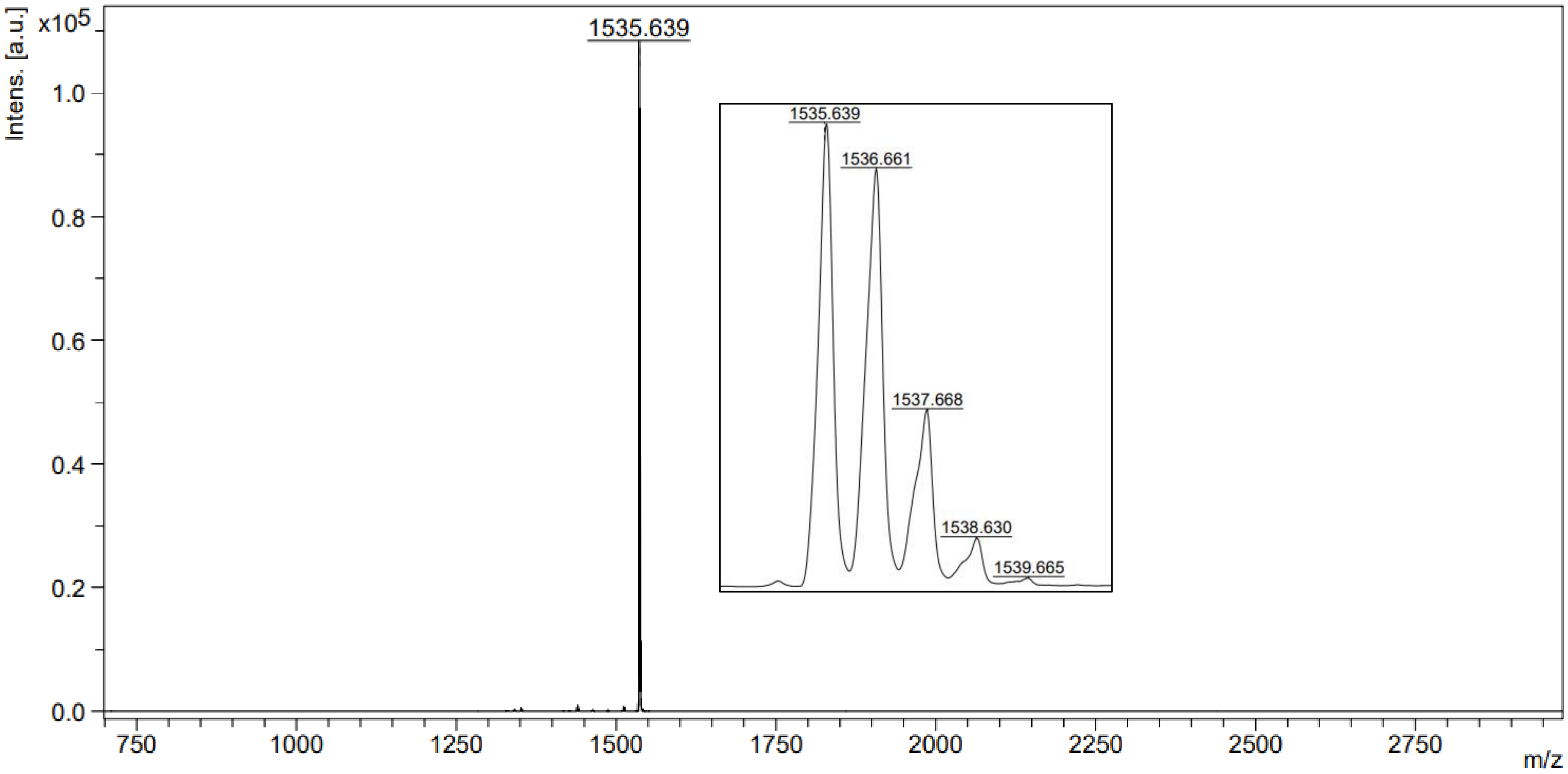

Observed MALDI (TOF) mass spectrum of **1-*MM/PP/MP***.

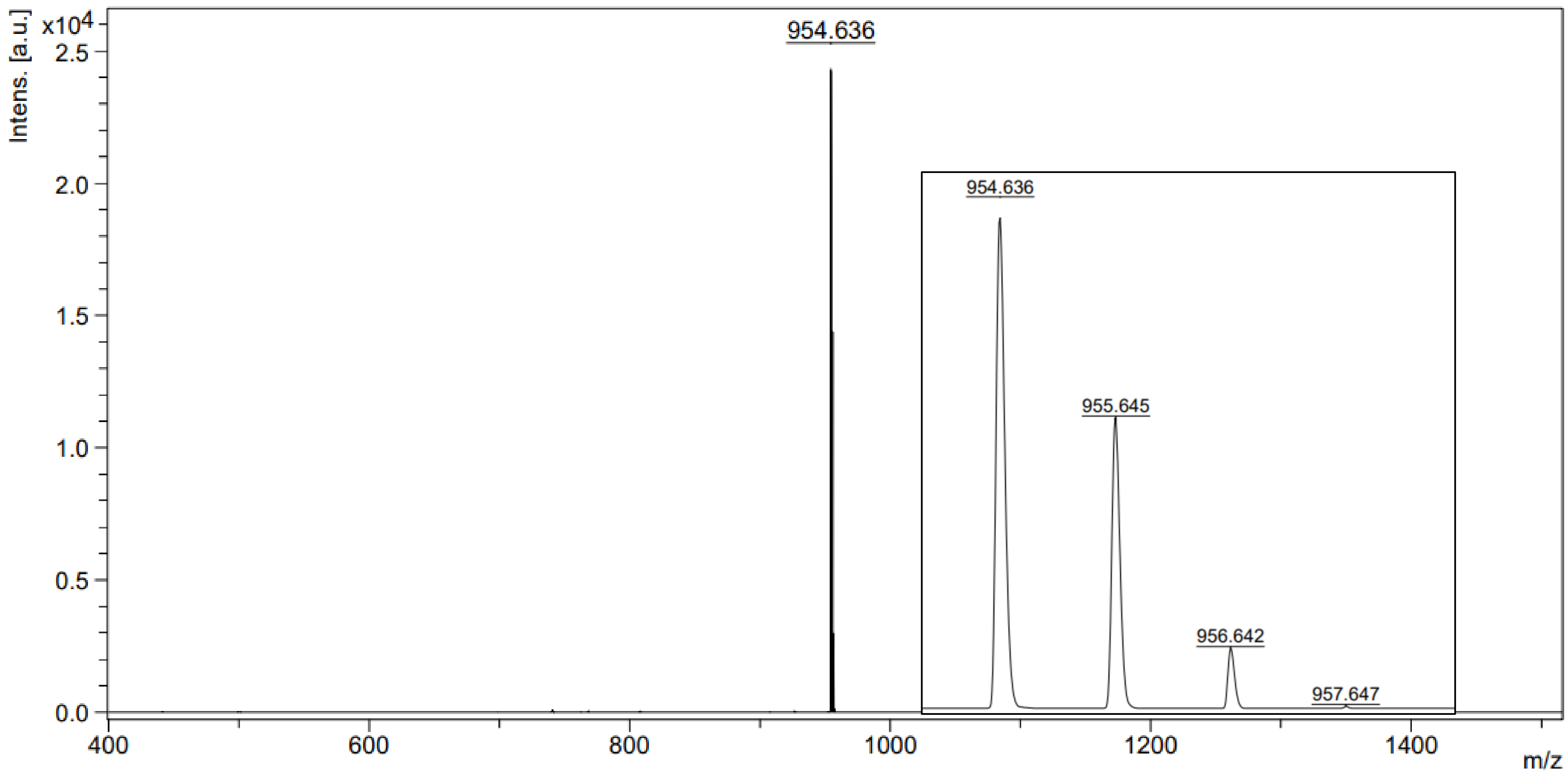

Observed MALDI (TOF) mass spectrum of ***2-MM/PP/MP***.

14. References

(1) Chan, T. R.; Hilgraf, R.; Sharpless, K. B.; Fokin, V. V. Polytriazoles as Copper(I)-Stabilizing Ligands in Catalysis. *Org. Lett.* **2004**, *6* (17), 2853–2855. https://doi.org/10.1021/ol0493094.

(2) Schmidt, C. D.; Lang, N.; Jux, N.; Hirsch, A. A Facile Route to Water-Soluble Coronenes and Benzo[*ghi*]Perylenes. *Chemistry – A European Journal* **2011**, *17* (19), 5289–5299. https://doi.org/10.1002/chem.201003232.

(3) Rajasingh, P.; Cohen, R.; Shirman, E.; Shimon, L. J. W.; Rybtchinski, B. Selective Bromination of Perylene Diimides under Mild Conditions. *J. Org. Chem.* **2007**, *72* (16), 5973–5979. https://doi.org/10.1021/jo070367n.

(4) Hartmann, D.; Penty, S. E.; Pal, R.; Barendt, T. A. Chirally Locked and Dynamic Bis-Perylene Diimide Macrocycles with Multiple Sources of Chirality. *Commun. Chem* **2026**, *9* (1), 102. https://doi.org/10.1038/s42004-026-01904-z.

(5) Magallón, C.; Planas, O.; Roldán-Gómez, S.; Luis, J. M.; Company, A.; Ribas, X. Well-Defined Aryl-FeII Complexes in Cross-Coupling and C–H Activation Processes. *Organometallics* **2021**, *40* (9), 1195–1200. https://doi.org/10.1021/acs.organomet.1c00100.

(6) Shao, C.; Grüne, M.; Stolte, M.; Würthner, F. Perylene Bisimide Dimer Aggregates: Fundamental Insights into Self-Assembly by NMR and UV/Vis Spectroscopy. *Chemistry – A European Journal* **2012**, *18* (43), 13665–13677. https://doi.org/10.1002/chem.201201661.

(7) Penty, S. E.; Zwijnenburg, M. A.; Orton, G. R. F.; Stachelek, P.; Pal, R.; Xie, Y.; Griffin, S. L.; Barendt, T. A. The Pink Box: Exclusive Homochiral Aromatic Stacking in a Bis-Perylene Diimide Macrocycle. *J. Am. Chem. Soc.* **2022**, *144* (27), 12290–12298. https://doi.org/10.1021/jacs.2c03531.

(8) *21st Century Challenges in Chemical Crystallography I: History and Technical Developments*; Mingos, D. M. P., Raithby, P. R., Eds.; Structure and Bonding; Springer International Publishing: Cham, **2020**; Vol. 185. https://doi.org/10.1007/978-3-030-64743-8.

(9) Dolomanov, O. V.; Bourhis, L. J.; Gildea, R. J.; Howard, J. A. K.; Puschmann, H. *OLEX2*: A Complete Structure Solution, Refinement and Analysis Program. *J Appl Crystallogr* **2009**, *42* (2), 339–341. https://doi.org/10.1107/S0021889808042726.

(10) Winter, G.; Waterman, D. G.; Parkhurst, J. M.; Brewster, A. S.; Gildea, R. J.; Gerstel, M.; Fuentes-Montero, L.; Vollmar, M.; Michels-Clark, T.; Young, I. D.; Sauter, N. K.; Evans, G. *DIALS*: Implementation and Evaluation of a New Integration Package. *Acta Crystallogr D Struct Biol* **2018**, *74* (2), 85–97. https://doi.org/10.1107/S2059798317017235.

(11) Beilsten-Edmands, J.; Winter, G.; Gildea, R.; Parkhurst, J.; Waterman, D.; Evans, G. Scaling Diffraction Data in the *DIALS* Software Package: Algorithms and New Approaches for Multi-Crystal Scaling. *Acta Crystallogr D Struct Biol* **2020**, *76* (4), 385–399. https://doi.org/10.1107/S2059798320003198.

(12) Winter, G. *Xia2*: An Expert System for Macromolecular Crystallography Data Reduction. *J Appl Crystallogr* **2010**, *43* (1), 186–190. https://doi.org/10.1107/S0021889809045701.

(13) Bolte, M. *TWINLAW* and *HKLF* 5: Two Programs for the Handling of Non-Merohedral Twins. *J Appl Crystallogr* **2004**, *37* (1), 162–165. https://doi.org/10.1107/S0021889803027146.

(14) Ugras, T. J.; Yao, Y.; Robinson, R. D. Can We Still Measure Circular Dichroism with Circular Dichroism Spectrometers: The Dangers of Anisotropic Artifacts. *Chirality* **2023**, *35* (11), 846–855. https://doi.org/10.1002/chir.23597.

(15) Lamnawar, K.; Zhang, H.; Maazouz, A. Coextrusion of Multilayer Structures, Interfacial Phenomena. In *Encyclopedia of Polymer Science and Technology*; Mark, H. F., Ed.; Wiley, **2013**. https://doi.org/10.1002/0471440264.pst584.

(16) Hussain, R.; Jávorfi, T.; Siligardi, G. CD Imaging at High Spatial Resolution at Diamond B23 Beamline: Evolution and Applications. *Front. Chem.* **2021**, *9*, 616928. https://doi.org/10.3389/fchem.2021.616928.

(17) Martin, A. T.; Nichols, S. M.; Murphy, V. L.; Kahr, B. Chiroptical Anisotropy of Crystals and Molecules. *Chem. Commun.* **2021**, *57* (66), 8107–8120. https://doi.org/10.1039/D1CC00991E.

(18) Hussain, R.; Krumpa, N.; Strachan, J.; Clarke, D.; Wagner, U.; Macdonald, B.; Reading, D.; Cobb, T.; Gillingham, I.; Price, A.; Smith, M.; Siligardi, G. Design of B23 Circular Dichroism Beamline at Diamond Light Source. *Adv. Syn. Rad.* **2008**, *01* (02), 265–270. https://doi.org/10.1142/S1793617908000173.

(19) Gedeon, C.; Del Rio, N.; Furlan, F.; Taddeucci, A.; Vanthuyne, N.; Gregoriou, V. G.; Fuchter, M. J.; Siligardi, G.; Gasparini, N.; Crassous, J.; Chochos, C. L. Rational Design of New Conjugated Polymers with Main Chain Chirality for Efficient Optoelectronic Devices: Carbo[6]Helicene and

Indacenodithiophene Copolymers as Model Compounds. *Advanced Materials* **2024**, *36* (25), 2314337. https://doi.org/10.1002/adma.202314337.
(20) Chowdhury, R.; Preuss, M. D.; Cho, H.-H.; Thompson, J. J. P.; Sen, S.; K. Baikie, T.; Ghosh, P.; Boeije, Y.; Chua, X. W.; Chang, K.-W.; Guo, E.; Van Der Tol, J.; Van Den Bersselaar, B. W. L.; Taddeucci, A.; Daub, N.; Dekker, D. M.; Keene, S. T.; Vantomme, G.; Ehrler, B.; Meskers, S. C. J.; Rao, A.; Monserrat, B.; Meijer, E. W.; Friend, R. H. Circularly Polarized Electroluminescence from Chiral Supramolecular Semiconductor Thin Films. *Science* **2025**, *387* (6739), 1175–1181. https://doi.org/10.1126/science.adt3011.
(21) Pancotti, G.; Killalea, C. E.; Rees, T. W.; Liirò-Peluso, L.; Riera-Galindo, S.; Beton, P. H.; Campoy-Quiles, M.; Siligardi, G.; Amabilino, D. B. Film Thickness Dependence of Nanoscale Arrangement of a Chiral Electron Donor in Its Blends with an Achiral Electron Acceptor. *Nanoscale* **2025**, *17* (6), 3133–3144. https://doi.org/10.1039/D4NR04269G.
(22) Wasiluk, M.; Goldmann, C.; Bagiński, M.; Pawlak, M.; Majewski, PawełW.; Abramowicz, J.; Roszkowski, P.; Rebholz, L.; Rockstuhl, C.; Hamon, C.; Lewandowski, W. From Low Symmetry to High Dissymmetry: Chiral Plasmonic Films of Binary and Nanobipyramid Assemblies. *Adv. Funct. Materials* **2025**, *35* (45), 2500933. https://doi.org/10.1002/adfm.202500933.
(23) Ugras, T. J.; Gracias, D. J.; Arteaga, O.; Robinson, R. D. Realizing Nonreciprocal Linear Dichroism and Emission from Simple Media. arXiv **2025**. https://doi.org/10.48550/ARXIV.2507.23051.
(24) Albano, G.; Bertuolo, M.; Zinna, F.; Taddeucci, A.; Jávorfi, T.; Hussain, R.; Farinola, G. M.; Pescitelli, G.; Punzi, A.; Siligardi, G.; Di Bari, L. Unravelling the Origin of Strong Non-Reciprocal Chiroptical Features in Thin Films of a Chiral Diketopyrrolo[3,4-*c*]pyrrole Dye. *Nanoscale* 2025, *17* (9), 5128–5140. https://doi.org/10.1039/D4NR04956J.
(25) Gil, J. J.; Bernabeu, E. Depolarization and Polarization Indices of an Optical System. *Optica Acta: International Journal of Optics* **1986**, *33* (2), 185–189. https://doi.org/10.1080/713821924.
(26) Azzam, R. M. A. Propagation of Partially Polarized Light through Anisotropic Media with or without Depolarization: A Differential 4 × 4 Matrix Calculus. *J. Opt. Soc. Am.* **1978**, *68* (12), 1756-1767. https://doi.org/10.1364/JOSA.68.001756.
(27) Arteaga, O.; Canillas, A. Analytic Inversion of the Mueller-Jones Polarization Matrices for Homogeneous Media. *Opt. Lett.* **2010**, *35* (4), 559-561. https://doi.org/10.1364/OL.35.000559.
(28) Álvaro-Martins, M. J.; Billiaux, C.; Godard, P.; Oda, R.; Raffy, G.; Bassani, D. M. Strong Circularly Polarized Luminescence via Intramolecular Excited-State Symmetry-Breaking Charge Separation. *Chem. Commun.* **2023**, *59* (51), 7963–7966. https://doi.org/10.1039/d3cc01802d.
(29) Ito, S.; Ikeda, K.; Nakanishi, S.; Imai, Y.; Asami, M. Concentration-Dependent Circularly Polarized Luminescence (CPL) of Chiral N,N′-Dipyrenyldiamines: Sign-Inverted CPL Switching between Monomer and Excimer Regions under Retention of the Monomer Emission for Photoluminescence. *Chem. Commun.* **2017**, *53* (47), 6323–6326. https://doi.org/10.1039/c7cc01351e.
(30) Miki, K.; Noda, T.; Gon, M.; Tanaka, K.; Chujo, Y.; Mizuhata, Y.; Tokitoh, N.; Ohe, K. Near-Infrared Circularly Polarized Luminescence through Intramolecular Excimer Formation of Oligo(p-Phenyleneethynylene)-Based Double Helicates. *Chemistry – A European Journal* **2019**, *25* (39), 9211–9216. https://doi.org/10.1002/chem.201901467.
(31) Zhu, J.; Chen, S.; He, C. Catalytic Enantioselective Dehydrogenative Si–O Coupling to Access Chiroptical Silicon-Stereogenic Siloxanes and Alkoxysilanes. *J. Am. Chem. Soc.* **2021**, *143* (14), 5301–5307. https://doi.org/10.1021/jacs.1c01106.
(32) Reiné, P.; Justicia, J.; Morcillo, S. P.; Abbate, S.; Vaz, B.; Ribagorda, M.; Orte, Á.; Álvarez de Cienfuegos, L.; Longhi, G.; Campaña, A. G.; Miguel, D.; Cuerva, J. M. Pyrene-Containing Ortho-Oligo(Phenylene)Ethynylene Foldamer as a Ratiometric Probe Based on Circularly Polarized Luminescence. *J. Org. Chem.* **2018**, *83* (8), 4455–4463. https://doi.org/10.1021/acs.joc.8b00162.
(33) Takaishi, K.; Iwachido, K.; Ema, T. Solvent-Induced Sign Inversion of Circularly Polarized Luminescence: Control of Excimer Chirality by Hydrogen Bonding. *J. Am. Chem. Soc.* **2020**, *142* (4), 1774–1779. https://doi.org/10.1021/jacs.9b13184.
(34) Deb, D.; Parashar, M.; Sarkar, S.; Ghosh, S.; Balasubramanian, S.; George, S. J. Bright and Color-Tunable Circularly Polarized Excimer Emission from Pre-Organized, Naphthalene Diimide Molecular Designs. *Angewandte Chemie International Edition* **2026**, *65* (1), e202520385. https://doi.org/10.1002/anie.202520385.
(35) Hayashi, K.; Miyaoka, Y.; Ohishi, Y.; Uchida, T.; Iwamura, M.; Nozaki, K.; Inouye, M. Observation of Circularly Polarized Luminescence of the Excimer from Two Perylene Cores in the Form of [4]Rotaxane. *Chemistry – A European Journal* **2018**, *24* (55), 14613–14616. https://doi.org/10.1002/chem.201803215.

(36) Fu, R.; Chen, F.-Y.; Dai, L.; Li, D.-Y.; Zhao, Q.-Y.; Guo, S.-D.; Song, Z.-H.; Jing, L.-B.; Wang, H.; Li, X.; Guo, D.-S.; Cai, K. Chiral Macrocycles Forming High-Affinity Host-Guest Homoternary Complexes for Chiral Discrimination and Amplified Circularly Polarized Luminescence. *Nat. Commun.* **2025**, *16* (1), 9718. https://doi.org/10.1038/s41467-025-64739-7.
(37) Du, Y.-B.; Lu, Q.-T.; Cui, Y.-S.; Wu, K.-W.; Wang, Y.; Zhang, Y.-Z.; Zhao, Z.; Hou, J.-L.; Cai, Q. Enantioselective Synthesis of Atropisomeric Tri-Axis Naphthalenes via Diels–Alder Reaction and Dehydrative Aromatization of Isobenzofurans. *Angewandte Chemie International Edition* **2025**, *64* (10), e202421060. https://doi.org/10.1002/anie.202421060.
(38) Bao, L.-T.; Zhang, R.-H.; Yuan, X.; Wang, X.; Wu, P.; Wang, X.-Q.; Chen, J.; Zhu, A.; Yang, H.-B.; Wang, W. Rigidly Locked Pyrene Excimers in Planar Chiral Pyrenophanes for Intense and Stable Circularly Polarized Photoluminescence and Electrochemiluminescence. *Angewandte Chemie International Edition* **2025**, *64* (15), e202500472. https://doi.org/10.1002/anie.202500472.
(39) Kano, K.; Matsumoto, H.; Hashimoto, S.; Sisido, M.; Imanishi, Y. A Chiral Pyrene Excimer in γ-Cyclodextrin Cavity. *J. Am. Chem. Soc.* **1985**, *107* (21), 6117–6118. https://doi.org/10.1021/ja00307a055.
(40) Nishioki, K.; Kimura, A.; Ohishi, Y.; Yamashita, J.; Kitamoto, M.; Iwamura, M.; Nozaki, K.; Chiba, J.; Inouye, M. High-Yield Syntheses of Doubly 2,7-Dialkynylpyrene-Threaded Photostable [8]Rotaxanes Exhibiting Extremely Bright Circularly Polarized Luminescence. *Chem. Sci.* **2025**, *16* (43), 20414–20421. https://doi.org/10.1039/d5sc06304c.
(41) Wang, Y.; Gong, J.; Wang, X.; Li, W.-J.; Wang, X.-Q.; He, X.; Wang, W.; Yang, H.-B. Multistate Circularly Polarized Luminescence Switching through Stimuli-Induced Co-Conformation Regulations of Pyrene-Functionalized Topologically Chiral [2]Catenane. *Angewandte Chemie International Edition* **2022**, *61* (44), e202210542. https://doi.org/10.1002/anie.202210542.
(42) Inouye, M.; Hayashi, K.; Yonenaga, Y.; Itou, T.; Fujimoto, K.; Uchida, T.; Iwamura, M.; Nozaki, K. A Doubly Alkynylpyrene-Threaded [4]Rotaxane That Exhibits Strong Circularly Polarized Luminescence from the Spatially Restricted Excimer. *Angewandte Chemie International Edition* **2014**, *53* (52), 14392–14396. https://doi.org/10.1002/anie.201408193.
(43) Takaishi, K.; Murakami, S.; Yoshinami, F.; Ema, T. Binaphthyl-Bridged Pyrenophanes: Intense Circularly Polarized Luminescence Based on a D2 Symmetry Strategy. *Angewandte Chemie International Edition* **2022**, *61* (27), e202204609. https://doi.org/10.1002/anie.202204609.
(44) Zhou, Q.; Yuan, W.; Li, Y.; Han, Y.; Bao, L.; Fan, W.; Jiao, L.; Zhao, Y.; Ni, Y.; Zou, Y.; Yang, H.-B.; Wu, J. [5]Helicene Based π-Conjugated Macrocycles with Persistent Figure-Eight and Möbius Shapes: Efficient Synthesis, Chiral Resolution and Bright Circularly Polarized Luminescence. *Angewandte Chemie International Edition* **2025**, *64* (5), e202417749. https://doi.org/10.1002/anie.202417749.
(45) Han, D.; Li, C.; Jin, X.; Zhou, J.; Xu, Y.; Jiao, T.; Duan, P. Tunable Circularly Polarized Luminescence of Excited-State-Proton-Transfer-Based Chiral Guanidine. *Advanced Photonics Research* **2022**, *3* (3), 2100287. https://doi.org/10.1002/adpr.202100287.
(46) Salerno, F.; Berrocal, J. A.; Haedler, A. T.; Zinna, F.; Meijer, E. W.; Di Bari, L. Highly Circularly Polarized Broad-Band Emission from Chiral Naphthalene Diimide-Based Supramolecular Aggregates. *J. Mater. Chem. C* **2017**, *5* (14), 3609–3615. https://doi.org/10.1039/c7tc00281e.
(47) San Jose, B. A.; Matsushita, S.; Akagi, K. Lyotropic Chiral Nematic Liquid Crystalline Aliphatic Conjugated Polymers Based on Disubstituted Polyacetylene Derivatives That Exhibit High Dissymmetry Factors in Circularly Polarized Luminescence. *J. Am. Chem. Soc.* **2012**, *134* (48), 19795–19807. https://doi.org/10.1021/ja3086565.
(48) Nakamura, M.; Suzuki, J.; Ota, F.; Takada, T.; Akagi, K.; Yamana, K. Helically Assembled Pyrene Arrays on an RNA Duplex That Exhibit Circularly Polarized Luminescence with Excimer Formation. *Chemistry – A European Journal* **2016**, *22* (27), 9121–9124. https://doi.org/10.1002/chem.201602043.
(49) Guo, Y.; Zhang, Y.; Ma, J.; Liao, R.; Wang, F. Wide-Range Tunable Circularly Polarized Luminescence in Triphenylamine Supramolecular Polymers via Charge-Transfer Complexation. *Nat. Commun.* **2024**, *15* (1), 9303. https://doi.org/10.1038/s41467-024-53297-z.
(50) Watanabe, K.; Sakamoto, T.; Taguchi, M.; Fujiki, M.; Nakano, T. A Chiral π-Stacked Vinyl Polymer Emitting White Circularly Polarized Light. *Chem. Commun.* **2011**, *47* (39), 10996–10998. https://doi.org/10.1039/c1cc13711e.
(51) Snellenburg, J. J.; Laptenok, S. P.; Seger, R.; Mullen, K. M.; Stokkum, I. H. M. V. Glotaran: A *Java*-Based Graphical User Interface for the *R* Package TIMP. *J. Stat. Soft.* **2012**, *49* (3), 1-22. https://doi.org/10.18637/jss.v049.i03.

(52) Bressan, G.; Penty, S. E.; Green, D.; Heisler, I. A.; Jones, G. A.; Barendt, T. A.; Meech, S. R. Ultrafast and Coherent Dynamics in a Solvent Switchable “Pink Box” Perylene Diimide Dimer. *Angew. Chem. Int. Ed.* **2024**, *63* (39), e202407242. https://doi.org/10.1002/anie.202407242.
(53) Heitmüller, J.; Fröhlich, R.; Renner, R.; Würthner, F.; Brixner, T. Intersystem Crossing of Perylene Bisimide Neutral, Radical Anion, and Dianion Derivatives Compared *via* Ultrafast Spectroelectrochemistry. *Phys. Chem. Chem. Phys.* **2023**, *25* (26), 17214–17229. https://doi.org/10.1039/D2CP05602J.
(54) Alzola, J. M.; Tcyrulnikov, N. A.; Brown, P. J.; Marks, T. J.; Wasielewski, M. R.; Young, R. M. Symmetry-Breaking Charge Separation in Phenylene-Bridged Perylenediimide Dimers. *J. Phys. Chem. A* **2021**, *125* (35), 7633–7643. https://doi.org/10.1021/acs.jpca.1c05100.
(55) Bressan, G.; Hartmann, D.; Brouwer, J.; Braun, E. M.; Bull, J. N.; Barendt, T. A. Conformational Switching Modulates Excited-State Pathways in a Cofacial Perylene Dimer. *Chem. Sci.* **2026**, *17* (12), 5921–5933. https://doi.org/10.1039/D5SC09512C.
(56) Neese, F. Software Update: The ORCA Program System—Version 5.0. *WIREs Comput. Mol. Sci.* **2022**, *12* (5), e1606. https://doi.org/10.1002/wcms.1606.
(57) Bursch, M.; Mewes, J.; Hansen, A.; Grimme, S. Best-Practice DFT Protocols for Basic Molecular Computational Chemistry. *Angew. Chem. Int. Ed.* **2022**, *61* (42), e202205735. https://doi.org/10.1002/anie.202205735.
(58) Chai, J.-D.; Head-Gordon, M. Systematic Optimization of Long-Range Corrected Hybrid Density Functionals. *The Journal of Chemical Physics* **2008**, *128* (8), 084106. https://doi.org/10.1063/1.2834918.
(59) Weigend, F.; Ahlrichs, R. Balanced Basis Sets of Split Valence, Triple Zeta Valence and Quadruple Zeta Valence Quality for H to Rn: Design and Assessment of Accuracy. *Phys. Chem. Chem. Phys.* **2005**, *7* (18), 3297-3305. https://doi.org/10.1039/b508541a.
(60) Novoa, T.; Laplaza, R.; Peccati, F.; Fuster, F.; Contreras-García, J. The NCIWEB Server: A Novel Implementation of the Noncovalent Interactions Index for Biomolecular Systems. *J. Chem. Inf. Model.* **2023**, *63* (15), 4483–4489. https://doi.org/10.1021/acs.jcim.3c00271.
(61) Pracht, P.; Bohle, F.; Grimme, S. Automated Exploration of the Low-Energy Chemical Space with Fast Quantum Chemical Methods. *Phys. Chem. Chem. Phys.* **2020**, *22* (14), 7169–7192. https://doi.org/10.1039/C9CP06869D.
(62) Bannwarth, C.; Ehlert, S.; Grimme, S. GFN2-xTB—An Accurate and Broadly Parametrized Self-Consistent Tight-Binding Quantum Chemical Method with Multipole Electrostatics and Density-Dependent Dispersion Contributions. *J. Chem. Theory Comput.* **2019**, *15* (3), 1652–1671. https://doi.org/10.1021/acs.jctc.8b01176.
(63) Brandenburg, J. G.; Bannwarth, C.; Hansen, A.; Grimme, S. B97-3c: A Revised Low-Cost Variant of the B97-D Density Functional Method. *The Journal of Chemical Physics* **2018**, *148* (6), 064104. https://doi.org/10.1063/1.5012601.
(64) Hestand, N. J.; Spano, F. C. Expanded Theory of H- and J-Molecular Aggregates: The Effects of Vibronic Coupling and Intermolecular Charge Transfer. *Chem. Rev.* **2018**, *118* (15), 7069–7163. https://doi.org/10.1021/acs.chemrev.7b00581.
(65) Hestand, N. J.; Spano, F. C. Interference between Coulombic and CT-Mediated Couplings in Molecular Aggregates: H- to J-Aggregate Transformation in Perylene-Based π-Stacks. *The Journal of Chemical Physics* **2015**, *143* (24), 244707. https://doi.org/10.1063/1.4938012.
(66) Hong, Y.; Schlosser, F.; Kim, W.; Würthner, F.; Kim, D. Ultrafast Symmetry-Breaking Charge Separation in a Perylene Bisimide Dimer Enabled by Vibronic Coupling and Breakdown of Adiabaticity. *J. Am. Chem. Soc.* **2022**, *144* (34), 15539–15548. https://doi.org/10.1021/jacs.2c03916.
